# Categorical Perception of Fear and Anger Expressions in Whole, Masked and Composite Faces

**DOI:** 10.1371/journal.pone.0134790

**Published:** 2015-08-11

**Authors:** Martin Wegrzyn, Isabelle Bruckhaus, Johanna Kissler

**Affiliations:** 1 Department of Psychology, Bielefeld University, Bielefeld, Germany; 2 Center of Excellence Cognitive Interaction Technology (CITEC), Bielefeld University, Bielefeld, Germany; University of Lincoln, UNITED KINGDOM

## Abstract

Human observers are remarkably proficient at recognizing expressions of emotions and at readily grouping them into distinct categories. When morphing one facial expression into another, the linear changes in low-level features are insufficient to describe the changes in perception, which instead follow an s-shaped function. Important questions are, whether there are single diagnostic regions in the face that drive categorical perception for certain parings of emotion expressions, and how information in those regions interacts when presented together. We report results from two experiments with morphed fear-anger expressions, where (a) half of the face was masked or (b) composite faces made up of different expressions were presented. When isolated upper and lower halves of faces were shown, the eyes were found to be almost as diagnostic as the whole face, with the response function showing a steep category boundary. In contrast, the mouth allowed for a substantially lesser amount of accuracy and responses followed a much flatter psychometric function. When a composite face consisting of mismatched upper and lower halves was used and observers were instructed to exclusively judge either the expression of mouth or eyes, the to-be-ignored part always influenced perception of the target region. In line with experiment 1, the eye region exerted a much stronger influence on mouth judgements than vice versa. Again, categorical perception was significantly more pronounced for upper halves of faces. The present study shows that identification of fear and anger in morphed faces relies heavily on information from the upper half of the face, most likely the eye region. Categorical perception is possible when only the upper face half is present, but compromised when only the lower part is shown. Moreover, observers tend to integrate all available features of a face, even when trying to focus on only one part.

## Introduction

Facial expressions are a powerful means of conveying information about the emotional state of an individual. Recognizing and correctly interpreting these non-verbal signs is of vital importance for successful social interaction [1].

Faces are not only complex stimuli, but their expressions can often be subtle and ambiguous. Despite this, human observers can readily recognize expressions of emotions and assign them distinct labels. A large body of research suggests that there are a number of basic emotions which are correctly recognized by most observers [2], although their exact number is under debate [3]. Even when the expressions' intensity varies continuously, as in manipulated faces where one expression is gradually morphed into another, human observers readily group them into distinct categories with a high level of confidence [4].

Such categorical perception is demonstrated by identification tasks where linear changes in low-level features (e.g. basic visual properties like colour or shape) lead to non-linear changes in perception, which are best described by a sigmoid function with a steep category boundary. In general, categorical perception refers to the phenomenon that objects from the same category will be perceived as more similar than objects from different categories [5], despite being equally far apart from each other on a given physical dimension. One example is human colour perception, where two different wavelengths will only be perceived as two different colours when a category boundary is crossed [6]. For facial expressions, categorical perception has been demonstrated in a number of seminal studies [7–9].

Faces are inherently multidimensional stimuli, and in face processing categorical perception can relate to different dimensions (identity, age, attractiveness, gender, emotion), which in turn may depend on different facial features. Regarding emotion, each expression of a basic emotion can be described in terms of a number of muscle groups that are active when a person shows that particular emotion [10]. For example, fear will most frequently be expressed in the face by the brows raised and drawn together, the upper eyelids raised, the lower eyelids tensed, the lips stretched back and, in some cases, the mouth opened [11,12]. Furthermore, masking studies have demonstrated that each basic emotion is recognized through different diagnostic areas of the face [13]. For example, fear can be best recognized when the eyes are visible, while happiness is best inferred from the mouth region. On the other hand, eye tracking studies have shown that for all six basic expressions, including ones of low intensity, people focus primarily on the eyes [14]. While eye tracking studies shed light on how observers usually inspect faces under free viewing conditions, masking studies help to understand what information is indispensable in order to make a correct decision. Therefore a masking approach helps to make causal inferences about the diagnostic value of each facial feature.

To understand categorical perception of facial expressions, it is essential to know which information in the face is used to make a categorical decision and how facial features are integrated into a perceptive whole, so that successful categorization can occur.

The term 'holistic processing' refers to the fact that faces are preferentially processed as a whole [15–17]. Findings from the composite face illusion, where complementary upper and lower face halves are combined into a whole face, serve to illustrate this mechanism: It is considerably more difficult to make a decision about the properties of either half in such composites than it is to judge each part in isolation [18]. For example, it may be more difficult to recognize a person's identity from a lower face half when it has been combined with the upper face half of another person, while the same task is easy when each half is presented in isolation [18]. Using happy and angry faces, interference from composites with mis-matching expressions have been found reflected in slower reaction times [19,20].

So far, both masking studies [13] and composite face paradigms [19] used full-blown facial expressions instead of gradually morphed faces. Chen and Chen [21] have used morphed happy-sad expressions in a composite face, but always asked to judge the whole face and not its parts, therefore making it difficult to draw inferences on how the perception of one facial feature is influenced by another one.

Masking or composite face studies with morphed faces however allow to better describe human behaviour in psychophysical terms, as the subjective perception dissociates from the changes in low-level features. They also allow to better understand how we process subtle and ambiguous expressions, which are arguably most relevant in daily life.

Therefore, it is useful to investigate the relationship of categorical perception and featural (i.e. focusing on single parts) or configural (i.e. integrating parts into a whole) face processing with masked and composite faces. Main questions are, whether there are diagnostic regions in the face that are sufficient to allow for categorical perception, and whether multiple features need to be integrated when making categorical judgements. Therefore, to better understand mechanisms of categorical perception of facial expressions, a promising approach is to break down the face into its features and understand how each contributes to identifying a certain expression. In a second step, the features can be re-assembled into a whole and one can investigate how they are integrated to infer an expression from a whole face.

To address these questions, two experiments with faces morphed from fear to anger were performed. Beginning with the seminal work by Etcoff and Magee [9], fear-anger pairings have been frequently used for investigating categorical perception (e.g. [7,8]; cf. [5]). Previous masking studies with full-blown fear and anger expressions have shown that both expressions are mainly recognized from the eye region [13] and studies with neurological patients also point to a special role of the eyes for recognizing fear [22]. Therefore, one can hypothesize that categorical perception of fear and anger will rely mostly on the features in the upper half of the face. In line with research on the composite face illusion [19,20], the more informative half of the face should also dominate the perception of a full face.

In the present study, the first experiment was carried out to investigate how observers make categorical decisions from morphed faces when only a limited amount of features is present. In this experiment, an upper face half, a lower face half, or an intact face were shown and observers were asked to categorize each morph as being either angry or fearful. The second experiment investigated how this relates to performance in a composite face task, when participants have to ignore one half of the face to make an optimal decision. In this experiment, a face assembled of an upper and lower half was presented. The observers had to judge only one half at a time, while the distractor half was showing either full-blown anger of fear. Together, these experiments aim to elucidate the psychophysics of recognizing facial expressions.

## Experiment 1

### Participants

30 participants took part in the experiment (22 female). Mean age of participants was 25 years (range: 18–32). Participants received course credit or 5 EUR for participation. Participants reported no history of neurological or psychiatric illness and had normal or corrected-to-normal vision. The study was approved by the ethics board of Bielefeld University (Ethic Statement Nr. 2014–010). All participants gave oral informed consent before taking part in the experiments.

Two participants showed performance close to guessing for all conditions combined with exceptionally fast reaction times, indicative of non-compliance. Their data were excluded, leaving 28 participants for further analysis.

### Material

Anger and fear pictures of 20 identities (10 male, 10 female) were selected from the KDEF [23] and NimStim [24] databases. The pictures were used to generate morphs in 9 steps with Gimp 2.6 (www.gimp.org) and the GAP toolbox, resulting in 11 morphing grades including the original images. Faces were divided into an upper and lower half, with the border defined as being above the nostrils at the bridge of the nose of the middle morph. This border was defined for each face identity and subsequently applied to all of its morphs.

**Fig 1:**
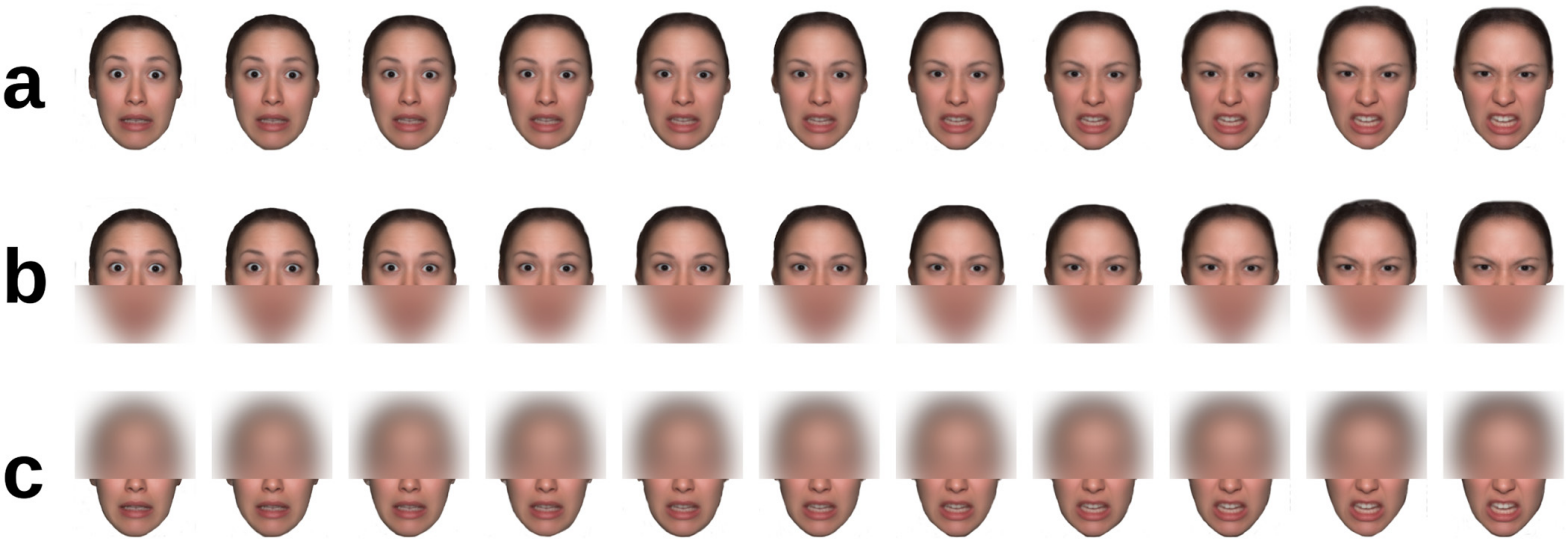
Design of Experiment 1. Illustration of a face morphed from the original fearful (outer left) to the original angry expression (outer right) in 9 intermediary steps, resulting in a total of 11 face morphs; a, whole face; b, upper half intact ('eyes' condition); c, lower half intact ('mouth' condition); due to copyright restrictions, the depicted example is an in-house generated averaged face based on 16 different identities and not depicting an actual person. This example is a representative illustration of the type of stimuli used but was not itself part of the present experiment.

In Experiment 1 there were three visibility conditions (whole face, upper half, lower half). The half of no interest was blurred with a very broad Gaussian filter, preserving only a rough outline of the face, thereby conveying a feeling of wholeness, while rendering the masked features invisible (cf. Fig 1).

### Design

A two-alternatives forced choice identification task was used, in which participants had to decide for each face whether its expression was 'angry' or 'fearful'. Each of the 20 identities was presented in 11 morphing grades. The experiment consisted of two runs with a total of 40 trials per cell, resulting in a resolution of 2.5% for identification performance. The first run was repeated after a short break, to allow for analyses of intra-subject stability. This resulted in a total of 440 trials per masking condition (11 morphs x 20 identities x 2 runs).

Pictures were shown with no time limit until a response was given, after which the next stimulus was shown directly, to allow for smooth work-flow. Participants were able to monitor their progress on a status bar presented at the bottom of the screen. Order of stimuli was randomized, the only constraint being that two subsequent trials never contained the same face identity.

Participants had to press the left or right mouse button to indicate whether the target face part showed an angry or fearful expression (button assignment counterbalanced between participants). Experiments were performed using Presentation software (Version 17.1, www.neurobs.com). All experiment files, including raw data, can be found in S8 Code and S1 Dataset.

### Analysis

To characterise the participants' performance, a logistic function was fitted to their data. The logistic function (F_logistic_(x;α,β) = 1/ [1+exp(-β(x-α))] ) is well-suited to describe observers' performance in psychometric terms ([25], p. 82). An s-shaped logistic function with a steep slope is indicative of high precision in distinguishingg between two categories and little uncertainty ([25],p. 20). If categorical perception occurs, the steepest point in the psychometric curve corresponds to the category boundary, as this is the point where two stimuli will be discriminated best [5]. However, it should be noted that the logistic function might occur for any linear system limited by Gaussian noise [26], including decisions based on simple image features such as contrast [27].

To derive the point of steepest increase, the limits of the logistic function were always defined by the lowest and highest value of the raw data, so that it provided an optimal fit even in cases when responses never crossed 50% guessing (cf. Experiment 2). Functions were fitted using an iterative optimization strategy, employing a non-linear least-squares procedure to derive the best-fitting function for the raw data. The steepest point of each curve was defined by computing the first derivative of the function. The position of this point on the x- and y-axis (threshold) as well as its value (slope) were then compared between conditions.

Furthermore, for cross-validation, data from one half of the experiment were used to predict the other half of the data. Since each participant performed every trial twice, data from the first half of the experiment could be used to predict the data from the second half (and vice versa). For each condition, one half of the participants' data was compared to all conditions of the other half using sums of squared errors (ss^2^). This allowed assessing whether results were stable across experiment repetitions. The more similar two conditions are, the smaller the ss^2^ should be. Using sums of squared errors has the advantages of being an intuitive measure of similarity and being virtually assumption-free ([28], p. 106).

We also investigated whether left-out data can be better predicted by using raw data or by a logistic function fitted to these data. If a logistic function provides a superior fit to left-out raw data (smaller ss^2^), this should indicate that the simplifying assumptions made by the psychometric function are sensible and add to our understanding of the underlying perceptual mechanisms.

Data analysis was performed with Python 2.7.9 (www.python.org) using NumPy, SciPy, Pandas, Matplotlib, and the IPython Notebook, all as provided with Anaconda 2.2.0 (Continuum Analytics; docs.continuum.io/anaconda). Full code and output for all analyses can be found in the online supplement. Refer to S1 and S2 Codes for data import and restructuring; S3 Code for main data analysis; S4 Code for fitting psychometric functions; S5 Code for cross-validation; and to S6 Code for analyses of single face identities. Analyses of Variance (ANOVA) for repeated measure designs were additionally carried out using IBM SPSS 22.

### Results

#### Comparison of response curves for different masking types

**Fig 2:**
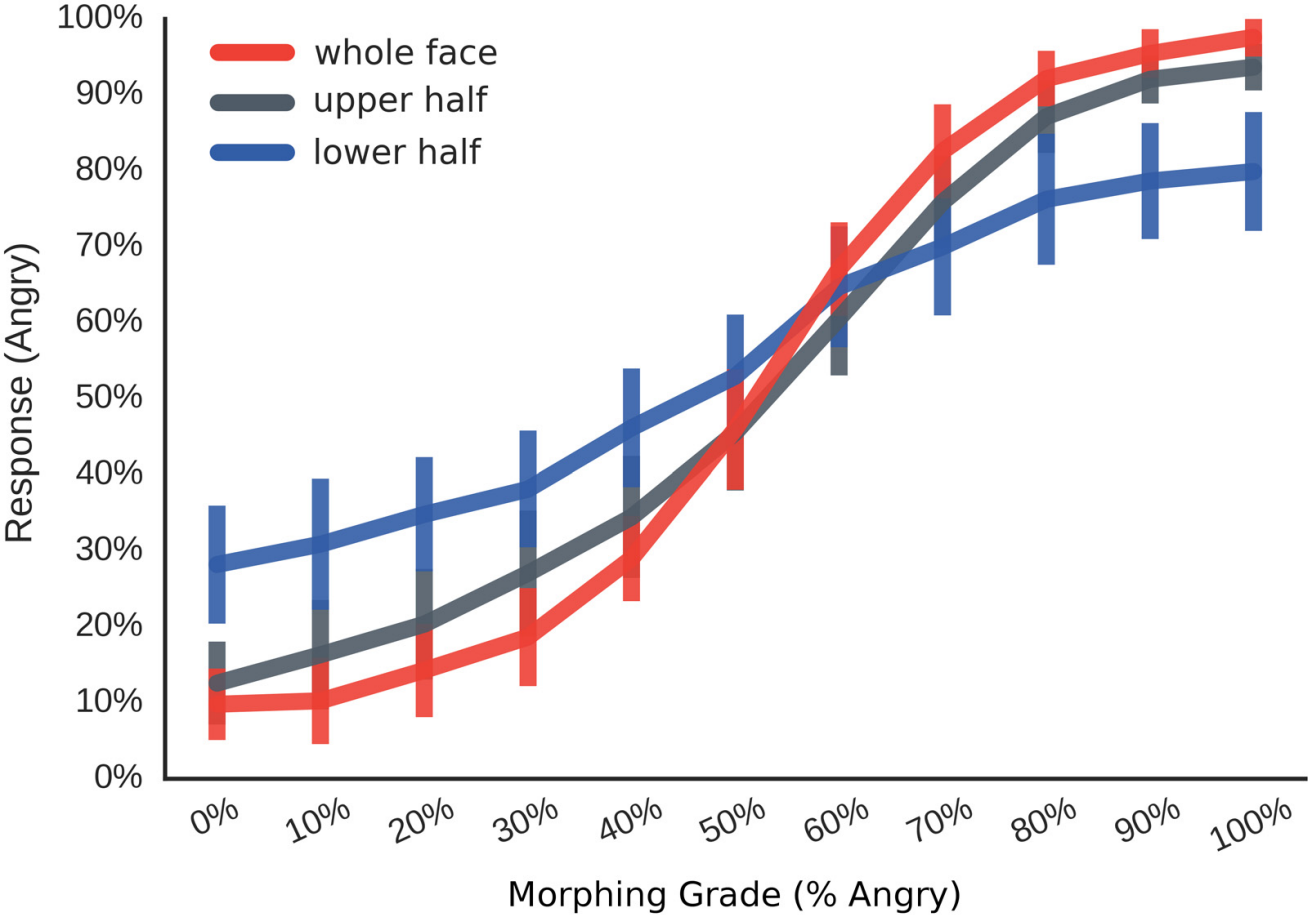
Main Results Experiment 1. Percent angry responses (y-axis) across the 11 morphing grades morphed from fear to anger (x-axis) for the three different visibility conditions (coloured lines); 'whole face', whole face is visible (no masking); 'lower half', lower face half is visible (upper half is masked); ‘upper half', upper face half is visible (lower half is masked).

Identification performance was compared across all 11 morphing grades for the three masking conditions (full face, upper face half, lower face half). Participants’ performance is illustrated in Fig 2. A 3x11 repeated-measures ANOVA showed a significant main effect of morphing grade, reflecting that anger responses increase as the faces are morphed towards this expression (*F*_(10,270)_ = 620.7, *p*<0.001, *η*_*p*_^*2*^ = 0.96). There was no main effect of masking condition (*F*_(2,54)_ = 2.3, *p* = 0.112, *η*_*p*_^*2*^ = 0.08), indicating that there is no bias that would shift the responses between conditions. A significant condition-by-morphing grade interaction (*F*_(20,540)_ = 63.5, *p*<0.001, *η*_*p*_^*2*^ = 0.70) indicated that the shape of the response curve differs between conditions. Paired t-tests for repeated measures and the Wilcoxon signed rank test as its non-parametric equivalent were carried out to test differences between conditions for all morphing grades. Results were only considered significant if the p-value for both metrics fell below *α* = 0.05.

Identification performance was best (i.e. significantly farthest from guessing) for the whole face, compared both to the upper (all *ps*<0.05, except 50% angry morphs) and lower half (all *ps*<0.05, except 60% angry morphs). Furthermore, performance was significantly better for the upper than for the lower half (all *ps* < .05 except for 50% and 60% angry morphs). This indicates that observers were able to make the most accurate decisions when presented with a whole face, followed by face halves containing only the eye region. Performance was worst when only the mouth region was visible.

Judgements of the eye region closely resembled the whole face responses, as indicated by a significantly smaller difference between upper and whole face decisions compared to lower and whole face decisions (all *ps*<0.05 for pairwise comparisons of differences, except for 50% and 60% morphs).

#### Cross-validation of conditions

If differences between conditions are genuine and stable, each condition should be able to predict itself best, e.g. the whole face condition of the first half of the experiment should be most similar to the whole face condition of the second half.

This was confirmed by split-half cross-validation with each half of the experiment used once as test and as training set, within each participant. Each condition was significantly closest to its analogue form the other half of the experiment, as expressed in significantly smallest ss^2^ for these pairings (all *p*<0.01 for pairwise comparisons).

Furthermore, the upper and whole face conditions were significantly more similar to each other than to the lower half condition (*p*<0.01). This stronger similarity of the eye region to the whole face ratings further corroborates that the upper half carries more information about fear and anger expressions than does the mouth region.

#### Comparison of thresholds and slopes of the logistic function between conditions

**Fig 3:**
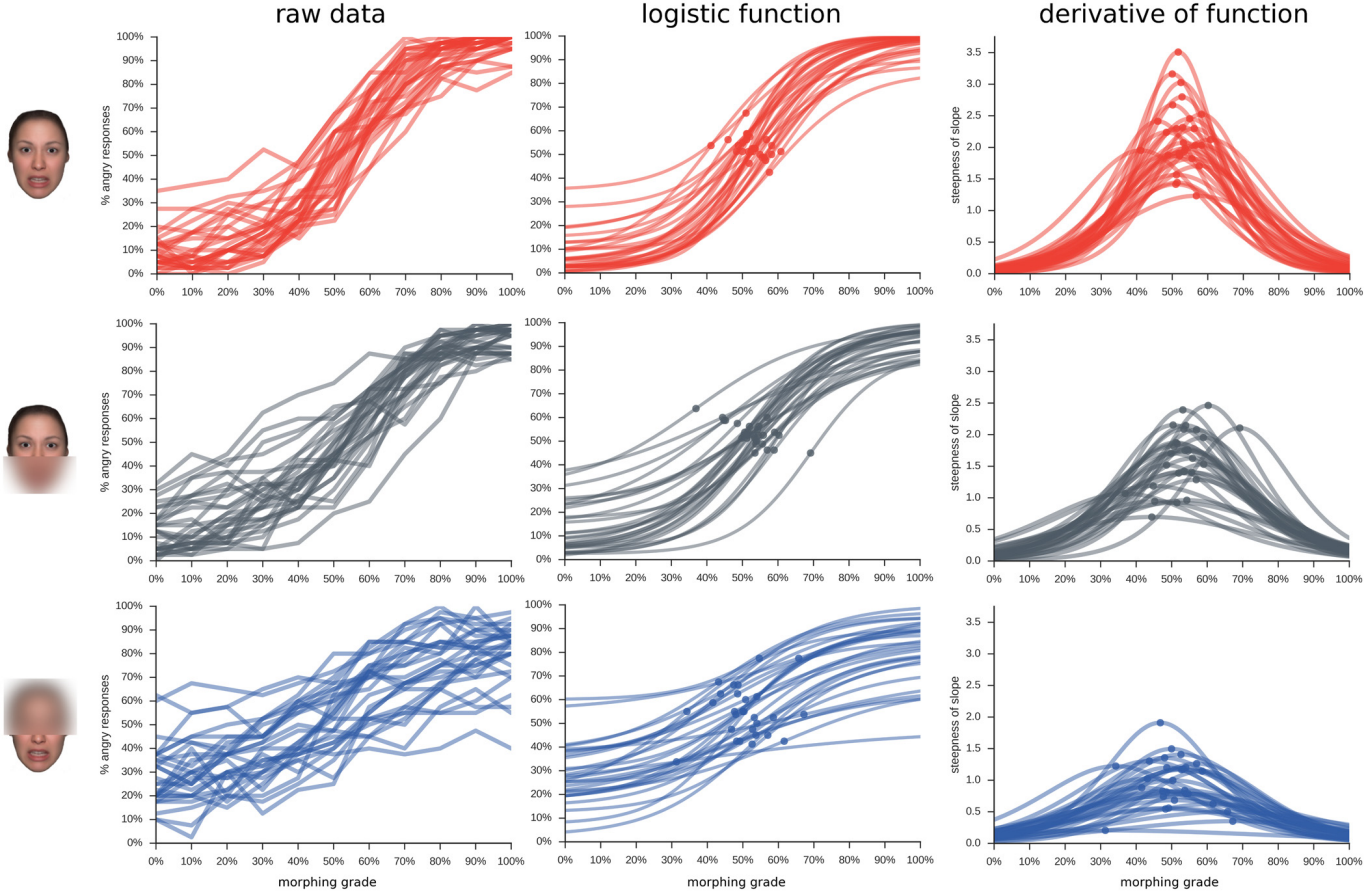
Data on single participant level. Each line represents a participant; each row of the figure indicates a masking condition, as illustrated by the example stimulus on the left-hand side. Each column indicates a different metric describing the data: 'raw data', the original data for each participant; 'logistic function', the best-fitting logistic function for each individual participant, with point of steepest slope indicated by a dot; 'derivative of function', the slope of the fitted logistic function, with the highest value indicated by a dot.

When fitting a logistic function to each participant’s data for each condition, the steepness of the slopes can be compared as measure for the presence of a category boundary, with a steep slope indicating stronger categorical perception (Fig 3).

**Fig 4:**
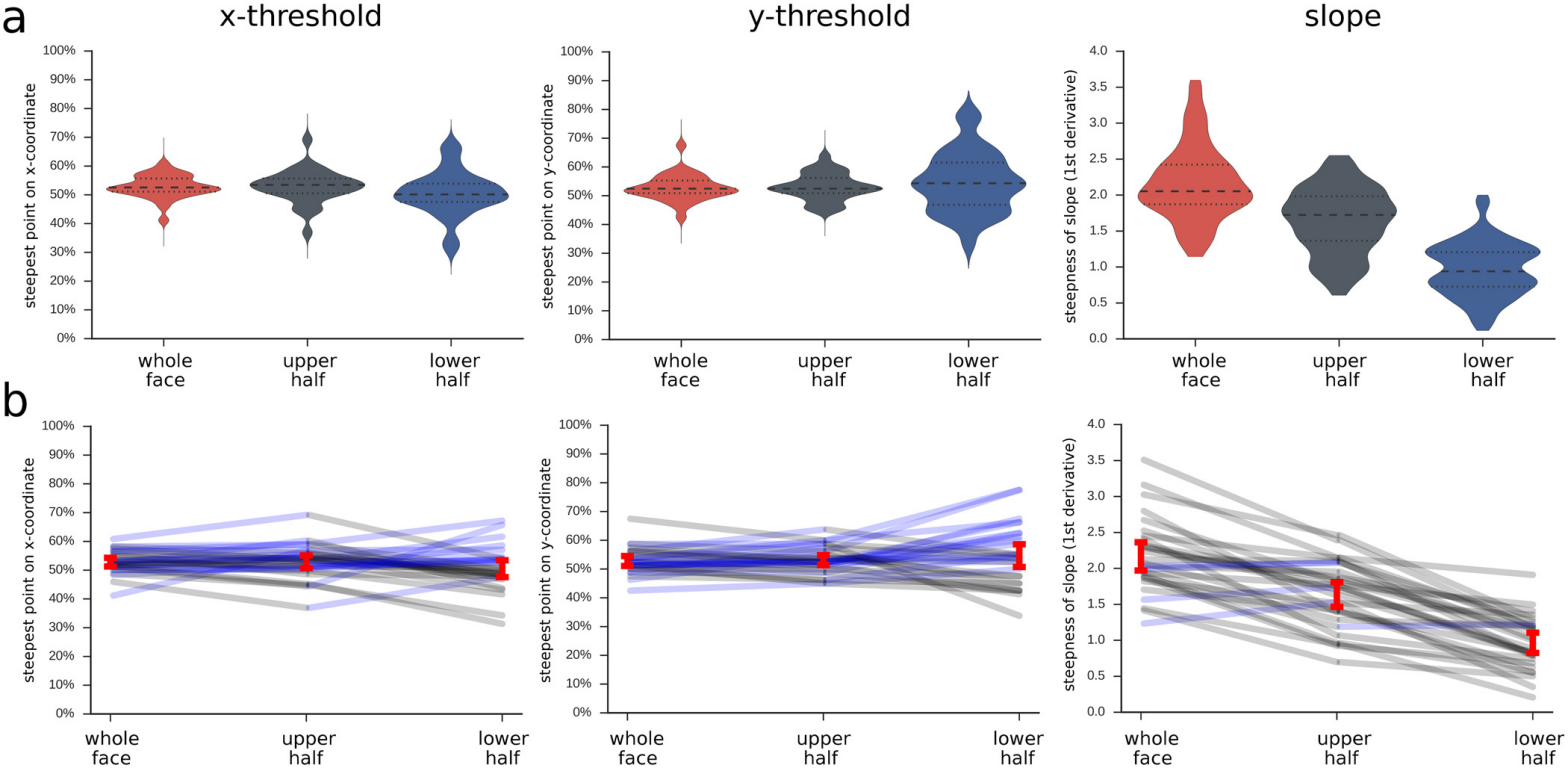
Parameters of logistic function for different masking conditions. a, violin plots with width indicating density of values, inner lines indicating median and 25% and 75% quartiles; b, red bars indicate 95% confidence intervals of the mean for each condition; line graphs in the background depict the raw data of each participant; black lines indicate that the participant's value for the left-hand condition is numerically bigger than the right-hand value; blue line indicates that the value is smaller for the left-hand compared to right-hand condition; 'whole face', whole face is visible (no masking); 'upper half', upper face half is visible (lower half is masked); 'lower half', lower face half is visible (upper half is masked).

Results show that the logistic functions for all conditions had their thresholds close to 50% guessing, with no significant differences between conditions (Table 1, Fig 4). There was a clear effect for the slope, which was steepest for the whole face condition, followed by the upper and lower half (Table 1, Fig 4; all *ps*<0.001 for pairwise comparisons), indicating a more abrupt shift from fear to anger responses when the whole face is visible and more smooth shifts in responses when viewing the lower face half.

**Table 1:** Comparison of curve parameters for experiment 1.

condition	mean (SD)			inferential statistics		
	whole face	upper half	lower half	F	p	n_p_²
**x-threshold**	0.53^a^ (0.04)	0.53^a^ (0.06)	0.51^a^ (0.08)	1.83	0.180	0.064
**y-threshold**	0.53^a^ (0.05)	0.53^a^ (0.05)	0.55^a^ (0.11)	0.77	0.415	0.028
**slope**	2.17^a^ (0.53)	1.64^b^ (0.47)	0.96^c^ (0.38)	105.30	**<0.001**	0.796

Results of a repeated measures ANOVA for the different curve parameters; means in the same row sharing the same superscript letter do not differ significantly from one another at α = 0.05; degrees of freedom (df) for x-threshold: 1.5,41.6 (Greenhouse-Geisser corrected); df for y-threshold: 1.3, 33.9 (Greenhouse-Geisser corrected); df for slope: 2,54.

#### Cross-validation of response curves for different masking types

Left-out data from one half of the experiment were predicted using data from the other half of the experiment with the two runs of the experiment used for cross-validation. Each condition was modelled for one half of the experiment using raw data or a logistic function fitted to these data. These training sets were then compared to the left-out half of the experiment to check how the models compare to each other in predicting new data.

The results of the cross-validation analysis show that in fact the logistic function fits left-out raw data significantly better than the raw data does (p<0.001 for all conditions), indicting its suitability for describing the participants' performance.

## Experiment 2

### Methods

Mean age of participants was 24 years (range 19–29). Of the 30 participants, 24 were female. Participants received course credit or 7EUR for participation and gave oral informed consent before starting the experiment.

**Fig 5:**
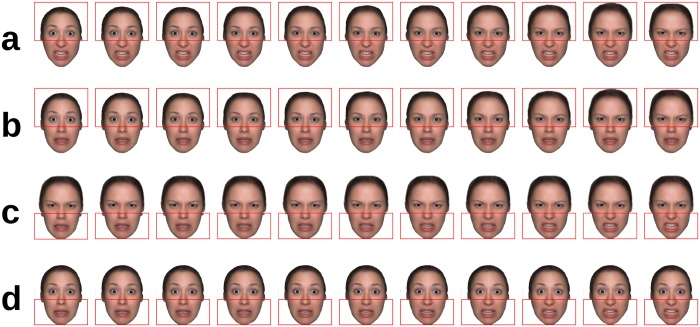
Design of Experiment 2 (Composite Face). Illustration of a face morphed from the original fearful (outer left) to the original angry expression (outer right) in 9 intermediary steps, resulting in a total of 11 face morphs; a, eye judgements with 100% angry lower face half; b, eye judgements with 100% fearful lower half; c, mouth judgements with 100% angry upper face half; d, mouth judgements with 100% fearful upper half; conditions a and b or conditions c and d were always presented in one block, to aid participants in focusing on one face half only; due to copyright restrictions, the depicted example is an in-house generated face which was not used in the present experiment.

The same boundary to divide faces into lower and upper halves as in experiment 1 was used and design choices were identical to experiment 1, with the following exceptions: This time 100% fearful or 100% angry expression halves were combined with the morphed pictures to create the composite face illusion. There were four conditions: 'lower half 100% angry' (Fig 5a), 'lower half 100% fearful (Fig 5b), 'upper half 100% angry' (Fig 5c) and 'upper half 100% fearful' (Fig 5d). The complementary half was presented in 11 morphing steps, and was the part which the observers were asked to identify as either angry of fearful. This part was always framed by a red square (cf. Fig 5). Participants were explicitly instructed to focus only on the framed face half and to ignore the other half. Unlike experiment 1, order of conditions was blocked, to make it easier for the participants to focus on the part of interest. Each block required either always judging the upper half or always judging the lower half, and consisted of 440 trials, with block order counterbalanced between participants. Within blocks, order of the 20 face identities, the 11 morphed face halves and whether the to-be-ignored half was fearful or angry were randomised.

One participant reported to have only focused on the mouth region throughout the experiment, ignoring which face half was currently framed. These data were excluded, leaving 29 data sets for analysis.

### Results

#### Comparison of response curves for different composite faces

**Fig 6:**
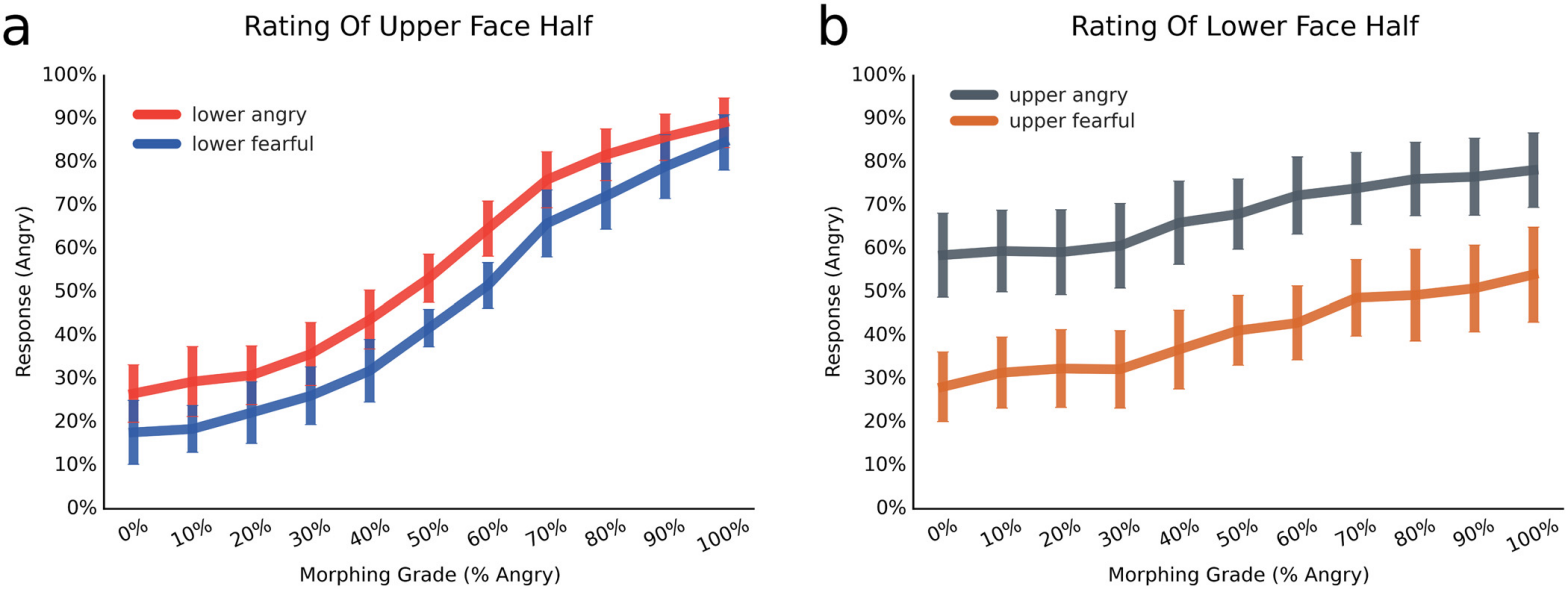
Main Results for Composite Faces. Percent angry responses (y-axis) across the 11 morphing grades morphed from fear to anger (x-axis) for the four different composite face conditions (coloured lines); 'lower fearful', observers judge the upper half, while the lower half is always 100% fearful; 'lower angry', observers judge the upper half, while the lower half is always 100% angry; 'upper fearful', observers judge the lower half, while the upper half is always 100% fearful; 'upper angry', observers judge the lower half, while the upper half is always 100% angry.

**Fig 7:**
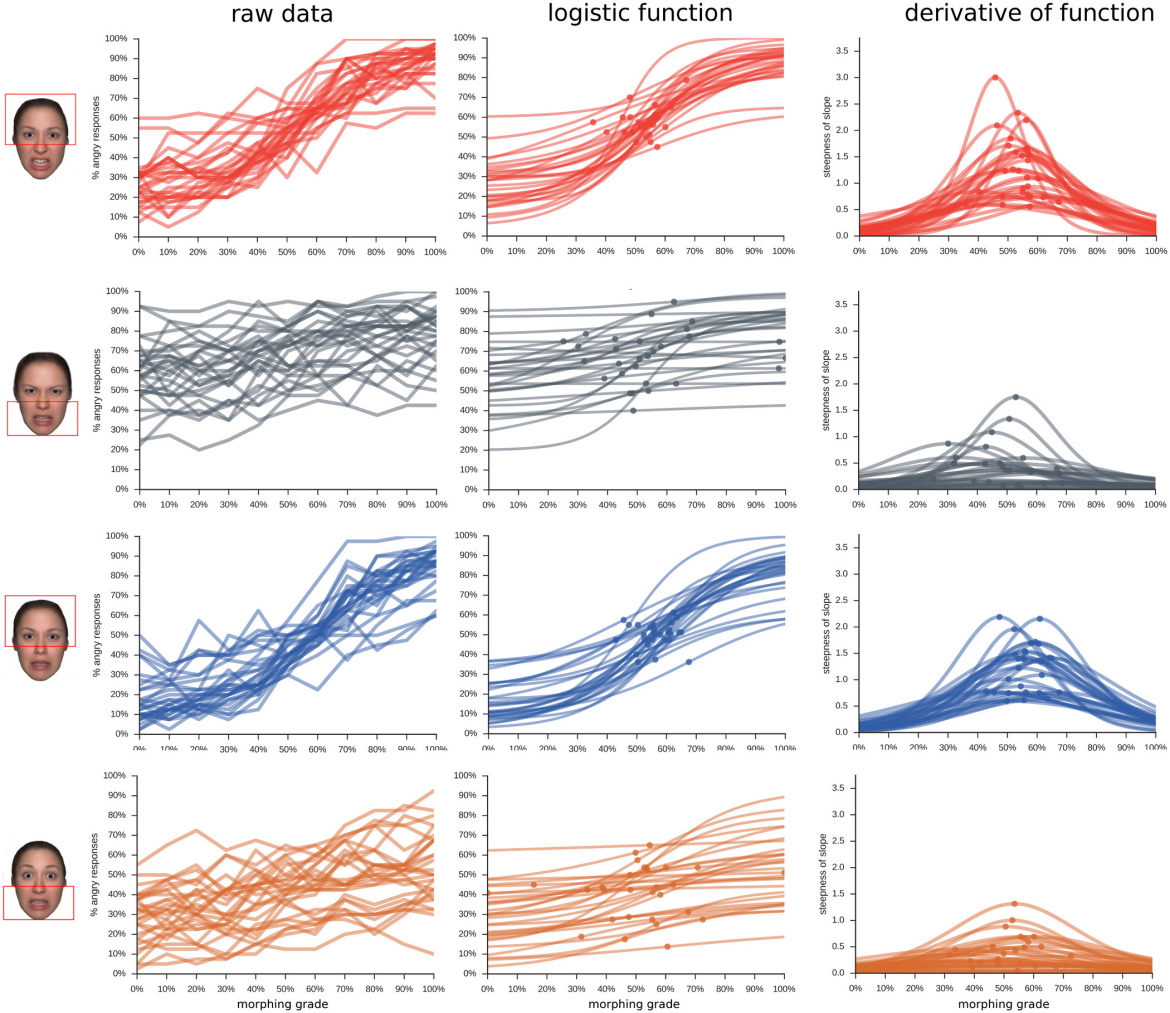
Data on single participant level. Each line represents a participant; each row of the figure indicates a composite face condition, as illustrated by the example stimulus on the left-hand side. Each column indicates a different metric describing the data: 'raw data', the original data for each participant; 'logistic function', the best-fitting logistic function for each individual participant, with point of steepest slope indicated by a dot; 'derivative of function', the slope of the fitted logistic function, with the highest value indicated by a dot.

Depending on the viewing condition, responses for the attended face half were biased in the direction of the to-be-ignored face half (Figs 6 and 7).

A 2x2x11 ANOVA with the factors upper/lower face half, anger/fear distractor and morphing grade (fear to anger) revealed a main effect of morphing grade (*F*_(10,280)_ = 213.7, *p*<0.001, *η*_*p*_^*2*^ = 0.88), a main effect of emotion (*F*_(1,28)_ = 109.6, *p*<0.001, *η*_*p*_^*2*^ = 0.80) and significant interactions between all factors (all *ps*<0.001). The main effect of emotion in the distractor half reflects that responses for the eye region were shifted towards anger when the lower face half was angry, and towards fear when the lower half was fearful (*p*<0.01 for all 11 morphing grades). The same bias occurred for the judgements of the mouth region, when the upper half was either angry or fearful (Fig 6; all *p*<0.001).

The interaction effect between emotion and face half (*F*_(1,28)_ = 26.0, *p*<0.001, *η*_*p*_^*2*^ = 0.48) reflects that the bias was significantly stronger for mouth judgements than for eye judgements, indicating that the upper face half exerts a stronger influence on the lower half than vice versa. Across all 11 morphing grades, fearful and angry distracting halves led to bigger differences in mouth judgements, compared to the differences in eye judgements (all *p*<0.001, for pairwise comparisons of differences).

#### Cross-validation of conditions

Split-half cross-validation with each half of the experiment used once as test and as training set showed that each condition was significantly closest to its analogue form the other half of the experiment (all *p*<0.01 for pairwise comparisons), indicative of reliable differences between conditions.

#### Comparison of thresholds and slopes of the logistic function between conditions

**Fig 8:**
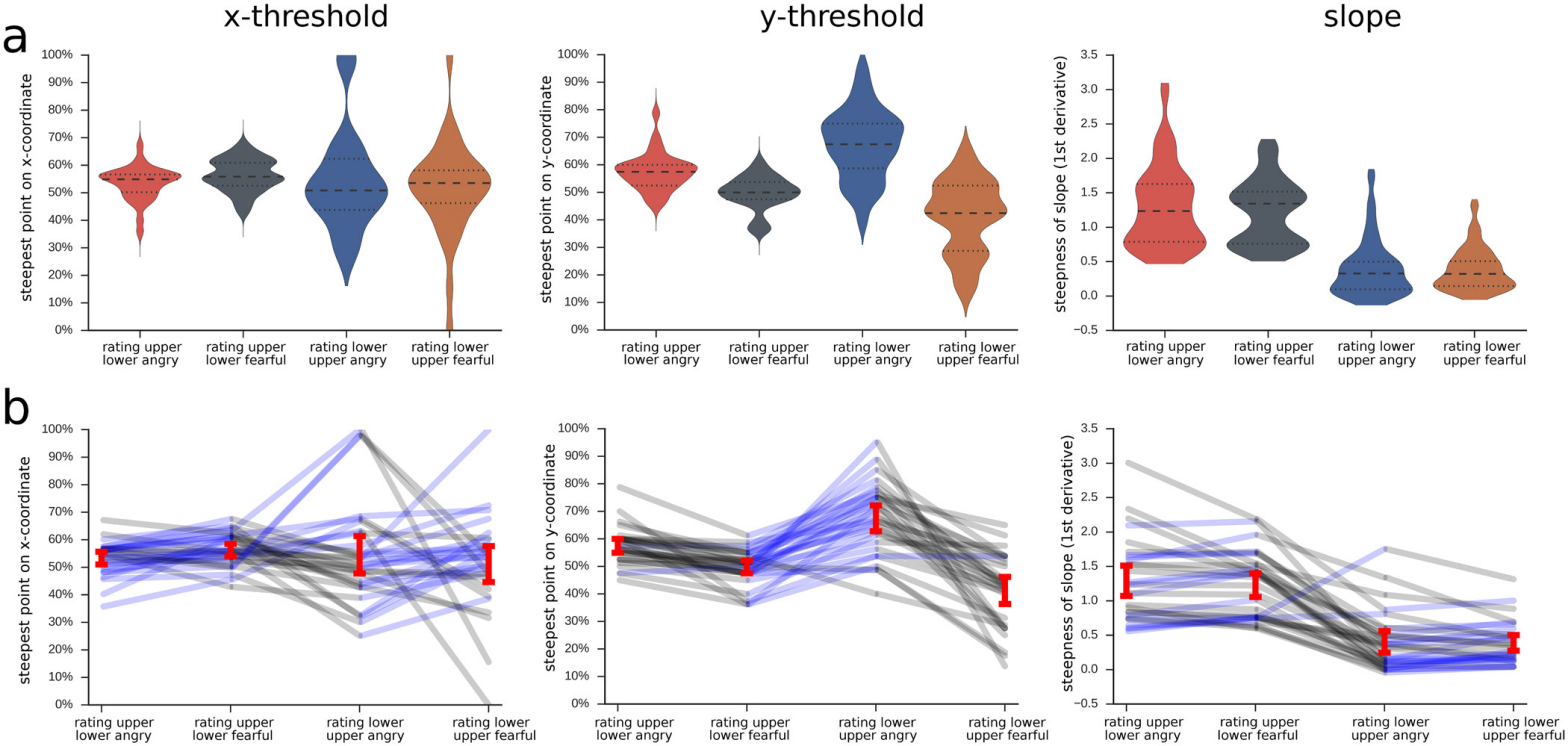
Parameters of logistic function for different composite face conditions. a, violin plots with width indicating density of values, inner lines indicating median and 25% and 75% quartiles; b, red bars indicate 95% confidence intervals of the mean for each condition; line graphs in the background depict the raw data of each participant; black lines indicate that the participant's value for the left-hand condition is numerically bigger than the right-hand value; blue line indicates that the value is smaller for the left-hand compared to right-hand condition.

As in experiment 1, the steepest point of the psychometric function was located around the intermediary morph (50% fear/50% anger). However, responses were significantly shifted on the y-axis for the different viewing conditions, indicating that there was a response bias towards fear or anger, depending on the expression of the to-be-ignored half (Table 2). When the to-be-ignored half was more fearful, the threshold was shifted towards fear, and when that half was more angry, responses were shifted towards anger. This main effect was accompanied by a significant interaction, reflecting that this bias was much strongest when the lower face half had to be rated and the upper face was the distractor (Figs 7 and 8, Table 2). Also, when judging the lower face half, the psychometric functions were almost flat, while they had a steep slope when the upper half had to be judged (Fig 7, Table 2).

**Table 2:** Comparison of curve parameters for experiment 2.

condition	mean (SD)				contrast								
	rating of upper half		rating of lower half		face half			emotion			interaction		
	lower angry	lower fearful	upper angry	upper fearful	F_(1,28)_	p	n_p_^2^	F_(1,28)_	p	n_p_^2^	F_(1,28)_	p	n_p_^2^
**x-threshold**	0.53^a^ (0.06)	0.56^b^ (0.06)	0.55^a,b^ (0.19)	0.51^a,b^ (0.18)	0.77	0.389	0.03	0.01	0.939	<0.01	1.44	0.241	0.05
**y-threshold**	0.57^a^ (0.07)	0.50^b^ (0.06)	0.67^c^ (0.13)	0.41^d^ (0.14)	0.22	0.624	0.01	100.23	**<0.001**	0.78	24.67	**<0.001**	0.47
**slope**	1.29^a^ (0.61)	1.23^a^ (0.47)	0.40^b^ (0.43)	0.39^b^ (0.31)	69.31	**<0.001**	0.71	0.18	0.178	0.06	0.54	0.542	0.01

Results of a repeated measures ANOVA for the different curve parameters of experiment 2; means in the same row sharing the same superscript letter do not differ significantly from one another at α = 0.05.; 'face half', main effect of face half which is rated; 'emotion', main effect of whether the to-be-ignored half is fearful or angry; 'interaction', interaction between the two factors.

#### Cross-validation of response curves for different masking types

Analogous to experiment 1, results of the cross-validation analysis show that the logistic function was better able to fit left-out raw data then the raw data it was based on (*p*<0.001 for all conditions), indicating that fitting a logistic function to the present data is suitable. Again, this might indicate that the fitting of a logistic function reduces irrelevant noise in the raw data and is therefore better able to generalize to new measurements.

## Results for Single Face Identities

Since 20 different face identities were used as stimuli in the experiments, the present results may underestimate or blur the sigmoid shape of the response functions, due to averaging over a heterogeneous set of stimuli with shifted thresholds.

**Fig 9:**
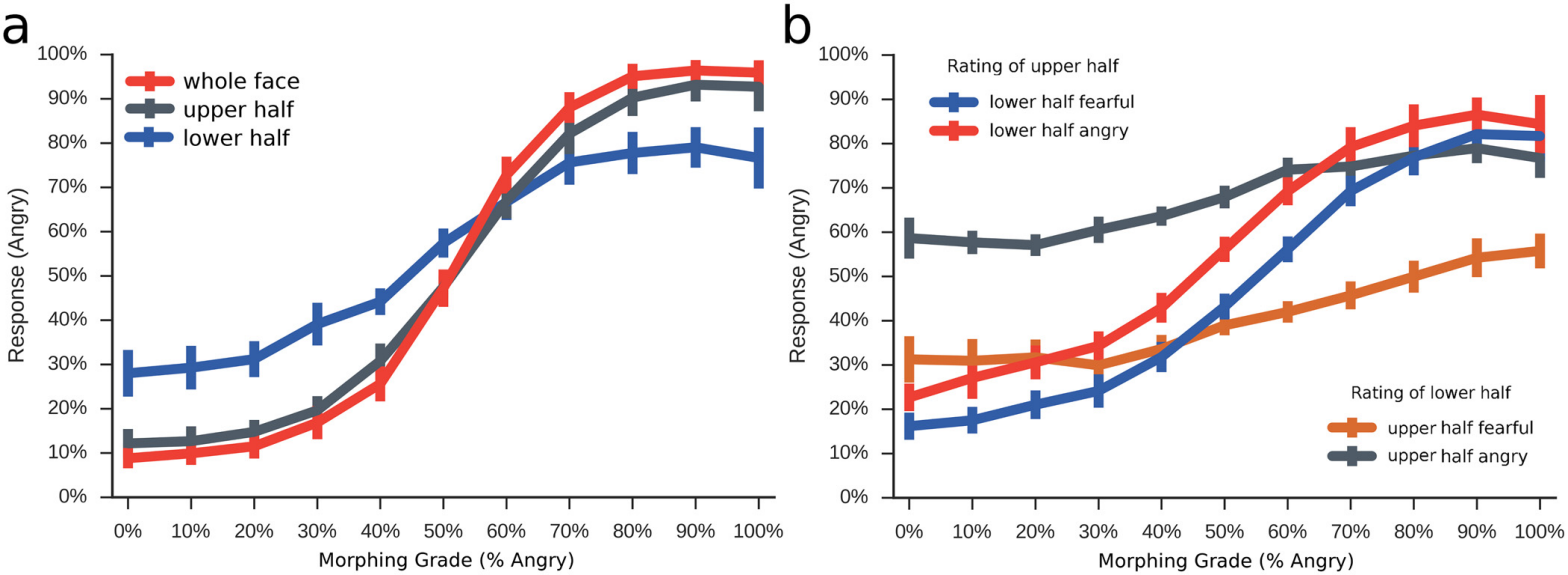
Correction for different thresholds between face identities. Main analyses for both experiments after modelling responses to each face identity individually and shifting the thresholds to be at the respective group mean; a, results for experiment 1 (masking); b, results for experiment 2 (composite faces); note that a different number of data points is available for each bin on the x-axis, as functions may be partly shifted out of the displayed range, rendering estimates at the extremes less reliable; also, variability is modelled at the stimulus level, not at participant level; please refer to S6 Code for more details on the analysis procedure.

Therefore, data of both experiments were re-analysed by fitting a logistic function to the responses to each of the 20 individual face identities, averaged across participants. Subsequently, the thresholds were shifted to be uniformly at the mean value of the respective condition, so that all curves share the x- and y-threshold and only differ in their (unchanged) slopes. The necessary shift determined from the fitted logistic functions was then applied to the underlying raw data, allowing to compare how the curves would be shaped, given equal thresholds for all face identities. The results are illustrated in Fig 9 and are described in-depth in S6 Code. Visual inspection of the data suggests that a sigmoid function clearly emerges only for whole faces and upper halves in experiment 1 and for upper halves in experiment 2 when accounting for potential variability between faces, thereby closely replicating the above results and demonstrating their generalizability across many stimuli.

## Discussion

The present study set out to investigate what part of the face is most diagnostic for categorical perception of fear-anger morphed expressions, and how this information is integrated when presented in a composite face.

Experiment 1 showed that the upper face half is more diagnostic than the lower half for deciding between fearful and angry expressions in an identification task. The upper half alone was sufficient for high identification performance, closely reflecting the whole face condition. Its perception could also be well-described by a logistic function with a steep category boundary, providing converging evidence for categorical perception based on the upper face half alone. When viewing the lower half, responses were significantly closer to chance, were less s-shaped and had a comparably shallower slope.

In experiment 2, it was expected that the to-be-ignored halves of the face would bias performance, and that regions that have proven most diagnostic in experiment 1 should exert a stronger biasing influence over complementary face parts than vice versa.

Accordingly, results indicated that both eye and mouth regions bias judgements on the respective other face half, with strongest influences exerted by the eyes, leading to what might be considered a breakdown of performance for mouth judgements. This effect is noteworthy, as information in the mouth region is equally present in both experiments. Nevertheless, when instructed to evaluate the mouth and ignore the eyes in a composite face, the participants' ratings reflect the expression of the eyes numerically more strongly than they reflect mouth judgements. This means that a cue that is reasonably useful for expression recognition in isolation, will be of almost no use when exposed to the influence of a strongly diagnostic cue.

The influence of the mouth on eye judgements should be considered equally striking, since it illustrates that even a very diagnostic cue that allows for recognition levels almost as high as a whole face, can be subject to significant bias form a substantially weaker cue. This suggests that even when analytical processing of a single feature is sufficient to allow for categorical perception, human observers seem to integrate features into a whole whenever possible.

The dominance of the eye region in the present experiments is well in line with masking studies which point towards its prominent role for both fear and anger recognition [13]. Studies with neurological patients also indicate a specific importance of the eyes in fear detection [22]. Also, when asked to evaluate expressions of emotion, observers most readily fixate the eyes [14], irrespective of the emotion in question.

Regarding identity recognition, there are many studies that demonstrate the importance of the eyes. For example, when a face has to be learned in a training run and later distinguished from distractors differing in one feature, it is easier to recognize identity changes due to differences in the eyes, compared to nose or mouth changes [29]. Masking out the eyes may even represent a legal requirement in some contexts for ensuring anonymity. Although the scientific basis for the effectiveness of this procedure is equivocal at best (cf. [30]), it illustrates the paramount importance we intuitively place on the eye region. Regarding mental states, it is also commonly assumed that they can be assessed best from the eye region, as reflected for example in the 'reading the mind in the eyes' test [31]. Accordingly, one must ask whether the eye advantage reported in the present study can be generalised to other features and other expressions of emotion.

Even though the eyes may be most likely fixated when judging emotions from intact faces [14], this does not necessarily imply that most information is coded in the eye region. This becomes obvious when considering the studies carried out with patient SM, who exhibits major difficulties at recognizing fear from faces, due to a failure to fixate the eyes [22]. Nevertheless, she is virtually unimpaired at recognizing all other basic emotions. This indicates that it must be possible to recognize those emotions utilising other diagnostic features. Accordingly, masking studies [13] and coding systems for basic emotion expressions [10], suggest that each expression can be best recognized from a set of features specific to that emotion and that focusing on the eyes cannot be the optimal strategy in all cases.

Therefore, regarding the results of the present experiments, we would predict that the basic mechanisms outlined here will be replicated with other facial expressions, but perhaps not for the same features. For example, happy-surprised pairings should be best recognized from the mouth, as both involve mainly muscles of the lower face half [10,13,32].

Interestingly, eye-tracking studies found that increased fixating of the eye region correlates with improved emotion recognition for all expressions [14,33] and argue that fixating the eyes reflects a holistic processing strategy. One could test this assumption using happy-surprise pairings and the composite face task from experiment 2. If holistic processing is associated with fixating the eyes, then eye judgements should be easier in a composite face task, even if a strongly diagnostic distracting lower face half is present.

The present findings should not only be extended to investigate other expressions, but also further elaborated for the currently used fear-anger pairing, to shed light on the basic perceptual mechanisms at work. The employed identification task could be complemented by a discrimination task. There, it should be easier to discriminate two images when they are on opposing sides of the category boundary, compared with being both sampled from within the same category [5,6]. Thus, the sigmoid curves from the present experiments could be used to predict the discrimination performance in a follow-up experiment. As differences in slope between conditions can reflect differences in variance of the underlying noise distribution or the uncertainty of the observer [34,35], it would be important to establish that a between-category advantage [5] is indeed present at the point where the psychometric function is steepest in the identification task.

On the basis of the present data from experiment 1, we would therefore predict that discrimination performance at the category boundary should be higher for eye than mouth judgements.

In the composite face task, the peak of the discrimination function might be shifted to the right and left, respectively, when judging the eyes, in accordance with the shifts of the psychometric curve due to the mouth expressions. Finally the discrimination function should be almost flat when judging the mouth in a composite face, as there is virtually no crossing of a category boundary in the present data.

To further generalise the present findings regarding fear-anger pairings, averaged faces could be used, which have the advantage that idiosyncrasies would average out while expressions prototypical for a certain emotion would be emphasized ([36], p. 76). While studies on categorical perception are often performed with a very limited number of identities (< = 4; cf. [4,7,8,21]), the 20 different faces used in the present experiments should be considered a valuable step towards greater generalisability. However, the fact that the mouth provided so little diagnostic information might be partly due to the fact that 12 out of the 20 face identities expressed the emotions with a closed mouth. While this might have reduced variance in the expressiveness of the lower face half, both the pressing of the lips as well as the baring of the teeth are valid anger expressions [10,12]. Of the two, lip pressing is probably much more frequent in everyday life. Also, when making statements about the importance of either eyes or mouth for recognizing an expression, it should be kept in mind that these features were rather crudely operationalised as the upper and lower face half, respectively. While such a division is standard practice in the field [18,21,37], the muscles around the nose have likely contributed to the judgements of both face halves, and the eyebrows [38] and forehead [11] might have contributed to judgements of the upper face half. While the sclera of the eyes seems crucial for fear detection [39], anger judgements might depend more on the surrounding muscles of the eyes and the eyebrows [13,38], especially since patient SM shows no difficulty at recognizing anger [22].

Another interesting question is to what degree the failure to concentrate on one half of a composite face can be voluntarily overridden, especially since previous work has shown that emotion recognition improvements are associated with increased fixation of the eyes [33]. This could be investigated by introducing training blocks before the experiment proper, a forced minimum exposure time to discourage spontaneous responses, biofeedback from an eye-tracking device helping to fixate on a specific face part, and rewards for correct responses.

To summarise, the present studies show that identification of fear and anger in morphed faces relies heavily on the eye region, corroborating previous research [10,13,14,19].

Expanding previous work, the study shows that observers can perform categorical perception even when viewing only a single face part. For the employed fear-anger pairings, the eye region was sufficient to allow for decisions with a high degree of certainty. This demonstrates that categorical perception can emerge based on single features only, adding to our understanding of how categorization works in complex stimuli such as faces.

Even though the eye region was almost as diagnostic as the whole face, it was not immune to biasing influences in a composite face task. When a full face with conflicting expressions was shown, the psychometric curves for eye judgements were shifted in accordance with the expression in the lower half of the face. For the first time, we can therefore show that holistic perception of a full face can be explained based on the diagnostic value of its constituent parts. In addition, given that observers were aware that a holistic processing strategy impaired their performance, this illustrates that humans involuntarily process faces as a gestalt when trying to make categorical decisions about its expression.

## Supporting Information

S1 Dataset Complete raw data of all participants. (ZIP)

S1 Code Data import for experiment 1. (HTML)

S2 Code Data import for experiment 2. (HTML)

S3 Code Main analysis and plotting. (HTML)

S4 Code Fitting of logistic function. (HTML)

S5 Code Cross-validation of data. (HTML)

S6 Code Additional analyses for face identities. (HTML)

S7 Code Complete analysis code from S1–S6 Codes in executable IPython Notebook format. (ZIP)

S8 Code Complete code for experiment presentation in Neurobs Presentation format. (ZIP)

## References

[1] Keltner D, Haidt J. Social Functions of Emotions at Four Levels of Analysis. Cogn Emot. 1999;13: 505–521. 10.1080/026999399379168

[2] Ekman P. Are there basic emotions? Psychol Rev. 1992;99: 550–553. 1344638 10.1037/0033-295x.99.3.550

[3] Du S, Tao Y, Martinez AM. Compound facial expressions of emotion. Proc Natl Acad Sci. 2014;111: E1454–E1462. 10.1073/pnas.1322355111 24706770 PMC3992629

[4] Young AW, Rowland D, Calder AJ, Etcoff NL, Seth A, Perrett DI. Facial expression megamix: Tests of dimensional and category accounts of emotion recognition. Cognition. 1997;63: 271–313. 10.1016/S0010-0277(97)00003-6 9265872

[5] Fugate JMB. Categorical Perception for Emotional Faces. Emot Rev. 2013;5: 84–89. 10.1177/1754073912451350 25525458 PMC4267261

[6] Harnad SR. Categorical perception: the groundwork of cognition. Cambridge, NY: Cambridge University Press; 1990.

[7] Calder AJ, Young AW, Perrett DI, Etcoff NL, Rowland D. Categorical Perception of Morphed Facial Expressions. Vis Cogn. 1996;3: 81–118. 10.1080/713756735

[8] De Gelder B, Teunisse J-P, Benson PJ. Categorical Perception of Facial Expressions: Categories and their Internal Structure. Cogn Emot. 1997;11: 1–23. 10.1080/026999397380005

[9] Etcoff NL, Magee JJ. Categorical perception of facial expressions. Cognition. 1992;44: 227–240. 10.1016/0010-0277(92)90002-Y 1424493

[10] Ekman P, Friesen WV, Hager JC. Facial action coding system. Salt Lake City, UT: Network Information Research Corporation;. 2002.

[11] Ekman P, Friesen WV. Unmasking the face: a guide to recognizing emotions from facial clues. Cambridge, MA: Malor Books; 1975.

[12] Matsumoto D, Ekman P. Facial expression analysis. Scholarpedia. 2008;3: 4237 10.4249/scholarpedia.4237

[13] Smith ML, Cottrell GW, Gosselin F, Schyns PG. Transmitting and Decoding Facial Expressions. Psychol Sci. 2005;16: 184–189. 10.1111/j.0956-7976.2005.00801.x 15733197

[14] Guo K. Holistic Gaze Strategy to Categorize Facial Expression of Varying Intensities Verdejo García A, editor. PLoS ONE. 2012;7: e42585 10.1371/journal.pone.0042585 22880043 PMC3411802

[15] Rossion B. Picture-plane inversion leads to qualitative changes of face perception. Acta Psychol (Amst). 2008;128: 274–289. 10.1016/j.actpsy.2008.02.003 18396260

[16] Sergent J. An investigation into component and configural processes underlying face perception. Br J Psychol. 1984;75: 221–242. 10.1111/j.2044-8295.1984.tb01895.x 6733396

[17] McKone E, Yovel G. A single holistic representation of spacing and feature shape in faces. J Vis. 2010;8: 163–163. 10.1167/8.6.163

[18] Young AW, Hellawell D, Hay DC. Configurational information in face perception. Perception. 1987;16: 747–759. 10.1068/p160747 3454432

[19] Tanaka JW, Kaiser MD, Butler S, Le Grand R. Mixed emotions: Holistic and analytic perception of facial expressions. Cogn Emot. 2012;26: 961–977. 10.1080/02699931.2011.630933 22273429

[20] Calder AJ, Jansen J. Configural coding of facial expressions: The impact of inversion and photographic negative. Vis Cogn. 2005;12: 495–518. 10.1080/13506280444000418

[21] Chen M-Y, Chen C-C. The contribution of the upper and lower face in happy and sad facial expression classification. Vision Res. 2010;50: 1814–1823. 10.1016/j.visres.2010.06.002 20542054

[22] Adolphs R, Gosselin F, Buchanan TW, Tranel D, Schyns P, Damasio AR. A mechanism for impaired fear recognition after amygdala damage. Nature. 2005;433: 68–72. 10.1038/nature03086 15635411

[23] Lundqvist D, Flykt A, Öhman A. The Karolinska directed emotional faces (KDEF). CD ROM Dep Clin Neurosci Psychol Sect Karolinska Institutet. 1998; 91–630.

[24] Tottenham N, Tanaka JW, Leon AC, McCarry T, Nurse M, Hare TA, et al The NimStim set of facial expressions: Judgments from untrained research participants. Psychiatry Res. 2009;168: 242–249. 10.1016/j.psychres.2008.05.006 19564050 PMC3474329

[25] Kingdom FAA, Prins N. Psychophysics: a practical introduction. London: Academic; 2010.

[26] Duda RO, Hart PE, Stork DG. Pattern classification and scene analysis. New York: Wiley; 1973.

[27] Foley JM, Legge GE. Contrast detection and near-threshold discrimination in human vision. Vision Res. 1981;21: 1041–1053. 10.1016/0042-6989(81)90009-2 7314485

[28] Hilborn R. The ecological detective: confronting models with data. Princeton, NJ: Princeton University Press; 1997.

[29] Tanaka JW, Farah MJ. Parts and wholes in face recognition. Q J Exp Psychol Sect A. 1993;46: 225–245. 10.1080/14640749308401045 8316637

[30] Newton EM, Sweeney L, Malin B. Preserving privacy by de-identifying face images. IEEE Trans Knowl Data Eng. 2005;17: 232–243. 10.1109/TKDE.2005.32

[31] Baron-Cohen S, Wheelwright S, Hill J, Raste Y, Plumb I. The “Reading the Mind in the Eyes” Test Revised Version: A Study with Normal Adults, and Adults with Asperger Syndrome or High-functioning Autism. J Child Psychol Psychiatry. 2001;42: 241–251. 11280420

[32] Leppänen JM, Hietanen JK. Is there more in a happy face than just a big smile? Vis Cogn. 2007;15: 468–490. 10.1080/13506280600765333

[33] Pollux PMJ, Hall S, Guo K. Facial Expression Training Optimises Viewing Strategy in Children and Adults Barton JJS, editor. PLoS ONE. 2014;9: e105418 10.1371/journal.pone.0105418 25144680 PMC4140761

[34] Ashby FG, Townsend JT. Varieties of perceptual independence. Psychol Rev. 1986;93: 154–179. 3714926

[35] Pelli DG. Uncertainty explains many aspects of visual contrast detection and discrimination. J Opt Soc Am A. 1985;2: 1508–1532. 4045584 10.1364/josaa.2.001508

[36] Bruce V, Young AW. Face perception. London; New York: Psychology Press; 2012.

[37] Grand RL, Mondloch CJ, Maurer D, Brent HP. Impairment in Holistic Face Processing Following Early Visual Deprivation. Psychol Sci. 2004;15: 762–768. 10.1111/j.0956-7976.2004.00753.x 15482448

[38] Tipples J, Atkinson AP, Young AW. The eyebrow frown: A salient social signal. Emotion. 2002;2: 288–296. 12899361 10.1037/1528-3542.2.3.288

[39] Whalen PJ. Human Amygdala Responsivity to Masked Fearful Eye Whites. Science. 2004;306: 2061–2061. 10.1126/science.1103617 15604401

