# Supplementary material for: Categorical Perception of Fear and Anger Expressions in Whole, Masked and Composite Faces
Source: PLoS One. 2015 Aug 11;10(8):e0134790. doi: 10.1371/journal.pone.0134790 (PMC4532458; doi:10.1371/journal.pone.0134790)
Supplement: S1 Code — (HTML) [file pone.0134790.s002.html]

SupplementS1\_Experiment1\_Data\_Import


# Supplement S1 of

## Categorical Perception of Fear and Anger Expressions in Whole, Masked and Composite Faces.

# Get Data for Experiment 1 (Masking)¶

This file contains the full code to extract all information from the original logfiles, count the responses and create a table with the main results, which can be used in later analyses.

## Import Libraries¶

In [1]:

```
import fnmatch
import os
import sys
import csv

import numpy as np

import pandas as pd
pd.set_option('max_columns', 100)
pd.set_option('max_rows', 1000)

%pylab inline
```

```
Populating the interactive namespace from numpy and matplotlib
```

## Get List of Subject Files¶

In [2]:

```
def getsubject(whichfolder, whichexperiment):
    os.chdir(whichfolder)
    pList = []
    for file in os.listdir(whichfolder):
        if fnmatch.fnmatch(file, whichexperiment):
            pList.append(file)
    return pList
```

In [3]:

```
pList = getsubject("../Exp1/", "*.log")
pList.sort()
```

Example:

In [5]:

```
pList[:10]
```

Out[5]:

```
['01-Vp001_ParaMasked_pt1.log',
 '01-Vp001_ParaMasked_pt2.log',
 '02-Vp002_ParaMasked_pt1.log',
 '02-Vp002_ParaMasked_pt2.log',
 '03-Vp003_ParaMasked_pt1.log',
 '03-Vp003_ParaMasked_pt2.log',
 '04-Vp004_ParaMasked_pt1.log',
 '04-Vp004_ParaMasked_pt2.log',
 '05-Vp005_ParaMasked_pt1.log',
 '05-Vp005_ParaMasked_pt2.log']
```

## Compute Number of hits¶

In [6]:

```
def howmany(d):
    i = 0.
    for e in d:
        if d[e] == "hit":
            i = i + 1
    return i/len(d)
```

## Find Entries and count # of hits for each condition¶

In the logfiles, the masking conditions have a legacy naming, which goes as follows:

- whole face: 'orig'
- upper half: 'nom' (no mouth)
- lower half: 'noe' (no eyes)

In [9]:

```
def findentry_sub(cond,grade,filename):
    tempDict = {}
    fileContent = open(filename,'r')
    origArray = ["*_00_orig*", "*_01_orig*", "*_02_orig*", "*_03_orig*", "*_04_orig*", "*_05_orig*", "*_06_orig*", "*_07_orig*", "*_08_orig*", "*_09_orig*", "*_10_orig*"]
    noeArray = ["*_00_noe*", "*_01_noe*", "*_02_noe*", "*_03_noe*", "*_04_noe*", "*_05_noe*", "*_06_noe*", "*_07_noe*", "*_08_noe*", "*_09_noe*", "*_10_noe*"]
    nomArray = ["*_00_nom*", "*_01_nom*", "*_02_nom*", "*_03_nom*", "*_04_nom*", "*_05_nom*", "*_06_nom*", "*_07_nom*", "*_08_nom*", "*_09_nom*", "*_10_nom*"]
    condArray = [origArray, noeArray, nomArray]

    for entry in fileContent:
            if fnmatch.fnmatch(entry, condArray[cond][grade]) == True:
                thisEntry = entry.split()
                picName = thisEntry[3][:-4]
                response = thisEntry[-2]
                tempDict[picName] = response

    return howmany(tempDict)
```

Example (for one condition, one morph and one logfile)

In [11]:

```
findentry_sub(0,0,pList[0])
```

Out[11]:

```
0.1
```

## For all morphing grades in one condition¶

In [12]:

```
def get_cond(cond,filename):
    d = {}
    for i in np.arange(0,11):
        if i < 10:
            d["m0"+str(i)] = findentry_sub(cond,i,filename)
        else:
            d["m"+str(i)] = findentry_sub(cond,i,filename)
    return d
```

Example:

In [13]:

```
get_cond(0,pList[0])
```

Out[13]:

```
{'m00': 0.1,
 'm01': 0.15,
 'm02': 0.25,
 'm03': 0.25,
 'm04': 0.35,
 'm05': 0.6,
 'm06': 0.65,
 'm07': 0.9,
 'm08': 1.0,
 'm09': 1.0,
 'm10': 1.0}
```

## For all Conditions in one experiment part¶

In [14]:

```
def get_pt(filename):
    d = {}
    for i in np.arange(0,3):
        d[i] = get_cond(i,filename)
    return d
```

Example:

In [15]:

```
get_pt(pList[0])
```

Out[15]:

```
{0: {'m00': 0.1,
  'm01': 0.15,
  'm02': 0.25,
  'm03': 0.25,
  'm04': 0.35,
  'm05': 0.6,
  'm06': 0.65,
  'm07': 0.9,
  'm08': 1.0,
  'm09': 1.0,
  'm10': 1.0},
 1: {'m00': 0.2,
  'm01': 0.4,
  'm02': 0.35,
  'm03': 0.4,
  'm04': 0.45,
  'm05': 0.5,
  'm06': 0.8,
  'm07': 0.75,
  'm08': 0.85,
  'm09': 0.7,
  'm10': 0.75},
 2: {'m00': 0.15,
  'm01': 0.0,
  'm02': 0.2,
  'm03': 0.2,
  'm04': 0.4,
  'm05': 0.3,
  'm06': 0.5,
  'm07': 0.85,
  'm08': 0.85,
  'm09': 1.0,
  'm10': 0.95}}
```

In [16]:

```
def get_ident_parts(pList,p):
    pt1 = get_pt(pList[p])
    pt2 = get_pt(pList[p+1])
    return pt1,pt2
```

In [18]:

```
def make_ident_df(pList,p):
    # each subject has two logfiles which follow each other
    # in the file list
    pt1_df = pd.DataFrame(get_ident_parts(pList,p)[0])
    pt2_df = pd.DataFrame(get_ident_parts(pList,p)[1])
    cond_names = ["whole","mouth","eyes"]
    pt1_df.columns = cond_names
    pt2_df.columns = cond_names
    avg_df = (pt1_df + pt2_df)/2
    return pt1_df, pt2_df, avg_df
```

Example:

In [20]:

```
make_ident_df(pList,0)[2]
```

Out[20]:

|  | whole | mouth | eyes |
| --- | --- | --- | --- |
| m00 | 0.125 | 0.325 | 0.125 |
| m01 | 0.175 | 0.425 | 0.050 |
| m02 | 0.250 | 0.400 | 0.175 |
| m03 | 0.200 | 0.525 | 0.225 |
| m04 | 0.275 | 0.575 | 0.425 |
| m05 | 0.600 | 0.600 | 0.425 |
| m06 | 0.675 | 0.750 | 0.650 |
| m07 | 0.950 | 0.800 | 0.850 |
| m08 | 0.975 | 0.850 | 0.900 |
| m09 | 1.000 | 0.825 | 0.975 |
| m10 | 1.000 | 0.825 | 0.975 |

## Get Data for entire sample¶

In [21]:

```
def make_ident_sample(pList):
    # make the first subject to establish the df-structure
    pt1_df, pt2_df, avg_df = make_ident_df(pList,0)
    pt1_df.index = [["p001"]*len(pt1_df), pt1_df.index ]
    pt2_df.index = [["p001"]*len(pt2_df), pt2_df.index ]
    avg_df.index = [["p001"]*len(avg_df), avg_df.index ]
    
    # make the rest of the sample
    for p_nr in range(2,len(pList),2):
        p_pt1,p_pt2,p_avg = make_ident_df(pList,p_nr)
        # get the naming of the subject right
        thisNr = "00"+str(p_nr/2+1)
        p_pt1.index = [['p'+thisNr[-3:] ]*len(p_pt1), p_pt1.index ]
        p_pt2.index = [['p'+thisNr[-3:] ]*len(p_pt2), p_pt2.index ]
        p_avg.index = [['p'+thisNr[-3:] ]*len(p_avg), p_avg.index ]

        # add this subject to sample-df
        pt1_df = pd.concat([pt1_df,p_pt1])
        pt2_df = pd.concat([pt2_df,p_pt2])
        avg_df = pd.concat([avg_df,p_avg])

    # finalising the df
    pt1_df.index.names = ['p','grade']
    pt2_df.index.names = ['p','grade']
    avg_df.index.names = ['p','grade']
    
    cond_names = ["whole","mouth","eyes"]
    pt1_df.columns = cond_names
    pt2_df.columns = cond_names
    avg_df.columns = cond_names
    
    return pt1_df, pt2_df, avg_df
```

In [22]:

```
pt1_df, pt2_df, avg_df = make_ident_sample(pList)
avg_df
```

Out[22]:

|  |  | whole | mouth | eyes |
| --- | --- | --- | --- | --- |
| p | grade |  |  |  |
| p001 | m00 | 0.125 | 0.325 | 0.125 |
|  | m01 | 0.175 | 0.425 | 0.050 |
|  | m02 | 0.250 | 0.400 | 0.175 |
|  | m03 | 0.200 | 0.525 | 0.225 |
|  | m04 | 0.275 | 0.575 | 0.425 |
|  | m05 | 0.600 | 0.600 | 0.425 |
|  | m06 | 0.675 | 0.750 | 0.650 |
|  | m07 | 0.950 | 0.800 | 0.850 |
|  | m08 | 0.975 | 0.850 | 0.900 |
|  | m09 | 1.000 | 0.825 | 0.975 |
|  | m10 | 1.000 | 0.825 | 0.975 |
| p002 | m00 | 0.150 | 0.175 | 0.225 |
|  | m01 | 0.000 | 0.200 | 0.250 |
|  | m02 | 0.050 | 0.300 | 0.275 |
|  | m03 | 0.150 | 0.325 | 0.350 |
|  | m04 | 0.225 | 0.400 | 0.450 |
|  | m05 | 0.375 | 0.550 | 0.600 |
|  | m06 | 0.575 | 0.750 | 0.725 |
|  | m07 | 0.700 | 0.750 | 0.825 |
|  | m08 | 0.875 | 0.850 | 0.900 |
|  | m09 | 0.900 | 0.825 | 0.925 |
|  | m10 | 0.975 | 0.900 | 0.900 |
| p003 | m00 | 0.125 | 0.250 | 0.300 |
|  | m01 | 0.150 | 0.250 | 0.375 |
|  | m02 | 0.150 | 0.375 | 0.450 |
|  | m03 | 0.200 | 0.425 | 0.625 |
|  | m04 | 0.475 | 0.600 | 0.700 |
|  | m05 | 0.675 | 0.525 | 0.750 |
|  | m06 | 0.775 | 0.725 | 0.875 |
|  | m07 | 0.975 | 0.650 | 0.850 |
|  | m08 | 1.000 | 0.650 | 0.850 |
|  | m09 | 1.000 | 0.775 | 0.975 |
|  | m10 | 1.000 | 0.850 | 0.975 |
| p004 | m00 | 0.050 | 0.200 | 0.225 |
|  | m01 | 0.075 | 0.300 | 0.275 |
|  | m02 | 0.200 | 0.350 | 0.375 |
|  | m03 | 0.125 | 0.225 | 0.275 |
|  | m04 | 0.325 | 0.400 | 0.375 |
|  | m05 | 0.325 | 0.450 | 0.575 |
|  | m06 | 0.600 | 0.450 | 0.675 |
|  | m07 | 0.800 | 0.525 | 0.575 |
|  | m08 | 0.875 | 0.650 | 0.775 |
|  | m09 | 0.850 | 0.575 | 0.800 |
|  | m10 | 0.875 | 0.575 | 0.875 |
| p005 | m00 | 0.000 | 0.200 | 0.050 |
|  | m01 | 0.000 | 0.200 | 0.025 |
|  | m02 | 0.100 | 0.300 | 0.150 |
|  | m03 | 0.150 | 0.275 | 0.275 |
|  | m04 | 0.200 | 0.350 | 0.250 |
|  | m05 | 0.225 | 0.275 | 0.350 |
|  | m06 | 0.450 | 0.450 | 0.525 |
|  | m07 | 0.600 | 0.400 | 0.600 |
|  | m08 | 0.825 | 0.575 | 0.825 |
|  | m09 | 0.775 | 0.650 | 0.875 |
|  | m10 | 0.850 | 0.550 | 0.875 |
| p006 | m00 | 0.050 | 0.175 | 0.050 |
|  | m01 | 0.075 | 0.175 | 0.075 |
|  | m02 | 0.025 | 0.225 | 0.075 |
|  | m03 | 0.075 | 0.350 | 0.050 |
|  | m04 | 0.175 | 0.375 | 0.350 |
|  | m05 | 0.450 | 0.475 | 0.400 |
|  | m06 | 0.775 | 0.625 | 0.700 |
|  | m07 | 0.950 | 0.675 | 0.825 |
|  | m08 | 0.950 | 0.750 | 0.975 |
|  | m09 | 1.000 | 0.850 | 0.975 |
|  | m10 | 1.000 | 0.875 | 1.000 |
| p007 | m00 | 0.125 | 0.375 | 0.100 |
|  | m01 | 0.100 | 0.550 | 0.100 |
|  | m02 | 0.150 | 0.575 | 0.200 |
|  | m03 | 0.225 | 0.450 | 0.225 |
|  | m04 | 0.150 | 0.550 | 0.250 |
|  | m05 | 0.325 | 0.650 | 0.450 |
|  | m06 | 0.600 | 0.725 | 0.600 |
|  | m07 | 0.800 | 0.925 | 0.700 |
|  | m08 | 0.900 | 0.950 | 0.850 |
|  | m09 | 0.950 | 0.875 | 0.900 |
|  | m10 | 0.975 | 0.875 | 0.950 |
| p008 | m00 | 0.175 | 0.600 | 0.150 |
|  | m01 | 0.275 | 0.675 | 0.150 |
|  | m02 | 0.250 | 0.650 | 0.300 |
|  | m03 | 0.300 | 0.625 | 0.325 |
|  | m04 | 0.425 | 0.675 | 0.300 |
|  | m05 | 0.600 | 0.725 | 0.425 |
|  | m06 | 0.625 | 0.800 | 0.650 |
|  | m07 | 0.900 | 0.900 | 0.800 |
|  | m08 | 0.950 | 0.950 | 0.975 |
|  | m09 | 0.950 | 0.925 | 0.925 |
|  | m10 | 1.000 | 0.925 | 0.975 |
| p009 | m00 | 0.050 | 0.100 | 0.025 |
|  | m01 | 0.025 | 0.075 | 0.025 |
|  | m02 | 0.000 | 0.200 | 0.050 |
|  | m03 | 0.050 | 0.200 | 0.150 |
|  | m04 | 0.250 | 0.275 | 0.225 |
|  | m05 | 0.350 | 0.625 | 0.450 |
|  | m06 | 0.625 | 0.650 | 0.550 |
|  | m07 | 0.800 | 0.800 | 0.875 |
|  | m08 | 0.950 | 0.850 | 0.850 |
|  | m09 | 0.975 | 0.850 | 0.950 |
|  | m10 | 1.000 | 0.875 | 0.975 |
| p010 | m00 | 0.150 | 0.350 | 0.175 |
|  | m01 | 0.225 | 0.375 | 0.350 |
|  | m02 | 0.300 | 0.375 | 0.375 |
|  | m03 | 0.275 | 0.500 | 0.375 |
|  | m04 | 0.250 | 0.550 | 0.400 |
|  | m05 | 0.600 | 0.600 | 0.400 |
|  | m06 | 0.700 | 0.700 | 0.525 |
|  | m07 | 0.850 | 0.700 | 0.700 |
|  | m08 | 0.975 | 0.775 | 0.875 |
|  | m09 | 0.950 | 0.700 | 0.825 |
|  | m10 | 1.000 | 0.850 | 0.850 |
| p011 | m00 | 0.025 | 0.325 | 0.050 |
|  | m01 | 0.100 | 0.250 | 0.050 |
|  | m02 | 0.100 | 0.425 | 0.075 |
|  | m03 | 0.075 | 0.450 | 0.100 |
|  | m04 | 0.200 | 0.575 | 0.275 |
|  | m05 | 0.400 | 0.675 | 0.250 |
|  | m06 | 0.775 | 0.825 | 0.450 |
|  | m07 | 0.900 | 0.925 | 0.800 |
|  | m08 | 0.925 | 1.000 | 0.900 |
|  | m09 | 0.950 | 0.900 | 1.000 |
|  | m10 | 1.000 | 0.950 | 0.975 |
| p012 | m00 | 0.125 | 0.375 | 0.250 |
|  | m01 | 0.075 | 0.450 | 0.275 |
|  | m02 | 0.250 | 0.450 | 0.325 |
|  | m03 | 0.375 | 0.450 | 0.250 |
|  | m04 | 0.475 | 0.575 | 0.450 |
|  | m05 | 0.675 | 0.625 | 0.550 |
|  | m06 | 0.850 | 0.850 | 0.625 |
|  | m07 | 1.000 | 0.850 | 0.700 |
|  | m08 | 0.950 | 0.925 | 0.875 |
|  | m09 | 1.000 | 0.950 | 0.900 |
|  | m10 | 0.950 | 0.875 | 0.950 |
| p013 | m00 | 0.025 | 0.375 | 0.000 |
|  | m01 | 0.025 | 0.325 | 0.125 |
|  | m02 | 0.025 | 0.450 | 0.150 |
|  | m03 | 0.125 | 0.450 | 0.175 |
|  | m04 | 0.225 | 0.600 | 0.250 |
|  | m05 | 0.275 | 0.650 | 0.350 |
|  | m06 | 0.625 | 0.700 | 0.575 |
|  | m07 | 0.725 | 0.775 | 0.775 |
|  | m08 | 0.900 | 0.925 | 0.925 |
|  | m09 | 0.925 | 0.800 | 0.875 |
|  | m10 | 0.950 | 0.800 | 0.975 |
| p014 | m00 | 0.050 | 0.100 | 0.050 |
|  | m01 | 0.025 | 0.025 | 0.075 |
|  | m02 | 0.100 | 0.300 | 0.100 |
|  | m03 | 0.100 | 0.125 | 0.250 |
|  | m04 | 0.225 | 0.225 | 0.300 |
|  | m05 | 0.500 | 0.250 | 0.500 |
|  | m06 | 0.625 | 0.575 | 0.625 |
|  | m07 | 0.825 | 0.650 | 0.800 |
|  | m08 | 0.975 | 0.750 | 0.950 |
|  | m09 | 0.975 | 0.750 | 0.975 |
|  | m10 | 1.000 | 0.800 | 0.950 |
| p015 | m00 | 0.075 | 0.275 | 0.125 |
|  | m01 | 0.050 | 0.225 | 0.075 |
|  | m02 | 0.100 | 0.300 | 0.075 |
|  | m03 | 0.175 | 0.350 | 0.350 |
|  | m04 | 0.350 | 0.375 | 0.225 |
|  | m05 | 0.375 | 0.425 | 0.425 |
|  | m06 | 0.675 | 0.500 | 0.525 |
|  | m07 | 0.825 | 0.550 | 0.650 |
|  | m08 | 0.950 | 0.550 | 0.750 |
|  | m09 | 0.975 | 0.550 | 0.900 |
|  | m10 | 1.000 | 0.625 | 0.900 |
| p016 | m00 | 0.100 | 0.375 | 0.050 |
|  | m01 | 0.050 | 0.450 | 0.150 |
|  | m02 | 0.100 | 0.500 | 0.175 |
|  | m03 | 0.175 | 0.525 | 0.175 |
|  | m04 | 0.275 | 0.625 | 0.225 |
|  | m05 | 0.475 | 0.800 | 0.425 |
|  | m06 | 0.625 | 0.800 | 0.625 |
|  | m07 | 0.800 | 0.850 | 0.825 |
|  | m08 | 0.900 | 0.975 | 0.950 |
|  | m09 | 1.000 | 0.950 | 0.950 |
|  | m10 | 1.000 | 0.975 | 0.975 |
| p017 | m00 | 0.575 | 0.525 | 0.625 |
|  | m01 | 0.400 | 0.550 | 0.625 |
|  | m02 | 0.600 | 0.625 | 0.675 |
|  | m03 | 0.550 | 0.650 | 0.650 |
|  | m04 | 0.625 | 0.650 | 0.550 |
|  | m05 | 0.650 | 0.700 | 0.600 |
|  | m06 | 0.700 | 0.675 | 0.675 |
|  | m07 | 0.775 | 0.625 | 0.625 |
|  | m08 | 0.825 | 0.700 | 0.825 |
|  | m09 | 0.825 | 0.750 | 0.875 |
|  | m10 | 0.825 | 0.650 | 0.850 |
| p018 | m00 | 0.450 | 0.550 | 0.350 |
|  | m01 | 0.425 | 0.550 | 0.450 |
|  | m02 | 0.425 | 0.500 | 0.550 |
|  | m03 | 0.450 | 0.525 | 0.375 |
|  | m04 | 0.550 | 0.600 | 0.650 |
|  | m05 | 0.575 | 0.450 | 0.550 |
|  | m06 | 0.625 | 0.625 | 0.650 |
|  | m07 | 0.525 | 0.575 | 0.725 |
|  | m08 | 0.875 | 0.500 | 0.725 |
|  | m09 | 0.900 | 0.650 | 0.800 |
|  | m10 | 0.800 | 0.500 | 0.950 |
| p019 | m00 | 0.050 | 0.350 | 0.075 |
|  | m01 | 0.025 | 0.300 | 0.050 |
|  | m02 | 0.100 | 0.375 | 0.150 |
|  | m03 | 0.075 | 0.400 | 0.225 |
|  | m04 | 0.225 | 0.475 | 0.300 |
|  | m05 | 0.525 | 0.475 | 0.475 |
|  | m06 | 0.775 | 0.725 | 0.725 |
|  | m07 | 0.950 | 0.850 | 0.900 |
|  | m08 | 0.975 | 0.825 | 0.950 |
|  | m09 | 1.000 | 0.825 | 0.975 |
|  | m10 | 1.000 | 0.925 | 1.000 |
| p020 | m00 | 0.275 | 0.200 | 0.275 |
|  | m01 | 0.275 | 0.400 | 0.350 |
|  | m02 | 0.325 | 0.200 | 0.425 |
|  | m03 | 0.350 | 0.400 | 0.450 |
|  | m04 | 0.400 | 0.400 | 0.525 |
|  | m05 | 0.600 | 0.375 | 0.650 |
|  | m06 | 0.725 | 0.400 | 0.675 |
|  | m07 | 0.750 | 0.375 | 0.775 |
|  | m08 | 0.875 | 0.400 | 0.900 |
|  | m09 | 0.900 | 0.475 | 0.850 |
|  | m10 | 0.875 | 0.400 | 0.875 |
| p021 | m00 | 0.025 | 0.175 | 0.025 |
|  | m01 | 0.025 | 0.275 | 0.050 |
|  | m02 | 0.050 | 0.275 | 0.050 |
|  | m03 | 0.100 | 0.300 | 0.050 |
|  | m04 | 0.175 | 0.425 | 0.075 |
|  | m05 | 0.325 | 0.525 | 0.200 |
|  | m06 | 0.450 | 0.525 | 0.250 |
|  | m07 | 0.700 | 0.550 | 0.450 |
|  | m08 | 0.850 | 0.650 | 0.600 |
|  | m09 | 0.950 | 0.825 | 0.875 |
|  | m10 | 1.000 | 0.750 | 0.875 |
| p022 | m00 | 0.050 | 0.225 | 0.050 |
|  | m01 | 0.050 | 0.200 | 0.075 |
|  | m02 | 0.100 | 0.225 | 0.125 |
|  | m03 | 0.175 | 0.300 | 0.150 |
|  | m04 | 0.325 | 0.400 | 0.350 |
|  | m05 | 0.475 | 0.575 | 0.450 |
|  | m06 | 0.600 | 0.725 | 0.575 |
|  | m07 | 0.775 | 0.750 | 0.775 |
|  | m08 | 0.950 | 0.800 | 0.875 |
|  | m09 | 1.000 | 0.900 | 1.000 |
|  | m10 | 1.000 | 0.900 | 0.950 |
| p023 | m00 | 0.075 | 0.200 | 0.150 |
|  | m01 | 0.025 | 0.200 | 0.175 |
|  | m02 | 0.125 | 0.150 | 0.075 |
|  | m03 | 0.200 | 0.250 | 0.250 |
|  | m04 | 0.325 | 0.375 | 0.325 |
|  | m05 | 0.525 | 0.425 | 0.500 |
|  | m06 | 0.750 | 0.550 | 0.675 |
|  | m07 | 0.900 | 0.675 | 0.850 |
|  | m08 | 0.975 | 0.700 | 0.925 |
|  | m09 | 1.000 | 0.750 | 0.950 |
|  | m10 | 1.000 | 0.800 | 1.000 |
| p024 | m00 | 0.100 | 0.250 | 0.125 |
|  | m01 | 0.175 | 0.300 | 0.100 |
|  | m02 | 0.150 | 0.275 | 0.200 |
|  | m03 | 0.200 | 0.375 | 0.300 |
|  | m04 | 0.275 | 0.300 | 0.350 |
|  | m05 | 0.550 | 0.400 | 0.450 |
|  | m06 | 0.800 | 0.550 | 0.725 |
|  | m07 | 0.925 | 0.675 | 0.825 |
|  | m08 | 0.975 | 0.725 | 0.950 |
|  | m09 | 0.975 | 0.725 | 0.975 |
|  | m10 | 1.000 | 0.800 | 0.975 |
| p025 | m00 | 0.200 | 0.450 | 0.175 |
|  | m01 | 0.175 | 0.425 | 0.225 |
|  | m02 | 0.275 | 0.375 | 0.225 |
|  | m03 | 0.225 | 0.425 | 0.325 |
|  | m04 | 0.425 | 0.500 | 0.325 |
|  | m05 | 0.500 | 0.475 | 0.425 |
|  | m06 | 0.600 | 0.450 | 0.400 |
|  | m07 | 0.700 | 0.575 | 0.725 |
|  | m08 | 0.750 | 0.550 | 0.900 |
|  | m09 | 0.900 | 0.600 | 0.900 |
|  | m10 | 0.950 | 0.700 | 0.850 |
| p026 | m00 | 0.075 | 0.200 | 0.150 |
|  | m01 | 0.075 | 0.300 | 0.250 |
|  | m02 | 0.050 | 0.175 | 0.225 |
|  | m03 | 0.075 | 0.300 | 0.300 |
|  | m04 | 0.225 | 0.350 | 0.400 |
|  | m05 | 0.475 | 0.400 | 0.525 |
|  | m06 | 0.700 | 0.525 | 0.675 |
|  | m07 | 0.875 | 0.500 | 0.800 |
|  | m08 | 0.975 | 0.675 | 0.875 |
|  | m09 | 1.000 | 0.700 | 0.875 |
|  | m10 | 1.000 | 0.725 | 0.975 |
| p027 | m00 | 0.075 | 0.200 | 0.175 |
|  | m01 | 0.175 | 0.350 | 0.275 |
|  | m02 | 0.200 | 0.375 | 0.300 |
|  | m03 | 0.300 | 0.525 | 0.500 |
|  | m04 | 0.325 | 0.600 | 0.550 |
|  | m05 | 0.500 | 0.700 | 0.625 |
|  | m06 | 0.750 | 0.850 | 0.700 |
|  | m07 | 0.925 | 0.850 | 0.875 |
|  | m08 | 0.925 | 0.825 | 0.875 |
|  | m09 | 1.000 | 0.900 | 0.975 |
|  | m10 | 1.000 | 0.850 | 1.000 |
| p028 | m00 | 0.075 | 0.325 | 0.025 |
|  | m01 | 0.025 | 0.250 | 0.100 |
|  | m02 | 0.025 | 0.375 | 0.100 |
|  | m03 | 0.150 | 0.325 | 0.175 |
|  | m04 | 0.250 | 0.425 | 0.225 |
|  | m05 | 0.275 | 0.550 | 0.225 |
|  | m06 | 0.550 | 0.725 | 0.425 |
|  | m07 | 0.700 | 0.675 | 0.675 |
|  | m08 | 0.850 | 0.750 | 0.850 |
|  | m09 | 0.975 | 0.800 | 0.900 |
|  | m10 | 0.975 | 0.850 | 0.900 |
| p029 | m00 | 0.000 | 0.125 | 0.025 |
|  | m01 | 0.050 | 0.150 | 0.050 |
|  | m02 | 0.050 | 0.200 | 0.100 |
|  | m03 | 0.075 | 0.175 | 0.125 |
|  | m04 | 0.225 | 0.250 | 0.175 |
|  | m05 | 0.250 | 0.350 | 0.300 |
|  | m06 | 0.650 | 0.525 | 0.500 |
|  | m07 | 0.675 | 0.575 | 0.725 |
|  | m08 | 0.825 | 0.675 | 0.825 |
|  | m09 | 0.900 | 0.775 | 0.900 |
|  | m10 | 0.950 | 0.700 | 0.875 |
| p030 | m00 | 0.350 | 0.625 | 0.325 |
|  | m01 | 0.375 | 0.550 | 0.450 |
|  | m02 | 0.400 | 0.575 | 0.400 |
|  | m03 | 0.525 | 0.650 | 0.550 |
|  | m04 | 0.450 | 0.700 | 0.600 |
|  | m05 | 0.650 | 0.675 | 0.600 |
|  | m06 | 0.850 | 0.700 | 0.725 |
|  | m07 | 0.850 | 0.825 | 0.725 |
|  | m08 | 1.000 | 0.800 | 0.750 |
|  | m09 | 0.950 | 1.000 | 0.875 |
|  | m10 | 1.000 | 0.850 | 0.875 |

## create all data frames¶

Here we convert the probabilities into percentages

In [23]:

```
avg_between_df = avg_df.unstack("grade")*100
pt1_between_df = pt1_df.unstack("grade")*100
pt2_between_df = pt2_df.unstack("grade")*100
avg_between_df
```

Out[23]:

|  | whole | | | | | | | | | | | mouth | | | | | | | | | | | eyes | | | | | | | | | | |
| --- | --- | --- | --- | --- | --- | --- | --- | --- | --- | --- | --- | --- | --- | --- | --- | --- | --- | --- | --- | --- | --- | --- | --- | --- | --- | --- | --- | --- | --- | --- | --- | --- | --- |
| grade | m00 | m01 | m02 | m03 | m04 | m05 | m06 | m07 | m08 | m09 | m10 | m00 | m01 | m02 | m03 | m04 | m05 | m06 | m07 | m08 | m09 | m10 | m00 | m01 | m02 | m03 | m04 | m05 | m06 | m07 | m08 | m09 | m10 |
| p |  |  |  |  |  |  |  |  |  |  |  |  |  |  |  |  |  |  |  |  |  |  |  |  |  |  |  |  |  |  |  |  |  |
| p001 | 12.5 | 17.5 | 25.0 | 20.0 | 27.5 | 60.0 | 67.5 | 95.0 | 97.5 | 100.0 | 100.0 | 32.5 | 42.5 | 40.0 | 52.5 | 57.5 | 60.0 | 75.0 | 80.0 | 85.0 | 82.5 | 82.5 | 12.5 | 5.0 | 17.5 | 22.5 | 42.5 | 42.5 | 65.0 | 85.0 | 90.0 | 97.5 | 97.5 |
| p002 | 15.0 | 0.0 | 5.0 | 15.0 | 22.5 | 37.5 | 57.5 | 70.0 | 87.5 | 90.0 | 97.5 | 17.5 | 20.0 | 30.0 | 32.5 | 40.0 | 55.0 | 75.0 | 75.0 | 85.0 | 82.5 | 90.0 | 22.5 | 25.0 | 27.5 | 35.0 | 45.0 | 60.0 | 72.5 | 82.5 | 90.0 | 92.5 | 90.0 |
| p003 | 12.5 | 15.0 | 15.0 | 20.0 | 47.5 | 67.5 | 77.5 | 97.5 | 100.0 | 100.0 | 100.0 | 25.0 | 25.0 | 37.5 | 42.5 | 60.0 | 52.5 | 72.5 | 65.0 | 65.0 | 77.5 | 85.0 | 30.0 | 37.5 | 45.0 | 62.5 | 70.0 | 75.0 | 87.5 | 85.0 | 85.0 | 97.5 | 97.5 |
| p004 | 5.0 | 7.5 | 20.0 | 12.5 | 32.5 | 32.5 | 60.0 | 80.0 | 87.5 | 85.0 | 87.5 | 20.0 | 30.0 | 35.0 | 22.5 | 40.0 | 45.0 | 45.0 | 52.5 | 65.0 | 57.5 | 57.5 | 22.5 | 27.5 | 37.5 | 27.5 | 37.5 | 57.5 | 67.5 | 57.5 | 77.5 | 80.0 | 87.5 |
| p005 | 0.0 | 0.0 | 10.0 | 15.0 | 20.0 | 22.5 | 45.0 | 60.0 | 82.5 | 77.5 | 85.0 | 20.0 | 20.0 | 30.0 | 27.5 | 35.0 | 27.5 | 45.0 | 40.0 | 57.5 | 65.0 | 55.0 | 5.0 | 2.5 | 15.0 | 27.5 | 25.0 | 35.0 | 52.5 | 60.0 | 82.5 | 87.5 | 87.5 |
| p006 | 5.0 | 7.5 | 2.5 | 7.5 | 17.5 | 45.0 | 77.5 | 95.0 | 95.0 | 100.0 | 100.0 | 17.5 | 17.5 | 22.5 | 35.0 | 37.5 | 47.5 | 62.5 | 67.5 | 75.0 | 85.0 | 87.5 | 5.0 | 7.5 | 7.5 | 5.0 | 35.0 | 40.0 | 70.0 | 82.5 | 97.5 | 97.5 | 100.0 |
| p007 | 12.5 | 10.0 | 15.0 | 22.5 | 15.0 | 32.5 | 60.0 | 80.0 | 90.0 | 95.0 | 97.5 | 37.5 | 55.0 | 57.5 | 45.0 | 55.0 | 65.0 | 72.5 | 92.5 | 95.0 | 87.5 | 87.5 | 10.0 | 10.0 | 20.0 | 22.5 | 25.0 | 45.0 | 60.0 | 70.0 | 85.0 | 90.0 | 95.0 |
| p008 | 17.5 | 27.5 | 25.0 | 30.0 | 42.5 | 60.0 | 62.5 | 90.0 | 95.0 | 95.0 | 100.0 | 60.0 | 67.5 | 65.0 | 62.5 | 67.5 | 72.5 | 80.0 | 90.0 | 95.0 | 92.5 | 92.5 | 15.0 | 15.0 | 30.0 | 32.5 | 30.0 | 42.5 | 65.0 | 80.0 | 97.5 | 92.5 | 97.5 |
| p009 | 5.0 | 2.5 | 0.0 | 5.0 | 25.0 | 35.0 | 62.5 | 80.0 | 95.0 | 97.5 | 100.0 | 10.0 | 7.5 | 20.0 | 20.0 | 27.5 | 62.5 | 65.0 | 80.0 | 85.0 | 85.0 | 87.5 | 2.5 | 2.5 | 5.0 | 15.0 | 22.5 | 45.0 | 55.0 | 87.5 | 85.0 | 95.0 | 97.5 |
| p010 | 15.0 | 22.5 | 30.0 | 27.5 | 25.0 | 60.0 | 70.0 | 85.0 | 97.5 | 95.0 | 100.0 | 35.0 | 37.5 | 37.5 | 50.0 | 55.0 | 60.0 | 70.0 | 70.0 | 77.5 | 70.0 | 85.0 | 17.5 | 35.0 | 37.5 | 37.5 | 40.0 | 40.0 | 52.5 | 70.0 | 87.5 | 82.5 | 85.0 |
| p011 | 2.5 | 10.0 | 10.0 | 7.5 | 20.0 | 40.0 | 77.5 | 90.0 | 92.5 | 95.0 | 100.0 | 32.5 | 25.0 | 42.5 | 45.0 | 57.5 | 67.5 | 82.5 | 92.5 | 100.0 | 90.0 | 95.0 | 5.0 | 5.0 | 7.5 | 10.0 | 27.5 | 25.0 | 45.0 | 80.0 | 90.0 | 100.0 | 97.5 |
| p012 | 12.5 | 7.5 | 25.0 | 37.5 | 47.5 | 67.5 | 85.0 | 100.0 | 95.0 | 100.0 | 95.0 | 37.5 | 45.0 | 45.0 | 45.0 | 57.5 | 62.5 | 85.0 | 85.0 | 92.5 | 95.0 | 87.5 | 25.0 | 27.5 | 32.5 | 25.0 | 45.0 | 55.0 | 62.5 | 70.0 | 87.5 | 90.0 | 95.0 |
| p013 | 2.5 | 2.5 | 2.5 | 12.5 | 22.5 | 27.5 | 62.5 | 72.5 | 90.0 | 92.5 | 95.0 | 37.5 | 32.5 | 45.0 | 45.0 | 60.0 | 65.0 | 70.0 | 77.5 | 92.5 | 80.0 | 80.0 | 0.0 | 12.5 | 15.0 | 17.5 | 25.0 | 35.0 | 57.5 | 77.5 | 92.5 | 87.5 | 97.5 |
| p014 | 5.0 | 2.5 | 10.0 | 10.0 | 22.5 | 50.0 | 62.5 | 82.5 | 97.5 | 97.5 | 100.0 | 10.0 | 2.5 | 30.0 | 12.5 | 22.5 | 25.0 | 57.5 | 65.0 | 75.0 | 75.0 | 80.0 | 5.0 | 7.5 | 10.0 | 25.0 | 30.0 | 50.0 | 62.5 | 80.0 | 95.0 | 97.5 | 95.0 |
| p015 | 7.5 | 5.0 | 10.0 | 17.5 | 35.0 | 37.5 | 67.5 | 82.5 | 95.0 | 97.5 | 100.0 | 27.5 | 22.5 | 30.0 | 35.0 | 37.5 | 42.5 | 50.0 | 55.0 | 55.0 | 55.0 | 62.5 | 12.5 | 7.5 | 7.5 | 35.0 | 22.5 | 42.5 | 52.5 | 65.0 | 75.0 | 90.0 | 90.0 |
| p016 | 10.0 | 5.0 | 10.0 | 17.5 | 27.5 | 47.5 | 62.5 | 80.0 | 90.0 | 100.0 | 100.0 | 37.5 | 45.0 | 50.0 | 52.5 | 62.5 | 80.0 | 80.0 | 85.0 | 97.5 | 95.0 | 97.5 | 5.0 | 15.0 | 17.5 | 17.5 | 22.5 | 42.5 | 62.5 | 82.5 | 95.0 | 95.0 | 97.5 |
| p017 | 57.5 | 40.0 | 60.0 | 55.0 | 62.5 | 65.0 | 70.0 | 77.5 | 82.5 | 82.5 | 82.5 | 52.5 | 55.0 | 62.5 | 65.0 | 65.0 | 70.0 | 67.5 | 62.5 | 70.0 | 75.0 | 65.0 | 62.5 | 62.5 | 67.5 | 65.0 | 55.0 | 60.0 | 67.5 | 62.5 | 82.5 | 87.5 | 85.0 |
| p018 | 45.0 | 42.5 | 42.5 | 45.0 | 55.0 | 57.5 | 62.5 | 52.5 | 87.5 | 90.0 | 80.0 | 55.0 | 55.0 | 50.0 | 52.5 | 60.0 | 45.0 | 62.5 | 57.5 | 50.0 | 65.0 | 50.0 | 35.0 | 45.0 | 55.0 | 37.5 | 65.0 | 55.0 | 65.0 | 72.5 | 72.5 | 80.0 | 95.0 |
| p019 | 5.0 | 2.5 | 10.0 | 7.5 | 22.5 | 52.5 | 77.5 | 95.0 | 97.5 | 100.0 | 100.0 | 35.0 | 30.0 | 37.5 | 40.0 | 47.5 | 47.5 | 72.5 | 85.0 | 82.5 | 82.5 | 92.5 | 7.5 | 5.0 | 15.0 | 22.5 | 30.0 | 47.5 | 72.5 | 90.0 | 95.0 | 97.5 | 100.0 |
| p020 | 27.5 | 27.5 | 32.5 | 35.0 | 40.0 | 60.0 | 72.5 | 75.0 | 87.5 | 90.0 | 87.5 | 20.0 | 40.0 | 20.0 | 40.0 | 40.0 | 37.5 | 40.0 | 37.5 | 40.0 | 47.5 | 40.0 | 27.5 | 35.0 | 42.5 | 45.0 | 52.5 | 65.0 | 67.5 | 77.5 | 90.0 | 85.0 | 87.5 |
| p021 | 2.5 | 2.5 | 5.0 | 10.0 | 17.5 | 32.5 | 45.0 | 70.0 | 85.0 | 95.0 | 100.0 | 17.5 | 27.5 | 27.5 | 30.0 | 42.5 | 52.5 | 52.5 | 55.0 | 65.0 | 82.5 | 75.0 | 2.5 | 5.0 | 5.0 | 5.0 | 7.5 | 20.0 | 25.0 | 45.0 | 60.0 | 87.5 | 87.5 |
| p022 | 5.0 | 5.0 | 10.0 | 17.5 | 32.5 | 47.5 | 60.0 | 77.5 | 95.0 | 100.0 | 100.0 | 22.5 | 20.0 | 22.5 | 30.0 | 40.0 | 57.5 | 72.5 | 75.0 | 80.0 | 90.0 | 90.0 | 5.0 | 7.5 | 12.5 | 15.0 | 35.0 | 45.0 | 57.5 | 77.5 | 87.5 | 100.0 | 95.0 |
| p023 | 7.5 | 2.5 | 12.5 | 20.0 | 32.5 | 52.5 | 75.0 | 90.0 | 97.5 | 100.0 | 100.0 | 20.0 | 20.0 | 15.0 | 25.0 | 37.5 | 42.5 | 55.0 | 67.5 | 70.0 | 75.0 | 80.0 | 15.0 | 17.5 | 7.5 | 25.0 | 32.5 | 50.0 | 67.5 | 85.0 | 92.5 | 95.0 | 100.0 |
| p024 | 10.0 | 17.5 | 15.0 | 20.0 | 27.5 | 55.0 | 80.0 | 92.5 | 97.5 | 97.5 | 100.0 | 25.0 | 30.0 | 27.5 | 37.5 | 30.0 | 40.0 | 55.0 | 67.5 | 72.5 | 72.5 | 80.0 | 12.5 | 10.0 | 20.0 | 30.0 | 35.0 | 45.0 | 72.5 | 82.5 | 95.0 | 97.5 | 97.5 |
| p025 | 20.0 | 17.5 | 27.5 | 22.5 | 42.5 | 50.0 | 60.0 | 70.0 | 75.0 | 90.0 | 95.0 | 45.0 | 42.5 | 37.5 | 42.5 | 50.0 | 47.5 | 45.0 | 57.5 | 55.0 | 60.0 | 70.0 | 17.5 | 22.5 | 22.5 | 32.5 | 32.5 | 42.5 | 40.0 | 72.5 | 90.0 | 90.0 | 85.0 |
| p026 | 7.5 | 7.5 | 5.0 | 7.5 | 22.5 | 47.5 | 70.0 | 87.5 | 97.5 | 100.0 | 100.0 | 20.0 | 30.0 | 17.5 | 30.0 | 35.0 | 40.0 | 52.5 | 50.0 | 67.5 | 70.0 | 72.5 | 15.0 | 25.0 | 22.5 | 30.0 | 40.0 | 52.5 | 67.5 | 80.0 | 87.5 | 87.5 | 97.5 |
| p027 | 7.5 | 17.5 | 20.0 | 30.0 | 32.5 | 50.0 | 75.0 | 92.5 | 92.5 | 100.0 | 100.0 | 20.0 | 35.0 | 37.5 | 52.5 | 60.0 | 70.0 | 85.0 | 85.0 | 82.5 | 90.0 | 85.0 | 17.5 | 27.5 | 30.0 | 50.0 | 55.0 | 62.5 | 70.0 | 87.5 | 87.5 | 97.5 | 100.0 |
| p028 | 7.5 | 2.5 | 2.5 | 15.0 | 25.0 | 27.5 | 55.0 | 70.0 | 85.0 | 97.5 | 97.5 | 32.5 | 25.0 | 37.5 | 32.5 | 42.5 | 55.0 | 72.5 | 67.5 | 75.0 | 80.0 | 85.0 | 2.5 | 10.0 | 10.0 | 17.5 | 22.5 | 22.5 | 42.5 | 67.5 | 85.0 | 90.0 | 90.0 |
| p029 | 0.0 | 5.0 | 5.0 | 7.5 | 22.5 | 25.0 | 65.0 | 67.5 | 82.5 | 90.0 | 95.0 | 12.5 | 15.0 | 20.0 | 17.5 | 25.0 | 35.0 | 52.5 | 57.5 | 67.5 | 77.5 | 70.0 | 2.5 | 5.0 | 10.0 | 12.5 | 17.5 | 30.0 | 50.0 | 72.5 | 82.5 | 90.0 | 87.5 |
| p030 | 35.0 | 37.5 | 40.0 | 52.5 | 45.0 | 65.0 | 85.0 | 85.0 | 100.0 | 95.0 | 100.0 | 62.5 | 55.0 | 57.5 | 65.0 | 70.0 | 67.5 | 70.0 | 82.5 | 80.0 | 100.0 | 85.0 | 32.5 | 45.0 | 40.0 | 55.0 | 60.0 | 60.0 | 72.5 | 72.5 | 75.0 | 87.5 | 87.5 |

## Exclude Non-Compliant Subjects¶

By comparing accuracies and reaction times (see below), we identified two subjects who were both fast and very inaccurate. These were excluded from all further analyses.

In [24]:

```
rev_avg_between_df = pd.concat([avg_between_df[0:16], avg_between_df[18:] ])
rev_pt1_between_df = pd.concat([pt1_between_df[0:16], pt1_between_df[18:] ])
rev_pt2_between_df = pd.concat([pt2_between_df[0:16], pt2_between_df[18:] ])
rev_avg_between_df
```

Out[24]:

|  | whole | | | | | | | | | | | mouth | | | | | | | | | | | eyes | | | | | | | | | | |
| --- | --- | --- | --- | --- | --- | --- | --- | --- | --- | --- | --- | --- | --- | --- | --- | --- | --- | --- | --- | --- | --- | --- | --- | --- | --- | --- | --- | --- | --- | --- | --- | --- | --- |
| grade | m00 | m01 | m02 | m03 | m04 | m05 | m06 | m07 | m08 | m09 | m10 | m00 | m01 | m02 | m03 | m04 | m05 | m06 | m07 | m08 | m09 | m10 | m00 | m01 | m02 | m03 | m04 | m05 | m06 | m07 | m08 | m09 | m10 |
| p |  |  |  |  |  |  |  |  |  |  |  |  |  |  |  |  |  |  |  |  |  |  |  |  |  |  |  |  |  |  |  |  |  |
| p001 | 12.5 | 17.5 | 25.0 | 20.0 | 27.5 | 60.0 | 67.5 | 95.0 | 97.5 | 100.0 | 100.0 | 32.5 | 42.5 | 40.0 | 52.5 | 57.5 | 60.0 | 75.0 | 80.0 | 85.0 | 82.5 | 82.5 | 12.5 | 5.0 | 17.5 | 22.5 | 42.5 | 42.5 | 65.0 | 85.0 | 90.0 | 97.5 | 97.5 |
| p002 | 15.0 | 0.0 | 5.0 | 15.0 | 22.5 | 37.5 | 57.5 | 70.0 | 87.5 | 90.0 | 97.5 | 17.5 | 20.0 | 30.0 | 32.5 | 40.0 | 55.0 | 75.0 | 75.0 | 85.0 | 82.5 | 90.0 | 22.5 | 25.0 | 27.5 | 35.0 | 45.0 | 60.0 | 72.5 | 82.5 | 90.0 | 92.5 | 90.0 |
| p003 | 12.5 | 15.0 | 15.0 | 20.0 | 47.5 | 67.5 | 77.5 | 97.5 | 100.0 | 100.0 | 100.0 | 25.0 | 25.0 | 37.5 | 42.5 | 60.0 | 52.5 | 72.5 | 65.0 | 65.0 | 77.5 | 85.0 | 30.0 | 37.5 | 45.0 | 62.5 | 70.0 | 75.0 | 87.5 | 85.0 | 85.0 | 97.5 | 97.5 |
| p004 | 5.0 | 7.5 | 20.0 | 12.5 | 32.5 | 32.5 | 60.0 | 80.0 | 87.5 | 85.0 | 87.5 | 20.0 | 30.0 | 35.0 | 22.5 | 40.0 | 45.0 | 45.0 | 52.5 | 65.0 | 57.5 | 57.5 | 22.5 | 27.5 | 37.5 | 27.5 | 37.5 | 57.5 | 67.5 | 57.5 | 77.5 | 80.0 | 87.5 |
| p005 | 0.0 | 0.0 | 10.0 | 15.0 | 20.0 | 22.5 | 45.0 | 60.0 | 82.5 | 77.5 | 85.0 | 20.0 | 20.0 | 30.0 | 27.5 | 35.0 | 27.5 | 45.0 | 40.0 | 57.5 | 65.0 | 55.0 | 5.0 | 2.5 | 15.0 | 27.5 | 25.0 | 35.0 | 52.5 | 60.0 | 82.5 | 87.5 | 87.5 |
| p006 | 5.0 | 7.5 | 2.5 | 7.5 | 17.5 | 45.0 | 77.5 | 95.0 | 95.0 | 100.0 | 100.0 | 17.5 | 17.5 | 22.5 | 35.0 | 37.5 | 47.5 | 62.5 | 67.5 | 75.0 | 85.0 | 87.5 | 5.0 | 7.5 | 7.5 | 5.0 | 35.0 | 40.0 | 70.0 | 82.5 | 97.5 | 97.5 | 100.0 |
| p007 | 12.5 | 10.0 | 15.0 | 22.5 | 15.0 | 32.5 | 60.0 | 80.0 | 90.0 | 95.0 | 97.5 | 37.5 | 55.0 | 57.5 | 45.0 | 55.0 | 65.0 | 72.5 | 92.5 | 95.0 | 87.5 | 87.5 | 10.0 | 10.0 | 20.0 | 22.5 | 25.0 | 45.0 | 60.0 | 70.0 | 85.0 | 90.0 | 95.0 |
| p008 | 17.5 | 27.5 | 25.0 | 30.0 | 42.5 | 60.0 | 62.5 | 90.0 | 95.0 | 95.0 | 100.0 | 60.0 | 67.5 | 65.0 | 62.5 | 67.5 | 72.5 | 80.0 | 90.0 | 95.0 | 92.5 | 92.5 | 15.0 | 15.0 | 30.0 | 32.5 | 30.0 | 42.5 | 65.0 | 80.0 | 97.5 | 92.5 | 97.5 |
| p009 | 5.0 | 2.5 | 0.0 | 5.0 | 25.0 | 35.0 | 62.5 | 80.0 | 95.0 | 97.5 | 100.0 | 10.0 | 7.5 | 20.0 | 20.0 | 27.5 | 62.5 | 65.0 | 80.0 | 85.0 | 85.0 | 87.5 | 2.5 | 2.5 | 5.0 | 15.0 | 22.5 | 45.0 | 55.0 | 87.5 | 85.0 | 95.0 | 97.5 |
| p010 | 15.0 | 22.5 | 30.0 | 27.5 | 25.0 | 60.0 | 70.0 | 85.0 | 97.5 | 95.0 | 100.0 | 35.0 | 37.5 | 37.5 | 50.0 | 55.0 | 60.0 | 70.0 | 70.0 | 77.5 | 70.0 | 85.0 | 17.5 | 35.0 | 37.5 | 37.5 | 40.0 | 40.0 | 52.5 | 70.0 | 87.5 | 82.5 | 85.0 |
| p011 | 2.5 | 10.0 | 10.0 | 7.5 | 20.0 | 40.0 | 77.5 | 90.0 | 92.5 | 95.0 | 100.0 | 32.5 | 25.0 | 42.5 | 45.0 | 57.5 | 67.5 | 82.5 | 92.5 | 100.0 | 90.0 | 95.0 | 5.0 | 5.0 | 7.5 | 10.0 | 27.5 | 25.0 | 45.0 | 80.0 | 90.0 | 100.0 | 97.5 |
| p012 | 12.5 | 7.5 | 25.0 | 37.5 | 47.5 | 67.5 | 85.0 | 100.0 | 95.0 | 100.0 | 95.0 | 37.5 | 45.0 | 45.0 | 45.0 | 57.5 | 62.5 | 85.0 | 85.0 | 92.5 | 95.0 | 87.5 | 25.0 | 27.5 | 32.5 | 25.0 | 45.0 | 55.0 | 62.5 | 70.0 | 87.5 | 90.0 | 95.0 |
| p013 | 2.5 | 2.5 | 2.5 | 12.5 | 22.5 | 27.5 | 62.5 | 72.5 | 90.0 | 92.5 | 95.0 | 37.5 | 32.5 | 45.0 | 45.0 | 60.0 | 65.0 | 70.0 | 77.5 | 92.5 | 80.0 | 80.0 | 0.0 | 12.5 | 15.0 | 17.5 | 25.0 | 35.0 | 57.5 | 77.5 | 92.5 | 87.5 | 97.5 |
| p014 | 5.0 | 2.5 | 10.0 | 10.0 | 22.5 | 50.0 | 62.5 | 82.5 | 97.5 | 97.5 | 100.0 | 10.0 | 2.5 | 30.0 | 12.5 | 22.5 | 25.0 | 57.5 | 65.0 | 75.0 | 75.0 | 80.0 | 5.0 | 7.5 | 10.0 | 25.0 | 30.0 | 50.0 | 62.5 | 80.0 | 95.0 | 97.5 | 95.0 |
| p015 | 7.5 | 5.0 | 10.0 | 17.5 | 35.0 | 37.5 | 67.5 | 82.5 | 95.0 | 97.5 | 100.0 | 27.5 | 22.5 | 30.0 | 35.0 | 37.5 | 42.5 | 50.0 | 55.0 | 55.0 | 55.0 | 62.5 | 12.5 | 7.5 | 7.5 | 35.0 | 22.5 | 42.5 | 52.5 | 65.0 | 75.0 | 90.0 | 90.0 |
| p016 | 10.0 | 5.0 | 10.0 | 17.5 | 27.5 | 47.5 | 62.5 | 80.0 | 90.0 | 100.0 | 100.0 | 37.5 | 45.0 | 50.0 | 52.5 | 62.5 | 80.0 | 80.0 | 85.0 | 97.5 | 95.0 | 97.5 | 5.0 | 15.0 | 17.5 | 17.5 | 22.5 | 42.5 | 62.5 | 82.5 | 95.0 | 95.0 | 97.5 |
| p019 | 5.0 | 2.5 | 10.0 | 7.5 | 22.5 | 52.5 | 77.5 | 95.0 | 97.5 | 100.0 | 100.0 | 35.0 | 30.0 | 37.5 | 40.0 | 47.5 | 47.5 | 72.5 | 85.0 | 82.5 | 82.5 | 92.5 | 7.5 | 5.0 | 15.0 | 22.5 | 30.0 | 47.5 | 72.5 | 90.0 | 95.0 | 97.5 | 100.0 |
| p020 | 27.5 | 27.5 | 32.5 | 35.0 | 40.0 | 60.0 | 72.5 | 75.0 | 87.5 | 90.0 | 87.5 | 20.0 | 40.0 | 20.0 | 40.0 | 40.0 | 37.5 | 40.0 | 37.5 | 40.0 | 47.5 | 40.0 | 27.5 | 35.0 | 42.5 | 45.0 | 52.5 | 65.0 | 67.5 | 77.5 | 90.0 | 85.0 | 87.5 |
| p021 | 2.5 | 2.5 | 5.0 | 10.0 | 17.5 | 32.5 | 45.0 | 70.0 | 85.0 | 95.0 | 100.0 | 17.5 | 27.5 | 27.5 | 30.0 | 42.5 | 52.5 | 52.5 | 55.0 | 65.0 | 82.5 | 75.0 | 2.5 | 5.0 | 5.0 | 5.0 | 7.5 | 20.0 | 25.0 | 45.0 | 60.0 | 87.5 | 87.5 |
| p022 | 5.0 | 5.0 | 10.0 | 17.5 | 32.5 | 47.5 | 60.0 | 77.5 | 95.0 | 100.0 | 100.0 | 22.5 | 20.0 | 22.5 | 30.0 | 40.0 | 57.5 | 72.5 | 75.0 | 80.0 | 90.0 | 90.0 | 5.0 | 7.5 | 12.5 | 15.0 | 35.0 | 45.0 | 57.5 | 77.5 | 87.5 | 100.0 | 95.0 |
| p023 | 7.5 | 2.5 | 12.5 | 20.0 | 32.5 | 52.5 | 75.0 | 90.0 | 97.5 | 100.0 | 100.0 | 20.0 | 20.0 | 15.0 | 25.0 | 37.5 | 42.5 | 55.0 | 67.5 | 70.0 | 75.0 | 80.0 | 15.0 | 17.5 | 7.5 | 25.0 | 32.5 | 50.0 | 67.5 | 85.0 | 92.5 | 95.0 | 100.0 |
| p024 | 10.0 | 17.5 | 15.0 | 20.0 | 27.5 | 55.0 | 80.0 | 92.5 | 97.5 | 97.5 | 100.0 | 25.0 | 30.0 | 27.5 | 37.5 | 30.0 | 40.0 | 55.0 | 67.5 | 72.5 | 72.5 | 80.0 | 12.5 | 10.0 | 20.0 | 30.0 | 35.0 | 45.0 | 72.5 | 82.5 | 95.0 | 97.5 | 97.5 |
| p025 | 20.0 | 17.5 | 27.5 | 22.5 | 42.5 | 50.0 | 60.0 | 70.0 | 75.0 | 90.0 | 95.0 | 45.0 | 42.5 | 37.5 | 42.5 | 50.0 | 47.5 | 45.0 | 57.5 | 55.0 | 60.0 | 70.0 | 17.5 | 22.5 | 22.5 | 32.5 | 32.5 | 42.5 | 40.0 | 72.5 | 90.0 | 90.0 | 85.0 |
| p026 | 7.5 | 7.5 | 5.0 | 7.5 | 22.5 | 47.5 | 70.0 | 87.5 | 97.5 | 100.0 | 100.0 | 20.0 | 30.0 | 17.5 | 30.0 | 35.0 | 40.0 | 52.5 | 50.0 | 67.5 | 70.0 | 72.5 | 15.0 | 25.0 | 22.5 | 30.0 | 40.0 | 52.5 | 67.5 | 80.0 | 87.5 | 87.5 | 97.5 |
| p027 | 7.5 | 17.5 | 20.0 | 30.0 | 32.5 | 50.0 | 75.0 | 92.5 | 92.5 | 100.0 | 100.0 | 20.0 | 35.0 | 37.5 | 52.5 | 60.0 | 70.0 | 85.0 | 85.0 | 82.5 | 90.0 | 85.0 | 17.5 | 27.5 | 30.0 | 50.0 | 55.0 | 62.5 | 70.0 | 87.5 | 87.5 | 97.5 | 100.0 |
| p028 | 7.5 | 2.5 | 2.5 | 15.0 | 25.0 | 27.5 | 55.0 | 70.0 | 85.0 | 97.5 | 97.5 | 32.5 | 25.0 | 37.5 | 32.5 | 42.5 | 55.0 | 72.5 | 67.5 | 75.0 | 80.0 | 85.0 | 2.5 | 10.0 | 10.0 | 17.5 | 22.5 | 22.5 | 42.5 | 67.5 | 85.0 | 90.0 | 90.0 |
| p029 | 0.0 | 5.0 | 5.0 | 7.5 | 22.5 | 25.0 | 65.0 | 67.5 | 82.5 | 90.0 | 95.0 | 12.5 | 15.0 | 20.0 | 17.5 | 25.0 | 35.0 | 52.5 | 57.5 | 67.5 | 77.5 | 70.0 | 2.5 | 5.0 | 10.0 | 12.5 | 17.5 | 30.0 | 50.0 | 72.5 | 82.5 | 90.0 | 87.5 |
| p030 | 35.0 | 37.5 | 40.0 | 52.5 | 45.0 | 65.0 | 85.0 | 85.0 | 100.0 | 95.0 | 100.0 | 62.5 | 55.0 | 57.5 | 65.0 | 70.0 | 67.5 | 70.0 | 82.5 | 80.0 | 100.0 | 85.0 | 32.5 | 45.0 | 40.0 | 55.0 | 60.0 | 60.0 | 72.5 | 72.5 | 75.0 | 87.5 | 87.5 |

## Save Table to File¶

The pandas tables are saved as csv files. They are then re-used in the following notebooks. The csv files can also easily be imported with other toolboxes/software.

In [25]:

```
def savePandas(where,df,csv):
    os.chdir(where)
    df.to_csv(csv)
```

In [26]:

```
my_folder = './data/'

# save table with all 30 subjects
savePandas(my_folder,avg_between_df,'Exp1AvgResultsAll.txt')
# save revised table with 28 subjects
savePandas(my_folder,rev_avg_between_df,'Exp1AvgResults.txt')

savePandas(my_folder,pt1_between_df,'Exp1Pt1ResultsAll.txt')
savePandas(my_folder,rev_pt1_between_df,'Exp1Pt1MainResults.txt')

savePandas(my_folder,pt2_between_df,'Exp1Pt2ResultsAll.txt')
savePandas(my_folder,rev_pt2_between_df,'Exp1Pt2MainResults.txt')
```

# Reaction Times¶

While reaction times were also extracted, their analysis is not part of the publication. However, their timecourse is well aligned with the accuracy data (the more difficult a decision, the longer the RTs).

In [27]:

```
def findentry_rt_sub(d, cond,grade,filename):
    filecontent = open(filename,'r')
    r  = 0
    for entry in filecontent:
        if entry.find(cond) != -1 and entry.find(grade) != -1:
            #print entry
            r += 1
        if entry.find('Response') != -1 and r == 1:
            #print entry
            r = 0
            if grade in d[cond]:
                d[cond][grade].append(float(entry.split()[-2]))
            else:
                d[cond][grade] = []
    return d
```

In [28]:

```
os.chdir("../Exp1/")
```

In [29]:

```
def participant_rt(List,nr):
    d = {'orig':{},'noe':{},'nom':{}}
    for cond in ['noe','nom','orig']:
        for grade in ['_00_','_01_','_02_','_03_','_04_','_05_','_06_','_07_','_08_','_09_','_10_']:
            d = findentry_rt_sub(d,cond,grade,List[nr])
            d = findentry_rt_sub(d,cond,grade,List[nr+1])
    
    d_median = {'orig':{},'noe':{},'nom':{}}
    for cond in ['noe','nom','orig']:
        for grade in ['_00_','_01_','_02_','_03_','_04_','_05_','_06_','_07_','_08_','_09_','_10_']:
            d_median[cond][grade] = np.median(d[cond][grade])
    
        for key in d[cond]:
            plt.hist(d[cond][key])

    return pd.DataFrame(d_median)/10
```

In [30]:

```
participant_rt(pList,0)
```

Out[30]:

|  | noe | nom | orig |
| --- | --- | --- | --- |
| \_00\_ | 1420.5 | 1224.4 | 1083.0 |
| \_01\_ | 1523.4 | 1244.4 | 1159.0 |
| \_02\_ | 1374.2 | 1114.7 | 1248.9 |
| \_03\_ | 1663.6 | 1148.2 | 1351.1 |
| \_04\_ | 1183.5 | 1300.6 | 1770.1 |
| \_05\_ | 1162.1 | 1559.5 | 1259.4 |
| \_06\_ | 1513.5 | 1346.9 | 1370.9 |
| \_07\_ | 1279.6 | 1431.7 | 1283.8 |
| \_08\_ | 1099.5 | 1270.8 | 1076.3 |
| \_09\_ | 1241.9 | 1170.4 | 976.4 |
| \_10\_ | 1163.2 | 960.1 | 895.9 |

In [31]:

```
def make_rt_sample(pList):
    # make the first subject to establish the df-structure
    df = participant_rt(pList,0)
    df.index = [["p001"]*len(df), df.index ]
    
    # make the rest of the sample
    for i in range(2,len(pList))[::2]:
        this_df = participant_rt(pList,i)
        # get the naming of the subject right
        thisName = "00"+str(i/2+1)
        this_df.index = [ ["p"+thisName[-3:] ]*len(this_df), this_df.index ]
        # add this subject to sample-df
        df = pd.concat([df,this_df])
    # finalising the df
    df.index.names = ['participant','grade']
    df.columns = ['mouth','eyes','whole']
    return df
```

In [32]:

```
rt_between = make_rt_sample(pList).unstack('grade')
rt_between
```

Out[32]:

|  | mouth | | | | | | | | | | | eyes | | | | | | | | | | | whole | | | | | | | | | | |
| --- | --- | --- | --- | --- | --- | --- | --- | --- | --- | --- | --- | --- | --- | --- | --- | --- | --- | --- | --- | --- | --- | --- | --- | --- | --- | --- | --- | --- | --- | --- | --- | --- | --- |
| grade | \_00\_ | \_01\_ | \_02\_ | \_03\_ | \_04\_ | \_05\_ | \_06\_ | \_07\_ | \_08\_ | \_09\_ | \_10\_ | \_00\_ | \_01\_ | \_02\_ | \_03\_ | \_04\_ | \_05\_ | \_06\_ | \_07\_ | \_08\_ | \_09\_ | \_10\_ | \_00\_ | \_01\_ | \_02\_ | \_03\_ | \_04\_ | \_05\_ | \_06\_ | \_07\_ | \_08\_ | \_09\_ | \_10\_ |
| participant |  |  |  |  |  |  |  |  |  |  |  |  |  |  |  |  |  |  |  |  |  |  |  |  |  |  |  |  |  |  |  |  |  |
| p001 | 1420.5 | 1523.4 | 1374.2 | 1663.6 | 1183.5 | 1162.1 | 1513.5 | 1279.6 | 1099.5 | 1241.9 | 1163.2 | 1224.4 | 1244.4 | 1114.7 | 1148.2 | 1300.6 | 1559.5 | 1346.9 | 1431.7 | 1270.8 | 1170.4 | 960.1 | 1083.0 | 1159.0 | 1248.9 | 1351.1 | 1770.1 | 1259.4 | 1370.9 | 1283.8 | 1076.3 | 976.4 | 895.9 |
| p002 | 1847.2 | 1449.9 | 1271.7 | 1425.5 | 1212.3 | 1779.9 | 1377.9 | 1513.8 | 1273.6 | 1462.6 | 1106.3 | 1287.6 | 1071.4 | 1314.1 | 1675.1 | 1524.8 | 1347.9 | 1287.7 | 1124.7 | 1028.5 | 1033.2 | 1001.6 | 1045.3 | 1240.2 | 1210.3 | 1324.4 | 1377.3 | 1126.5 | 1609.6 | 1427.6 | 1052.6 | 1139.2 | 1018.9 |
| p003 | 1476.3 | 1329.5 | 1289.1 | 1479.6 | 1504.4 | 1319.4 | 1576.6 | 1333.0 | 1516.5 | 1316.3 | 1055.6 | 1476.9 | 1572.0 | 1526.9 | 1121.4 | 1300.6 | 1211.1 | 1151.6 | 1336.0 | 1075.4 | 1012.3 | 915.3 | 1805.0 | 1737.6 | 1919.8 | 1677.7 | 2442.7 | 1831.1 | 1761.1 | 1571.1 | 1255.6 | 1211.3 | 1139.5 |
| p004 | 756.7 | 739.8 | 722.0 | 802.5 | 835.8 | 765.4 | 852.2 | 705.9 | 748.0 | 681.7 | 826.7 | 831.6 | 767.6 | 786.5 | 815.5 | 805.9 | 804.4 | 740.0 | 804.2 | 691.7 | 668.1 | 677.1 | 801.9 | 759.4 | 743.7 | 756.1 | 783.7 | 722.6 | 852.0 | 759.8 | 706.1 | 725.3 | 667.3 |
| p005 | 1175.6 | 1012.2 | 1586.3 | 1278.9 | 1380.6 | 1349.2 | 1486.4 | 1264.7 | 1628.1 | 1475.1 | 1650.0 | 1041.4 | 1180.9 | 1377.5 | 1063.5 | 1328.6 | 1453.4 | 1662.1 | 1563.1 | 1215.3 | 1140.4 | 1051.6 | 1390.4 | 1531.4 | 1100.5 | 1303.6 | 1471.6 | 1260.8 | 1628.5 | 1325.1 | 1339.0 | 986.9 | 1164.2 |
| p006 | 1546.9 | 1240.0 | 1634.2 | 1842.3 | 1404.6 | 1476.8 | 1631.7 | 1690.8 | 1611.9 | 1434.5 | 1474.0 | 911.7 | 985.2 | 1160.7 | 1211.1 | 1452.7 | 1479.6 | 1709.3 | 1328.0 | 1313.7 | 1023.1 | 954.6 | 1279.8 | 1257.8 | 1018.0 | 1381.6 | 1435.4 | 1921.7 | 1469.0 | 1634.2 | 1177.8 | 1112.7 | 1048.7 |
| p007 | 1132.6 | 1218.7 | 1298.8 | 1092.1 | 1135.6 | 1159.7 | 1100.9 | 1005.4 | 1148.7 | 996.8 | 829.6 | 981.2 | 962.7 | 957.6 | 936.2 | 1018.0 | 1188.1 | 940.6 | 1114.4 | 1034.8 | 908.6 | 861.4 | 909.2 | 1023.6 | 1119.7 | 1028.8 | 1102.3 | 1211.2 | 1201.6 | 1062.2 | 960.5 | 906.6 | 833.2 |
| p008 | 877.1 | 884.4 | 805.0 | 799.6 | 804.5 | 952.8 | 819.3 | 824.6 | 708.1 | 693.1 | 819.4 | 674.9 | 724.0 | 770.1 | 691.9 | 857.4 | 876.6 | 833.1 | 685.5 | 815.6 | 853.8 | 691.2 | 751.2 | 757.1 | 811.1 | 666.1 | 763.1 | 796.3 | 705.5 | 892.3 | 650.5 | 645.3 | 627.3 |
| p009 | 1279.7 | 1565.0 | 1415.6 | 1600.5 | 2276.4 | 2462.1 | 1995.5 | 1763.8 | 2359.7 | 1634.9 | 1559.6 | 1060.4 | 1311.1 | 1121.4 | 1214.6 | 1561.7 | 1474.4 | 1415.7 | 1442.9 | 1210.1 | 1016.0 | 1060.9 | 1042.9 | 1095.5 | 1293.4 | 1391.7 | 1740.2 | 1612.5 | 1872.4 | 1514.4 | 1209.5 | 1164.2 | 1087.1 |
| p010 | 1198.9 | 890.4 | 912.1 | 887.1 | 1055.7 | 851.5 | 881.3 | 918.2 | 860.1 | 833.4 | 914.6 | 862.4 | 775.6 | 935.7 | 982.2 | 908.8 | 956.0 | 967.9 | 823.5 | 791.6 | 852.3 | 751.6 | 918.1 | 953.2 | 953.5 | 906.0 | 985.6 | 1017.7 | 883.1 | 812.6 | 774.6 | 825.8 | 764.2 |
| p011 | 1403.9 | 1691.6 | 1450.3 | 1877.8 | 1772.6 | 1483.1 | 1522.4 | 1140.3 | 1193.6 | 1017.8 | 1042.2 | 873.9 | 873.8 | 1027.3 | 1447.6 | 1250.8 | 1761.6 | 1744.2 | 1422.9 | 1210.4 | 976.6 | 795.9 | 882.8 | 797.2 | 860.1 | 1216.3 | 1671.6 | 1752.5 | 1439.7 | 1065.6 | 922.2 | 817.6 | 813.9 |
| p012 | 1343.3 | 1318.2 | 1543.8 | 1274.0 | 1605.2 | 1238.9 | 1319.4 | 1171.9 | 1073.9 | 1026.9 | 1008.0 | 1388.5 | 1355.2 | 2919.3 | 2331.0 | 1817.4 | 1735.6 | 2387.0 | 2879.6 | 1314.2 | 1252.2 | 1004.6 | 1290.4 | 1683.8 | 1591.0 | 1714.3 | 2038.9 | 1284.0 | 1516.6 | 1097.1 | 1202.2 | 1079.1 | 959.9 |
| p013 | 1133.3 | 1384.3 | 1223.8 | 1256.7 | 1356.4 | 1269.5 | 1101.9 | 1215.6 | 1121.2 | 1111.9 | 1130.0 | 994.1 | 1115.6 | 963.0 | 1039.6 | 1457.3 | 1152.8 | 1086.2 | 1031.7 | 1065.7 | 1079.8 | 879.7 | 899.8 | 1022.4 | 1108.0 | 968.1 | 1157.1 | 1142.1 | 1346.0 | 1126.4 | 965.2 | 920.2 | 778.2 |
| p014 | 1906.7 | 2190.1 | 1979.2 | 1834.3 | 2002.7 | 2051.1 | 2020.4 | 1557.3 | 1552.1 | 1678.4 | 1644.8 | 1702.1 | 2145.0 | 1668.2 | 1962.3 | 2351.3 | 2042.1 | 2419.9 | 2134.9 | 1751.5 | 1235.2 | 1256.5 | 1744.1 | 1776.2 | 1781.0 | 2162.5 | 2371.9 | 2014.0 | 2307.7 | 2003.6 | 1721.8 | 1477.9 | 1424.3 |
| p015 | 1134.2 | 1150.9 | 943.1 | 1109.1 | 1047.7 | 945.7 | 1018.3 | 978.3 | 1194.6 | 1002.2 | 930.5 | 1137.9 | 1343.6 | 1163.5 | 1160.6 | 1157.4 | 1254.3 | 1615.9 | 1347.6 | 1209.5 | 953.8 | 975.8 | 1383.7 | 1231.7 | 1164.1 | 1309.8 | 1423.6 | 1412.6 | 1831.7 | 1262.5 | 1262.0 | 1088.2 | 795.2 |
| p016 | 1265.4 | 1187.0 | 1255.7 | 1068.2 | 1103.9 | 991.7 | 1282.3 | 987.3 | 1136.6 | 961.8 | 942.2 | 1113.6 | 1031.6 | 979.9 | 1049.0 | 1057.7 | 1471.6 | 1046.8 | 1042.1 | 1066.1 | 817.7 | 883.9 | 893.2 | 1060.4 | 970.2 | 943.3 | 1042.0 | 1266.5 | 1019.0 | 1103.6 | 1064.6 | 940.9 | 882.4 |
| p017 | 819.5 | 722.8 | 1021.8 | 832.7 | 825.1 | 946.6 | 842.7 | 863.7 | 921.0 | 836.0 | 727.3 | 939.8 | 824.0 | 809.7 | 935.7 | 804.5 | 1321.0 | 1001.2 | 916.1 | 733.3 | 786.6 | 736.0 | 775.8 | 956.6 | 938.4 | 919.7 | 835.5 | 755.5 | 697.9 | 912.6 | 825.1 | 719.7 | 687.7 |
| p018 | 549.1 | 510.9 | 599.7 | 537.6 | 601.8 | 523.9 | 618.6 | 634.9 | 549.3 | 524.7 | 495.6 | 698.1 | 490.9 | 628.3 | 693.1 | 485.9 | 615.6 | 680.4 | 593.7 | 657.7 | 645.3 | 619.0 | 692.5 | 730.3 | 606.5 | 699.0 | 708.3 | 607.6 | 649.1 | 676.2 | 631.7 | 671.6 | 640.1 |
| p019 | 1103.8 | 1600.4 | 1364.2 | 1368.2 | 1567.6 | 1692.2 | 1122.3 | 1139.8 | 1267.5 | 1125.0 | 883.5 | 883.5 | 1044.2 | 1211.2 | 1180.5 | 1301.8 | 1641.9 | 1668.1 | 1183.1 | 868.9 | 983.9 | 889.4 | 892.4 | 996.5 | 922.7 | 1192.3 | 1280.2 | 1196.9 | 1443.6 | 1139.7 | 842.6 | 858.3 | 763.9 |
| p020 | 905.8 | 808.0 | 887.7 | 862.3 | 903.6 | 879.4 | 961.5 | 791.9 | 729.8 | 828.6 | 850.4 | 1077.2 | 985.4 | 943.6 | 1036.4 | 1098.5 | 935.6 | 883.5 | 892.7 | 805.2 | 710.5 | 769.7 | 1071.6 | 1071.7 | 976.6 | 994.9 | 984.8 | 1020.5 | 875.6 | 839.7 | 771.0 | 827.5 | 723.8 |
| p021 | 1321.4 | 1108.2 | 1224.2 | 1227.3 | 1386.7 | 1435.6 | 1353.7 | 1585.5 | 1959.6 | 1363.6 | 1127.7 | 869.5 | 810.4 | 905.8 | 1016.8 | 1109.9 | 1120.1 | 1132.8 | 1172.7 | 1295.7 | 942.7 | 954.1 | 1196.1 | 1058.7 | 1015.6 | 1140.0 | 1473.7 | 1411.7 | 1797.4 | 1918.0 | 1224.1 | 922.5 | 885.0 |
| p022 | 1427.0 | 1305.8 | 1205.1 | 1361.0 | 1461.9 | 1313.3 | 1425.5 | 1181.7 | 1071.6 | 1335.7 | 1130.5 | 930.6 | 1077.6 | 1026.2 | 1071.6 | 1266.6 | 1334.7 | 1331.7 | 1254.4 | 936.7 | 1023.4 | 947.4 | 1082.7 | 1143.5 | 1048.7 | 1229.2 | 1503.6 | 1199.2 | 1399.0 | 1007.4 | 975.7 | 983.6 | 955.6 |
| p023 | 2070.2 | 1930.1 | 1888.2 | 1882.0 | 2329.5 | 1957.8 | 2046.5 | 1967.1 | 1715.5 | 1689.3 | 1751.7 | 1817.7 | 1506.8 | 1455.3 | 1638.1 | 1524.2 | 2106.7 | 2956.8 | 1851.3 | 1430.4 | 1119.6 | 1151.6 | 1231.7 | 1560.0 | 1383.6 | 2035.4 | 2122.1 | 2910.9 | 2327.7 | 1532.4 | 1653.2 | 1064.5 | 1275.7 |
| p024 | 1230.8 | 1183.6 | 1293.5 | 1233.8 | 1385.8 | 1171.0 | 1547.3 | 1310.5 | 1274.4 | 1350.0 | 1038.6 | 959.6 | 1060.7 | 1010.0 | 1111.6 | 1047.7 | 1170.9 | 1168.2 | 967.7 | 907.0 | 850.5 | 797.8 | 924.6 | 1163.8 | 1080.3 | 1076.7 | 1044.2 | 1549.3 | 1649.4 | 1211.4 | 936.0 | 1017.9 | 870.0 |
| p025 | 998.1 | 1024.7 | 1072.8 | 1028.9 | 1199.2 | 983.7 | 1028.4 | 1044.0 | 995.1 | 1026.0 | 1172.9 | 904.0 | 957.7 | 983.6 | 979.9 | 977.0 | 823.7 | 1165.3 | 1161.7 | 839.6 | 955.1 | 826.6 | 930.6 | 977.9 | 1034.7 | 1107.5 | 1062.7 | 1309.5 | 935.6 | 1116.9 | 860.8 | 964.8 | 842.3 |
| p026 | 1266.7 | 1351.7 | 1300.3 | 1187.9 | 1169.2 | 1159.3 | 1182.2 | 1337.2 | 1216.2 | 1566.4 | 1239.4 | 1225.5 | 953.8 | 1499.9 | 1618.0 | 1472.4 | 1295.6 | 1169.9 | 1215.6 | 1090.6 | 1083.2 | 1010.7 | 1027.8 | 1079.7 | 1289.9 | 1303.6 | 1385.8 | 1500.0 | 1385.4 | 1183.6 | 891.0 | 943.9 | 863.1 |
| p027 | 1273.6 | 1195.3 | 1285.0 | 1007.6 | 1276.9 | 1183.6 | 1059.5 | 954.1 | 1093.9 | 883.6 | 1010.9 | 1230.3 | 1095.6 | 1127.6 | 973.8 | 1322.2 | 1086.4 | 913.4 | 936.4 | 883.9 | 845.8 | 911.7 | 1031.6 | 1141.0 | 1021.0 | 977.7 | 1218.5 | 1292.5 | 902.0 | 963.9 | 961.7 | 921.6 | 817.4 |
| p028 | 1285.3 | 1368.5 | 1479.7 | 1541.1 | 1481.0 | 1594.9 | 1403.1 | 1578.6 | 1297.1 | 1554.7 | 1236.6 | 1121.4 | 1047.4 | 1194.3 | 1137.3 | 1417.5 | 1244.0 | 1499.0 | 1248.8 | 1141.5 | 991.7 | 918.9 | 883.3 | 970.7 | 1044.2 | 964.5 | 1028.6 | 1118.8 | 1284.2 | 1045.3 | 1066.0 | 967.7 | 983.8 |
| p029 | 2201.4 | 2532.7 | 2143.2 | 2175.6 | 2122.8 | 2331.5 | 2367.6 | 2337.7 | 2343.6 | 2148.8 | 2058.8 | 2055.7 | 2066.2 | 2173.5 | 2165.1 | 2144.3 | 2036.8 | 1916.0 | 1827.0 | 1657.8 | 1501.9 | 1603.9 | 2215.7 | 2344.9 | 2149.1 | 2274.0 | 2516.8 | 1951.6 | 2534.6 | 2239.6 | 2127.6 | 1815.8 | 2219.3 |
| p030 | 812.4 | 784.5 | 884.4 | 756.1 | 871.7 | 756.6 | 986.8 | 867.1 | 737.6 | 754.1 | 790.0 | 848.4 | 912.6 | 804.7 | 861.4 | 917.2 | 803.6 | 789.0 | 828.2 | 826.5 | 806.8 | 710.6 | 892.9 | 904.8 | 969.8 | 1064.2 | 930.7 | 900.2 | 767.0 | 726.9 | 812.6 | 730.0 | 756.2 |

Excluding the non-compliant participants:

In [33]:

```
rev_rt_between = pd.concat([rt_between[0:16], rt_between[18:] ])
```

In [40]:

```
for cond in rev_rt_between.columns.levels[0]:
    errorbar(range(len(rev_rt_between[cond].mean())),
             rev_rt_between[cond].mean(),
             rev_rt_between[cond].std(),
             label=cond)
legend(loc='best')
show()
```

In [41]:

```
# save table with all 30 subjects
savePandas(my_folder,rt_between,'Exp1RtMedian.txt')
# save revised table with 28 subjects
savePandas(my_folder,rev_rt_between,'Exp1RtMedianAll.txt')
```
