# Supplementary material for: Categorical Perception of Fear and Anger Expressions in Whole, Masked and Composite Faces
Source: PLoS One. 2015 Aug 11;10(8):e0134790. doi: 10.1371/journal.pone.0134790 (PMC4532458; doi:10.1371/journal.pone.0134790)
Supplement: S2 Code — (HTML) [file pone.0134790.s003.html]

SupplementS2\_Experiment2\_Data\_Import


# Supplement S2 of

## Categorical Perception of Fear and Anger Expressions in Whole, Masked and Composite Faces.

# Get Data for Experiment 2 (Composite)¶

This file contains the full code to extract all information from the original logfiles, count the responses and create a table with the main results, which can be used in later analyses.

## import libraries¶

In [1]:

```
import fnmatch # filename matching
import os # navigating directories
import pickle # saving python objects to file

import numpy as np

import pandas as pd
pd.set_option('max_columns', 100)
pd.set_option('max_rows', 1000)

%pylab inline
```

```
Populating the interactive namespace from numpy and matplotlib
```

## get list of logfiles¶

In [2]:

```
def getsubject(whichfolder, whichexperiment):
    os.chdir(whichfolder)
    VpList = []
    for file in os.listdir(whichfolder):
        if fnmatch.fnmatch(file, whichexperiment):
            VpList.append(file)
    return VpList
```

In [3]:

```
IdentList = getsubject('../Exp2/','*.log')
IdentList.sort()

for i in range(0,len(IdentList)):
    print str(i) + ":\t" + IdentList[i]
```

```
0:	Vp001-Vp001_UpDown_Identification.log
1:	Vp002-Vp002_UpDown_Identification.log
2:	Vp003-Vp003_UpDown_Identification.log
3:	Vp004-Vp004_UpDown_Identification.log
4:	Vp005-Vp005_UpDown_Identification.log
5:	Vp006-Vp006_UpDown_Identification.log
6:	Vp007-Vp007_UpDown_Identification.log
7:	Vp008-Vp008_UpDown_Identification.log
8:	Vp009-Vp009_UpDown_Identification.log
9:	Vp010-Vp010_UpDown_Identification.log
10:	Vp011-Vp011_UpDown_Identification.log
11:	Vp012-Vp012_UpDown_Identification.log
12:	Vp013-Vp013_UpDown_Identification.log
13:	Vp014-Vp014_UpDown_Identification.log
14:	Vp015-Vp015_UpDown_Identification.log
15:	Vp016-Vp016_UpDown_Identification.log
16:	Vp017-Vp017_UpDown_Identification.log
17:	Vp018-Vp018_UpDown_Identification.log
18:	Vp019-Vp019_UpDown_Identification.log
19:	Vp020-Vp020_UpDown_Identification.log
20:	Vp021-Vp021_UpDown_Identification.log
21:	Vp022-Vp022_UpDown_Identification.log
22:	Vp023-Vp023_UpDown_Identification.log
23:	Vp024-Vp024_UpDown_Identification.log
24:	Vp025-Vp025_UpDown_Identification.log
25:	Vp026-Vp026_UpDown_Identification.log
26:	Vp027-Vp027_UpDown_Identification.log
27:	Vp028-Vp028_UpDown_Identification.log
28:	Vp029-Vp029_UpDown_Identification.log
29:	Vp030-Vp030_UpDown_Identification.log
```

# Get Responses¶

## Transform each row into dict entry¶

The logfiles have a legacy naming of the composite face conditions which is as follows:

- rating of upper half
  - lower half angry: low10 (as 10 is the number of the 100% angry face)
  - lower half fearful: low00 (as 0 is the number of the 0% angry / 100% fearful face)
- rating of lower half:
  - upper half angry: up10
  - upper half angry: up00

In [4]:

```
def get_ident_data(Vp):
    
    filename = open(Vp,'r')
    d = {'pt1': {'up10':{}, 'low10':{}, 'up00':{}, 'low00':{} },
         'pt2': {'up10':{}, 'low10':{}, 'up00':{}, 'low00':{} }} # basic conditions
    
    i = 1 # count of items
    
    pt_switch = 1
    r = 0
    for entry in filename:  
        # check if experiment part has changed
        if entry.find("pause") != -1: 
            pt_switch = 2
        
        # check if logfile row contains relevant information
        if entry.find("Morph") != -1:
            first = entry.split()[3] # name of target face
            second = entry.split()[5] # name of mask face
            resp = entry.split()[-2] # respose given
            
            # get condition
            if second.find("10_UPPER") != -1:
                cond = 'up10'
            if second.find("10_LOWER") != -1:
                cond = 'low10'
            if second.find("00_UPPER") != -1:
                cond = 'up00'
            if second.find("00_LOWER") != -1:
                cond = 'low00' 
                
            # write to dict
            if pt_switch == 1:
                d['pt1'][cond]["item_" + str(i)] = {"target": first, "mask": second, "resp": resp, "rt": 0.0}
            if pt_switch == 2:
                d['pt2'][cond]["item_" + str(i)] = {"target": first, "mask": second, "resp": resp, "rt": 0.0}
            i += 1
            r += 1
        
        if entry.find('Response') != -1 and i > 1 and r == 1:
            rt = float(entry.split()[-2])/10 # ms
            # write to dict
            if pt_switch == 1:
                d['pt1'][cond]["item_" + str(i-1)]['rt'] = rt
                r = 0
            if pt_switch == 2:
                d['pt2'][cond]["item_" + str(i-1)]['rt'] = rt
                r = 0
            
    return d
```

Example:

In [5]:

```
mydict = get_ident_data(IdentList[0])
mydict['pt1']['low00']['item_1']
```

Out[5]:

```
{'mask': 'MorphM2_00_LOWER.jpg',
 'resp': 'hit',
 'rt': 25198.8,
 'target': 'MorphM2_07_orig.jpg'}
```

## Count the hits for all morphing grades of a certain condition¶

In [7]:

```
def get_ident_morphs(d,pt,cond):
    morphs = ['_00_','_01_','_02_','_03_','_04_','_05_','_06_','_07_','_08_','_09_','_10_']
    d_names = ['m00','m01','m02','m03','m04','m05','m06','m07','m08','m09','m10']
    d_count = {'m00':{},'m01':{},'m02':{},'m03':{},'m04':{},'m05':{},'m06':{},'m07':{},'m08':{},'m09':{},'m10':{}}
    j = 0
    for m in morphs:
        i = 0
        for entry in d[pt][cond]:
            if d[pt][cond][entry]['target'].find(m) != -1:
                if d[pt][cond][entry]['resp'] == 'hit':
                    i += 1
        d_count[d_names[j]] = i
        j += 1
    return d_count
```

## Count hits for all conditions¶

In [8]:

```
def get_ident_cond(d,pt):
    d_conds = {'up10':{}, 'low10':{}, 'up00':{}, 'low00':{} }
    d_names = ['up10','low10','up00','low00']
    i = 0
    for c in d_names:
        d_conds[d_names[i]] = get_ident_morphs(d,pt,d_names[i])
        i += 1
    return d_conds
```

Example:

In [9]:

```
get_ident_cond(mydict,'pt1')
```

Out[9]:

```
{'low00': {'m00': 4,
  'm01': 6,
  'm02': 10,
  'm03': 7,
  'm04': 12,
  'm05': 10,
  'm06': 12,
  'm07': 15,
  'm08': 16,
  'm09': 16,
  'm10': 17},
 'low10': {'m00': 7,
  'm01': 9,
  'm02': 6,
  'm03': 12,
  'm04': 7,
  'm05': 11,
  'm06': 13,
  'm07': 15,
  'm08': 15,
  'm09': 15,
  'm10': 19},
 'up00': {'m00': 7,
  'm01': 4,
  'm02': 6,
  'm03': 8,
  'm04': 7,
  'm05': 9,
  'm06': 8,
  'm07': 9,
  'm08': 9,
  'm09': 11,
  'm10': 12},
 'up10': {'m00': 9,
  'm01': 8,
  'm02': 10,
  'm03': 12,
  'm04': 11,
  'm05': 11,
  'm06': 12,
  'm07': 11,
  'm08': 15,
  'm09': 12,
  'm10': 15}}
```

## Count Hits for both parts¶

In [10]:

```
def get_ident_parts(d):
    pt1 = get_ident_cond(d,'pt1')
    pt2 = get_ident_cond(d,'pt2')
    return pt1,pt2
```

## Transform into Pandas Data Frame¶

00 --> fear  
10 --> anger

In [11]:

```
def make_ident_df(d):
    pt1_df = pd.DataFrame(get_ident_parts(d)[0])/20.*100 # transformation into percent
    pt2_df = pd.DataFrame(get_ident_parts(d)[1])/20.*100
    pt1_df.columns = ["fear_low", "anger_low","fear_high","anger_high" ]
    pt2_df.columns = ["fear_low", "anger_low","fear_high","anger_high" ]
    avg_df = (pt1_df + pt2_df)/2
    return pt1_df, pt2_df, avg_df
```

In [12]:

```
pt1,pt2,avg = make_ident_df(get_ident_data(IdentList[0]))
```

Example:

In [13]:

```
avg
```

Out[13]:

|  | fear\_low | anger\_low | fear\_high | anger\_high |
| --- | --- | --- | --- | --- |
| m00 | 15.0 | 25.0 | 35.0 | 57.5 |
| m01 | 25.0 | 32.5 | 32.5 | 42.5 |
| m02 | 42.5 | 32.5 | 42.5 | 55.0 |
| m03 | 40.0 | 45.0 | 40.0 | 57.5 |
| m04 | 42.5 | 37.5 | 42.5 | 62.5 |
| m05 | 50.0 | 52.5 | 45.0 | 50.0 |
| m06 | 55.0 | 62.5 | 42.5 | 57.5 |
| m07 | 62.5 | 67.5 | 42.5 | 57.5 |
| m08 | 77.5 | 75.0 | 45.0 | 67.5 |
| m09 | 77.5 | 75.0 | 52.5 | 60.0 |
| m10 | 80.0 | 87.5 | 50.0 | 70.0 |

## Do This for all Participants¶

In [14]:

```
def make_ident_sample(VpList):
    # make the first subject to establish the df-structure
    pt1_df, pt2_df, avg_df = make_ident_df(get_ident_data(VpList[0]))
    pt1_df.index = [["p001"]*len(pt1_df), pt1_df.index ]
    pt2_df.index = [["p001"]*len(pt2_df), pt2_df.index ]
    avg_df.index = [["p001"]*len(avg_df), avg_df.index ]
    
    # make the rest of the sample
    for vp_nr in range(1,len(VpList)):
        vp_pt1,vp_pt2,vp_avg = make_ident_df(get_ident_data(VpList[vp_nr]))
        # get the naming of the subject right
        if vp_nr < 10-1:
            vp_pt1.index = [["p00"+str(vp_nr+1) ]*len(vp_pt1), vp_pt1.index ]
            vp_pt2.index = [["p00"+str(vp_nr+1) ]*len(vp_pt2), vp_pt2.index ]
            vp_avg.index = [["p00"+str(vp_nr+1) ]*len(vp_avg), vp_avg.index ]
        if vp_nr >=10-1:
            vp_pt1.index = [[ "p0"+str(vp_nr+1) ]*len(vp_pt1), vp_pt1.index ]
            vp_pt2.index = [[ "p0"+str(vp_nr+1) ]*len(vp_pt2), vp_pt2.index ]
            vp_avg.index = [[ "p0"+str(vp_nr+1) ]*len(vp_avg), vp_avg.index ]
        # add this subject to sample-df
        pt1_df = pd.concat([pt1_df,vp_pt1])
        pt2_df = pd.concat([pt2_df,vp_pt2])
        avg_df = pd.concat([avg_df,vp_avg])
    # finalising the df
    pt1_df.index.names = ['p','grade']
    pt2_df.index.names = ['p','grade']
    avg_df.index.names = ['p','grade']
    
    pt1_df.columns = ['fearLOW','angerLOW','fearUP','angerUP']
    pt2_df.columns = ['fearLOW','angerLOW','fearUP','angerUP']
    avg_df.columns = ['fearLOW','angerLOW','fearUP','angerUP']
    
    return pt1_df, pt2_df, avg_df
```

In [15]:

```
pt1_df, pt2_df, avg_df = make_ident_sample(IdentList)
avg_df
```

Out[15]:

|  |  | fearLOW | angerLOW | fearUP | angerUP |
| --- | --- | --- | --- | --- | --- |
| p | grade |  |  |  |  |
| p001 | m00 | 15.0 | 25.0 | 35.0 | 57.5 |
|  | m01 | 25.0 | 32.5 | 32.5 | 42.5 |
|  | m02 | 42.5 | 32.5 | 42.5 | 55.0 |
|  | m03 | 40.0 | 45.0 | 40.0 | 57.5 |
|  | m04 | 42.5 | 37.5 | 42.5 | 62.5 |
|  | m05 | 50.0 | 52.5 | 45.0 | 50.0 |
|  | m06 | 55.0 | 62.5 | 42.5 | 57.5 |
|  | m07 | 62.5 | 67.5 | 42.5 | 57.5 |
|  | m08 | 77.5 | 75.0 | 45.0 | 67.5 |
|  | m09 | 77.5 | 75.0 | 52.5 | 60.0 |
|  | m10 | 80.0 | 87.5 | 50.0 | 70.0 |
| p002 | m00 | 40.0 | 27.5 | 40.0 | 67.5 |
|  | m01 | 27.5 | 52.5 | 40.0 | 72.5 |
|  | m02 | 57.5 | 52.5 | 40.0 | 80.0 |
|  | m03 | 42.5 | 52.5 | 35.0 | 72.5 |
|  | m04 | 52.5 | 60.0 | 50.0 | 67.5 |
|  | m05 | 50.0 | 57.5 | 42.5 | 72.5 |
|  | m06 | 60.0 | 75.0 | 42.5 | 77.5 |
|  | m07 | 87.5 | 87.5 | 45.0 | 82.5 |
|  | m08 | 80.0 | 67.5 | 47.5 | 85.0 |
|  | m09 | 85.0 | 82.5 | 47.5 | 95.0 |
|  | m10 | 87.5 | 82.5 | 50.0 | 90.0 |
| p003 | m00 | 10.0 | 22.5 | 32.5 | 62.5 |
|  | m01 | 7.5 | 10.0 | 30.0 | 65.0 |
|  | m02 | 15.0 | 22.5 | 35.0 | 52.5 |
|  | m03 | 10.0 | 20.0 | 30.0 | 60.0 |
|  | m04 | 12.5 | 40.0 | 32.5 | 67.5 |
|  | m05 | 35.0 | 57.5 | 42.5 | 62.5 |
|  | m06 | 52.5 | 82.5 | 45.0 | 57.5 |
|  | m07 | 65.0 | 85.0 | 57.5 | 70.0 |
|  | m08 | 90.0 | 92.5 | 47.5 | 72.5 |
|  | m09 | 90.0 | 95.0 | 52.5 | 67.5 |
|  | m10 | 97.5 | 95.0 | 52.5 | 75.0 |
| p004 | m00 | 12.5 | 35.0 | 37.5 | 57.5 |
|  | m01 | 17.5 | 37.5 | 22.5 | 65.0 |
|  | m02 | 17.5 | 47.5 | 37.5 | 60.0 |
|  | m03 | 25.0 | 60.0 | 42.5 | 60.0 |
|  | m04 | 25.0 | 57.5 | 45.0 | 70.0 |
|  | m05 | 35.0 | 55.0 | 47.5 | 75.0 |
|  | m06 | 45.0 | 65.0 | 60.0 | 85.0 |
|  | m07 | 60.0 | 65.0 | 55.0 | 82.5 |
|  | m08 | 42.5 | 77.5 | 62.5 | 85.0 |
|  | m09 | 52.5 | 85.0 | 50.0 | 85.0 |
|  | m10 | 60.0 | 85.0 | 57.5 | 80.0 |
| p005 | m00 | 2.5 | 17.5 | 5.0 | 92.5 |
|  | m01 | 12.5 | 22.5 | 5.0 | 90.0 |
|  | m02 | 17.5 | 20.0 | 7.5 | 90.0 |
|  | m03 | 20.0 | 25.0 | 7.5 | 95.0 |
|  | m04 | 25.0 | 42.5 | 15.0 | 92.5 |
|  | m05 | 35.0 | 55.0 | 12.5 | 95.0 |
|  | m06 | 52.5 | 70.0 | 12.5 | 92.5 |
|  | m07 | 70.0 | 90.0 | 20.0 | 92.5 |
|  | m08 | 82.5 | 92.5 | 22.5 | 97.5 |
|  | m09 | 77.5 | 92.5 | 15.0 | 100.0 |
|  | m10 | 92.5 | 97.5 | 10.0 | 100.0 |
| p006 | m00 | 40.0 | 32.5 | 37.5 | 62.5 |
|  | m01 | 32.5 | 35.0 | 45.0 | 72.5 |
|  | m02 | 42.5 | 37.5 | 47.5 | 60.0 |
|  | m03 | 37.5 | 42.5 | 60.0 | 65.0 |
|  | m04 | 50.0 | 57.5 | 52.5 | 60.0 |
|  | m05 | 50.0 | 60.0 | 40.0 | 77.5 |
|  | m06 | 52.5 | 65.0 | 55.0 | 57.5 |
|  | m07 | 65.0 | 72.5 | 67.5 | 77.5 |
|  | m08 | 75.0 | 67.5 | 50.0 | 67.5 |
|  | m09 | 65.0 | 77.5 | 50.0 | 72.5 |
|  | m10 | 77.5 | 97.5 | 37.5 | 67.5 |
| p007 | m00 | 17.5 | 30.0 | 25.0 | 92.5 |
|  | m01 | 22.5 | 37.5 | 17.5 | 85.0 |
|  | m02 | 22.5 | 32.5 | 30.0 | 82.5 |
|  | m03 | 25.0 | 32.5 | 20.0 | 90.0 |
|  | m04 | 22.5 | 37.5 | 22.5 | 87.5 |
|  | m05 | 30.0 | 45.0 | 25.0 | 85.0 |
|  | m06 | 50.0 | 67.5 | 20.0 | 95.0 |
|  | m07 | 55.0 | 90.0 | 37.5 | 87.5 |
|  | m08 | 72.5 | 80.0 | 30.0 | 92.5 |
|  | m09 | 77.5 | 82.5 | 35.0 | 92.5 |
|  | m10 | 85.0 | 82.5 | 37.5 | 85.0 |
| p008 | m00 | 22.5 | 22.5 | 37.5 | 62.5 |
|  | m01 | 25.0 | 30.0 | 42.5 | 57.5 |
|  | m02 | 27.5 | 35.0 | 30.0 | 70.0 |
|  | m03 | 30.0 | 42.5 | 42.5 | 65.0 |
|  | m04 | 32.5 | 60.0 | 52.5 | 70.0 |
|  | m05 | 40.0 | 65.0 | 45.0 | 77.5 |
|  | m06 | 50.0 | 65.0 | 55.0 | 80.0 |
|  | m07 | 50.0 | 67.5 | 67.5 | 77.5 |
|  | m08 | 60.0 | 75.0 | 62.5 | 92.5 |
|  | m09 | 65.0 | 82.5 | 55.0 | 80.0 |
|  | m10 | 72.5 | 70.0 | 75.0 | 87.5 |
| p009 | m00 | 42.5 | 32.5 | 37.5 | 70.0 |
|  | m01 | 35.0 | 45.0 | 47.5 | 70.0 |
|  | m02 | 40.0 | 40.0 | 52.5 | 75.0 |
|  | m03 | 40.0 | 47.5 | 42.5 | 70.0 |
|  | m04 | 62.5 | 42.5 | 45.0 | 77.5 |
|  | m05 | 40.0 | 50.0 | 47.5 | 62.5 |
|  | m06 | 50.0 | 60.0 | 50.0 | 65.0 |
|  | m07 | 62.5 | 67.5 | 45.0 | 62.5 |
|  | m08 | 70.0 | 87.5 | 50.0 | 65.0 |
|  | m09 | 75.0 | 77.5 | 45.0 | 77.5 |
|  | m10 | 82.5 | 77.5 | 47.5 | 65.0 |
| p010 | m00 | 7.5 | 32.5 | 17.5 | 55.0 |
|  | m01 | 15.0 | 10.0 | 12.5 | 67.5 |
|  | m02 | 22.5 | 27.5 | 12.5 | 57.5 |
|  | m03 | 22.5 | 30.0 | 20.0 | 70.0 |
|  | m04 | 25.0 | 35.0 | 15.0 | 57.5 |
|  | m05 | 50.0 | 52.5 | 22.5 | 75.0 |
|  | m06 | 55.0 | 70.0 | 25.0 | 72.5 |
|  | m07 | 52.5 | 77.5 | 32.5 | 80.0 |
|  | m08 | 82.5 | 82.5 | 20.0 | 72.5 |
|  | m09 | 82.5 | 85.0 | 30.0 | 80.0 |
|  | m10 | 87.5 | 95.0 | 42.5 | 90.0 |
| p011 | m00 | 15.0 | 7.5 | 30.0 | 47.5 |
|  | m01 | 7.5 | 17.5 | 37.5 | 37.5 |
|  | m02 | 10.0 | 20.0 | 30.0 | 45.0 |
|  | m03 | 20.0 | 20.0 | 42.5 | 35.0 |
|  | m04 | 30.0 | 30.0 | 50.0 | 60.0 |
|  | m05 | 37.5 | 40.0 | 60.0 | 60.0 |
|  | m06 | 55.0 | 60.0 | 65.0 | 72.5 |
|  | m07 | 80.0 | 72.5 | 70.0 | 82.5 |
|  | m08 | 70.0 | 82.5 | 75.0 | 82.5 |
|  | m09 | 82.5 | 90.0 | 85.0 | 82.5 |
|  | m10 | 92.5 | 95.0 | 82.5 | 80.0 |
| p012 | m00 | 7.5 | 22.5 | 12.5 | 57.5 |
|  | m01 | 12.5 | 22.5 | 15.0 | 55.0 |
|  | m02 | 10.0 | 30.0 | 15.0 | 47.5 |
|  | m03 | 17.5 | 27.5 | 7.5 | 55.0 |
|  | m04 | 22.5 | 42.5 | 15.0 | 65.0 |
|  | m05 | 40.0 | 47.5 | 20.0 | 60.0 |
|  | m06 | 50.0 | 65.0 | 22.5 | 77.5 |
|  | m07 | 62.5 | 67.5 | 40.0 | 77.5 |
|  | m08 | 60.0 | 92.5 | 35.0 | 82.5 |
|  | m09 | 85.0 | 92.5 | 40.0 | 92.5 |
|  | m10 | 92.5 | 95.0 | 42.5 | 82.5 |
| p013 | m00 | 12.5 | 32.5 | 25.0 | 52.5 |
|  | m01 | 17.5 | 27.5 | 32.5 | 50.0 |
|  | m02 | 12.5 | 35.0 | 30.0 | 37.5 |
|  | m03 | 10.0 | 30.0 | 22.5 | 42.5 |
|  | m04 | 22.5 | 37.5 | 22.5 | 52.5 |
|  | m05 | 30.0 | 47.5 | 27.5 | 47.5 |
|  | m06 | 22.5 | 32.5 | 32.5 | 47.5 |
|  | m07 | 40.0 | 57.5 | 32.5 | 57.5 |
|  | m08 | 45.0 | 55.0 | 27.5 | 52.5 |
|  | m09 | 47.5 | 62.5 | 35.0 | 45.0 |
|  | m10 | 62.5 | 62.5 | 40.0 | 62.5 |
| p014 | m00 | 30.0 | 90.0 | 17.5 | 27.5 |
|  | m01 | 32.5 | 92.5 | 27.5 | 37.5 |
|  | m02 | 40.0 | 95.0 | 25.0 | 42.5 |
|  | m03 | 30.0 | 95.0 | 27.5 | 37.5 |
|  | m04 | 35.0 | 95.0 | 37.5 | 60.0 |
|  | m05 | 30.0 | 92.5 | 50.0 | 75.0 |
|  | m06 | 30.0 | 97.5 | 62.5 | 80.0 |
|  | m07 | 32.5 | 95.0 | 75.0 | 95.0 |
|  | m08 | 30.0 | 95.0 | 82.5 | 92.5 |
|  | m09 | 35.0 | 92.5 | 82.5 | 90.0 |
|  | m10 | 32.5 | 92.5 | 75.0 | 97.5 |
| p015 | m00 | 5.0 | 12.5 | 45.0 | 72.5 |
|  | m01 | 12.5 | 20.0 | 47.5 | 47.5 |
|  | m02 | 12.5 | 20.0 | 32.5 | 50.0 |
|  | m03 | 20.0 | 30.0 | 42.5 | 55.0 |
|  | m04 | 25.0 | 32.5 | 35.0 | 42.5 |
|  | m05 | 40.0 | 47.5 | 40.0 | 57.5 |
|  | m06 | 50.0 | 50.0 | 47.5 | 57.5 |
|  | m07 | 72.5 | 67.5 | 45.0 | 42.5 |
|  | m08 | 67.5 | 85.0 | 45.0 | 62.5 |
|  | m09 | 82.5 | 80.0 | 47.5 | 52.5 |
|  | m10 | 85.0 | 87.5 | 42.5 | 50.0 |
| p016 | m00 | 7.5 | 17.5 | 17.5 | 47.5 |
|  | m01 | 15.0 | 20.0 | 27.5 | 60.0 |
|  | m02 | 7.5 | 25.0 | 15.0 | 67.5 |
|  | m03 | 17.5 | 27.5 | 30.0 | 52.5 |
|  | m04 | 20.0 | 37.5 | 27.5 | 70.0 |
|  | m05 | 35.0 | 45.0 | 37.5 | 72.5 |
|  | m06 | 45.0 | 55.0 | 40.0 | 80.0 |
|  | m07 | 57.5 | 62.5 | 60.0 | 80.0 |
|  | m08 | 67.5 | 77.5 | 55.0 | 82.5 |
|  | m09 | 77.5 | 75.0 | 52.5 | 80.0 |
|  | m10 | 95.0 | 92.5 | 67.5 | 77.5 |
| p017 | m00 | 27.5 | 30.0 | 10.0 | 35.0 |
|  | m01 | 25.0 | 40.0 | 22.5 | 40.0 |
|  | m02 | 22.5 | 30.0 | 27.5 | 35.0 |
|  | m03 | 50.0 | 37.5 | 20.0 | 45.0 |
|  | m04 | 45.0 | 47.5 | 35.0 | 35.0 |
|  | m05 | 40.0 | 55.0 | 37.5 | 40.0 |
|  | m06 | 55.0 | 65.0 | 27.5 | 45.0 |
|  | m07 | 72.5 | 70.0 | 30.0 | 42.5 |
|  | m08 | 65.0 | 72.5 | 30.0 | 37.5 |
|  | m09 | 85.0 | 87.5 | 32.5 | 42.5 |
|  | m10 | 85.0 | 90.0 | 47.5 | 42.5 |
| p018 | m00 | 15.0 | 25.0 | 40.0 | 77.5 |
|  | m01 | 12.5 | 30.0 | 47.5 | 80.0 |
|  | m02 | 15.0 | 30.0 | 55.0 | 82.5 |
|  | m03 | 27.5 | 30.0 | 40.0 | 82.5 |
|  | m04 | 32.5 | 40.0 | 62.5 | 80.0 |
|  | m05 | 47.5 | 47.5 | 50.0 | 80.0 |
|  | m06 | 47.5 | 65.0 | 47.5 | 80.0 |
|  | m07 | 75.0 | 77.5 | 47.5 | 90.0 |
|  | m08 | 75.0 | 87.5 | 50.0 | 87.5 |
|  | m09 | 87.5 | 95.0 | 55.0 | 92.5 |
|  | m10 | 90.0 | 92.5 | 67.5 | 82.5 |
| p019 | m00 | 25.0 | 30.0 | 25.0 | 65.0 |
|  | m01 | 32.5 | 40.0 | 22.5 | 67.5 |
|  | m02 | 30.0 | 30.0 | 22.5 | 62.5 |
|  | m03 | 37.5 | 30.0 | 25.0 | 65.0 |
|  | m04 | 35.0 | 42.5 | 17.5 | 75.0 |
|  | m05 | 40.0 | 65.0 | 35.0 | 72.5 |
|  | m06 | 50.0 | 62.5 | 30.0 | 80.0 |
|  | m07 | 72.5 | 60.0 | 37.5 | 72.5 |
|  | m08 | 72.5 | 87.5 | 32.5 | 77.5 |
|  | m09 | 77.5 | 90.0 | 37.5 | 82.5 |
|  | m10 | 87.5 | 90.0 | 32.5 | 92.5 |
| p020 | m00 | 17.5 | 55.0 | 15.0 | 25.0 |
|  | m01 | 15.0 | 55.0 | 20.0 | 27.5 |
|  | m02 | 20.0 | 47.5 | 25.0 | 20.0 |
|  | m03 | 22.5 | 62.5 | 25.0 | 25.0 |
|  | m04 | 25.0 | 60.0 | 35.0 | 32.5 |
|  | m05 | 32.5 | 65.0 | 55.0 | 47.5 |
|  | m06 | 40.0 | 87.5 | 57.5 | 70.0 |
|  | m07 | 50.0 | 90.0 | 75.0 | 75.0 |
|  | m08 | 50.0 | 80.0 | 82.5 | 82.5 |
|  | m09 | 55.0 | 85.0 | 82.5 | 82.5 |
|  | m10 | 60.0 | 92.5 | 92.5 | 87.5 |
| p021 | m00 | 10.0 | 12.5 | 2.5 | 40.0 |
|  | m01 | 7.5 | 5.0 | 10.0 | 40.0 |
|  | m02 | 15.0 | 12.5 | 5.0 | 37.5 |
|  | m03 | 15.0 | 27.5 | 10.0 | 45.0 |
|  | m04 | 25.0 | 25.0 | 10.0 | 42.5 |
|  | m05 | 37.5 | 45.0 | 22.5 | 50.0 |
|  | m06 | 50.0 | 65.0 | 27.5 | 52.5 |
|  | m07 | 70.0 | 75.0 | 27.5 | 57.5 |
|  | m08 | 80.0 | 85.0 | 22.5 | 60.0 |
|  | m09 | 92.5 | 90.0 | 32.5 | 60.0 |
|  | m10 | 85.0 | 90.0 | 32.5 | 55.0 |
| p022 | m00 | 22.5 | 30.0 | 55.0 | 77.5 |
|  | m01 | 27.5 | 32.5 | 65.0 | 72.5 |
|  | m02 | 30.0 | 40.0 | 72.5 | 72.5 |
|  | m03 | 42.5 | 52.5 | 62.5 | 70.0 |
|  | m04 | 47.5 | 52.5 | 67.5 | 85.0 |
|  | m05 | 47.5 | 52.5 | 62.5 | 80.0 |
|  | m06 | 65.0 | 52.5 | 65.0 | 72.5 |
|  | m07 | 72.5 | 75.0 | 55.0 | 75.0 |
|  | m08 | 77.5 | 77.5 | 75.0 | 67.5 |
|  | m09 | 87.5 | 85.0 | 62.5 | 77.5 |
|  | m10 | 87.5 | 92.5 | 70.0 | 72.5 |
| p023 | m00 | 15.0 | 25.0 | 32.5 | 50.0 |
|  | m01 | 20.0 | 20.0 | 27.5 | 47.5 |
|  | m02 | 20.0 | 22.5 | 42.5 | 47.5 |
|  | m03 | 20.0 | 35.0 | 27.5 | 42.5 |
|  | m04 | 30.0 | 42.5 | 42.5 | 50.0 |
|  | m05 | 45.0 | 40.0 | 57.5 | 70.0 |
|  | m06 | 60.0 | 72.5 | 42.5 | 75.0 |
|  | m07 | 85.0 | 82.5 | 60.0 | 87.5 |
|  | m08 | 80.0 | 87.5 | 62.5 | 90.0 |
|  | m09 | 82.5 | 87.5 | 80.0 | 75.0 |
|  | m10 | 80.0 | 92.5 | 75.0 | 85.0 |
| p024 | m00 | 2.5 | 15.0 | 5.0 | 22.5 |
|  | m01 | 10.0 | 25.0 | 20.0 | 37.5 |
|  | m02 | 20.0 | 25.0 | 15.0 | 40.0 |
|  | m03 | 20.0 | 30.0 | 10.0 | 35.0 |
|  | m04 | 27.5 | 40.0 | 25.0 | 47.5 |
|  | m05 | 45.0 | 55.0 | 27.5 | 55.0 |
|  | m06 | 50.0 | 62.5 | 25.0 | 50.0 |
|  | m07 | 72.5 | 77.5 | 30.0 | 57.5 |
|  | m08 | 82.5 | 80.0 | 30.0 | 67.5 |
|  | m09 | 90.0 | 95.0 | 32.5 | 57.5 |
|  | m10 | 92.5 | 90.0 | 30.0 | 75.0 |
| p025 | m00 | 50.0 | 60.0 | 50.0 | 67.5 |
|  | m01 | 35.0 | 60.0 | 50.0 | 67.5 |
|  | m02 | 40.0 | 62.5 | 45.0 | 62.5 |
|  | m03 | 40.0 | 60.0 | 60.0 | 70.0 |
|  | m04 | 50.0 | 75.0 | 55.0 | 72.5 |
|  | m05 | 55.0 | 67.5 | 65.0 | 82.5 |
|  | m06 | 55.0 | 67.5 | 60.0 | 90.0 |
|  | m07 | 65.0 | 82.5 | 67.5 | 85.0 |
|  | m08 | 75.0 | 87.5 | 77.5 | 82.5 |
|  | m09 | 75.0 | 90.0 | 75.0 | 82.5 |
|  | m10 | 87.5 | 97.5 | 67.5 | 90.0 |
| p026 | m00 | 10.0 | 32.5 | 27.5 | 57.5 |
|  | m01 | 12.5 | 32.5 | 32.5 | 62.5 |
|  | m02 | 15.0 | 32.5 | 22.5 | 70.0 |
|  | m03 | 12.5 | 35.0 | 25.0 | 80.0 |
|  | m04 | 22.5 | 45.0 | 45.0 | 95.0 |
|  | m05 | 40.0 | 60.0 | 35.0 | 82.5 |
|  | m06 | 45.0 | 65.0 | 45.0 | 95.0 |
|  | m07 | 70.0 | 90.0 | 45.0 | 90.0 |
|  | m08 | 72.5 | 92.5 | 52.5 | 92.5 |
|  | m09 | 90.0 | 100.0 | 55.0 | 90.0 |
|  | m10 | 92.5 | 100.0 | 67.5 | 100.0 |
| p027 | m00 | 30.0 | 30.0 | 40.0 | 65.0 |
|  | m01 | 27.5 | 40.0 | 42.5 | 60.0 |
|  | m02 | 12.5 | 30.0 | 47.5 | 52.5 |
|  | m03 | 25.0 | 32.5 | 37.5 | 57.5 |
|  | m04 | 37.5 | 47.5 | 45.0 | 75.0 |
|  | m05 | 40.0 | 30.0 | 60.0 | 62.5 |
|  | m06 | 52.5 | 55.0 | 47.5 | 62.5 |
|  | m07 | 37.5 | 62.5 | 62.5 | 62.5 |
|  | m08 | 55.0 | 62.5 | 55.0 | 52.5 |
|  | m09 | 67.5 | 65.0 | 55.0 | 65.0 |
|  | m10 | 67.5 | 65.0 | 60.0 | 60.0 |
| p028 | m00 | 10.0 | 20.0 | 27.5 | 47.5 |
|  | m01 | 10.0 | 20.0 | 35.0 | 52.5 |
|  | m02 | 17.5 | 20.0 | 32.5 | 70.0 |
|  | m03 | 27.5 | 27.5 | 30.0 | 75.0 |
|  | m04 | 40.0 | 42.5 | 45.0 | 82.5 |
|  | m05 | 57.5 | 75.0 | 50.0 | 80.0 |
|  | m06 | 77.5 | 87.5 | 60.0 | 95.0 |
|  | m07 | 97.5 | 100.0 | 70.0 | 92.5 |
|  | m08 | 97.5 | 100.0 | 75.0 | 95.0 |
|  | m09 | 100.0 | 100.0 | 75.0 | 95.0 |
|  | m10 | 100.0 | 100.0 | 80.0 | 97.5 |
| p029 | m00 | 10.0 | 22.5 | 32.5 | 60.0 |
|  | m01 | 12.5 | 17.5 | 37.5 | 85.0 |
|  | m02 | 17.5 | 20.0 | 40.0 | 75.0 |
|  | m03 | 32.5 | 20.0 | 50.0 | 70.0 |
|  | m04 | 20.0 | 27.5 | 40.0 | 75.0 |
|  | m05 | 50.0 | 52.5 | 42.5 | 82.5 |
|  | m06 | 55.0 | 62.5 | 52.5 | 90.0 |
|  | m07 | 65.0 | 90.0 | 45.0 | 82.5 |
|  | m08 | 90.0 | 90.0 | 55.0 | 77.5 |
|  | m09 | 92.5 | 92.5 | 55.0 | 82.5 |
|  | m10 | 95.0 | 92.5 | 50.0 | 87.5 |
| p030 | m00 | 10.0 | 15.0 | 20.0 | 50.0 |
|  | m01 | 2.5 | 15.0 | 25.0 | 47.5 |
|  | m02 | 12.5 | 15.0 | 30.0 | 60.0 |
|  | m03 | 7.5 | 25.0 | 27.5 | 52.5 |
|  | m04 | 17.5 | 30.0 | 20.0 | 65.0 |
|  | m05 | 35.0 | 55.0 | 40.0 | 57.5 |
|  | m06 | 47.5 | 60.0 | 42.5 | 65.0 |
|  | m07 | 62.5 | 72.5 | 42.5 | 65.0 |
|  | m08 | 77.5 | 90.0 | 55.0 | 77.5 |
|  | m09 | 85.0 | 90.0 | 52.5 | 75.0 |
|  | m10 | 92.5 | 97.5 | 60.0 | 77.5 |

## Transform Table for within-subject analysis¶

In [16]:

```
avg_between_df = avg_df.unstack("grade")
pt1_between_df = pt1_df.unstack("grade")
pt2_between_df = pt2_df.unstack("grade")
avg_between_df
```

Out[16]:

|  | fearLOW | | | | | | | | | | | angerLOW | | | | | | | | | | | fearUP | | | | | | | | | | | angerUP | | | | | | | | | | |
| --- | --- | --- | --- | --- | --- | --- | --- | --- | --- | --- | --- | --- | --- | --- | --- | --- | --- | --- | --- | --- | --- | --- | --- | --- | --- | --- | --- | --- | --- | --- | --- | --- | --- | --- | --- | --- | --- | --- | --- | --- | --- | --- | --- | --- |
| grade | m00 | m01 | m02 | m03 | m04 | m05 | m06 | m07 | m08 | m09 | m10 | m00 | m01 | m02 | m03 | m04 | m05 | m06 | m07 | m08 | m09 | m10 | m00 | m01 | m02 | m03 | m04 | m05 | m06 | m07 | m08 | m09 | m10 | m00 | m01 | m02 | m03 | m04 | m05 | m06 | m07 | m08 | m09 | m10 |
| p |  |  |  |  |  |  |  |  |  |  |  |  |  |  |  |  |  |  |  |  |  |  |  |  |  |  |  |  |  |  |  |  |  |  |  |  |  |  |  |  |  |  |  |  |
| p001 | 15.0 | 25.0 | 42.5 | 40.0 | 42.5 | 50.0 | 55.0 | 62.5 | 77.5 | 77.5 | 80.0 | 25.0 | 32.5 | 32.5 | 45.0 | 37.5 | 52.5 | 62.5 | 67.5 | 75.0 | 75.0 | 87.5 | 35.0 | 32.5 | 42.5 | 40.0 | 42.5 | 45.0 | 42.5 | 42.5 | 45.0 | 52.5 | 50.0 | 57.5 | 42.5 | 55.0 | 57.5 | 62.5 | 50.0 | 57.5 | 57.5 | 67.5 | 60.0 | 70.0 |
| p002 | 40.0 | 27.5 | 57.5 | 42.5 | 52.5 | 50.0 | 60.0 | 87.5 | 80.0 | 85.0 | 87.5 | 27.5 | 52.5 | 52.5 | 52.5 | 60.0 | 57.5 | 75.0 | 87.5 | 67.5 | 82.5 | 82.5 | 40.0 | 40.0 | 40.0 | 35.0 | 50.0 | 42.5 | 42.5 | 45.0 | 47.5 | 47.5 | 50.0 | 67.5 | 72.5 | 80.0 | 72.5 | 67.5 | 72.5 | 77.5 | 82.5 | 85.0 | 95.0 | 90.0 |
| p003 | 10.0 | 7.5 | 15.0 | 10.0 | 12.5 | 35.0 | 52.5 | 65.0 | 90.0 | 90.0 | 97.5 | 22.5 | 10.0 | 22.5 | 20.0 | 40.0 | 57.5 | 82.5 | 85.0 | 92.5 | 95.0 | 95.0 | 32.5 | 30.0 | 35.0 | 30.0 | 32.5 | 42.5 | 45.0 | 57.5 | 47.5 | 52.5 | 52.5 | 62.5 | 65.0 | 52.5 | 60.0 | 67.5 | 62.5 | 57.5 | 70.0 | 72.5 | 67.5 | 75.0 |
| p004 | 12.5 | 17.5 | 17.5 | 25.0 | 25.0 | 35.0 | 45.0 | 60.0 | 42.5 | 52.5 | 60.0 | 35.0 | 37.5 | 47.5 | 60.0 | 57.5 | 55.0 | 65.0 | 65.0 | 77.5 | 85.0 | 85.0 | 37.5 | 22.5 | 37.5 | 42.5 | 45.0 | 47.5 | 60.0 | 55.0 | 62.5 | 50.0 | 57.5 | 57.5 | 65.0 | 60.0 | 60.0 | 70.0 | 75.0 | 85.0 | 82.5 | 85.0 | 85.0 | 80.0 |
| p005 | 2.5 | 12.5 | 17.5 | 20.0 | 25.0 | 35.0 | 52.5 | 70.0 | 82.5 | 77.5 | 92.5 | 17.5 | 22.5 | 20.0 | 25.0 | 42.5 | 55.0 | 70.0 | 90.0 | 92.5 | 92.5 | 97.5 | 5.0 | 5.0 | 7.5 | 7.5 | 15.0 | 12.5 | 12.5 | 20.0 | 22.5 | 15.0 | 10.0 | 92.5 | 90.0 | 90.0 | 95.0 | 92.5 | 95.0 | 92.5 | 92.5 | 97.5 | 100.0 | 100.0 |
| p006 | 40.0 | 32.5 | 42.5 | 37.5 | 50.0 | 50.0 | 52.5 | 65.0 | 75.0 | 65.0 | 77.5 | 32.5 | 35.0 | 37.5 | 42.5 | 57.5 | 60.0 | 65.0 | 72.5 | 67.5 | 77.5 | 97.5 | 37.5 | 45.0 | 47.5 | 60.0 | 52.5 | 40.0 | 55.0 | 67.5 | 50.0 | 50.0 | 37.5 | 62.5 | 72.5 | 60.0 | 65.0 | 60.0 | 77.5 | 57.5 | 77.5 | 67.5 | 72.5 | 67.5 |
| p007 | 17.5 | 22.5 | 22.5 | 25.0 | 22.5 | 30.0 | 50.0 | 55.0 | 72.5 | 77.5 | 85.0 | 30.0 | 37.5 | 32.5 | 32.5 | 37.5 | 45.0 | 67.5 | 90.0 | 80.0 | 82.5 | 82.5 | 25.0 | 17.5 | 30.0 | 20.0 | 22.5 | 25.0 | 20.0 | 37.5 | 30.0 | 35.0 | 37.5 | 92.5 | 85.0 | 82.5 | 90.0 | 87.5 | 85.0 | 95.0 | 87.5 | 92.5 | 92.5 | 85.0 |
| p008 | 22.5 | 25.0 | 27.5 | 30.0 | 32.5 | 40.0 | 50.0 | 50.0 | 60.0 | 65.0 | 72.5 | 22.5 | 30.0 | 35.0 | 42.5 | 60.0 | 65.0 | 65.0 | 67.5 | 75.0 | 82.5 | 70.0 | 37.5 | 42.5 | 30.0 | 42.5 | 52.5 | 45.0 | 55.0 | 67.5 | 62.5 | 55.0 | 75.0 | 62.5 | 57.5 | 70.0 | 65.0 | 70.0 | 77.5 | 80.0 | 77.5 | 92.5 | 80.0 | 87.5 |
| p009 | 42.5 | 35.0 | 40.0 | 40.0 | 62.5 | 40.0 | 50.0 | 62.5 | 70.0 | 75.0 | 82.5 | 32.5 | 45.0 | 40.0 | 47.5 | 42.5 | 50.0 | 60.0 | 67.5 | 87.5 | 77.5 | 77.5 | 37.5 | 47.5 | 52.5 | 42.5 | 45.0 | 47.5 | 50.0 | 45.0 | 50.0 | 45.0 | 47.5 | 70.0 | 70.0 | 75.0 | 70.0 | 77.5 | 62.5 | 65.0 | 62.5 | 65.0 | 77.5 | 65.0 |
| p010 | 7.5 | 15.0 | 22.5 | 22.5 | 25.0 | 50.0 | 55.0 | 52.5 | 82.5 | 82.5 | 87.5 | 32.5 | 10.0 | 27.5 | 30.0 | 35.0 | 52.5 | 70.0 | 77.5 | 82.5 | 85.0 | 95.0 | 17.5 | 12.5 | 12.5 | 20.0 | 15.0 | 22.5 | 25.0 | 32.5 | 20.0 | 30.0 | 42.5 | 55.0 | 67.5 | 57.5 | 70.0 | 57.5 | 75.0 | 72.5 | 80.0 | 72.5 | 80.0 | 90.0 |
| p011 | 15.0 | 7.5 | 10.0 | 20.0 | 30.0 | 37.5 | 55.0 | 80.0 | 70.0 | 82.5 | 92.5 | 7.5 | 17.5 | 20.0 | 20.0 | 30.0 | 40.0 | 60.0 | 72.5 | 82.5 | 90.0 | 95.0 | 30.0 | 37.5 | 30.0 | 42.5 | 50.0 | 60.0 | 65.0 | 70.0 | 75.0 | 85.0 | 82.5 | 47.5 | 37.5 | 45.0 | 35.0 | 60.0 | 60.0 | 72.5 | 82.5 | 82.5 | 82.5 | 80.0 |
| p012 | 7.5 | 12.5 | 10.0 | 17.5 | 22.5 | 40.0 | 50.0 | 62.5 | 60.0 | 85.0 | 92.5 | 22.5 | 22.5 | 30.0 | 27.5 | 42.5 | 47.5 | 65.0 | 67.5 | 92.5 | 92.5 | 95.0 | 12.5 | 15.0 | 15.0 | 7.5 | 15.0 | 20.0 | 22.5 | 40.0 | 35.0 | 40.0 | 42.5 | 57.5 | 55.0 | 47.5 | 55.0 | 65.0 | 60.0 | 77.5 | 77.5 | 82.5 | 92.5 | 82.5 |
| p013 | 12.5 | 17.5 | 12.5 | 10.0 | 22.5 | 30.0 | 22.5 | 40.0 | 45.0 | 47.5 | 62.5 | 32.5 | 27.5 | 35.0 | 30.0 | 37.5 | 47.5 | 32.5 | 57.5 | 55.0 | 62.5 | 62.5 | 25.0 | 32.5 | 30.0 | 22.5 | 22.5 | 27.5 | 32.5 | 32.5 | 27.5 | 35.0 | 40.0 | 52.5 | 50.0 | 37.5 | 42.5 | 52.5 | 47.5 | 47.5 | 57.5 | 52.5 | 45.0 | 62.5 |
| p014 | 30.0 | 32.5 | 40.0 | 30.0 | 35.0 | 30.0 | 30.0 | 32.5 | 30.0 | 35.0 | 32.5 | 90.0 | 92.5 | 95.0 | 95.0 | 95.0 | 92.5 | 97.5 | 95.0 | 95.0 | 92.5 | 92.5 | 17.5 | 27.5 | 25.0 | 27.5 | 37.5 | 50.0 | 62.5 | 75.0 | 82.5 | 82.5 | 75.0 | 27.5 | 37.5 | 42.5 | 37.5 | 60.0 | 75.0 | 80.0 | 95.0 | 92.5 | 90.0 | 97.5 |
| p015 | 5.0 | 12.5 | 12.5 | 20.0 | 25.0 | 40.0 | 50.0 | 72.5 | 67.5 | 82.5 | 85.0 | 12.5 | 20.0 | 20.0 | 30.0 | 32.5 | 47.5 | 50.0 | 67.5 | 85.0 | 80.0 | 87.5 | 45.0 | 47.5 | 32.5 | 42.5 | 35.0 | 40.0 | 47.5 | 45.0 | 45.0 | 47.5 | 42.5 | 72.5 | 47.5 | 50.0 | 55.0 | 42.5 | 57.5 | 57.5 | 42.5 | 62.5 | 52.5 | 50.0 |
| p016 | 7.5 | 15.0 | 7.5 | 17.5 | 20.0 | 35.0 | 45.0 | 57.5 | 67.5 | 77.5 | 95.0 | 17.5 | 20.0 | 25.0 | 27.5 | 37.5 | 45.0 | 55.0 | 62.5 | 77.5 | 75.0 | 92.5 | 17.5 | 27.5 | 15.0 | 30.0 | 27.5 | 37.5 | 40.0 | 60.0 | 55.0 | 52.5 | 67.5 | 47.5 | 60.0 | 67.5 | 52.5 | 70.0 | 72.5 | 80.0 | 80.0 | 82.5 | 80.0 | 77.5 |
| p017 | 27.5 | 25.0 | 22.5 | 50.0 | 45.0 | 40.0 | 55.0 | 72.5 | 65.0 | 85.0 | 85.0 | 30.0 | 40.0 | 30.0 | 37.5 | 47.5 | 55.0 | 65.0 | 70.0 | 72.5 | 87.5 | 90.0 | 10.0 | 22.5 | 27.5 | 20.0 | 35.0 | 37.5 | 27.5 | 30.0 | 30.0 | 32.5 | 47.5 | 35.0 | 40.0 | 35.0 | 45.0 | 35.0 | 40.0 | 45.0 | 42.5 | 37.5 | 42.5 | 42.5 |
| p018 | 15.0 | 12.5 | 15.0 | 27.5 | 32.5 | 47.5 | 47.5 | 75.0 | 75.0 | 87.5 | 90.0 | 25.0 | 30.0 | 30.0 | 30.0 | 40.0 | 47.5 | 65.0 | 77.5 | 87.5 | 95.0 | 92.5 | 40.0 | 47.5 | 55.0 | 40.0 | 62.5 | 50.0 | 47.5 | 47.5 | 50.0 | 55.0 | 67.5 | 77.5 | 80.0 | 82.5 | 82.5 | 80.0 | 80.0 | 80.0 | 90.0 | 87.5 | 92.5 | 82.5 |
| p019 | 25.0 | 32.5 | 30.0 | 37.5 | 35.0 | 40.0 | 50.0 | 72.5 | 72.5 | 77.5 | 87.5 | 30.0 | 40.0 | 30.0 | 30.0 | 42.5 | 65.0 | 62.5 | 60.0 | 87.5 | 90.0 | 90.0 | 25.0 | 22.5 | 22.5 | 25.0 | 17.5 | 35.0 | 30.0 | 37.5 | 32.5 | 37.5 | 32.5 | 65.0 | 67.5 | 62.5 | 65.0 | 75.0 | 72.5 | 80.0 | 72.5 | 77.5 | 82.5 | 92.5 |
| p020 | 17.5 | 15.0 | 20.0 | 22.5 | 25.0 | 32.5 | 40.0 | 50.0 | 50.0 | 55.0 | 60.0 | 55.0 | 55.0 | 47.5 | 62.5 | 60.0 | 65.0 | 87.5 | 90.0 | 80.0 | 85.0 | 92.5 | 15.0 | 20.0 | 25.0 | 25.0 | 35.0 | 55.0 | 57.5 | 75.0 | 82.5 | 82.5 | 92.5 | 25.0 | 27.5 | 20.0 | 25.0 | 32.5 | 47.5 | 70.0 | 75.0 | 82.5 | 82.5 | 87.5 |
| p021 | 10.0 | 7.5 | 15.0 | 15.0 | 25.0 | 37.5 | 50.0 | 70.0 | 80.0 | 92.5 | 85.0 | 12.5 | 5.0 | 12.5 | 27.5 | 25.0 | 45.0 | 65.0 | 75.0 | 85.0 | 90.0 | 90.0 | 2.5 | 10.0 | 5.0 | 10.0 | 10.0 | 22.5 | 27.5 | 27.5 | 22.5 | 32.5 | 32.5 | 40.0 | 40.0 | 37.5 | 45.0 | 42.5 | 50.0 | 52.5 | 57.5 | 60.0 | 60.0 | 55.0 |
| p022 | 22.5 | 27.5 | 30.0 | 42.5 | 47.5 | 47.5 | 65.0 | 72.5 | 77.5 | 87.5 | 87.5 | 30.0 | 32.5 | 40.0 | 52.5 | 52.5 | 52.5 | 52.5 | 75.0 | 77.5 | 85.0 | 92.5 | 55.0 | 65.0 | 72.5 | 62.5 | 67.5 | 62.5 | 65.0 | 55.0 | 75.0 | 62.5 | 70.0 | 77.5 | 72.5 | 72.5 | 70.0 | 85.0 | 80.0 | 72.5 | 75.0 | 67.5 | 77.5 | 72.5 |
| p023 | 15.0 | 20.0 | 20.0 | 20.0 | 30.0 | 45.0 | 60.0 | 85.0 | 80.0 | 82.5 | 80.0 | 25.0 | 20.0 | 22.5 | 35.0 | 42.5 | 40.0 | 72.5 | 82.5 | 87.5 | 87.5 | 92.5 | 32.5 | 27.5 | 42.5 | 27.5 | 42.5 | 57.5 | 42.5 | 60.0 | 62.5 | 80.0 | 75.0 | 50.0 | 47.5 | 47.5 | 42.5 | 50.0 | 70.0 | 75.0 | 87.5 | 90.0 | 75.0 | 85.0 |
| p024 | 2.5 | 10.0 | 20.0 | 20.0 | 27.5 | 45.0 | 50.0 | 72.5 | 82.5 | 90.0 | 92.5 | 15.0 | 25.0 | 25.0 | 30.0 | 40.0 | 55.0 | 62.5 | 77.5 | 80.0 | 95.0 | 90.0 | 5.0 | 20.0 | 15.0 | 10.0 | 25.0 | 27.5 | 25.0 | 30.0 | 30.0 | 32.5 | 30.0 | 22.5 | 37.5 | 40.0 | 35.0 | 47.5 | 55.0 | 50.0 | 57.5 | 67.5 | 57.5 | 75.0 |
| p025 | 50.0 | 35.0 | 40.0 | 40.0 | 50.0 | 55.0 | 55.0 | 65.0 | 75.0 | 75.0 | 87.5 | 60.0 | 60.0 | 62.5 | 60.0 | 75.0 | 67.5 | 67.5 | 82.5 | 87.5 | 90.0 | 97.5 | 50.0 | 50.0 | 45.0 | 60.0 | 55.0 | 65.0 | 60.0 | 67.5 | 77.5 | 75.0 | 67.5 | 67.5 | 67.5 | 62.5 | 70.0 | 72.5 | 82.5 | 90.0 | 85.0 | 82.5 | 82.5 | 90.0 |
| p026 | 10.0 | 12.5 | 15.0 | 12.5 | 22.5 | 40.0 | 45.0 | 70.0 | 72.5 | 90.0 | 92.5 | 32.5 | 32.5 | 32.5 | 35.0 | 45.0 | 60.0 | 65.0 | 90.0 | 92.5 | 100.0 | 100.0 | 27.5 | 32.5 | 22.5 | 25.0 | 45.0 | 35.0 | 45.0 | 45.0 | 52.5 | 55.0 | 67.5 | 57.5 | 62.5 | 70.0 | 80.0 | 95.0 | 82.5 | 95.0 | 90.0 | 92.5 | 90.0 | 100.0 |
| p027 | 30.0 | 27.5 | 12.5 | 25.0 | 37.5 | 40.0 | 52.5 | 37.5 | 55.0 | 67.5 | 67.5 | 30.0 | 40.0 | 30.0 | 32.5 | 47.5 | 30.0 | 55.0 | 62.5 | 62.5 | 65.0 | 65.0 | 40.0 | 42.5 | 47.5 | 37.5 | 45.0 | 60.0 | 47.5 | 62.5 | 55.0 | 55.0 | 60.0 | 65.0 | 60.0 | 52.5 | 57.5 | 75.0 | 62.5 | 62.5 | 62.5 | 52.5 | 65.0 | 60.0 |
| p028 | 10.0 | 10.0 | 17.5 | 27.5 | 40.0 | 57.5 | 77.5 | 97.5 | 97.5 | 100.0 | 100.0 | 20.0 | 20.0 | 20.0 | 27.5 | 42.5 | 75.0 | 87.5 | 100.0 | 100.0 | 100.0 | 100.0 | 27.5 | 35.0 | 32.5 | 30.0 | 45.0 | 50.0 | 60.0 | 70.0 | 75.0 | 75.0 | 80.0 | 47.5 | 52.5 | 70.0 | 75.0 | 82.5 | 80.0 | 95.0 | 92.5 | 95.0 | 95.0 | 97.5 |
| p029 | 10.0 | 12.5 | 17.5 | 32.5 | 20.0 | 50.0 | 55.0 | 65.0 | 90.0 | 92.5 | 95.0 | 22.5 | 17.5 | 20.0 | 20.0 | 27.5 | 52.5 | 62.5 | 90.0 | 90.0 | 92.5 | 92.5 | 32.5 | 37.5 | 40.0 | 50.0 | 40.0 | 42.5 | 52.5 | 45.0 | 55.0 | 55.0 | 50.0 | 60.0 | 85.0 | 75.0 | 70.0 | 75.0 | 82.5 | 90.0 | 82.5 | 77.5 | 82.5 | 87.5 |
| p030 | 10.0 | 2.5 | 12.5 | 7.5 | 17.5 | 35.0 | 47.5 | 62.5 | 77.5 | 85.0 | 92.5 | 15.0 | 15.0 | 15.0 | 25.0 | 30.0 | 55.0 | 60.0 | 72.5 | 90.0 | 90.0 | 97.5 | 20.0 | 25.0 | 30.0 | 27.5 | 20.0 | 40.0 | 42.5 | 42.5 | 55.0 | 52.5 | 60.0 | 50.0 | 47.5 | 60.0 | 52.5 | 65.0 | 57.5 | 65.0 | 65.0 | 77.5 | 75.0 | 77.5 |

## Exclude non-compliant Participants¶

The outlier participant shows behavior that is exactly opposite to all other participants. The participant reported to have focused so intensly on the lower face half that she ignored the change of instructions and always rated the lower half. It is noteworthy that she reached a remarably good (and s-shaped) response for the lower half, unlike virtually everybody else. This in itself is very interesting in its own right, as it indicates that under some cirumstances the bias reported in our manuscript can be overcome.

In [18]:

```
rev_avg_between_df = pd.concat([avg_between_df[0:13], avg_between_df[14:] ])
rev_pt1_between_df = pd.concat([pt1_between_df[0:13], pt1_between_df[14:] ])
rev_pt2_between_df = pd.concat([pt2_between_df[0:13], pt2_between_df[14:] ])
rev_avg_between_df
```

Out[18]:

|  | fearLOW | | | | | | | | | | | angerLOW | | | | | | | | | | | fearUP | | | | | | | | | | | angerUP | | | | | | | | | | |
| --- | --- | --- | --- | --- | --- | --- | --- | --- | --- | --- | --- | --- | --- | --- | --- | --- | --- | --- | --- | --- | --- | --- | --- | --- | --- | --- | --- | --- | --- | --- | --- | --- | --- | --- | --- | --- | --- | --- | --- | --- | --- | --- | --- | --- |
| grade | m00 | m01 | m02 | m03 | m04 | m05 | m06 | m07 | m08 | m09 | m10 | m00 | m01 | m02 | m03 | m04 | m05 | m06 | m07 | m08 | m09 | m10 | m00 | m01 | m02 | m03 | m04 | m05 | m06 | m07 | m08 | m09 | m10 | m00 | m01 | m02 | m03 | m04 | m05 | m06 | m07 | m08 | m09 | m10 |
| p |  |  |  |  |  |  |  |  |  |  |  |  |  |  |  |  |  |  |  |  |  |  |  |  |  |  |  |  |  |  |  |  |  |  |  |  |  |  |  |  |  |  |  |  |
| p001 | 15.0 | 25.0 | 42.5 | 40.0 | 42.5 | 50.0 | 55.0 | 62.5 | 77.5 | 77.5 | 80.0 | 25.0 | 32.5 | 32.5 | 45.0 | 37.5 | 52.5 | 62.5 | 67.5 | 75.0 | 75.0 | 87.5 | 35.0 | 32.5 | 42.5 | 40.0 | 42.5 | 45.0 | 42.5 | 42.5 | 45.0 | 52.5 | 50.0 | 57.5 | 42.5 | 55.0 | 57.5 | 62.5 | 50.0 | 57.5 | 57.5 | 67.5 | 60.0 | 70.0 |
| p002 | 40.0 | 27.5 | 57.5 | 42.5 | 52.5 | 50.0 | 60.0 | 87.5 | 80.0 | 85.0 | 87.5 | 27.5 | 52.5 | 52.5 | 52.5 | 60.0 | 57.5 | 75.0 | 87.5 | 67.5 | 82.5 | 82.5 | 40.0 | 40.0 | 40.0 | 35.0 | 50.0 | 42.5 | 42.5 | 45.0 | 47.5 | 47.5 | 50.0 | 67.5 | 72.5 | 80.0 | 72.5 | 67.5 | 72.5 | 77.5 | 82.5 | 85.0 | 95.0 | 90.0 |
| p003 | 10.0 | 7.5 | 15.0 | 10.0 | 12.5 | 35.0 | 52.5 | 65.0 | 90.0 | 90.0 | 97.5 | 22.5 | 10.0 | 22.5 | 20.0 | 40.0 | 57.5 | 82.5 | 85.0 | 92.5 | 95.0 | 95.0 | 32.5 | 30.0 | 35.0 | 30.0 | 32.5 | 42.5 | 45.0 | 57.5 | 47.5 | 52.5 | 52.5 | 62.5 | 65.0 | 52.5 | 60.0 | 67.5 | 62.5 | 57.5 | 70.0 | 72.5 | 67.5 | 75.0 |
| p004 | 12.5 | 17.5 | 17.5 | 25.0 | 25.0 | 35.0 | 45.0 | 60.0 | 42.5 | 52.5 | 60.0 | 35.0 | 37.5 | 47.5 | 60.0 | 57.5 | 55.0 | 65.0 | 65.0 | 77.5 | 85.0 | 85.0 | 37.5 | 22.5 | 37.5 | 42.5 | 45.0 | 47.5 | 60.0 | 55.0 | 62.5 | 50.0 | 57.5 | 57.5 | 65.0 | 60.0 | 60.0 | 70.0 | 75.0 | 85.0 | 82.5 | 85.0 | 85.0 | 80.0 |
| p005 | 2.5 | 12.5 | 17.5 | 20.0 | 25.0 | 35.0 | 52.5 | 70.0 | 82.5 | 77.5 | 92.5 | 17.5 | 22.5 | 20.0 | 25.0 | 42.5 | 55.0 | 70.0 | 90.0 | 92.5 | 92.5 | 97.5 | 5.0 | 5.0 | 7.5 | 7.5 | 15.0 | 12.5 | 12.5 | 20.0 | 22.5 | 15.0 | 10.0 | 92.5 | 90.0 | 90.0 | 95.0 | 92.5 | 95.0 | 92.5 | 92.5 | 97.5 | 100.0 | 100.0 |
| p006 | 40.0 | 32.5 | 42.5 | 37.5 | 50.0 | 50.0 | 52.5 | 65.0 | 75.0 | 65.0 | 77.5 | 32.5 | 35.0 | 37.5 | 42.5 | 57.5 | 60.0 | 65.0 | 72.5 | 67.5 | 77.5 | 97.5 | 37.5 | 45.0 | 47.5 | 60.0 | 52.5 | 40.0 | 55.0 | 67.5 | 50.0 | 50.0 | 37.5 | 62.5 | 72.5 | 60.0 | 65.0 | 60.0 | 77.5 | 57.5 | 77.5 | 67.5 | 72.5 | 67.5 |
| p007 | 17.5 | 22.5 | 22.5 | 25.0 | 22.5 | 30.0 | 50.0 | 55.0 | 72.5 | 77.5 | 85.0 | 30.0 | 37.5 | 32.5 | 32.5 | 37.5 | 45.0 | 67.5 | 90.0 | 80.0 | 82.5 | 82.5 | 25.0 | 17.5 | 30.0 | 20.0 | 22.5 | 25.0 | 20.0 | 37.5 | 30.0 | 35.0 | 37.5 | 92.5 | 85.0 | 82.5 | 90.0 | 87.5 | 85.0 | 95.0 | 87.5 | 92.5 | 92.5 | 85.0 |
| p008 | 22.5 | 25.0 | 27.5 | 30.0 | 32.5 | 40.0 | 50.0 | 50.0 | 60.0 | 65.0 | 72.5 | 22.5 | 30.0 | 35.0 | 42.5 | 60.0 | 65.0 | 65.0 | 67.5 | 75.0 | 82.5 | 70.0 | 37.5 | 42.5 | 30.0 | 42.5 | 52.5 | 45.0 | 55.0 | 67.5 | 62.5 | 55.0 | 75.0 | 62.5 | 57.5 | 70.0 | 65.0 | 70.0 | 77.5 | 80.0 | 77.5 | 92.5 | 80.0 | 87.5 |
| p009 | 42.5 | 35.0 | 40.0 | 40.0 | 62.5 | 40.0 | 50.0 | 62.5 | 70.0 | 75.0 | 82.5 | 32.5 | 45.0 | 40.0 | 47.5 | 42.5 | 50.0 | 60.0 | 67.5 | 87.5 | 77.5 | 77.5 | 37.5 | 47.5 | 52.5 | 42.5 | 45.0 | 47.5 | 50.0 | 45.0 | 50.0 | 45.0 | 47.5 | 70.0 | 70.0 | 75.0 | 70.0 | 77.5 | 62.5 | 65.0 | 62.5 | 65.0 | 77.5 | 65.0 |
| p010 | 7.5 | 15.0 | 22.5 | 22.5 | 25.0 | 50.0 | 55.0 | 52.5 | 82.5 | 82.5 | 87.5 | 32.5 | 10.0 | 27.5 | 30.0 | 35.0 | 52.5 | 70.0 | 77.5 | 82.5 | 85.0 | 95.0 | 17.5 | 12.5 | 12.5 | 20.0 | 15.0 | 22.5 | 25.0 | 32.5 | 20.0 | 30.0 | 42.5 | 55.0 | 67.5 | 57.5 | 70.0 | 57.5 | 75.0 | 72.5 | 80.0 | 72.5 | 80.0 | 90.0 |
| p011 | 15.0 | 7.5 | 10.0 | 20.0 | 30.0 | 37.5 | 55.0 | 80.0 | 70.0 | 82.5 | 92.5 | 7.5 | 17.5 | 20.0 | 20.0 | 30.0 | 40.0 | 60.0 | 72.5 | 82.5 | 90.0 | 95.0 | 30.0 | 37.5 | 30.0 | 42.5 | 50.0 | 60.0 | 65.0 | 70.0 | 75.0 | 85.0 | 82.5 | 47.5 | 37.5 | 45.0 | 35.0 | 60.0 | 60.0 | 72.5 | 82.5 | 82.5 | 82.5 | 80.0 |
| p012 | 7.5 | 12.5 | 10.0 | 17.5 | 22.5 | 40.0 | 50.0 | 62.5 | 60.0 | 85.0 | 92.5 | 22.5 | 22.5 | 30.0 | 27.5 | 42.5 | 47.5 | 65.0 | 67.5 | 92.5 | 92.5 | 95.0 | 12.5 | 15.0 | 15.0 | 7.5 | 15.0 | 20.0 | 22.5 | 40.0 | 35.0 | 40.0 | 42.5 | 57.5 | 55.0 | 47.5 | 55.0 | 65.0 | 60.0 | 77.5 | 77.5 | 82.5 | 92.5 | 82.5 |
| p013 | 12.5 | 17.5 | 12.5 | 10.0 | 22.5 | 30.0 | 22.5 | 40.0 | 45.0 | 47.5 | 62.5 | 32.5 | 27.5 | 35.0 | 30.0 | 37.5 | 47.5 | 32.5 | 57.5 | 55.0 | 62.5 | 62.5 | 25.0 | 32.5 | 30.0 | 22.5 | 22.5 | 27.5 | 32.5 | 32.5 | 27.5 | 35.0 | 40.0 | 52.5 | 50.0 | 37.5 | 42.5 | 52.5 | 47.5 | 47.5 | 57.5 | 52.5 | 45.0 | 62.5 |
| p015 | 5.0 | 12.5 | 12.5 | 20.0 | 25.0 | 40.0 | 50.0 | 72.5 | 67.5 | 82.5 | 85.0 | 12.5 | 20.0 | 20.0 | 30.0 | 32.5 | 47.5 | 50.0 | 67.5 | 85.0 | 80.0 | 87.5 | 45.0 | 47.5 | 32.5 | 42.5 | 35.0 | 40.0 | 47.5 | 45.0 | 45.0 | 47.5 | 42.5 | 72.5 | 47.5 | 50.0 | 55.0 | 42.5 | 57.5 | 57.5 | 42.5 | 62.5 | 52.5 | 50.0 |
| p016 | 7.5 | 15.0 | 7.5 | 17.5 | 20.0 | 35.0 | 45.0 | 57.5 | 67.5 | 77.5 | 95.0 | 17.5 | 20.0 | 25.0 | 27.5 | 37.5 | 45.0 | 55.0 | 62.5 | 77.5 | 75.0 | 92.5 | 17.5 | 27.5 | 15.0 | 30.0 | 27.5 | 37.5 | 40.0 | 60.0 | 55.0 | 52.5 | 67.5 | 47.5 | 60.0 | 67.5 | 52.5 | 70.0 | 72.5 | 80.0 | 80.0 | 82.5 | 80.0 | 77.5 |
| p017 | 27.5 | 25.0 | 22.5 | 50.0 | 45.0 | 40.0 | 55.0 | 72.5 | 65.0 | 85.0 | 85.0 | 30.0 | 40.0 | 30.0 | 37.5 | 47.5 | 55.0 | 65.0 | 70.0 | 72.5 | 87.5 | 90.0 | 10.0 | 22.5 | 27.5 | 20.0 | 35.0 | 37.5 | 27.5 | 30.0 | 30.0 | 32.5 | 47.5 | 35.0 | 40.0 | 35.0 | 45.0 | 35.0 | 40.0 | 45.0 | 42.5 | 37.5 | 42.5 | 42.5 |
| p018 | 15.0 | 12.5 | 15.0 | 27.5 | 32.5 | 47.5 | 47.5 | 75.0 | 75.0 | 87.5 | 90.0 | 25.0 | 30.0 | 30.0 | 30.0 | 40.0 | 47.5 | 65.0 | 77.5 | 87.5 | 95.0 | 92.5 | 40.0 | 47.5 | 55.0 | 40.0 | 62.5 | 50.0 | 47.5 | 47.5 | 50.0 | 55.0 | 67.5 | 77.5 | 80.0 | 82.5 | 82.5 | 80.0 | 80.0 | 80.0 | 90.0 | 87.5 | 92.5 | 82.5 |
| p019 | 25.0 | 32.5 | 30.0 | 37.5 | 35.0 | 40.0 | 50.0 | 72.5 | 72.5 | 77.5 | 87.5 | 30.0 | 40.0 | 30.0 | 30.0 | 42.5 | 65.0 | 62.5 | 60.0 | 87.5 | 90.0 | 90.0 | 25.0 | 22.5 | 22.5 | 25.0 | 17.5 | 35.0 | 30.0 | 37.5 | 32.5 | 37.5 | 32.5 | 65.0 | 67.5 | 62.5 | 65.0 | 75.0 | 72.5 | 80.0 | 72.5 | 77.5 | 82.5 | 92.5 |
| p020 | 17.5 | 15.0 | 20.0 | 22.5 | 25.0 | 32.5 | 40.0 | 50.0 | 50.0 | 55.0 | 60.0 | 55.0 | 55.0 | 47.5 | 62.5 | 60.0 | 65.0 | 87.5 | 90.0 | 80.0 | 85.0 | 92.5 | 15.0 | 20.0 | 25.0 | 25.0 | 35.0 | 55.0 | 57.5 | 75.0 | 82.5 | 82.5 | 92.5 | 25.0 | 27.5 | 20.0 | 25.0 | 32.5 | 47.5 | 70.0 | 75.0 | 82.5 | 82.5 | 87.5 |
| p021 | 10.0 | 7.5 | 15.0 | 15.0 | 25.0 | 37.5 | 50.0 | 70.0 | 80.0 | 92.5 | 85.0 | 12.5 | 5.0 | 12.5 | 27.5 | 25.0 | 45.0 | 65.0 | 75.0 | 85.0 | 90.0 | 90.0 | 2.5 | 10.0 | 5.0 | 10.0 | 10.0 | 22.5 | 27.5 | 27.5 | 22.5 | 32.5 | 32.5 | 40.0 | 40.0 | 37.5 | 45.0 | 42.5 | 50.0 | 52.5 | 57.5 | 60.0 | 60.0 | 55.0 |
| p022 | 22.5 | 27.5 | 30.0 | 42.5 | 47.5 | 47.5 | 65.0 | 72.5 | 77.5 | 87.5 | 87.5 | 30.0 | 32.5 | 40.0 | 52.5 | 52.5 | 52.5 | 52.5 | 75.0 | 77.5 | 85.0 | 92.5 | 55.0 | 65.0 | 72.5 | 62.5 | 67.5 | 62.5 | 65.0 | 55.0 | 75.0 | 62.5 | 70.0 | 77.5 | 72.5 | 72.5 | 70.0 | 85.0 | 80.0 | 72.5 | 75.0 | 67.5 | 77.5 | 72.5 |
| p023 | 15.0 | 20.0 | 20.0 | 20.0 | 30.0 | 45.0 | 60.0 | 85.0 | 80.0 | 82.5 | 80.0 | 25.0 | 20.0 | 22.5 | 35.0 | 42.5 | 40.0 | 72.5 | 82.5 | 87.5 | 87.5 | 92.5 | 32.5 | 27.5 | 42.5 | 27.5 | 42.5 | 57.5 | 42.5 | 60.0 | 62.5 | 80.0 | 75.0 | 50.0 | 47.5 | 47.5 | 42.5 | 50.0 | 70.0 | 75.0 | 87.5 | 90.0 | 75.0 | 85.0 |
| p024 | 2.5 | 10.0 | 20.0 | 20.0 | 27.5 | 45.0 | 50.0 | 72.5 | 82.5 | 90.0 | 92.5 | 15.0 | 25.0 | 25.0 | 30.0 | 40.0 | 55.0 | 62.5 | 77.5 | 80.0 | 95.0 | 90.0 | 5.0 | 20.0 | 15.0 | 10.0 | 25.0 | 27.5 | 25.0 | 30.0 | 30.0 | 32.5 | 30.0 | 22.5 | 37.5 | 40.0 | 35.0 | 47.5 | 55.0 | 50.0 | 57.5 | 67.5 | 57.5 | 75.0 |
| p025 | 50.0 | 35.0 | 40.0 | 40.0 | 50.0 | 55.0 | 55.0 | 65.0 | 75.0 | 75.0 | 87.5 | 60.0 | 60.0 | 62.5 | 60.0 | 75.0 | 67.5 | 67.5 | 82.5 | 87.5 | 90.0 | 97.5 | 50.0 | 50.0 | 45.0 | 60.0 | 55.0 | 65.0 | 60.0 | 67.5 | 77.5 | 75.0 | 67.5 | 67.5 | 67.5 | 62.5 | 70.0 | 72.5 | 82.5 | 90.0 | 85.0 | 82.5 | 82.5 | 90.0 |
| p026 | 10.0 | 12.5 | 15.0 | 12.5 | 22.5 | 40.0 | 45.0 | 70.0 | 72.5 | 90.0 | 92.5 | 32.5 | 32.5 | 32.5 | 35.0 | 45.0 | 60.0 | 65.0 | 90.0 | 92.5 | 100.0 | 100.0 | 27.5 | 32.5 | 22.5 | 25.0 | 45.0 | 35.0 | 45.0 | 45.0 | 52.5 | 55.0 | 67.5 | 57.5 | 62.5 | 70.0 | 80.0 | 95.0 | 82.5 | 95.0 | 90.0 | 92.5 | 90.0 | 100.0 |
| p027 | 30.0 | 27.5 | 12.5 | 25.0 | 37.5 | 40.0 | 52.5 | 37.5 | 55.0 | 67.5 | 67.5 | 30.0 | 40.0 | 30.0 | 32.5 | 47.5 | 30.0 | 55.0 | 62.5 | 62.5 | 65.0 | 65.0 | 40.0 | 42.5 | 47.5 | 37.5 | 45.0 | 60.0 | 47.5 | 62.5 | 55.0 | 55.0 | 60.0 | 65.0 | 60.0 | 52.5 | 57.5 | 75.0 | 62.5 | 62.5 | 62.5 | 52.5 | 65.0 | 60.0 |
| p028 | 10.0 | 10.0 | 17.5 | 27.5 | 40.0 | 57.5 | 77.5 | 97.5 | 97.5 | 100.0 | 100.0 | 20.0 | 20.0 | 20.0 | 27.5 | 42.5 | 75.0 | 87.5 | 100.0 | 100.0 | 100.0 | 100.0 | 27.5 | 35.0 | 32.5 | 30.0 | 45.0 | 50.0 | 60.0 | 70.0 | 75.0 | 75.0 | 80.0 | 47.5 | 52.5 | 70.0 | 75.0 | 82.5 | 80.0 | 95.0 | 92.5 | 95.0 | 95.0 | 97.5 |
| p029 | 10.0 | 12.5 | 17.5 | 32.5 | 20.0 | 50.0 | 55.0 | 65.0 | 90.0 | 92.5 | 95.0 | 22.5 | 17.5 | 20.0 | 20.0 | 27.5 | 52.5 | 62.5 | 90.0 | 90.0 | 92.5 | 92.5 | 32.5 | 37.5 | 40.0 | 50.0 | 40.0 | 42.5 | 52.5 | 45.0 | 55.0 | 55.0 | 50.0 | 60.0 | 85.0 | 75.0 | 70.0 | 75.0 | 82.5 | 90.0 | 82.5 | 77.5 | 82.5 | 87.5 |
| p030 | 10.0 | 2.5 | 12.5 | 7.5 | 17.5 | 35.0 | 47.5 | 62.5 | 77.5 | 85.0 | 92.5 | 15.0 | 15.0 | 15.0 | 25.0 | 30.0 | 55.0 | 60.0 | 72.5 | 90.0 | 90.0 | 97.5 | 20.0 | 25.0 | 30.0 | 27.5 | 20.0 | 40.0 | 42.5 | 42.5 | 55.0 | 52.5 | 60.0 | 50.0 | 47.5 | 60.0 | 52.5 | 65.0 | 57.5 | 65.0 | 65.0 | 77.5 | 75.0 | 77.5 |

## Save df to file¶

In [19]:

```
def savePandas(where,df,csv):
    os.chdir(where)
    df.to_csv(csv)
```

In [20]:

```
my_folder = './data/'

savePandas(my_folder,pt1_between_df,'Exp2Pt1ResultsAll.txt')
savePandas(my_folder,rev_pt1_between_df,'Exp2Pt1Results.txt')

savePandas(my_folder,pt2_between_df,'Exp2Pt2ResultsAll.txt')
savePandas(my_folder,rev_pt2_between_df,'Exp2Pt2Results.txt')

savePandas(my_folder,avg_between_df,'Exp2AvgResultsAll.txt')
savePandas(my_folder,rev_avg_between_df,'Exp2AvgResults.txt')
```

# Reaction Time Extraction¶

## Get Reaction Times for one condition¶

In [21]:

```
def get_ident_rt(d,cond):
    morphs = ['_00_','_01_','_02_','_03_','_04_','_05_','_06_','_07_','_08_','_09_','_10_']
    d_names = ['m00','m01','m02','m03','m04','m05','m06','m07','m08','m09','m10']
    d_median = {'m00':{},'m01':{},'m02':{},'m03':{},'m04':{},'m05':{},'m06':{},'m07':{},'m08':{},'m09':{},'m10':{}}
    d_raw = {'m00':{},'m01':{},'m02':{},'m03':{},'m04':{},'m05':{},'m06':{},'m07':{},'m08':{},'m09':{},'m10':{}}
    j = 0
    for m in morphs:
        this_morph = []
        i = 0
        
        for entry in d['pt1'][cond]:
            if d['pt1'][cond][entry]['target'].find(m) != -1:
                    this_morph.append(d['pt1'][cond][entry]['rt'])
        for entry in d['pt2'][cond]:
            if d['pt2'][cond][entry]['target'].find(m) != -1:
                    this_morph.append(d['pt2'][cond][entry]['rt'])            
        d_raw[d_names[j]] = this_morph
        d_median[d_names[j]] = median(this_morph)
        j += 1
    return d_median, d_raw
```

Example:

Median Values:

In [22]:

```
get_ident_rt(mydict,'low00')[0]
```

Out[22]:

```
{'m00': 1078.5999999999999,
 'm01': 1120.05,
 'm02': 1129.1500000000001,
 'm03': 1302.0,
 'm04': 959.04999999999995,
 'm05': 1523.25,
 'm06': 1279.25,
 'm07': 1210.8,
 'm08': 1241.1999999999998,
 'm09': 999.14999999999998,
 'm10': 983.20000000000005}
```

Histograms:

In [23]:

```
for e in get_ident_rt(mydict,'low00')[1]:
    hist(get_ident_rt(mydict,'low00')[1][e])
```

## Get Data for all conditions¶

In [24]:

```
def get_ident_cond_rt(d):
    d_conds = {'up10':{}, 'low10':{}, 'up00':{}, 'low00':{} }
    d_names = ['up10','low10','up00','low00']
    i = 0
    for c in d_names:
        d_conds[d_names[i]] = get_ident_rt(d,d_names[i])[0]
        i += 1
    return pd.DataFrame(d_conds)
```

Example:

In [25]:

```
get_ident_cond_rt(mydict)
```

Out[25]:

|  | low00 | low10 | up00 | up10 |
| --- | --- | --- | --- | --- |
| m00 | 1078.60 | 1029.60 | 1005.90 | 1006.90 |
| m01 | 1120.05 | 1116.40 | 962.45 | 1138.70 |
| m02 | 1129.15 | 1113.45 | 971.30 | 961.80 |
| m03 | 1302.00 | 931.40 | 936.05 | 858.10 |
| m04 | 959.05 | 1077.65 | 1010.75 | 816.70 |
| m05 | 1523.25 | 1298.45 | 890.25 | 898.75 |
| m06 | 1279.25 | 1281.30 | 894.05 | 999.50 |
| m07 | 1210.80 | 1145.20 | 882.00 | 799.85 |
| m08 | 1241.20 | 1108.95 | 990.30 | 834.70 |
| m09 | 999.15 | 1091.20 | 971.20 | 805.60 |
| m10 | 983.20 | 929.05 | 866.80 | 811.30 |

## Make Whole Sample:¶

In [26]:

```
def make_ident_rt_sample(VpList):
    # make the first subject to establish the df-structure

    df = get_ident_cond_rt(get_ident_data(VpList[0]))
    df.index = [["p001"]*len(df), df.index ]
    
    # make the rest of the sample
    for vp_nr in range(1,len(VpList)):
        # make the following subjects
        this_vp = get_ident_cond_rt(get_ident_data(VpList[vp_nr]))
        
        # get the naming of the subject right
        if vp_nr < 10-1:
            this_vp.index = [["p00"+str(vp_nr+1) ]*len(this_vp), this_vp.index ]
        if vp_nr >=10-1:
            this_vp.index = [[ "p0"+str(vp_nr+1) ]*len(this_vp), this_vp.index ]

        # add this subject to sample-df
        df = pd.concat([df,this_vp])

    # finalising the df
    df.index.names = ['p','grade']
    df.columns = ['fearLOW','angerLOW','fearUP','angerUP']
    
    return df
```

In [27]:

```
os.chdir('../Exp2/')

ident_rt_df = make_ident_rt_sample(IdentList)
ident_rt_df
```

Out[27]:

|  |  | fearLOW | angerLOW | fearUP | angerUP |
| --- | --- | --- | --- | --- | --- |
| p | grade |  |  |  |  |
| p001 | m00 | 1078.60 | 1029.60 | 1005.90 | 1006.90 |
|  | m01 | 1120.05 | 1116.40 | 962.45 | 1138.70 |
|  | m02 | 1129.15 | 1113.45 | 971.30 | 961.80 |
|  | m03 | 1302.00 | 931.40 | 936.05 | 858.10 |
|  | m04 | 959.05 | 1077.65 | 1010.75 | 816.70 |
|  | m05 | 1523.25 | 1298.45 | 890.25 | 898.75 |
|  | m06 | 1279.25 | 1281.30 | 894.05 | 999.50 |
|  | m07 | 1210.80 | 1145.20 | 882.00 | 799.85 |
|  | m08 | 1241.20 | 1108.95 | 990.30 | 834.70 |
|  | m09 | 999.15 | 1091.20 | 971.20 | 805.60 |
|  | m10 | 983.20 | 929.05 | 866.80 | 811.30 |
| p002 | m00 | 1000.15 | 1156.10 | 1620.65 | 1551.10 |
|  | m01 | 1389.15 | 919.75 | 1677.60 | 1584.45 |
|  | m02 | 1116.60 | 963.85 | 1242.90 | 1584.40 |
|  | m03 | 931.25 | 909.05 | 1631.15 | 1343.55 |
|  | m04 | 1130.30 | 1184.35 | 2152.30 | 1630.40 |
|  | m05 | 1592.30 | 1067.70 | 2043.05 | 1372.55 |
|  | m06 | 984.70 | 992.50 | 2072.15 | 1250.50 |
|  | m07 | 902.75 | 1163.45 | 1501.85 | 1442.50 |
|  | m08 | 983.50 | 1011.25 | 1596.55 | 1259.40 |
|  | m09 | 900.25 | 969.40 | 2052.60 | 1503.10 |
|  | m10 | 863.15 | 937.85 | 1391.35 | 1427.20 |
| p003 | m00 | 1990.95 | 1882.00 | 1555.40 | 1972.50 |
|  | m01 | 1552.65 | 2270.60 | 1772.95 | 1675.95 |
|  | m02 | 2322.00 | 2471.15 | 1651.85 | 1530.30 |
|  | m03 | 2282.40 | 2556.60 | 1969.75 | 1648.40 |
|  | m04 | 2116.40 | 2022.25 | 1907.90 | 1663.85 |
|  | m05 | 2546.90 | 2408.50 | 1594.05 | 1806.55 |
|  | m06 | 2603.35 | 2049.15 | 1924.05 | 1538.65 |
|  | m07 | 2168.45 | 2808.65 | 1868.55 | 1620.15 |
|  | m08 | 1924.30 | 1416.55 | 1519.20 | 1361.30 |
|  | m09 | 1566.45 | 1624.35 | 2363.30 | 1873.35 |
|  | m10 | 1539.95 | 1336.25 | 2462.70 | 1774.90 |
| p004 | m00 | 1055.75 | 1019.55 | 1142.25 | 1000.35 |
|  | m01 | 994.15 | 1079.25 | 1099.35 | 1108.80 |
|  | m02 | 1081.90 | 949.75 | 1200.70 | 1139.75 |
|  | m03 | 1074.65 | 1182.35 | 1295.05 | 904.80 |
|  | m04 | 1219.90 | 1110.25 | 1303.40 | 1076.60 |
|  | m05 | 1113.75 | 1224.40 | 1179.50 | 1180.40 |
|  | m06 | 1015.45 | 1017.20 | 1227.20 | 1047.25 |
|  | m07 | 1085.80 | 853.45 | 1095.65 | 1047.10 |
|  | m08 | 1057.30 | 1050.10 | 1065.00 | 975.60 |
|  | m09 | 895.00 | 956.35 | 995.25 | 1124.75 |
|  | m10 | 929.20 | 929.40 | 1093.30 | 1040.20 |
| p005 | m00 | 1127.00 | 1092.05 | 998.80 | 982.00 |
|  | m01 | 1198.35 | 937.30 | 1141.05 | 971.65 |
|  | m02 | 1000.90 | 1311.80 | 1033.70 | 886.65 |
|  | m03 | 1039.40 | 1098.75 | 1067.35 | 900.65 |
|  | m04 | 1873.35 | 1050.85 | 977.30 | 885.70 |
|  | m05 | 1568.85 | 1603.35 | 1150.55 | 911.40 |
|  | m06 | 1619.05 | 1315.20 | 1023.90 | 899.25 |
|  | m07 | 1771.25 | 1026.00 | 1329.55 | 856.00 |
|  | m08 | 1158.10 | 1129.70 | 1214.45 | 874.65 |
|  | m09 | 1305.90 | 887.85 | 1033.55 | 946.80 |
|  | m10 | 980.20 | 921.75 | 1132.20 | 911.05 |
| p006 | m00 | 768.65 | 817.80 | 739.90 | 980.10 |
|  | m01 | 824.20 | 865.50 | 1025.75 | 899.45 |
|  | m02 | 815.90 | 914.25 | 855.10 | 802.25 |
|  | m03 | 766.85 | 1006.35 | 811.15 | 719.15 |
|  | m04 | 764.95 | 858.55 | 786.95 | 901.25 |
|  | m05 | 850.00 | 829.90 | 827.45 | 770.85 |
|  | m06 | 751.80 | 819.80 | 817.50 | 803.25 |
|  | m07 | 864.55 | 750.00 | 850.65 | 783.90 |
|  | m08 | 775.90 | 789.45 | 996.80 | 927.20 |
|  | m09 | 811.95 | 878.65 | 899.25 | 726.00 |
|  | m10 | 759.20 | 878.00 | 891.05 | 926.90 |
| p007 | m00 | 865.20 | 788.60 | 767.25 | 812.35 |
|  | m01 | 705.50 | 791.35 | 855.15 | 712.65 |
|  | m02 | 748.45 | 653.35 | 700.55 | 770.20 |
|  | m03 | 770.15 | 756.60 | 627.55 | 702.25 |
|  | m04 | 755.85 | 903.35 | 780.35 | 784.55 |
|  | m05 | 836.20 | 805.75 | 776.60 | 746.80 |
|  | m06 | 882.25 | 1053.50 | 745.25 | 728.45 |
|  | m07 | 783.35 | 861.40 | 742.35 | 743.20 |
|  | m08 | 798.80 | 766.95 | 711.65 | 783.35 |
|  | m09 | 848.05 | 751.20 | 770.80 | 722.20 |
|  | m10 | 958.75 | 761.85 | 735.60 | 659.85 |
| p008 | m00 | 1103.70 | 1140.05 | 1359.65 | 1210.95 |
|  | m01 | 1016.75 | 1040.35 | 1035.50 | 1226.40 |
|  | m02 | 1400.75 | 995.90 | 1009.10 | 1344.05 |
|  | m03 | 1364.75 | 1039.05 | 1237.15 | 1065.75 |
|  | m04 | 1263.10 | 1287.75 | 1307.15 | 1159.10 |
|  | m05 | 1332.50 | 1083.25 | 1103.25 | 1144.25 |
|  | m06 | 1160.95 | 1080.15 | 1171.15 | 1035.85 |
|  | m07 | 1235.40 | 1228.45 | 1269.20 | 1053.95 |
|  | m08 | 1075.95 | 1052.50 | 1305.95 | 1105.60 |
|  | m09 | 1097.45 | 1115.40 | 1223.20 | 1172.40 |
|  | m10 | 1152.25 | 1080.65 | 1336.40 | 1202.65 |
| p009 | m00 | 1403.60 | 1657.50 | 1359.55 | 1089.55 |
|  | m01 | 1423.50 | 1690.75 | 1249.70 | 979.75 |
|  | m02 | 1365.15 | 1255.40 | 1124.10 | 1273.40 |
|  | m03 | 1700.35 | 1412.30 | 1089.35 | 1179.15 |
|  | m04 | 1384.95 | 1411.20 | 1474.95 | 979.65 |
|  | m05 | 1596.05 | 1436.35 | 1201.65 | 988.20 |
|  | m06 | 1459.15 | 1347.75 | 1119.20 | 1175.90 |
|  | m07 | 1448.55 | 2095.80 | 1144.95 | 1300.55 |
|  | m08 | 1471.40 | 1263.30 | 1006.40 | 1169.25 |
|  | m09 | 1360.75 | 1356.80 | 1108.55 | 1228.50 |
|  | m10 | 1353.55 | 1411.80 | 1403.50 | 1057.30 |
| p010 | m00 | 1029.85 | 916.65 | 1063.15 | 1157.35 |
|  | m01 | 1099.70 | 1099.95 | 1091.80 | 1075.45 |
|  | m02 | 957.15 | 959.35 | 1017.20 | 1126.35 |
|  | m03 | 1073.80 | 1019.80 | 895.55 | 995.55 |
|  | m04 | 1059.70 | 956.25 | 914.95 | 1064.90 |
|  | m05 | 1269.50 | 1276.45 | 1148.05 | 1026.45 |
|  | m06 | 1278.95 | 1115.35 | 1050.35 | 1021.60 |
|  | m07 | 1104.55 | 1034.50 | 979.55 | 1014.65 |
|  | m08 | 1102.80 | 1040.70 | 1043.05 | 1053.05 |
|  | m09 | 1141.70 | 1077.65 | 1069.75 | 902.60 |
|  | m10 | 1196.50 | 1074.35 | 1058.40 | 1032.90 |
| p011 | m00 | 1227.40 | 1135.15 | 1138.10 | 921.35 |
|  | m01 | 1139.25 | 992.10 | 944.45 | 1030.30 |
|  | m02 | 1212.95 | 1056.35 | 1036.90 | 1008.65 |
|  | m03 | 1090.80 | 1227.25 | 1340.20 | 1150.40 |
|  | m04 | 1893.35 | 1517.75 | 1355.25 | 1026.10 |
|  | m05 | 1422.05 | 1216.10 | 1145.80 | 1104.60 |
|  | m06 | 1277.95 | 2163.50 | 1130.65 | 1013.90 |
|  | m07 | 1205.90 | 1230.05 | 1032.55 | 1024.65 |
|  | m08 | 1235.30 | 1114.95 | 1234.55 | 948.15 |
|  | m09 | 1015.05 | 967.20 | 864.75 | 856.10 |
|  | m10 | 911.20 | 851.15 | 930.40 | 946.65 |
| p012 | m00 | 907.55 | 1007.25 | 1128.50 | 1611.60 |
|  | m01 | 971.15 | 992.65 | 1220.10 | 1643.20 |
|  | m02 | 915.20 | 944.05 | 1506.40 | 1480.60 |
|  | m03 | 1002.75 | 1150.10 | 1080.45 | 1479.15 |
|  | m04 | 1005.65 | 993.90 | 1282.65 | 1580.45 |
|  | m05 | 1175.45 | 1352.30 | 1238.80 | 1204.40 |
|  | m06 | 1051.35 | 1218.35 | 1308.10 | 1290.55 |
|  | m07 | 1149.30 | 1309.05 | 1229.15 | 1158.60 |
|  | m08 | 1059.25 | 988.80 | 1257.05 | 1110.25 |
|  | m09 | 1023.75 | 853.30 | 1429.85 | 1184.70 |
|  | m10 | 871.20 | 886.15 | 1409.30 | 1380.70 |
| p013 | m00 | 989.60 | 887.45 | 896.75 | 816.30 |
|  | m01 | 824.65 | 853.25 | 707.20 | 807.25 |
|  | m02 | 794.35 | 862.95 | 1013.00 | 778.50 |
|  | m03 | 821.00 | 1011.25 | 755.20 | 683.10 |
|  | m04 | 843.25 | 821.50 | 827.30 | 811.45 |
|  | m05 | 1066.95 | 970.80 | 707.50 | 735.85 |
|  | m06 | 963.20 | 813.10 | 756.85 | 787.25 |
|  | m07 | 979.55 | 1015.40 | 795.85 | 682.45 |
|  | m08 | 944.25 | 899.40 | 808.10 | 789.60 |
|  | m09 | 1160.25 | 942.50 | 876.50 | 699.60 |
|  | m10 | 1129.80 | 960.40 | 710.65 | 763.15 |
| p014 | m00 | 854.25 | 799.10 | 1269.85 | 1086.55 |
|  | m01 | 788.90 | 821.75 | 1251.15 | 1158.65 |
|  | m02 | 907.75 | 873.60 | 1396.20 | 1247.30 |
|  | m03 | 1094.10 | 863.05 | 1082.70 | 1324.10 |
|  | m04 | 957.75 | 784.50 | 1431.45 | 1263.90 |
|  | m05 | 880.55 | 777.35 | 1663.40 | 1384.05 |
|  | m06 | 927.20 | 1036.15 | 1376.20 | 1110.55 |
|  | m07 | 923.75 | 837.70 | 1471.20 | 1192.90 |
|  | m08 | 984.35 | 796.55 | 1166.15 | 1051.85 |
|  | m09 | 890.40 | 809.05 | 1137.95 | 1037.75 |
|  | m10 | 932.40 | 800.25 | 2004.90 | 1069.85 |
| p015 | m00 | 825.05 | 775.30 | 757.90 | 742.95 |
|  | m01 | 748.15 | 751.55 | 765.50 | 784.20 |
|  | m02 | 809.45 | 739.75 | 734.85 | 784.95 |
|  | m03 | 782.80 | 741.10 | 712.30 | 690.95 |
|  | m04 | 765.50 | 766.10 | 763.05 | 749.40 |
|  | m05 | 796.85 | 847.05 | 709.90 | 855.75 |
|  | m06 | 878.50 | 720.30 | 827.60 | 734.45 |
|  | m07 | 816.05 | 746.00 | 654.00 | 730.35 |
|  | m08 | 836.80 | 762.85 | 657.00 | 679.85 |
|  | m09 | 719.30 | 724.00 | 691.05 | 666.20 |
|  | m10 | 729.25 | 739.35 | 638.85 | 631.95 |
| p016 | m00 | 1161.75 | 1011.45 | 1657.90 | 1756.15 |
|  | m01 | 1112.35 | 1081.20 | 2142.80 | 1666.85 |
|  | m02 | 1276.10 | 1196.80 | 1699.05 | 1709.95 |
|  | m03 | 1170.75 | 1529.75 | 1624.90 | 1395.85 |
|  | m04 | 1134.60 | 1221.55 | 1563.05 | 1375.70 |
|  | m05 | 1404.80 | 1425.20 | 1772.10 | 1403.50 |
|  | m06 | 1345.70 | 1461.85 | 1476.60 | 2208.55 |
|  | m07 | 1238.25 | 1333.25 | 1683.70 | 1390.20 |
|  | m08 | 2157.10 | 1643.50 | 1585.80 | 1500.70 |
|  | m09 | 1044.25 | 1266.30 | 1594.00 | 1445.00 |
|  | m10 | 1202.55 | 1264.15 | 1360.00 | 1378.25 |
| p017 | m00 | 1028.10 | 1049.95 | 906.45 | 839.30 |
|  | m01 | 1140.90 | 987.85 | 933.40 | 875.10 |
|  | m02 | 1003.20 | 1094.90 | 803.60 | 888.25 |
|  | m03 | 939.20 | 1190.45 | 802.65 | 809.55 |
|  | m04 | 950.95 | 978.05 | 813.60 | 787.60 |
|  | m05 | 982.45 | 1103.15 | 853.75 | 802.55 |
|  | m06 | 955.95 | 896.40 | 869.00 | 811.60 |
|  | m07 | 967.30 | 1050.60 | 740.55 | 801.85 |
|  | m08 | 992.25 | 906.20 | 711.25 | 755.85 |
|  | m09 | 959.15 | 999.75 | 875.05 | 825.75 |
|  | m10 | 835.20 | 863.55 | 800.10 | 851.45 |
| p018 | m00 | 1197.55 | 1148.60 | 1623.70 | 1515.25 |
|  | m01 | 1178.30 | 1333.05 | 2285.20 | 1387.60 |
|  | m02 | 1306.35 | 1480.75 | 1715.65 | 1418.90 |
|  | m03 | 1395.60 | 1176.95 | 1576.20 | 1451.20 |
|  | m04 | 1295.45 | 1316.30 | 1603.30 | 1344.40 |
|  | m05 | 1459.95 | 1716.40 | 1624.60 | 1433.20 |
|  | m06 | 1517.55 | 1668.20 | 1600.65 | 1667.15 |
|  | m07 | 1436.80 | 1737.70 | 1470.20 | 1283.10 |
|  | m08 | 1279.25 | 1698.60 | 1635.25 | 1323.25 |
|  | m09 | 1276.50 | 1186.10 | 1670.40 | 1617.90 |
|  | m10 | 1326.65 | 1150.05 | 1537.15 | 1524.30 |
| p019 | m00 | 818.05 | 865.75 | 1098.25 | 919.85 |
|  | m01 | 932.00 | 842.15 | 956.10 | 954.95 |
|  | m02 | 987.25 | 1020.40 | 937.65 | 900.85 |
|  | m03 | 836.95 | 916.45 | 993.10 | 888.35 |
|  | m04 | 830.20 | 988.30 | 945.80 | 915.50 |
|  | m05 | 899.25 | 983.10 | 1174.65 | 864.35 |
|  | m06 | 1016.30 | 970.80 | 957.65 | 892.05 |
|  | m07 | 831.95 | 914.55 | 945.40 | 835.15 |
|  | m08 | 860.65 | 945.20 | 832.90 | 754.90 |
|  | m09 | 774.30 | 852.15 | 790.00 | 776.05 |
|  | m10 | 771.25 | 751.35 | 978.30 | 944.75 |
| p020 | m00 | 1268.40 | 980.20 | 1405.70 | 1390.80 |
|  | m01 | 1107.35 | 1210.55 | 1447.55 | 1207.20 |
|  | m02 | 1121.30 | 1229.80 | 1210.75 | 1372.85 |
|  | m03 | 1247.20 | 1336.15 | 1179.65 | 1312.50 |
|  | m04 | 1123.50 | 1240.20 | 1467.20 | 1244.80 |
|  | m05 | 1123.25 | 1205.85 | 1489.60 | 1412.25 |
|  | m06 | 1035.00 | 1280.40 | 1299.15 | 1638.50 |
|  | m07 | 1297.40 | 1231.75 | 1311.45 | 1402.05 |
|  | m08 | 1059.15 | 1283.40 | 1242.75 | 1565.95 |
|  | m09 | 1093.75 | 1075.75 | 1319.90 | 1307.15 |
|  | m10 | 1051.75 | 1166.50 | 1061.55 | 1302.10 |
| p021 | m00 | 916.30 | 906.70 | 881.55 | 1325.65 |
|  | m01 | 827.90 | 1072.55 | 986.25 | 968.15 |
|  | m02 | 856.05 | 1279.65 | 1126.55 | 953.00 |
|  | m03 | 871.70 | 1051.10 | 1026.50 | 1039.95 |
|  | m04 | 1034.90 | 862.10 | 961.50 | 892.50 |
|  | m05 | 1115.65 | 998.45 | 978.95 | 1122.50 |
|  | m06 | 1199.80 | 1059.95 | 1001.75 | 719.95 |
|  | m07 | 1244.70 | 920.50 | 1032.20 | 981.70 |
|  | m08 | 898.50 | 951.30 | 1007.10 | 763.10 |
|  | m09 | 888.85 | 865.25 | 1016.95 | 786.05 |
|  | m10 | 911.05 | 792.25 | 1196.75 | 834.90 |
| p022 | m00 | 756.15 | 839.50 | 394.45 | 470.90 |
|  | m01 | 719.10 | 759.30 | 447.25 | 571.20 |
|  | m02 | 857.00 | 660.15 | 500.70 | 329.00 |
|  | m03 | 716.15 | 762.60 | 397.00 | 504.35 |
|  | m04 | 755.90 | 710.90 | 271.45 | 516.10 |
|  | m05 | 717.95 | 795.60 | 442.20 | 423.20 |
|  | m06 | 797.05 | 670.30 | 450.25 | 431.80 |
|  | m07 | 774.60 | 751.90 | 329.60 | 304.95 |
|  | m08 | 716.00 | 742.75 | 291.25 | 429.65 |
|  | m09 | 612.60 | 731.25 | 396.40 | 363.50 |
|  | m10 | 684.80 | 680.95 | 426.00 | 452.80 |
| p023 | m00 | 972.15 | 1016.90 | 823.45 | 1060.70 |
|  | m01 | 915.80 | 1056.00 | 842.20 | 896.60 |
|  | m02 | 1080.05 | 1217.40 | 952.10 | 1004.55 |
|  | m03 | 1126.00 | 1094.05 | 975.10 | 1045.40 |
|  | m04 | 1120.65 | 1203.10 | 1174.95 | 1148.20 |
|  | m05 | 1106.35 | 1248.35 | 1410.45 | 979.05 |
|  | m06 | 1442.80 | 1289.40 | 967.30 | 1199.20 |
|  | m07 | 1416.10 | 1131.60 | 958.60 | 1052.40 |
|  | m08 | 1275.25 | 1108.10 | 1155.10 | 915.65 |
|  | m09 | 1314.45 | 1353.15 | 1439.25 | 992.60 |
|  | m10 | 1031.15 | 1096.80 | 1145.40 | 852.45 |
| p024 | m00 | 1079.30 | 1080.10 | 903.20 | 1809.45 |
|  | m01 | 1058.15 | 1267.20 | 989.00 | 1496.20 |
|  | m02 | 1062.30 | 1288.35 | 1201.15 | 1201.25 |
|  | m03 | 1311.30 | 1241.10 | 1340.45 | 1102.95 |
|  | m04 | 1172.75 | 1258.20 | 1365.40 | 1163.50 |
|  | m05 | 1540.55 | 1257.65 | 1059.45 | 1345.20 |
|  | m06 | 1289.40 | 1293.30 | 1077.80 | 1382.80 |
|  | m07 | 1225.70 | 1076.20 | 1030.80 | 1223.40 |
|  | m08 | 1223.15 | 1133.05 | 1213.40 | 1346.30 |
|  | m09 | 1033.55 | 1027.00 | 1072.30 | 1160.05 |
|  | m10 | 984.10 | 1088.35 | 1396.50 | 1178.40 |
| p025 | m00 | 1327.15 | 1438.40 | 1285.50 | 1414.40 |
|  | m01 | 1403.60 | 1457.35 | 1439.45 | 1455.25 |
|  | m02 | 1479.35 | 1520.05 | 1119.20 | 1387.60 |
|  | m03 | 1488.55 | 1527.25 | 1324.50 | 1748.40 |
|  | m04 | 1643.00 | 1238.30 | 1335.25 | 1346.15 |
|  | m05 | 1548.15 | 1388.85 | 1430.25 | 1242.40 |
|  | m06 | 1453.10 | 1399.15 | 1292.50 | 1218.50 |
|  | m07 | 1700.55 | 1450.75 | 1463.40 | 1171.15 |
|  | m08 | 1540.20 | 1453.55 | 1082.75 | 1272.30 |
|  | m09 | 1472.20 | 1545.60 | 1327.90 | 1125.00 |
|  | m10 | 1543.20 | 1303.75 | 1155.70 | 1238.45 |
| p026 | m00 | 1392.55 | 1438.75 | 1725.75 | 1976.70 |
|  | m01 | 1371.75 | 1589.45 | 1902.60 | 1412.55 |
|  | m02 | 1473.95 | 1311.50 | 1583.25 | 1543.75 |
|  | m03 | 1485.25 | 1320.45 | 2179.10 | 1840.55 |
|  | m04 | 1459.50 | 1621.40 | 1439.10 | 1371.25 |
|  | m05 | 2056.45 | 1922.50 | 1997.25 | 1192.15 |
|  | m06 | 1631.65 | 1852.65 | 1821.25 | 1229.60 |
|  | m07 | 1713.75 | 1422.65 | 1292.75 | 1147.25 |
|  | m08 | 1478.95 | 1096.35 | 1936.90 | 1295.40 |
|  | m09 | 1328.10 | 1175.40 | 2079.75 | 1112.30 |
|  | m10 | 1496.70 | 971.70 | 1663.10 | 1112.90 |
| p027 | m00 | 755.60 | 885.20 | 831.75 | 1019.70 |
|  | m01 | 801.20 | 793.70 | 891.30 | 812.40 |
|  | m02 | 787.20 | 783.00 | 805.90 | 852.55 |
|  | m03 | 852.15 | 859.20 | 780.55 | 851.75 |
|  | m04 | 903.10 | 839.25 | 751.25 | 886.55 |
|  | m05 | 802.50 | 758.35 | 653.65 | 729.35 |
|  | m06 | 799.45 | 744.60 | 1215.80 | 700.45 |
|  | m07 | 996.45 | 800.50 | 777.45 | 831.30 |
|  | m08 | 757.55 | 798.10 | 951.65 | 786.85 |
|  | m09 | 876.95 | 853.40 | 838.10 | 911.80 |
|  | m10 | 831.50 | 743.80 | 839.50 | 814.10 |
| p028 | m00 | 1316.70 | 1336.15 | 1732.30 | 2845.25 |
|  | m01 | 1271.10 | 1636.25 | 2589.20 | 2090.80 |
|  | m02 | 1324.60 | 1583.75 | 1832.95 | 2479.45 |
|  | m03 | 1690.85 | 1451.40 | 2243.25 | 2774.15 |
|  | m04 | 1838.10 | 1760.15 | 2886.20 | 1566.50 |
|  | m05 | 1872.45 | 1463.75 | 3539.15 | 1719.20 |
|  | m06 | 1659.25 | 1541.40 | 2184.90 | 1144.15 |
|  | m07 | 1216.50 | 1011.20 | 3105.80 | 1204.15 |
|  | m08 | 980.05 | 971.50 | 1751.10 | 1299.10 |
|  | m09 | 992.25 | 1066.85 | 1735.45 | 1110.35 |
|  | m10 | 887.40 | 794.95 | 1837.50 | 989.20 |
| p029 | m00 | 1265.45 | 1125.20 | 1594.10 | 1280.20 |
|  | m01 | 1266.60 | 1302.80 | 1352.30 | 1389.60 |
|  | m02 | 1328.75 | 1284.25 | 1269.15 | 1063.70 |
|  | m03 | 1173.80 | 1399.95 | 1101.55 | 1149.85 |
|  | m04 | 1530.70 | 1739.70 | 1600.35 | 1093.85 |
|  | m05 | 1541.85 | 1490.35 | 1359.25 | 1111.95 |
|  | m06 | 1564.95 | 1536.25 | 938.30 | 1024.65 |
|  | m07 | 1505.55 | 1264.00 | 1344.65 | 1050.40 |
|  | m08 | 1319.35 | 1118.15 | 1214.25 | 961.70 |
|  | m09 | 1188.65 | 1134.50 | 1310.80 | 1060.90 |
|  | m10 | 1229.30 | 1056.10 | 1472.60 | 1193.30 |
| p030 | m00 | 925.80 | 1026.80 | 1637.35 | 1690.65 |
|  | m01 | 949.35 | 981.35 | 1515.65 | 2107.90 |
|  | m02 | 954.15 | 948.65 | 2035.40 | 1736.25 |
|  | m03 | 1207.25 | 1182.70 | 1494.30 | 1946.95 |
|  | m04 | 1136.65 | 1045.80 | 2716.15 | 1388.35 |
|  | m05 | 1303.60 | 1139.90 | 1652.20 | 1669.00 |
|  | m06 | 1150.25 | 1304.00 | 1981.45 | 2355.70 |
|  | m07 | 1100.25 | 948.00 | 1994.75 | 1347.40 |
|  | m08 | 1061.60 | 1062.35 | 2125.40 | 1896.60 |
|  | m09 | 1223.15 | 914.55 | 1738.40 | 1587.55 |
|  | m10 | 955.30 | 888.75 | 1702.30 | 1624.30 |

In [28]:

```
ident_rt_between_df = ident_rt_df.unstack('grade')
rev_ident_rt_between_df = pd.concat([ident_rt_between_df[0:13], ident_rt_between_df[14:] ])
```

In [29]:

```
rev_ident_rt_between_df
```

Out[29]:

|  | fearLOW | | | | | | | | | | | angerLOW | | | | | | | | | | | fearUP | | | | | | | | | | | angerUP | | | | | | | | | | |
| --- | --- | --- | --- | --- | --- | --- | --- | --- | --- | --- | --- | --- | --- | --- | --- | --- | --- | --- | --- | --- | --- | --- | --- | --- | --- | --- | --- | --- | --- | --- | --- | --- | --- | --- | --- | --- | --- | --- | --- | --- | --- | --- | --- | --- |
| grade | m00 | m01 | m02 | m03 | m04 | m05 | m06 | m07 | m08 | m09 | m10 | m00 | m01 | m02 | m03 | m04 | m05 | m06 | m07 | m08 | m09 | m10 | m00 | m01 | m02 | m03 | m04 | m05 | m06 | m07 | m08 | m09 | m10 | m00 | m01 | m02 | m03 | m04 | m05 | m06 | m07 | m08 | m09 | m10 |
| p |  |  |  |  |  |  |  |  |  |  |  |  |  |  |  |  |  |  |  |  |  |  |  |  |  |  |  |  |  |  |  |  |  |  |  |  |  |  |  |  |  |  |  |  |
| p001 | 1078.60 | 1120.05 | 1129.15 | 1302.00 | 959.05 | 1523.25 | 1279.25 | 1210.80 | 1241.20 | 999.15 | 983.20 | 1029.60 | 1116.40 | 1113.45 | 931.40 | 1077.65 | 1298.45 | 1281.30 | 1145.20 | 1108.95 | 1091.20 | 929.05 | 1005.90 | 962.45 | 971.30 | 936.05 | 1010.75 | 890.25 | 894.05 | 882.00 | 990.30 | 971.20 | 866.80 | 1006.90 | 1138.70 | 961.80 | 858.10 | 816.70 | 898.75 | 999.50 | 799.85 | 834.70 | 805.60 | 811.30 |
| p002 | 1000.15 | 1389.15 | 1116.60 | 931.25 | 1130.30 | 1592.30 | 984.70 | 902.75 | 983.50 | 900.25 | 863.15 | 1156.10 | 919.75 | 963.85 | 909.05 | 1184.35 | 1067.70 | 992.50 | 1163.45 | 1011.25 | 969.40 | 937.85 | 1620.65 | 1677.60 | 1242.90 | 1631.15 | 2152.30 | 2043.05 | 2072.15 | 1501.85 | 1596.55 | 2052.60 | 1391.35 | 1551.10 | 1584.45 | 1584.40 | 1343.55 | 1630.40 | 1372.55 | 1250.50 | 1442.50 | 1259.40 | 1503.10 | 1427.20 |
| p003 | 1990.95 | 1552.65 | 2322.00 | 2282.40 | 2116.40 | 2546.90 | 2603.35 | 2168.45 | 1924.30 | 1566.45 | 1539.95 | 1882.00 | 2270.60 | 2471.15 | 2556.60 | 2022.25 | 2408.50 | 2049.15 | 2808.65 | 1416.55 | 1624.35 | 1336.25 | 1555.40 | 1772.95 | 1651.85 | 1969.75 | 1907.90 | 1594.05 | 1924.05 | 1868.55 | 1519.20 | 2363.30 | 2462.70 | 1972.50 | 1675.95 | 1530.30 | 1648.40 | 1663.85 | 1806.55 | 1538.65 | 1620.15 | 1361.30 | 1873.35 | 1774.90 |
| p004 | 1055.75 | 994.15 | 1081.90 | 1074.65 | 1219.90 | 1113.75 | 1015.45 | 1085.80 | 1057.30 | 895.00 | 929.20 | 1019.55 | 1079.25 | 949.75 | 1182.35 | 1110.25 | 1224.40 | 1017.20 | 853.45 | 1050.10 | 956.35 | 929.40 | 1142.25 | 1099.35 | 1200.70 | 1295.05 | 1303.40 | 1179.50 | 1227.20 | 1095.65 | 1065.00 | 995.25 | 1093.30 | 1000.35 | 1108.80 | 1139.75 | 904.80 | 1076.60 | 1180.40 | 1047.25 | 1047.10 | 975.60 | 1124.75 | 1040.20 |
| p005 | 1127.00 | 1198.35 | 1000.90 | 1039.40 | 1873.35 | 1568.85 | 1619.05 | 1771.25 | 1158.10 | 1305.90 | 980.20 | 1092.05 | 937.30 | 1311.80 | 1098.75 | 1050.85 | 1603.35 | 1315.20 | 1026.00 | 1129.70 | 887.85 | 921.75 | 998.80 | 1141.05 | 1033.70 | 1067.35 | 977.30 | 1150.55 | 1023.90 | 1329.55 | 1214.45 | 1033.55 | 1132.20 | 982.00 | 971.65 | 886.65 | 900.65 | 885.70 | 911.40 | 899.25 | 856.00 | 874.65 | 946.80 | 911.05 |
| p006 | 768.65 | 824.20 | 815.90 | 766.85 | 764.95 | 850.00 | 751.80 | 864.55 | 775.90 | 811.95 | 759.20 | 817.80 | 865.50 | 914.25 | 1006.35 | 858.55 | 829.90 | 819.80 | 750.00 | 789.45 | 878.65 | 878.00 | 739.90 | 1025.75 | 855.10 | 811.15 | 786.95 | 827.45 | 817.50 | 850.65 | 996.80 | 899.25 | 891.05 | 980.10 | 899.45 | 802.25 | 719.15 | 901.25 | 770.85 | 803.25 | 783.90 | 927.20 | 726.00 | 926.90 |
| p007 | 865.20 | 705.50 | 748.45 | 770.15 | 755.85 | 836.20 | 882.25 | 783.35 | 798.80 | 848.05 | 958.75 | 788.60 | 791.35 | 653.35 | 756.60 | 903.35 | 805.75 | 1053.50 | 861.40 | 766.95 | 751.20 | 761.85 | 767.25 | 855.15 | 700.55 | 627.55 | 780.35 | 776.60 | 745.25 | 742.35 | 711.65 | 770.80 | 735.60 | 812.35 | 712.65 | 770.20 | 702.25 | 784.55 | 746.80 | 728.45 | 743.20 | 783.35 | 722.20 | 659.85 |
| p008 | 1103.70 | 1016.75 | 1400.75 | 1364.75 | 1263.10 | 1332.50 | 1160.95 | 1235.40 | 1075.95 | 1097.45 | 1152.25 | 1140.05 | 1040.35 | 995.90 | 1039.05 | 1287.75 | 1083.25 | 1080.15 | 1228.45 | 1052.50 | 1115.40 | 1080.65 | 1359.65 | 1035.50 | 1009.10 | 1237.15 | 1307.15 | 1103.25 | 1171.15 | 1269.20 | 1305.95 | 1223.20 | 1336.40 | 1210.95 | 1226.40 | 1344.05 | 1065.75 | 1159.10 | 1144.25 | 1035.85 | 1053.95 | 1105.60 | 1172.40 | 1202.65 |
| p009 | 1403.60 | 1423.50 | 1365.15 | 1700.35 | 1384.95 | 1596.05 | 1459.15 | 1448.55 | 1471.40 | 1360.75 | 1353.55 | 1657.50 | 1690.75 | 1255.40 | 1412.30 | 1411.20 | 1436.35 | 1347.75 | 2095.80 | 1263.30 | 1356.80 | 1411.80 | 1359.55 | 1249.70 | 1124.10 | 1089.35 | 1474.95 | 1201.65 | 1119.20 | 1144.95 | 1006.40 | 1108.55 | 1403.50 | 1089.55 | 979.75 | 1273.40 | 1179.15 | 979.65 | 988.20 | 1175.90 | 1300.55 | 1169.25 | 1228.50 | 1057.30 |
| p010 | 1029.85 | 1099.70 | 957.15 | 1073.80 | 1059.70 | 1269.50 | 1278.95 | 1104.55 | 1102.80 | 1141.70 | 1196.50 | 916.65 | 1099.95 | 959.35 | 1019.80 | 956.25 | 1276.45 | 1115.35 | 1034.50 | 1040.70 | 1077.65 | 1074.35 | 1063.15 | 1091.80 | 1017.20 | 895.55 | 914.95 | 1148.05 | 1050.35 | 979.55 | 1043.05 | 1069.75 | 1058.40 | 1157.35 | 1075.45 | 1126.35 | 995.55 | 1064.90 | 1026.45 | 1021.60 | 1014.65 | 1053.05 | 902.60 | 1032.90 |
| p011 | 1227.40 | 1139.25 | 1212.95 | 1090.80 | 1893.35 | 1422.05 | 1277.95 | 1205.90 | 1235.30 | 1015.05 | 911.20 | 1135.15 | 992.10 | 1056.35 | 1227.25 | 1517.75 | 1216.10 | 2163.50 | 1230.05 | 1114.95 | 967.20 | 851.15 | 1138.10 | 944.45 | 1036.90 | 1340.20 | 1355.25 | 1145.80 | 1130.65 | 1032.55 | 1234.55 | 864.75 | 930.40 | 921.35 | 1030.30 | 1008.65 | 1150.40 | 1026.10 | 1104.60 | 1013.90 | 1024.65 | 948.15 | 856.10 | 946.65 |
| p012 | 907.55 | 971.15 | 915.20 | 1002.75 | 1005.65 | 1175.45 | 1051.35 | 1149.30 | 1059.25 | 1023.75 | 871.20 | 1007.25 | 992.65 | 944.05 | 1150.10 | 993.90 | 1352.30 | 1218.35 | 1309.05 | 988.80 | 853.30 | 886.15 | 1128.50 | 1220.10 | 1506.40 | 1080.45 | 1282.65 | 1238.80 | 1308.10 | 1229.15 | 1257.05 | 1429.85 | 1409.30 | 1611.60 | 1643.20 | 1480.60 | 1479.15 | 1580.45 | 1204.40 | 1290.55 | 1158.60 | 1110.25 | 1184.70 | 1380.70 |
| p013 | 989.60 | 824.65 | 794.35 | 821.00 | 843.25 | 1066.95 | 963.20 | 979.55 | 944.25 | 1160.25 | 1129.80 | 887.45 | 853.25 | 862.95 | 1011.25 | 821.50 | 970.80 | 813.10 | 1015.40 | 899.40 | 942.50 | 960.40 | 896.75 | 707.20 | 1013.00 | 755.20 | 827.30 | 707.50 | 756.85 | 795.85 | 808.10 | 876.50 | 710.65 | 816.30 | 807.25 | 778.50 | 683.10 | 811.45 | 735.85 | 787.25 | 682.45 | 789.60 | 699.60 | 763.15 |
| p015 | 825.05 | 748.15 | 809.45 | 782.80 | 765.50 | 796.85 | 878.50 | 816.05 | 836.80 | 719.30 | 729.25 | 775.30 | 751.55 | 739.75 | 741.10 | 766.10 | 847.05 | 720.30 | 746.00 | 762.85 | 724.00 | 739.35 | 757.90 | 765.50 | 734.85 | 712.30 | 763.05 | 709.90 | 827.60 | 654.00 | 657.00 | 691.05 | 638.85 | 742.95 | 784.20 | 784.95 | 690.95 | 749.40 | 855.75 | 734.45 | 730.35 | 679.85 | 666.20 | 631.95 |
| p016 | 1161.75 | 1112.35 | 1276.10 | 1170.75 | 1134.60 | 1404.80 | 1345.70 | 1238.25 | 2157.10 | 1044.25 | 1202.55 | 1011.45 | 1081.20 | 1196.80 | 1529.75 | 1221.55 | 1425.20 | 1461.85 | 1333.25 | 1643.50 | 1266.30 | 1264.15 | 1657.90 | 2142.80 | 1699.05 | 1624.90 | 1563.05 | 1772.10 | 1476.60 | 1683.70 | 1585.80 | 1594.00 | 1360.00 | 1756.15 | 1666.85 | 1709.95 | 1395.85 | 1375.70 | 1403.50 | 2208.55 | 1390.20 | 1500.70 | 1445.00 | 1378.25 |
| p017 | 1028.10 | 1140.90 | 1003.20 | 939.20 | 950.95 | 982.45 | 955.95 | 967.30 | 992.25 | 959.15 | 835.20 | 1049.95 | 987.85 | 1094.90 | 1190.45 | 978.05 | 1103.15 | 896.40 | 1050.60 | 906.20 | 999.75 | 863.55 | 906.45 | 933.40 | 803.60 | 802.65 | 813.60 | 853.75 | 869.00 | 740.55 | 711.25 | 875.05 | 800.10 | 839.30 | 875.10 | 888.25 | 809.55 | 787.60 | 802.55 | 811.60 | 801.85 | 755.85 | 825.75 | 851.45 |
| p018 | 1197.55 | 1178.30 | 1306.35 | 1395.60 | 1295.45 | 1459.95 | 1517.55 | 1436.80 | 1279.25 | 1276.50 | 1326.65 | 1148.60 | 1333.05 | 1480.75 | 1176.95 | 1316.30 | 1716.40 | 1668.20 | 1737.70 | 1698.60 | 1186.10 | 1150.05 | 1623.70 | 2285.20 | 1715.65 | 1576.20 | 1603.30 | 1624.60 | 1600.65 | 1470.20 | 1635.25 | 1670.40 | 1537.15 | 1515.25 | 1387.60 | 1418.90 | 1451.20 | 1344.40 | 1433.20 | 1667.15 | 1283.10 | 1323.25 | 1617.90 | 1524.30 |
| p019 | 818.05 | 932.00 | 987.25 | 836.95 | 830.20 | 899.25 | 1016.30 | 831.95 | 860.65 | 774.30 | 771.25 | 865.75 | 842.15 | 1020.40 | 916.45 | 988.30 | 983.10 | 970.80 | 914.55 | 945.20 | 852.15 | 751.35 | 1098.25 | 956.10 | 937.65 | 993.10 | 945.80 | 1174.65 | 957.65 | 945.40 | 832.90 | 790.00 | 978.30 | 919.85 | 954.95 | 900.85 | 888.35 | 915.50 | 864.35 | 892.05 | 835.15 | 754.90 | 776.05 | 944.75 |
| p020 | 1268.40 | 1107.35 | 1121.30 | 1247.20 | 1123.50 | 1123.25 | 1035.00 | 1297.40 | 1059.15 | 1093.75 | 1051.75 | 980.20 | 1210.55 | 1229.80 | 1336.15 | 1240.20 | 1205.85 | 1280.40 | 1231.75 | 1283.40 | 1075.75 | 1166.50 | 1405.70 | 1447.55 | 1210.75 | 1179.65 | 1467.20 | 1489.60 | 1299.15 | 1311.45 | 1242.75 | 1319.90 | 1061.55 | 1390.80 | 1207.20 | 1372.85 | 1312.50 | 1244.80 | 1412.25 | 1638.50 | 1402.05 | 1565.95 | 1307.15 | 1302.10 |
| p021 | 916.30 | 827.90 | 856.05 | 871.70 | 1034.90 | 1115.65 | 1199.80 | 1244.70 | 898.50 | 888.85 | 911.05 | 906.70 | 1072.55 | 1279.65 | 1051.10 | 862.10 | 998.45 | 1059.95 | 920.50 | 951.30 | 865.25 | 792.25 | 881.55 | 986.25 | 1126.55 | 1026.50 | 961.50 | 978.95 | 1001.75 | 1032.20 | 1007.10 | 1016.95 | 1196.75 | 1325.65 | 968.15 | 953.00 | 1039.95 | 892.50 | 1122.50 | 719.95 | 981.70 | 763.10 | 786.05 | 834.90 |
| p022 | 756.15 | 719.10 | 857.00 | 716.15 | 755.90 | 717.95 | 797.05 | 774.60 | 716.00 | 612.60 | 684.80 | 839.50 | 759.30 | 660.15 | 762.60 | 710.90 | 795.60 | 670.30 | 751.90 | 742.75 | 731.25 | 680.95 | 394.45 | 447.25 | 500.70 | 397.00 | 271.45 | 442.20 | 450.25 | 329.60 | 291.25 | 396.40 | 426.00 | 470.90 | 571.20 | 329.00 | 504.35 | 516.10 | 423.20 | 431.80 | 304.95 | 429.65 | 363.50 | 452.80 |
| p023 | 972.15 | 915.80 | 1080.05 | 1126.00 | 1120.65 | 1106.35 | 1442.80 | 1416.10 | 1275.25 | 1314.45 | 1031.15 | 1016.90 | 1056.00 | 1217.40 | 1094.05 | 1203.10 | 1248.35 | 1289.40 | 1131.60 | 1108.10 | 1353.15 | 1096.80 | 823.45 | 842.20 | 952.10 | 975.10 | 1174.95 | 1410.45 | 967.30 | 958.60 | 1155.10 | 1439.25 | 1145.40 | 1060.70 | 896.60 | 1004.55 | 1045.40 | 1148.20 | 979.05 | 1199.20 | 1052.40 | 915.65 | 992.60 | 852.45 |
| p024 | 1079.30 | 1058.15 | 1062.30 | 1311.30 | 1172.75 | 1540.55 | 1289.40 | 1225.70 | 1223.15 | 1033.55 | 984.10 | 1080.10 | 1267.20 | 1288.35 | 1241.10 | 1258.20 | 1257.65 | 1293.30 | 1076.20 | 1133.05 | 1027.00 | 1088.35 | 903.20 | 989.00 | 1201.15 | 1340.45 | 1365.40 | 1059.45 | 1077.80 | 1030.80 | 1213.40 | 1072.30 | 1396.50 | 1809.45 | 1496.20 | 1201.25 | 1102.95 | 1163.50 | 1345.20 | 1382.80 | 1223.40 | 1346.30 | 1160.05 | 1178.40 |
| p025 | 1327.15 | 1403.60 | 1479.35 | 1488.55 | 1643.00 | 1548.15 | 1453.10 | 1700.55 | 1540.20 | 1472.20 | 1543.20 | 1438.40 | 1457.35 | 1520.05 | 1527.25 | 1238.30 | 1388.85 | 1399.15 | 1450.75 | 1453.55 | 1545.60 | 1303.75 | 1285.50 | 1439.45 | 1119.20 | 1324.50 | 1335.25 | 1430.25 | 1292.50 | 1463.40 | 1082.75 | 1327.90 | 1155.70 | 1414.40 | 1455.25 | 1387.60 | 1748.40 | 1346.15 | 1242.40 | 1218.50 | 1171.15 | 1272.30 | 1125.00 | 1238.45 |
| p026 | 1392.55 | 1371.75 | 1473.95 | 1485.25 | 1459.50 | 2056.45 | 1631.65 | 1713.75 | 1478.95 | 1328.10 | 1496.70 | 1438.75 | 1589.45 | 1311.50 | 1320.45 | 1621.40 | 1922.50 | 1852.65 | 1422.65 | 1096.35 | 1175.40 | 971.70 | 1725.75 | 1902.60 | 1583.25 | 2179.10 | 1439.10 | 1997.25 | 1821.25 | 1292.75 | 1936.90 | 2079.75 | 1663.10 | 1976.70 | 1412.55 | 1543.75 | 1840.55 | 1371.25 | 1192.15 | 1229.60 | 1147.25 | 1295.40 | 1112.30 | 1112.90 |
| p027 | 755.60 | 801.20 | 787.20 | 852.15 | 903.10 | 802.50 | 799.45 | 996.45 | 757.55 | 876.95 | 831.50 | 885.20 | 793.70 | 783.00 | 859.20 | 839.25 | 758.35 | 744.60 | 800.50 | 798.10 | 853.40 | 743.80 | 831.75 | 891.30 | 805.90 | 780.55 | 751.25 | 653.65 | 1215.80 | 777.45 | 951.65 | 838.10 | 839.50 | 1019.70 | 812.40 | 852.55 | 851.75 | 886.55 | 729.35 | 700.45 | 831.30 | 786.85 | 911.80 | 814.10 |
| p028 | 1316.70 | 1271.10 | 1324.60 | 1690.85 | 1838.10 | 1872.45 | 1659.25 | 1216.50 | 980.05 | 992.25 | 887.40 | 1336.15 | 1636.25 | 1583.75 | 1451.40 | 1760.15 | 1463.75 | 1541.40 | 1011.20 | 971.50 | 1066.85 | 794.95 | 1732.30 | 2589.20 | 1832.95 | 2243.25 | 2886.20 | 3539.15 | 2184.90 | 3105.80 | 1751.10 | 1735.45 | 1837.50 | 2845.25 | 2090.80 | 2479.45 | 2774.15 | 1566.50 | 1719.20 | 1144.15 | 1204.15 | 1299.10 | 1110.35 | 989.20 |
| p029 | 1265.45 | 1266.60 | 1328.75 | 1173.80 | 1530.70 | 1541.85 | 1564.95 | 1505.55 | 1319.35 | 1188.65 | 1229.30 | 1125.20 | 1302.80 | 1284.25 | 1399.95 | 1739.70 | 1490.35 | 1536.25 | 1264.00 | 1118.15 | 1134.50 | 1056.10 | 1594.10 | 1352.30 | 1269.15 | 1101.55 | 1600.35 | 1359.25 | 938.30 | 1344.65 | 1214.25 | 1310.80 | 1472.60 | 1280.20 | 1389.60 | 1063.70 | 1149.85 | 1093.85 | 1111.95 | 1024.65 | 1050.40 | 961.70 | 1060.90 | 1193.30 |
| p030 | 925.80 | 949.35 | 954.15 | 1207.25 | 1136.65 | 1303.60 | 1150.25 | 1100.25 | 1061.60 | 1223.15 | 955.30 | 1026.80 | 981.35 | 948.65 | 1182.70 | 1045.80 | 1139.90 | 1304.00 | 948.00 | 1062.35 | 914.55 | 888.75 | 1637.35 | 1515.65 | 2035.40 | 1494.30 | 2716.15 | 1652.20 | 1981.45 | 1994.75 | 2125.40 | 1738.40 | 1702.30 | 1690.65 | 2107.90 | 1736.25 | 1946.95 | 1388.35 | 1669.00 | 2355.70 | 1347.40 | 1896.60 | 1587.55 | 1624.30 |

In [31]:

```
for cond in rev_ident_rt_between_df.columns.levels[0]:
    errorbar(range(len(rev_ident_rt_between_df[cond].mean())),
             rev_ident_rt_between_df[cond].mean(),
             rev_ident_rt_between_df[cond].std(),
             label=cond)
legend(loc='best')
show()
```

## Save df to file¶

In [32]:

```
savePandas(my_folder,ident_rt_between_df,'Exp2RtMedianAll.txt')
savePandas(my_folder,rev_ident_rt_between_df,'Exp2RtMedian.txt')
```
