# Supplementary material for: Categorical Perception of Fear and Anger Expressions in Whole, Masked and Composite Faces
Source: PLoS One. 2015 Aug 11;10(8):e0134790. doi: 10.1371/journal.pone.0134790 (PMC4532458; doi:10.1371/journal.pone.0134790)
Supplement: S3 Code — (HTML) [file pone.0134790.s004.html]

SupplementS3\_MainResults


# Supplement S3 of

## Categorical Perception of Fear and Anger Expressions in Whole, Masked and Composite Faces.

# Main Analysis of Raw Data¶

- The main results for experiments 1 and 2 are plotted on a single-subject and group level
- Descriptive Statistics for all morphing grades and conditions are provided
- inferential statistics (parametric and non-parametric) are computed for the main analyses

## Import Libraries¶

In [1]:

```
import fnmatch # filename matching
import os # navigating directories

import numpy as np

import pandas as pd
pd.set_option('max_columns', 100)
pd.set_option('max_rows', 1000)

from scipy import stats

import seaborn as sns
sns.set_style("ticks")

%pylab inline
```

```
Populating the interactive namespace from numpy and matplotlib
```

## Get List of previously saved df's (pickle)¶

In [2]:

```
def get_list(where, what):
    os.chdir(where)
    fileList = []
    for fileName in os.listdir(where):
        if fnmatch.fnmatch(fileName, what):
            fileList.append(fileName)
    return fileList
```

In [3]:

```
pList = get_list('./data/','*.txt')
pList.sort()
for i in range(len(pList)):
    print i,':\t',pList[i]
```

```
0 :	Exp1AvgResults.txt
1 :	Exp1AvgResultsAll.txt
2 :	Exp1Pt1MainResults.txt
3 :	Exp1Pt1ResultsAll.txt
4 :	Exp1Pt2MainResults.txt
5 :	Exp1Pt2ResultsAll.txt
6 :	Exp1RtMedian.txt
7 :	Exp1RtMedianAll.txt
8 :	Exp2AvgResults.txt
9 :	Exp2AvgResultsAll.txt
10 :	Exp2Pt1Results.txt
11 :	Exp2Pt1ResultsAll.txt
12 :	Exp2Pt2Results.txt
13 :	Exp2Pt2ResultsAll.txt
14 :	Exp2RtMedian.txt
15 :	Exp2RtMedianAll.txt
16 :	exp1Pt1Logistic.txt
17 :	exp1Pt2Logistic.txt
18 :	exp1fittable.txt
19 :	exp1pt1Logistic.txt
20 :	exp1pt2Logistic.txt
21 :	exp2Pt1Logistic.txt
22 :	exp2Pt2Logistic.txt
23 :	exp2fittable.txt
24 :	exp2pt1Logistic.txt
25 :	exp2pt2Logistic.txt
```

# Experiment 1¶

## Load a df¶

In [4]:

```
def load_this(csv):
    df = pd.read_csv(csv,
                     index_col=[0],
                     header=[0,1])
    return df
```

In [5]:

```
exp1Table = load_this(pList[0])
```

In [6]:

```
exp1Table
```

Out[6]:

|  | whole | | | | | | | | | | | mouth | | | | | | | | | | | eyes | | | | | | | | | | |
| --- | --- | --- | --- | --- | --- | --- | --- | --- | --- | --- | --- | --- | --- | --- | --- | --- | --- | --- | --- | --- | --- | --- | --- | --- | --- | --- | --- | --- | --- | --- | --- | --- | --- |
| grade | m00 | m01 | m02 | m03 | m04 | m05 | m06 | m07 | m08 | m09 | m10 | m00 | m01 | m02 | m03 | m04 | m05 | m06 | m07 | m08 | m09 | m10 | m00 | m01 | m02 | m03 | m04 | m05 | m06 | m07 | m08 | m09 | m10 |
| p |  |  |  |  |  |  |  |  |  |  |  |  |  |  |  |  |  |  |  |  |  |  |  |  |  |  |  |  |  |  |  |  |  |
| p001 | 12.5 | 17.5 | 25.0 | 20.0 | 27.5 | 60.0 | 67.5 | 95.0 | 97.5 | 100.0 | 100.0 | 32.5 | 42.5 | 40.0 | 52.5 | 57.5 | 60.0 | 75.0 | 80.0 | 85.0 | 82.5 | 82.5 | 12.5 | 5.0 | 17.5 | 22.5 | 42.5 | 42.5 | 65.0 | 85.0 | 90.0 | 97.5 | 97.5 |
| p002 | 15.0 | 0.0 | 5.0 | 15.0 | 22.5 | 37.5 | 57.5 | 70.0 | 87.5 | 90.0 | 97.5 | 17.5 | 20.0 | 30.0 | 32.5 | 40.0 | 55.0 | 75.0 | 75.0 | 85.0 | 82.5 | 90.0 | 22.5 | 25.0 | 27.5 | 35.0 | 45.0 | 60.0 | 72.5 | 82.5 | 90.0 | 92.5 | 90.0 |
| p003 | 12.5 | 15.0 | 15.0 | 20.0 | 47.5 | 67.5 | 77.5 | 97.5 | 100.0 | 100.0 | 100.0 | 25.0 | 25.0 | 37.5 | 42.5 | 60.0 | 52.5 | 72.5 | 65.0 | 65.0 | 77.5 | 85.0 | 30.0 | 37.5 | 45.0 | 62.5 | 70.0 | 75.0 | 87.5 | 85.0 | 85.0 | 97.5 | 97.5 |
| p004 | 5.0 | 7.5 | 20.0 | 12.5 | 32.5 | 32.5 | 60.0 | 80.0 | 87.5 | 85.0 | 87.5 | 20.0 | 30.0 | 35.0 | 22.5 | 40.0 | 45.0 | 45.0 | 52.5 | 65.0 | 57.5 | 57.5 | 22.5 | 27.5 | 37.5 | 27.5 | 37.5 | 57.5 | 67.5 | 57.5 | 77.5 | 80.0 | 87.5 |
| p005 | 0.0 | 0.0 | 10.0 | 15.0 | 20.0 | 22.5 | 45.0 | 60.0 | 82.5 | 77.5 | 85.0 | 20.0 | 20.0 | 30.0 | 27.5 | 35.0 | 27.5 | 45.0 | 40.0 | 57.5 | 65.0 | 55.0 | 5.0 | 2.5 | 15.0 | 27.5 | 25.0 | 35.0 | 52.5 | 60.0 | 82.5 | 87.5 | 87.5 |
| p006 | 5.0 | 7.5 | 2.5 | 7.5 | 17.5 | 45.0 | 77.5 | 95.0 | 95.0 | 100.0 | 100.0 | 17.5 | 17.5 | 22.5 | 35.0 | 37.5 | 47.5 | 62.5 | 67.5 | 75.0 | 85.0 | 87.5 | 5.0 | 7.5 | 7.5 | 5.0 | 35.0 | 40.0 | 70.0 | 82.5 | 97.5 | 97.5 | 100.0 |
| p007 | 12.5 | 10.0 | 15.0 | 22.5 | 15.0 | 32.5 | 60.0 | 80.0 | 90.0 | 95.0 | 97.5 | 37.5 | 55.0 | 57.5 | 45.0 | 55.0 | 65.0 | 72.5 | 92.5 | 95.0 | 87.5 | 87.5 | 10.0 | 10.0 | 20.0 | 22.5 | 25.0 | 45.0 | 60.0 | 70.0 | 85.0 | 90.0 | 95.0 |
| p008 | 17.5 | 27.5 | 25.0 | 30.0 | 42.5 | 60.0 | 62.5 | 90.0 | 95.0 | 95.0 | 100.0 | 60.0 | 67.5 | 65.0 | 62.5 | 67.5 | 72.5 | 80.0 | 90.0 | 95.0 | 92.5 | 92.5 | 15.0 | 15.0 | 30.0 | 32.5 | 30.0 | 42.5 | 65.0 | 80.0 | 97.5 | 92.5 | 97.5 |
| p009 | 5.0 | 2.5 | 0.0 | 5.0 | 25.0 | 35.0 | 62.5 | 80.0 | 95.0 | 97.5 | 100.0 | 10.0 | 7.5 | 20.0 | 20.0 | 27.5 | 62.5 | 65.0 | 80.0 | 85.0 | 85.0 | 87.5 | 2.5 | 2.5 | 5.0 | 15.0 | 22.5 | 45.0 | 55.0 | 87.5 | 85.0 | 95.0 | 97.5 |
| p010 | 15.0 | 22.5 | 30.0 | 27.5 | 25.0 | 60.0 | 70.0 | 85.0 | 97.5 | 95.0 | 100.0 | 35.0 | 37.5 | 37.5 | 50.0 | 55.0 | 60.0 | 70.0 | 70.0 | 77.5 | 70.0 | 85.0 | 17.5 | 35.0 | 37.5 | 37.5 | 40.0 | 40.0 | 52.5 | 70.0 | 87.5 | 82.5 | 85.0 |
| p011 | 2.5 | 10.0 | 10.0 | 7.5 | 20.0 | 40.0 | 77.5 | 90.0 | 92.5 | 95.0 | 100.0 | 32.5 | 25.0 | 42.5 | 45.0 | 57.5 | 67.5 | 82.5 | 92.5 | 100.0 | 90.0 | 95.0 | 5.0 | 5.0 | 7.5 | 10.0 | 27.5 | 25.0 | 45.0 | 80.0 | 90.0 | 100.0 | 97.5 |
| p012 | 12.5 | 7.5 | 25.0 | 37.5 | 47.5 | 67.5 | 85.0 | 100.0 | 95.0 | 100.0 | 95.0 | 37.5 | 45.0 | 45.0 | 45.0 | 57.5 | 62.5 | 85.0 | 85.0 | 92.5 | 95.0 | 87.5 | 25.0 | 27.5 | 32.5 | 25.0 | 45.0 | 55.0 | 62.5 | 70.0 | 87.5 | 90.0 | 95.0 |
| p013 | 2.5 | 2.5 | 2.5 | 12.5 | 22.5 | 27.5 | 62.5 | 72.5 | 90.0 | 92.5 | 95.0 | 37.5 | 32.5 | 45.0 | 45.0 | 60.0 | 65.0 | 70.0 | 77.5 | 92.5 | 80.0 | 80.0 | 0.0 | 12.5 | 15.0 | 17.5 | 25.0 | 35.0 | 57.5 | 77.5 | 92.5 | 87.5 | 97.5 |
| p014 | 5.0 | 2.5 | 10.0 | 10.0 | 22.5 | 50.0 | 62.5 | 82.5 | 97.5 | 97.5 | 100.0 | 10.0 | 2.5 | 30.0 | 12.5 | 22.5 | 25.0 | 57.5 | 65.0 | 75.0 | 75.0 | 80.0 | 5.0 | 7.5 | 10.0 | 25.0 | 30.0 | 50.0 | 62.5 | 80.0 | 95.0 | 97.5 | 95.0 |
| p015 | 7.5 | 5.0 | 10.0 | 17.5 | 35.0 | 37.5 | 67.5 | 82.5 | 95.0 | 97.5 | 100.0 | 27.5 | 22.5 | 30.0 | 35.0 | 37.5 | 42.5 | 50.0 | 55.0 | 55.0 | 55.0 | 62.5 | 12.5 | 7.5 | 7.5 | 35.0 | 22.5 | 42.5 | 52.5 | 65.0 | 75.0 | 90.0 | 90.0 |
| p016 | 10.0 | 5.0 | 10.0 | 17.5 | 27.5 | 47.5 | 62.5 | 80.0 | 90.0 | 100.0 | 100.0 | 37.5 | 45.0 | 50.0 | 52.5 | 62.5 | 80.0 | 80.0 | 85.0 | 97.5 | 95.0 | 97.5 | 5.0 | 15.0 | 17.5 | 17.5 | 22.5 | 42.5 | 62.5 | 82.5 | 95.0 | 95.0 | 97.5 |
| p019 | 5.0 | 2.5 | 10.0 | 7.5 | 22.5 | 52.5 | 77.5 | 95.0 | 97.5 | 100.0 | 100.0 | 35.0 | 30.0 | 37.5 | 40.0 | 47.5 | 47.5 | 72.5 | 85.0 | 82.5 | 82.5 | 92.5 | 7.5 | 5.0 | 15.0 | 22.5 | 30.0 | 47.5 | 72.5 | 90.0 | 95.0 | 97.5 | 100.0 |
| p020 | 27.5 | 27.5 | 32.5 | 35.0 | 40.0 | 60.0 | 72.5 | 75.0 | 87.5 | 90.0 | 87.5 | 20.0 | 40.0 | 20.0 | 40.0 | 40.0 | 37.5 | 40.0 | 37.5 | 40.0 | 47.5 | 40.0 | 27.5 | 35.0 | 42.5 | 45.0 | 52.5 | 65.0 | 67.5 | 77.5 | 90.0 | 85.0 | 87.5 |
| p021 | 2.5 | 2.5 | 5.0 | 10.0 | 17.5 | 32.5 | 45.0 | 70.0 | 85.0 | 95.0 | 100.0 | 17.5 | 27.5 | 27.5 | 30.0 | 42.5 | 52.5 | 52.5 | 55.0 | 65.0 | 82.5 | 75.0 | 2.5 | 5.0 | 5.0 | 5.0 | 7.5 | 20.0 | 25.0 | 45.0 | 60.0 | 87.5 | 87.5 |
| p022 | 5.0 | 5.0 | 10.0 | 17.5 | 32.5 | 47.5 | 60.0 | 77.5 | 95.0 | 100.0 | 100.0 | 22.5 | 20.0 | 22.5 | 30.0 | 40.0 | 57.5 | 72.5 | 75.0 | 80.0 | 90.0 | 90.0 | 5.0 | 7.5 | 12.5 | 15.0 | 35.0 | 45.0 | 57.5 | 77.5 | 87.5 | 100.0 | 95.0 |
| p023 | 7.5 | 2.5 | 12.5 | 20.0 | 32.5 | 52.5 | 75.0 | 90.0 | 97.5 | 100.0 | 100.0 | 20.0 | 20.0 | 15.0 | 25.0 | 37.5 | 42.5 | 55.0 | 67.5 | 70.0 | 75.0 | 80.0 | 15.0 | 17.5 | 7.5 | 25.0 | 32.5 | 50.0 | 67.5 | 85.0 | 92.5 | 95.0 | 100.0 |
| p024 | 10.0 | 17.5 | 15.0 | 20.0 | 27.5 | 55.0 | 80.0 | 92.5 | 97.5 | 97.5 | 100.0 | 25.0 | 30.0 | 27.5 | 37.5 | 30.0 | 40.0 | 55.0 | 67.5 | 72.5 | 72.5 | 80.0 | 12.5 | 10.0 | 20.0 | 30.0 | 35.0 | 45.0 | 72.5 | 82.5 | 95.0 | 97.5 | 97.5 |
| p025 | 20.0 | 17.5 | 27.5 | 22.5 | 42.5 | 50.0 | 60.0 | 70.0 | 75.0 | 90.0 | 95.0 | 45.0 | 42.5 | 37.5 | 42.5 | 50.0 | 47.5 | 45.0 | 57.5 | 55.0 | 60.0 | 70.0 | 17.5 | 22.5 | 22.5 | 32.5 | 32.5 | 42.5 | 40.0 | 72.5 | 90.0 | 90.0 | 85.0 |
| p026 | 7.5 | 7.5 | 5.0 | 7.5 | 22.5 | 47.5 | 70.0 | 87.5 | 97.5 | 100.0 | 100.0 | 20.0 | 30.0 | 17.5 | 30.0 | 35.0 | 40.0 | 52.5 | 50.0 | 67.5 | 70.0 | 72.5 | 15.0 | 25.0 | 22.5 | 30.0 | 40.0 | 52.5 | 67.5 | 80.0 | 87.5 | 87.5 | 97.5 |
| p027 | 7.5 | 17.5 | 20.0 | 30.0 | 32.5 | 50.0 | 75.0 | 92.5 | 92.5 | 100.0 | 100.0 | 20.0 | 35.0 | 37.5 | 52.5 | 60.0 | 70.0 | 85.0 | 85.0 | 82.5 | 90.0 | 85.0 | 17.5 | 27.5 | 30.0 | 50.0 | 55.0 | 62.5 | 70.0 | 87.5 | 87.5 | 97.5 | 100.0 |
| p028 | 7.5 | 2.5 | 2.5 | 15.0 | 25.0 | 27.5 | 55.0 | 70.0 | 85.0 | 97.5 | 97.5 | 32.5 | 25.0 | 37.5 | 32.5 | 42.5 | 55.0 | 72.5 | 67.5 | 75.0 | 80.0 | 85.0 | 2.5 | 10.0 | 10.0 | 17.5 | 22.5 | 22.5 | 42.5 | 67.5 | 85.0 | 90.0 | 90.0 |
| p029 | 0.0 | 5.0 | 5.0 | 7.5 | 22.5 | 25.0 | 65.0 | 67.5 | 82.5 | 90.0 | 95.0 | 12.5 | 15.0 | 20.0 | 17.5 | 25.0 | 35.0 | 52.5 | 57.5 | 67.5 | 77.5 | 70.0 | 2.5 | 5.0 | 10.0 | 12.5 | 17.5 | 30.0 | 50.0 | 72.5 | 82.5 | 90.0 | 87.5 |
| p030 | 35.0 | 37.5 | 40.0 | 52.5 | 45.0 | 65.0 | 85.0 | 85.0 | 100.0 | 95.0 | 100.0 | 62.5 | 55.0 | 57.5 | 65.0 | 70.0 | 67.5 | 70.0 | 82.5 | 80.0 | 100.0 | 85.0 | 32.5 | 45.0 | 40.0 | 55.0 | 60.0 | 60.0 | 72.5 | 72.5 | 75.0 | 87.5 | 87.5 |

## Table with Descriptive Statistics¶

In [7]:

```
exp1Table.describe()
```

Out[7]:

|  | whole | | | | | | | | | | | mouth | | | | | | | | | | | eyes | | | | | | | | | | |
| --- | --- | --- | --- | --- | --- | --- | --- | --- | --- | --- | --- | --- | --- | --- | --- | --- | --- | --- | --- | --- | --- | --- | --- | --- | --- | --- | --- | --- | --- | --- | --- | --- | --- |
|  | m00 | m01 | m02 | m03 | m04 | m05 | m06 | m07 | m08 | m09 | m10 | m00 | m01 | m02 | m03 | m04 | m05 | m06 | m07 | m08 | m09 | m10 | m00 | m01 | m02 | m03 | m04 | m05 | m06 | m07 | m08 | m09 | m10 |
| count | 28.000000 | 28.000000 | 28.000000 | 28.000000 | 28.000000 | 28.000000 | 28.000000 | 28.000000 | 28.000000 | 28.000000 | 28.000000 | 28.000000 | 28.000000 | 28.000000 | 28.000000 | 28.000000 | 28.000000 | 28.000000 | 28.000000 | 28.000000 | 28.000000 | 28.000000 | 28.000000 | 28.000000 | 28.000000 | 28.000000 | 28.000000 | 28.000000 | 28.000000 | 28.000000 | 28.000000 | 28.000000 | 28.000000 |
| mean | 9.821429 | 10.267857 | 14.285714 | 18.660714 | 29.017857 | 45.982143 | 67.053571 | 82.589286 | 92.142857 | 95.446429 | 97.589286 | 28.214286 | 30.892857 | 34.821429 | 38.125000 | 46.160714 | 52.946429 | 64.732143 | 70.000000 | 76.250000 | 78.660714 | 79.910714 | 12.589286 | 16.339286 | 20.357143 | 27.053571 | 34.464286 | 45.535714 | 60.535714 | 75.892857 | 87.142857 | 92.053571 | 93.660714 |
| std | 7.961963 | 9.606597 | 10.405152 | 10.918090 | 9.509724 | 13.356565 | 10.431735 | 10.485487 | 6.188186 | 5.402913 | 4.275368 | 13.156064 | 14.612499 | 12.709878 | 12.956927 | 13.184625 | 13.778230 | 13.460148 | 15.244914 | 14.585317 | 12.936493 | 13.219692 | 9.241666 | 12.180778 | 12.298643 | 14.027974 | 13.597337 | 12.844460 | 12.662959 | 10.256305 | 8.042546 | 5.359898 | 5.158692 |
| min | 0.000000 | 0.000000 | 0.000000 | 5.000000 | 15.000000 | 22.500000 | 45.000000 | 60.000000 | 75.000000 | 77.500000 | 85.000000 | 10.000000 | 2.500000 | 15.000000 | 12.500000 | 22.500000 | 25.000000 | 40.000000 | 37.500000 | 40.000000 | 47.500000 | 40.000000 | 0.000000 | 2.500000 | 5.000000 | 5.000000 | 7.500000 | 20.000000 | 25.000000 | 45.000000 | 60.000000 | 80.000000 | 85.000000 |
| 25% | 5.000000 | 2.500000 | 5.000000 | 10.000000 | 22.500000 | 34.375000 | 60.000000 | 74.375000 | 87.500000 | 94.375000 | 96.875000 | 20.000000 | 20.000000 | 26.250000 | 30.000000 | 37.500000 | 42.500000 | 52.500000 | 57.500000 | 66.875000 | 71.875000 | 74.375000 | 5.000000 | 6.875000 | 10.000000 | 17.500000 | 25.000000 | 40.000000 | 52.500000 | 70.000000 | 85.000000 | 87.500000 | 87.500000 |
| 50% | 7.500000 | 7.500000 | 10.000000 | 17.500000 | 26.250000 | 47.500000 | 66.250000 | 82.500000 | 95.000000 | 97.500000 | 100.000000 | 25.000000 | 30.000000 | 36.250000 | 38.750000 | 42.500000 | 53.750000 | 70.000000 | 68.750000 | 76.250000 | 81.250000 | 85.000000 | 12.500000 | 11.250000 | 17.500000 | 25.000000 | 32.500000 | 45.000000 | 62.500000 | 78.750000 | 87.500000 | 91.250000 | 95.000000 |
| 75% | 12.500000 | 17.500000 | 21.250000 | 22.500000 | 33.125000 | 56.250000 | 75.625000 | 90.625000 | 97.500000 | 100.000000 | 100.000000 | 35.625000 | 40.625000 | 40.625000 | 45.000000 | 57.500000 | 63.125000 | 73.125000 | 83.125000 | 85.000000 | 88.125000 | 87.500000 | 17.500000 | 25.625000 | 30.000000 | 33.125000 | 40.625000 | 53.125000 | 68.125000 | 82.500000 | 92.500000 | 97.500000 | 97.500000 |
| max | 35.000000 | 37.500000 | 40.000000 | 52.500000 | 47.500000 | 67.500000 | 85.000000 | 100.000000 | 100.000000 | 100.000000 | 100.000000 | 62.500000 | 67.500000 | 65.000000 | 65.000000 | 70.000000 | 80.000000 | 85.000000 | 92.500000 | 100.000000 | 100.000000 | 97.500000 | 32.500000 | 45.000000 | 45.000000 | 62.500000 | 70.000000 | 75.000000 | 87.500000 | 90.000000 | 97.500000 | 100.000000 | 100.000000 |

## Descriptives on Group Level¶

In [8]:

```
# import in-house module with plot design
import my_plots as oo_plt
```

In [9]:

```
#colors of choice
myPalette=['#4f5b66','#325da7','#ee4035']
```

In [10]:

```
exp1Plt = oo_plt.GroupErrBar(exp1Table,"experiment 1")

oo_plt.GroupErrBar.plot_setup(exp1Plt,select=[0,1,2],mycolors=myPalette,xlab='Morphing Grade (% Angry)\n')
oo_plt.GroupErrBar.plot_sum(exp1Plt,mode='mean')
```

```
The Created Object Has The Following Properties:
conditions: ['eyes', 'mouth', 'whole']
cases: ['p001', 'p002', 'p003', 'p004', 'p005', 'p006', 'p007', 'p008', 'p009', 'p010', 'p011', 'p012', 'p013', 'p014', 'p015', 'p016', 'p019', 'p020', 'p021', 'p022', 'p023', 'p024', 'p025', 'p026', 'p027', 'p028', 'p029', 'p030']
grades: ['m00', 'm01', 'm02', 'm03', 'm04', 'm05', 'm06', 'm07', 'm08', 'm09', 'm10']

Currently Selected Conditions: ['eyes', 'mouth', 'whole']
```

## Inferential Statistics¶

In [11]:

```
def inf_stats(df,conds,my_alpha):
    for c1 in conds:
        for c2 in conds:
            if c1 != c2:
                print c1,c2
                for m in df.columns.levels[1]:
                    t,p = stats.ttest_rel(df[c1][m],df[c2][m])
                    T,np = stats.wilcoxon(df[c1][m],df[c2][m])
                    if p < my_alpha and np < my_alpha:
                        sig = '*'
                    else:
                        sig = 'n.s.'
                    
                    print m,'\tt:',round(t,2),'p:',round(p,3),'\tT:',round(T,2),'np:',round(np,3), sig
```

Pairwise differences between all conditions for all morphing grades:

In [12]:

```
inf_stats(exp1Table,['whole','mouth','eyes'],0.05)
```

```
whole mouth
m00 	t: -9.23 p: 0.0 	T: 4.0 np: 0.0 *
m01 	t: -10.17 p: 0.0 	T: 0.0 np: 0.0 *
m02 	t: -8.76 p: 0.0 	T: 5.0 np: 0.0 *
m03 	t: -10.56 p: 0.0 	T: 0.0 np: 0.0 *
m04 	t: -7.16 p: 0.0 	T: 0.0 np: 0.0 *
m05 	t: -2.15 p: 0.04 	T: 105.0 np: 0.044 *
m06 	t: 0.86 p: 0.397 	T: 136.5 np: 0.484 n.s.
m07 	t: 4.84 p: 0.0 	T: 37.5 np: 0.0 *
m08 	t: 6.05 p: 0.0 	T: 18.0 np: 0.0 *
m09 	t: 7.79 p: 0.0 	T: 3.0 np: 0.0 *
m10 	t: 9.03 p: 0.0 	T: 0.0 np: 0.0 *
whole eyes
m00 	t: -2.42 p: 0.023 	T: 64.5 np: 0.042 *
m01 	t: -3.32 p: 0.003 	T: 52.0 np: 0.005 *
m02 	t: -3.84 p: 0.001 	T: 40.5 np: 0.001 *
m03 	t: -4.16 p: 0.0 	T: 37.5 np: 0.0 *
m04 	t: -2.69 p: 0.012 	T: 91.5 np: 0.019 *
m05 	t: 0.2 p: 0.843 	T: 187.0 np: 0.962 n.s.
m06 	t: 3.26 p: 0.003 	T: 65.0 np: 0.005 *
m07 	t: 3.54 p: 0.001 	T: 64.5 np: 0.003 *
m08 	t: 2.98 p: 0.006 	T: 66.5 np: 0.005 *
m09 	t: 3.69 p: 0.001 	T: 44.5 np: 0.002 *
m10 	t: 4.44 p: 0.0 	T: 7.5 np: 0.0 *
mouth whole
m00 	t: 9.23 p: 0.0 	T: 4.0 np: 0.0 *
m01 	t: 10.17 p: 0.0 	T: 0.0 np: 0.0 *
m02 	t: 8.76 p: 0.0 	T: 5.0 np: 0.0 *
m03 	t: 10.56 p: 0.0 	T: 0.0 np: 0.0 *
m04 	t: 7.16 p: 0.0 	T: 0.0 np: 0.0 *
m05 	t: 2.15 p: 0.04 	T: 105.0 np: 0.044 *
m06 	t: -0.86 p: 0.397 	T: 136.5 np: 0.484 n.s.
m07 	t: -4.84 p: 0.0 	T: 37.5 np: 0.0 *
m08 	t: -6.05 p: 0.0 	T: 18.0 np: 0.0 *
m09 	t: -7.79 p: 0.0 	T: 3.0 np: 0.0 *
m10 	t: -9.03 p: 0.0 	T: 0.0 np: 0.0 *
mouth eyes
m00 	t: 6.09 p: 0.0 	T: 21.5 np: 0.0 *
m01 	t: 5.27 p: 0.0 	T: 24.5 np: 0.0 *
m02 	t: 5.38 p: 0.0 	T: 30.5 np: 0.0 *
m03 	t: 3.98 p: 0.0 	T: 38.0 np: 0.001 *
m04 	t: 4.14 p: 0.0 	T: 57.5 np: 0.001 *
m05 	t: 2.06 p: 0.049 	T: 101.5 np: 0.06 n.s.
m06 	t: 1.36 p: 0.186 	T: 146.5 np: 0.198 n.s.
m07 	t: -2.24 p: 0.034 	T: 93.5 np: 0.037 *
m08 	t: -4.11 p: 0.0 	T: 48.0 np: 0.001 *
m09 	t: -6.22 p: 0.0 	T: 15.5 np: 0.0 *
m10 	t: -6.54 p: 0.0 	T: 3.0 np: 0.0 *
eyes whole
m00 	t: 2.42 p: 0.023 	T: 64.5 np: 0.042 *
m01 	t: 3.32 p: 0.003 	T: 52.0 np: 0.005 *
m02 	t: 3.84 p: 0.001 	T: 40.5 np: 0.001 *
m03 	t: 4.16 p: 0.0 	T: 37.5 np: 0.0 *
m04 	t: 2.69 p: 0.012 	T: 91.5 np: 0.019 *
m05 	t: -0.2 p: 0.843 	T: 187.0 np: 0.962 n.s.
m06 	t: -3.26 p: 0.003 	T: 65.0 np: 0.005 *
m07 	t: -3.54 p: 0.001 	T: 64.5 np: 0.003 *
m08 	t: -2.98 p: 0.006 	T: 66.5 np: 0.005 *
m09 	t: -3.69 p: 0.001 	T: 44.5 np: 0.002 *
m10 	t: -4.44 p: 0.0 	T: 7.5 np: 0.0 *
eyes mouth
m00 	t: -6.09 p: 0.0 	T: 21.5 np: 0.0 *
m01 	t: -5.27 p: 0.0 	T: 24.5 np: 0.0 *
m02 	t: -5.38 p: 0.0 	T: 30.5 np: 0.0 *
m03 	t: -3.98 p: 0.0 	T: 38.0 np: 0.001 *
m04 	t: -4.14 p: 0.0 	T: 57.5 np: 0.001 *
m05 	t: -2.06 p: 0.049 	T: 101.5 np: 0.06 n.s.
m06 	t: -1.36 p: 0.186 	T: 146.5 np: 0.198 n.s.
m07 	t: 2.24 p: 0.034 	T: 93.5 np: 0.037 *
m08 	t: 4.11 p: 0.0 	T: 48.0 np: 0.001 *
m09 	t: 6.22 p: 0.0 	T: 15.5 np: 0.0 *
m10 	t: 6.54 p: 0.0 	T: 3.0 np: 0.0 *
```

Pairwise differences of the differences 'whole-eyes' and 'whole-mouth':  
This analysis asks whether the eyes are more similar to the whole face than the mouth is.

In [13]:

```
diff_whole_eyes = exp1Table['whole']-exp1Table['eyes']
diff_whole_mouth = exp1Table['whole']-exp1Table['mouth']

for m in exp1Table.columns.levels[1]:
    t,p = stats.ttest_rel(diff_whole_eyes[m],diff_whole_mouth[m])
    W,wp = stats.wilcoxon(diff_whole_eyes[m],diff_whole_mouth[m])
    if p < 0.05 and wp < 0.05:
        sig = '*'
    else:
        sig = 'n.s.'
                    
    print m,'\tt:',round(t,2),'p:',round(p,3),'\tT:',round(W,2),'np:',round(wp,3), sig
```

```
m00 	t: 6.09 p: 0.0 	T: 20.5 np: 0.0 *
m01 	t: 5.27 p: 0.0 	T: 25.0 np: 0.0 *
m02 	t: 5.38 p: 0.0 	T: 30.5 np: 0.0 *
m03 	t: 3.98 p: 0.0 	T: 37.5 np: 0.001 *
m04 	t: 4.14 p: 0.0 	T: 57.5 np: 0.001 *
m05 	t: 2.06 p: 0.049 	T: 101.5 np: 0.06 n.s.
m06 	t: 1.36 p: 0.186 	T: 146.5 np: 0.198 n.s.
m07 	t: -2.24 p: 0.034 	T: 93.5 np: 0.037 *
m08 	t: -4.11 p: 0.0 	T: 48.0 np: 0.001 *
m09 	t: -6.22 p: 0.0 	T: 15.5 np: 0.0 *
m10 	t: -6.54 p: 0.0 	T: 3.0 np: 0.0 *
```

# Experiment 2¶

In [19]:

```
# colors for exp. 2
myPalette=['#ee4035','#4f5b66','#325da7','#da6b30']
```

In [20]:

```
exp2Table = load_this(pList[8])
```

In [21]:

```
exp2Table
```

Out[21]:

|  | fearLOW | | | | | | | | | | | angerLOW | | | | | | | | | | | fearUP | | | | | | | | | | | angerUP | | | | | | | | | | |
| --- | --- | --- | --- | --- | --- | --- | --- | --- | --- | --- | --- | --- | --- | --- | --- | --- | --- | --- | --- | --- | --- | --- | --- | --- | --- | --- | --- | --- | --- | --- | --- | --- | --- | --- | --- | --- | --- | --- | --- | --- | --- | --- | --- | --- |
| grade | m00 | m01 | m02 | m03 | m04 | m05 | m06 | m07 | m08 | m09 | m10 | m00 | m01 | m02 | m03 | m04 | m05 | m06 | m07 | m08 | m09 | m10 | m00 | m01 | m02 | m03 | m04 | m05 | m06 | m07 | m08 | m09 | m10 | m00 | m01 | m02 | m03 | m04 | m05 | m06 | m07 | m08 | m09 | m10 |
| p |  |  |  |  |  |  |  |  |  |  |  |  |  |  |  |  |  |  |  |  |  |  |  |  |  |  |  |  |  |  |  |  |  |  |  |  |  |  |  |  |  |  |  |  |
| p001 | 15.0 | 25.0 | 42.5 | 40.0 | 42.5 | 50.0 | 55.0 | 62.5 | 77.5 | 77.5 | 80.0 | 25.0 | 32.5 | 32.5 | 45.0 | 37.5 | 52.5 | 62.5 | 67.5 | 75.0 | 75.0 | 87.5 | 35.0 | 32.5 | 42.5 | 40.0 | 42.5 | 45.0 | 42.5 | 42.5 | 45.0 | 52.5 | 50.0 | 57.5 | 42.5 | 55.0 | 57.5 | 62.5 | 50.0 | 57.5 | 57.5 | 67.5 | 60.0 | 70.0 |
| p002 | 40.0 | 27.5 | 57.5 | 42.5 | 52.5 | 50.0 | 60.0 | 87.5 | 80.0 | 85.0 | 87.5 | 27.5 | 52.5 | 52.5 | 52.5 | 60.0 | 57.5 | 75.0 | 87.5 | 67.5 | 82.5 | 82.5 | 40.0 | 40.0 | 40.0 | 35.0 | 50.0 | 42.5 | 42.5 | 45.0 | 47.5 | 47.5 | 50.0 | 67.5 | 72.5 | 80.0 | 72.5 | 67.5 | 72.5 | 77.5 | 82.5 | 85.0 | 95.0 | 90.0 |
| p003 | 10.0 | 7.5 | 15.0 | 10.0 | 12.5 | 35.0 | 52.5 | 65.0 | 90.0 | 90.0 | 97.5 | 22.5 | 10.0 | 22.5 | 20.0 | 40.0 | 57.5 | 82.5 | 85.0 | 92.5 | 95.0 | 95.0 | 32.5 | 30.0 | 35.0 | 30.0 | 32.5 | 42.5 | 45.0 | 57.5 | 47.5 | 52.5 | 52.5 | 62.5 | 65.0 | 52.5 | 60.0 | 67.5 | 62.5 | 57.5 | 70.0 | 72.5 | 67.5 | 75.0 |
| p004 | 12.5 | 17.5 | 17.5 | 25.0 | 25.0 | 35.0 | 45.0 | 60.0 | 42.5 | 52.5 | 60.0 | 35.0 | 37.5 | 47.5 | 60.0 | 57.5 | 55.0 | 65.0 | 65.0 | 77.5 | 85.0 | 85.0 | 37.5 | 22.5 | 37.5 | 42.5 | 45.0 | 47.5 | 60.0 | 55.0 | 62.5 | 50.0 | 57.5 | 57.5 | 65.0 | 60.0 | 60.0 | 70.0 | 75.0 | 85.0 | 82.5 | 85.0 | 85.0 | 80.0 |
| p005 | 2.5 | 12.5 | 17.5 | 20.0 | 25.0 | 35.0 | 52.5 | 70.0 | 82.5 | 77.5 | 92.5 | 17.5 | 22.5 | 20.0 | 25.0 | 42.5 | 55.0 | 70.0 | 90.0 | 92.5 | 92.5 | 97.5 | 5.0 | 5.0 | 7.5 | 7.5 | 15.0 | 12.5 | 12.5 | 20.0 | 22.5 | 15.0 | 10.0 | 92.5 | 90.0 | 90.0 | 95.0 | 92.5 | 95.0 | 92.5 | 92.5 | 97.5 | 100.0 | 100.0 |
| p006 | 40.0 | 32.5 | 42.5 | 37.5 | 50.0 | 50.0 | 52.5 | 65.0 | 75.0 | 65.0 | 77.5 | 32.5 | 35.0 | 37.5 | 42.5 | 57.5 | 60.0 | 65.0 | 72.5 | 67.5 | 77.5 | 97.5 | 37.5 | 45.0 | 47.5 | 60.0 | 52.5 | 40.0 | 55.0 | 67.5 | 50.0 | 50.0 | 37.5 | 62.5 | 72.5 | 60.0 | 65.0 | 60.0 | 77.5 | 57.5 | 77.5 | 67.5 | 72.5 | 67.5 |
| p007 | 17.5 | 22.5 | 22.5 | 25.0 | 22.5 | 30.0 | 50.0 | 55.0 | 72.5 | 77.5 | 85.0 | 30.0 | 37.5 | 32.5 | 32.5 | 37.5 | 45.0 | 67.5 | 90.0 | 80.0 | 82.5 | 82.5 | 25.0 | 17.5 | 30.0 | 20.0 | 22.5 | 25.0 | 20.0 | 37.5 | 30.0 | 35.0 | 37.5 | 92.5 | 85.0 | 82.5 | 90.0 | 87.5 | 85.0 | 95.0 | 87.5 | 92.5 | 92.5 | 85.0 |
| p008 | 22.5 | 25.0 | 27.5 | 30.0 | 32.5 | 40.0 | 50.0 | 50.0 | 60.0 | 65.0 | 72.5 | 22.5 | 30.0 | 35.0 | 42.5 | 60.0 | 65.0 | 65.0 | 67.5 | 75.0 | 82.5 | 70.0 | 37.5 | 42.5 | 30.0 | 42.5 | 52.5 | 45.0 | 55.0 | 67.5 | 62.5 | 55.0 | 75.0 | 62.5 | 57.5 | 70.0 | 65.0 | 70.0 | 77.5 | 80.0 | 77.5 | 92.5 | 80.0 | 87.5 |
| p009 | 42.5 | 35.0 | 40.0 | 40.0 | 62.5 | 40.0 | 50.0 | 62.5 | 70.0 | 75.0 | 82.5 | 32.5 | 45.0 | 40.0 | 47.5 | 42.5 | 50.0 | 60.0 | 67.5 | 87.5 | 77.5 | 77.5 | 37.5 | 47.5 | 52.5 | 42.5 | 45.0 | 47.5 | 50.0 | 45.0 | 50.0 | 45.0 | 47.5 | 70.0 | 70.0 | 75.0 | 70.0 | 77.5 | 62.5 | 65.0 | 62.5 | 65.0 | 77.5 | 65.0 |
| p010 | 7.5 | 15.0 | 22.5 | 22.5 | 25.0 | 50.0 | 55.0 | 52.5 | 82.5 | 82.5 | 87.5 | 32.5 | 10.0 | 27.5 | 30.0 | 35.0 | 52.5 | 70.0 | 77.5 | 82.5 | 85.0 | 95.0 | 17.5 | 12.5 | 12.5 | 20.0 | 15.0 | 22.5 | 25.0 | 32.5 | 20.0 | 30.0 | 42.5 | 55.0 | 67.5 | 57.5 | 70.0 | 57.5 | 75.0 | 72.5 | 80.0 | 72.5 | 80.0 | 90.0 |
| p011 | 15.0 | 7.5 | 10.0 | 20.0 | 30.0 | 37.5 | 55.0 | 80.0 | 70.0 | 82.5 | 92.5 | 7.5 | 17.5 | 20.0 | 20.0 | 30.0 | 40.0 | 60.0 | 72.5 | 82.5 | 90.0 | 95.0 | 30.0 | 37.5 | 30.0 | 42.5 | 50.0 | 60.0 | 65.0 | 70.0 | 75.0 | 85.0 | 82.5 | 47.5 | 37.5 | 45.0 | 35.0 | 60.0 | 60.0 | 72.5 | 82.5 | 82.5 | 82.5 | 80.0 |
| p012 | 7.5 | 12.5 | 10.0 | 17.5 | 22.5 | 40.0 | 50.0 | 62.5 | 60.0 | 85.0 | 92.5 | 22.5 | 22.5 | 30.0 | 27.5 | 42.5 | 47.5 | 65.0 | 67.5 | 92.5 | 92.5 | 95.0 | 12.5 | 15.0 | 15.0 | 7.5 | 15.0 | 20.0 | 22.5 | 40.0 | 35.0 | 40.0 | 42.5 | 57.5 | 55.0 | 47.5 | 55.0 | 65.0 | 60.0 | 77.5 | 77.5 | 82.5 | 92.5 | 82.5 |
| p013 | 12.5 | 17.5 | 12.5 | 10.0 | 22.5 | 30.0 | 22.5 | 40.0 | 45.0 | 47.5 | 62.5 | 32.5 | 27.5 | 35.0 | 30.0 | 37.5 | 47.5 | 32.5 | 57.5 | 55.0 | 62.5 | 62.5 | 25.0 | 32.5 | 30.0 | 22.5 | 22.5 | 27.5 | 32.5 | 32.5 | 27.5 | 35.0 | 40.0 | 52.5 | 50.0 | 37.5 | 42.5 | 52.5 | 47.5 | 47.5 | 57.5 | 52.5 | 45.0 | 62.5 |
| p015 | 5.0 | 12.5 | 12.5 | 20.0 | 25.0 | 40.0 | 50.0 | 72.5 | 67.5 | 82.5 | 85.0 | 12.5 | 20.0 | 20.0 | 30.0 | 32.5 | 47.5 | 50.0 | 67.5 | 85.0 | 80.0 | 87.5 | 45.0 | 47.5 | 32.5 | 42.5 | 35.0 | 40.0 | 47.5 | 45.0 | 45.0 | 47.5 | 42.5 | 72.5 | 47.5 | 50.0 | 55.0 | 42.5 | 57.5 | 57.5 | 42.5 | 62.5 | 52.5 | 50.0 |
| p016 | 7.5 | 15.0 | 7.5 | 17.5 | 20.0 | 35.0 | 45.0 | 57.5 | 67.5 | 77.5 | 95.0 | 17.5 | 20.0 | 25.0 | 27.5 | 37.5 | 45.0 | 55.0 | 62.5 | 77.5 | 75.0 | 92.5 | 17.5 | 27.5 | 15.0 | 30.0 | 27.5 | 37.5 | 40.0 | 60.0 | 55.0 | 52.5 | 67.5 | 47.5 | 60.0 | 67.5 | 52.5 | 70.0 | 72.5 | 80.0 | 80.0 | 82.5 | 80.0 | 77.5 |
| p017 | 27.5 | 25.0 | 22.5 | 50.0 | 45.0 | 40.0 | 55.0 | 72.5 | 65.0 | 85.0 | 85.0 | 30.0 | 40.0 | 30.0 | 37.5 | 47.5 | 55.0 | 65.0 | 70.0 | 72.5 | 87.5 | 90.0 | 10.0 | 22.5 | 27.5 | 20.0 | 35.0 | 37.5 | 27.5 | 30.0 | 30.0 | 32.5 | 47.5 | 35.0 | 40.0 | 35.0 | 45.0 | 35.0 | 40.0 | 45.0 | 42.5 | 37.5 | 42.5 | 42.5 |
| p018 | 15.0 | 12.5 | 15.0 | 27.5 | 32.5 | 47.5 | 47.5 | 75.0 | 75.0 | 87.5 | 90.0 | 25.0 | 30.0 | 30.0 | 30.0 | 40.0 | 47.5 | 65.0 | 77.5 | 87.5 | 95.0 | 92.5 | 40.0 | 47.5 | 55.0 | 40.0 | 62.5 | 50.0 | 47.5 | 47.5 | 50.0 | 55.0 | 67.5 | 77.5 | 80.0 | 82.5 | 82.5 | 80.0 | 80.0 | 80.0 | 90.0 | 87.5 | 92.5 | 82.5 |
| p019 | 25.0 | 32.5 | 30.0 | 37.5 | 35.0 | 40.0 | 50.0 | 72.5 | 72.5 | 77.5 | 87.5 | 30.0 | 40.0 | 30.0 | 30.0 | 42.5 | 65.0 | 62.5 | 60.0 | 87.5 | 90.0 | 90.0 | 25.0 | 22.5 | 22.5 | 25.0 | 17.5 | 35.0 | 30.0 | 37.5 | 32.5 | 37.5 | 32.5 | 65.0 | 67.5 | 62.5 | 65.0 | 75.0 | 72.5 | 80.0 | 72.5 | 77.5 | 82.5 | 92.5 |
| p020 | 17.5 | 15.0 | 20.0 | 22.5 | 25.0 | 32.5 | 40.0 | 50.0 | 50.0 | 55.0 | 60.0 | 55.0 | 55.0 | 47.5 | 62.5 | 60.0 | 65.0 | 87.5 | 90.0 | 80.0 | 85.0 | 92.5 | 15.0 | 20.0 | 25.0 | 25.0 | 35.0 | 55.0 | 57.5 | 75.0 | 82.5 | 82.5 | 92.5 | 25.0 | 27.5 | 20.0 | 25.0 | 32.5 | 47.5 | 70.0 | 75.0 | 82.5 | 82.5 | 87.5 |
| p021 | 10.0 | 7.5 | 15.0 | 15.0 | 25.0 | 37.5 | 50.0 | 70.0 | 80.0 | 92.5 | 85.0 | 12.5 | 5.0 | 12.5 | 27.5 | 25.0 | 45.0 | 65.0 | 75.0 | 85.0 | 90.0 | 90.0 | 2.5 | 10.0 | 5.0 | 10.0 | 10.0 | 22.5 | 27.5 | 27.5 | 22.5 | 32.5 | 32.5 | 40.0 | 40.0 | 37.5 | 45.0 | 42.5 | 50.0 | 52.5 | 57.5 | 60.0 | 60.0 | 55.0 |
| p022 | 22.5 | 27.5 | 30.0 | 42.5 | 47.5 | 47.5 | 65.0 | 72.5 | 77.5 | 87.5 | 87.5 | 30.0 | 32.5 | 40.0 | 52.5 | 52.5 | 52.5 | 52.5 | 75.0 | 77.5 | 85.0 | 92.5 | 55.0 | 65.0 | 72.5 | 62.5 | 67.5 | 62.5 | 65.0 | 55.0 | 75.0 | 62.5 | 70.0 | 77.5 | 72.5 | 72.5 | 70.0 | 85.0 | 80.0 | 72.5 | 75.0 | 67.5 | 77.5 | 72.5 |
| p023 | 15.0 | 20.0 | 20.0 | 20.0 | 30.0 | 45.0 | 60.0 | 85.0 | 80.0 | 82.5 | 80.0 | 25.0 | 20.0 | 22.5 | 35.0 | 42.5 | 40.0 | 72.5 | 82.5 | 87.5 | 87.5 | 92.5 | 32.5 | 27.5 | 42.5 | 27.5 | 42.5 | 57.5 | 42.5 | 60.0 | 62.5 | 80.0 | 75.0 | 50.0 | 47.5 | 47.5 | 42.5 | 50.0 | 70.0 | 75.0 | 87.5 | 90.0 | 75.0 | 85.0 |
| p024 | 2.5 | 10.0 | 20.0 | 20.0 | 27.5 | 45.0 | 50.0 | 72.5 | 82.5 | 90.0 | 92.5 | 15.0 | 25.0 | 25.0 | 30.0 | 40.0 | 55.0 | 62.5 | 77.5 | 80.0 | 95.0 | 90.0 | 5.0 | 20.0 | 15.0 | 10.0 | 25.0 | 27.5 | 25.0 | 30.0 | 30.0 | 32.5 | 30.0 | 22.5 | 37.5 | 40.0 | 35.0 | 47.5 | 55.0 | 50.0 | 57.5 | 67.5 | 57.5 | 75.0 |
| p025 | 50.0 | 35.0 | 40.0 | 40.0 | 50.0 | 55.0 | 55.0 | 65.0 | 75.0 | 75.0 | 87.5 | 60.0 | 60.0 | 62.5 | 60.0 | 75.0 | 67.5 | 67.5 | 82.5 | 87.5 | 90.0 | 97.5 | 50.0 | 50.0 | 45.0 | 60.0 | 55.0 | 65.0 | 60.0 | 67.5 | 77.5 | 75.0 | 67.5 | 67.5 | 67.5 | 62.5 | 70.0 | 72.5 | 82.5 | 90.0 | 85.0 | 82.5 | 82.5 | 90.0 |
| p026 | 10.0 | 12.5 | 15.0 | 12.5 | 22.5 | 40.0 | 45.0 | 70.0 | 72.5 | 90.0 | 92.5 | 32.5 | 32.5 | 32.5 | 35.0 | 45.0 | 60.0 | 65.0 | 90.0 | 92.5 | 100.0 | 100.0 | 27.5 | 32.5 | 22.5 | 25.0 | 45.0 | 35.0 | 45.0 | 45.0 | 52.5 | 55.0 | 67.5 | 57.5 | 62.5 | 70.0 | 80.0 | 95.0 | 82.5 | 95.0 | 90.0 | 92.5 | 90.0 | 100.0 |
| p027 | 30.0 | 27.5 | 12.5 | 25.0 | 37.5 | 40.0 | 52.5 | 37.5 | 55.0 | 67.5 | 67.5 | 30.0 | 40.0 | 30.0 | 32.5 | 47.5 | 30.0 | 55.0 | 62.5 | 62.5 | 65.0 | 65.0 | 40.0 | 42.5 | 47.5 | 37.5 | 45.0 | 60.0 | 47.5 | 62.5 | 55.0 | 55.0 | 60.0 | 65.0 | 60.0 | 52.5 | 57.5 | 75.0 | 62.5 | 62.5 | 62.5 | 52.5 | 65.0 | 60.0 |
| p028 | 10.0 | 10.0 | 17.5 | 27.5 | 40.0 | 57.5 | 77.5 | 97.5 | 97.5 | 100.0 | 100.0 | 20.0 | 20.0 | 20.0 | 27.5 | 42.5 | 75.0 | 87.5 | 100.0 | 100.0 | 100.0 | 100.0 | 27.5 | 35.0 | 32.5 | 30.0 | 45.0 | 50.0 | 60.0 | 70.0 | 75.0 | 75.0 | 80.0 | 47.5 | 52.5 | 70.0 | 75.0 | 82.5 | 80.0 | 95.0 | 92.5 | 95.0 | 95.0 | 97.5 |
| p029 | 10.0 | 12.5 | 17.5 | 32.5 | 20.0 | 50.0 | 55.0 | 65.0 | 90.0 | 92.5 | 95.0 | 22.5 | 17.5 | 20.0 | 20.0 | 27.5 | 52.5 | 62.5 | 90.0 | 90.0 | 92.5 | 92.5 | 32.5 | 37.5 | 40.0 | 50.0 | 40.0 | 42.5 | 52.5 | 45.0 | 55.0 | 55.0 | 50.0 | 60.0 | 85.0 | 75.0 | 70.0 | 75.0 | 82.5 | 90.0 | 82.5 | 77.5 | 82.5 | 87.5 |
| p030 | 10.0 | 2.5 | 12.5 | 7.5 | 17.5 | 35.0 | 47.5 | 62.5 | 77.5 | 85.0 | 92.5 | 15.0 | 15.0 | 15.0 | 25.0 | 30.0 | 55.0 | 60.0 | 72.5 | 90.0 | 90.0 | 97.5 | 20.0 | 25.0 | 30.0 | 27.5 | 20.0 | 40.0 | 42.5 | 42.5 | 55.0 | 52.5 | 60.0 | 50.0 | 47.5 | 60.0 | 52.5 | 65.0 | 57.5 | 65.0 | 65.0 | 77.5 | 75.0 | 77.5 |

In [22]:

```
exp2Plt = oo_plt.GroupErrBar(exp2Table,"Experiment 2")

oo_plt.GroupErrBar.plot_setup(exp2Plt,
                              select=[0,1,2,3],
                              mycolors=myPalette,
                              xlab='Morphing Grade (% Angry)\n')

oo_plt.GroupErrBar.plot_sum(exp2Plt,mode='mean')

oo_plt.GroupErrBar.plot_setup(exp2Plt,select=[0,2],mycolors=[myPalette[0],myPalette[2]],xlab='Morphing Grade (% Angry)\n')
oo_plt.GroupErrBar.plot_sum(exp2Plt,mode='mean')

oo_plt.GroupErrBar.plot_setup(exp2Plt,select=[1,3],mycolors=['c',myPalette[1],myPalette[3]],xlab='Morphing Grade (% Angry)\n')
oo_plt.GroupErrBar.plot_sum(exp2Plt,mode='mean')
```

```
The Created Object Has The Following Properties:
conditions: ['angerLOW', 'angerUP', 'fearLOW', 'fearUP']
cases: ['p001', 'p002', 'p003', 'p004', 'p005', 'p006', 'p007', 'p008', 'p009', 'p010', 'p011', 'p012', 'p013', 'p015', 'p016', 'p017', 'p018', 'p019', 'p020', 'p021', 'p022', 'p023', 'p024', 'p025', 'p026', 'p027', 'p028', 'p029', 'p030']
grades: ['m00', 'm01', 'm02', 'm03', 'm04', 'm05', 'm06', 'm07', 'm08', 'm09', 'm10']

Currently Selected Conditions: ['angerLOW', 'angerUP', 'fearLOW', 'fearUP']
```

```
Currently Selected Conditions: ['angerLOW', 'fearLOW']
```

```
Currently Selected Conditions: ['angerUP', 'fearUP']
```

## Descriptives¶

In [23]:

```
exp2Table.describe()
```

Out[23]:

|  | fearLOW | | | | | | | | | | | angerLOW | | | | | | | | | | | fearUP | | | | | | | | | | | angerUP | | | | | | | | | | |
| --- | --- | --- | --- | --- | --- | --- | --- | --- | --- | --- | --- | --- | --- | --- | --- | --- | --- | --- | --- | --- | --- | --- | --- | --- | --- | --- | --- | --- | --- | --- | --- | --- | --- | --- | --- | --- | --- | --- | --- | --- | --- | --- | --- | --- |
|  | m00 | m01 | m02 | m03 | m04 | m05 | m06 | m07 | m08 | m09 | m10 | m00 | m01 | m02 | m03 | m04 | m05 | m06 | m07 | m08 | m09 | m10 | m00 | m01 | m02 | m03 | m04 | m05 | m06 | m07 | m08 | m09 | m10 | m00 | m01 | m02 | m03 | m04 | m05 | m06 | m07 | m08 | m09 | m10 |
| count | 29.000000 | 29.000000 | 29.000000 | 29.00000 | 29.000000 | 29.000000 | 29.000000 | 29.000000 | 29.000000 | 29.000000 | 29.000000 | 29.000000 | 29.000000 | 29.000000 | 29.000000 | 29.000000 | 29.000000 | 29.000000 | 29.000000 | 29.000000 | 29.000000 | 29.000000 | 29.000000 | 29.000000 | 29.000000 | 29.000000 | 29.000000 | 29.000000 | 29.000000 | 29.000000 | 29.000000 | 29.000000 | 29.000000 | 29.000000 | 29.000000 | 29.000000 | 29.000000 | 29.000000 | 29.000000 | 29.000000 | 29.000000 | 29.000000 | 29.000000 | 29.000000 |
| mean | 17.672414 | 18.448276 | 22.241379 | 26.12069 | 31.896552 | 41.724138 | 51.551724 | 65.862069 | 72.155172 | 78.965517 | 84.568966 | 26.637931 | 29.396552 | 30.862069 | 35.775862 | 43.706897 | 53.189655 | 64.655172 | 75.948276 | 81.724138 | 85.775862 | 89.137931 | 28.189655 | 31.465517 | 32.413793 | 32.241379 | 36.810345 | 41.206897 | 42.931034 | 48.706897 | 49.310345 | 50.862069 | 54.051724 | 58.534483 | 59.482759 | 59.224138 | 60.689655 | 66.034483 | 68.017241 | 72.327586 | 73.965517 | 76.120690 | 76.637931 | 78.189655 |
| std | 12.498768 | 9.146960 | 12.033154 | 11.25171 | 12.222914 | 7.322405 | 8.924630 | 13.114807 | 12.951216 | 12.455586 | 10.857621 | 11.245552 | 13.638430 | 11.461813 | 12.303880 | 11.429534 | 9.399291 | 10.809308 | 10.987286 | 10.091179 | 9.065817 | 9.688816 | 13.576775 | 13.879540 | 15.199312 | 15.153159 | 15.409537 | 13.588675 | 14.439977 | 14.919736 | 17.889749 | 16.906728 | 18.545477 | 16.400638 | 15.999153 | 16.571052 | 16.568265 | 16.291393 | 13.764267 | 15.088001 | 14.039441 | 14.401547 | 15.048669 | 14.560474 |
| min | 2.500000 | 2.500000 | 7.500000 | 7.50000 | 12.500000 | 30.000000 | 22.500000 | 37.500000 | 42.500000 | 47.500000 | 60.000000 | 7.500000 | 5.000000 | 12.500000 | 20.000000 | 25.000000 | 30.000000 | 32.500000 | 57.500000 | 55.000000 | 62.500000 | 62.500000 | 2.500000 | 5.000000 | 5.000000 | 7.500000 | 10.000000 | 12.500000 | 12.500000 | 20.000000 | 20.000000 | 15.000000 | 10.000000 | 22.500000 | 27.500000 | 20.000000 | 25.000000 | 32.500000 | 40.000000 | 45.000000 | 42.500000 | 37.500000 | 42.500000 | 42.500000 |
| 25% | 10.000000 | 12.500000 | 15.000000 | 20.00000 | 22.500000 | 35.000000 | 50.000000 | 60.000000 | 67.500000 | 75.000000 | 80.000000 | 20.000000 | 20.000000 | 22.500000 | 27.500000 | 37.500000 | 47.500000 | 60.000000 | 67.500000 | 77.500000 | 82.500000 | 87.500000 | 17.500000 | 22.500000 | 22.500000 | 22.500000 | 22.500000 | 35.000000 | 30.000000 | 37.500000 | 32.500000 | 37.500000 | 42.500000 | 50.000000 | 47.500000 | 47.500000 | 52.500000 | 57.500000 | 57.500000 | 57.500000 | 62.500000 | 67.500000 | 67.500000 | 70.000000 |
| 50% | 15.000000 | 15.000000 | 17.500000 | 25.00000 | 27.500000 | 40.000000 | 50.000000 | 65.000000 | 75.000000 | 82.500000 | 87.500000 | 25.000000 | 30.000000 | 30.000000 | 30.000000 | 42.500000 | 52.500000 | 65.000000 | 75.000000 | 82.500000 | 87.500000 | 92.500000 | 30.000000 | 32.500000 | 30.000000 | 30.000000 | 40.000000 | 42.500000 | 45.000000 | 45.000000 | 50.000000 | 52.500000 | 50.000000 | 57.500000 | 60.000000 | 60.000000 | 60.000000 | 67.500000 | 72.500000 | 72.500000 | 77.500000 | 77.500000 | 80.000000 | 80.000000 |
| 75% | 22.500000 | 25.000000 | 27.500000 | 37.50000 | 40.000000 | 47.500000 | 55.000000 | 72.500000 | 80.000000 | 87.500000 | 92.500000 | 32.500000 | 37.500000 | 35.000000 | 42.500000 | 47.500000 | 57.500000 | 67.500000 | 85.000000 | 87.500000 | 92.500000 | 95.000000 | 37.500000 | 42.500000 | 42.500000 | 42.500000 | 45.000000 | 50.000000 | 55.000000 | 60.000000 | 62.500000 | 55.000000 | 67.500000 | 67.500000 | 70.000000 | 70.000000 | 70.000000 | 75.000000 | 80.000000 | 80.000000 | 82.500000 | 85.000000 | 85.000000 | 87.500000 |
| max | 50.000000 | 35.000000 | 57.500000 | 50.00000 | 62.500000 | 57.500000 | 77.500000 | 97.500000 | 97.500000 | 100.000000 | 100.000000 | 60.000000 | 60.000000 | 62.500000 | 62.500000 | 75.000000 | 75.000000 | 87.500000 | 100.000000 | 100.000000 | 100.000000 | 100.000000 | 55.000000 | 65.000000 | 72.500000 | 62.500000 | 67.500000 | 65.000000 | 65.000000 | 75.000000 | 82.500000 | 85.000000 | 92.500000 | 92.500000 | 90.000000 | 90.000000 | 95.000000 | 95.000000 | 95.000000 | 95.000000 | 92.500000 | 97.500000 | 100.000000 | 100.000000 |

## Inferential Statistics¶

Pairwise Comparisons of every condition with every other one, for each of the 11 morphing grades

In [24]:

```
inf_stats(exp2Table,['fearLOW', 'angerLOW', 'fearUP', 'angerUP'],0.01)
```

```
fearLOW angerLOW
m00 	t: -4.43 p: 0.0 	T: 43.0 np: 0.0 *
m01 	t: -6.39 p: 0.0 	T: 8.0 np: 0.0 *
m02 	t: -4.63 p: 0.0 	T: 37.0 np: 0.0 *
m03 	t: -4.56 p: 0.0 	T: 44.0 np: 0.0 *
m04 	t: -5.45 p: 0.0 	T: 24.0 np: 0.0 *
m05 	t: -6.38 p: 0.0 	T: 18.5 np: 0.0 *
m06 	t: -7.11 p: 0.0 	T: 13.5 np: 0.0 *
m07 	t: -4.3 p: 0.0 	T: 47.0 np: 0.0 *
m08 	t: -4.68 p: 0.0 	T: 29.0 np: 0.0 *
m09 	t: -3.99 p: 0.0 	T: 38.5 np: 0.0 *
m10 	t: -2.81 p: 0.009 	T: 77.5 np: 0.007 *
fearLOW fearUP
m00 	t: -4.45 p: 0.0 	T: 35.5 np: 0.0 *
m01 	t: -5.48 p: 0.0 	T: 31.5 np: 0.0 *
m02 	t: -3.67 p: 0.001 	T: 71.0 np: 0.003 *
m03 	t: -2.42 p: 0.022 	T: 97.0 np: 0.015 n.s.
m04 	t: -2.01 p: 0.054 	T: 100.0 np: 0.054 n.s.
m05 	t: 0.21 p: 0.833 	T: 195.5 np: 0.864 n.s.
m06 	t: 3.1 p: 0.004 	T: 62.0 np: 0.007 *
m07 	t: 4.71 p: 0.0 	T: 52.5 np: 0.0 *
m08 	t: 5.37 p: 0.0 	T: 35.0 np: 0.0 *
m09 	t: 7.18 p: 0.0 	T: 14.0 np: 0.0 *
m10 	t: 7.23 p: 0.0 	T: 16.0 np: 0.0 *
fearLOW angerUP
m00 	t: -11.92 p: 0.0 	T: 0.0 np: 0.0 *
m01 	t: -13.9 p: 0.0 	T: 0.0 np: 0.0 *
m02 	t: -11.38 p: 0.0 	T: 0.0 np: 0.0 *
m03 	t: -10.5 p: 0.0 	T: 2.0 np: 0.0 *
m04 	t: -9.43 p: 0.0 	T: 2.5 np: 0.0 *
m05 	t: -10.52 p: 0.0 	T: 0.0 np: 0.0 *
m06 	t: -7.41 p: 0.0 	T: 7.5 np: 0.0 *
m07 	t: -2.62 p: 0.014 	T: 92.0 np: 0.02 n.s.
m08 	t: -1.26 p: 0.218 	T: 154.5 np: 0.269 n.s.
m09 	t: 0.7 p: 0.49 	T: 168.5 np: 0.622 n.s.
m10 	t: 2.2 p: 0.036 	T: 99.5 np: 0.031 n.s.
angerLOW fearLOW
m00 	t: 4.43 p: 0.0 	T: 43.0 np: 0.0 *
m01 	t: 6.39 p: 0.0 	T: 8.0 np: 0.0 *
m02 	t: 4.63 p: 0.0 	T: 37.0 np: 0.0 *
m03 	t: 4.56 p: 0.0 	T: 44.0 np: 0.0 *
m04 	t: 5.45 p: 0.0 	T: 24.0 np: 0.0 *
m05 	t: 6.38 p: 0.0 	T: 18.5 np: 0.0 *
m06 	t: 7.11 p: 0.0 	T: 13.5 np: 0.0 *
m07 	t: 4.3 p: 0.0 	T: 47.0 np: 0.0 *
m08 	t: 4.68 p: 0.0 	T: 29.0 np: 0.0 *
m09 	t: 3.99 p: 0.0 	T: 38.5 np: 0.0 *
m10 	t: 2.81 p: 0.009 	T: 77.5 np: 0.007 *
angerLOW fearUP
m00 	t: -0.56 p: 0.58 	T: 174.0 np: 0.507 n.s.
m01 	t: -0.7 p: 0.489 	T: 158.0 np: 0.456 n.s.
m02 	t: -0.57 p: 0.573 	T: 186.5 np: 0.501 n.s.
m03 	t: 1.29 p: 0.209 	T: 133.5 np: 0.181 n.s.
m04 	t: 2.73 p: 0.011 	T: 87.0 np: 0.014 n.s.
m05 	t: 3.96 p: 0.0 	T: 70.5 np: 0.001 *
m06 	t: 6.77 p: 0.0 	T: 12.5 np: 0.0 *
m07 	t: 8.37 p: 0.0 	T: 0.0 np: 0.0 *
m08 	t: 8.97 p: 0.0 	T: 1.5 np: 0.0 *
m09 	t: 10.31 p: 0.0 	T: 0.0 np: 0.0 *
m10 	t: 9.36 p: 0.0 	T: 1.5 np: 0.0 *
angerLOW angerUP
m00 	t: -8.74 p: 0.0 	T: 12.0 np: 0.0 *
m01 	t: -8.16 p: 0.0 	T: 11.5 np: 0.0 *
m02 	t: -7.9 p: 0.0 	T: 12.5 np: 0.0 *
m03 	t: -6.41 p: 0.0 	T: 20.0 np: 0.0 *
m04 	t: -6.32 p: 0.0 	T: 26.0 np: 0.0 *
m05 	t: -5.43 p: 0.0 	T: 32.0 np: 0.0 *
m06 	t: -2.73 p: 0.011 	T: 103.5 np: 0.014 n.s.
m07 	t: 0.89 p: 0.382 	T: 136.0 np: 0.475 n.s.
m08 	t: 2.43 p: 0.022 	T: 79.0 np: 0.024 n.s.
m09 	t: 3.61 p: 0.001 	T: 46.0 np: 0.002 *
m10 	t: 4.2 p: 0.0 	T: 38.0 np: 0.0 *
fearUP fearLOW
m00 	t: 4.45 p: 0.0 	T: 35.5 np: 0.0 *
m01 	t: 5.48 p: 0.0 	T: 31.5 np: 0.0 *
m02 	t: 3.67 p: 0.001 	T: 71.0 np: 0.003 *
m03 	t: 2.42 p: 0.022 	T: 97.0 np: 0.015 n.s.
m04 	t: 2.01 p: 0.054 	T: 100.0 np: 0.054 n.s.
m05 	t: -0.21 p: 0.833 	T: 195.5 np: 0.864 n.s.
m06 	t: -3.1 p: 0.004 	T: 62.0 np: 0.007 *
m07 	t: -4.71 p: 0.0 	T: 52.5 np: 0.0 *
m08 	t: -5.37 p: 0.0 	T: 35.0 np: 0.0 *
m09 	t: -7.18 p: 0.0 	T: 14.0 np: 0.0 *
m10 	t: -7.23 p: 0.0 	T: 16.0 np: 0.0 *
fearUP angerLOW
m00 	t: 0.56 p: 0.58 	T: 174.0 np: 0.507 n.s.
m01 	t: 0.7 p: 0.489 	T: 158.0 np: 0.456 n.s.
m02 	t: 0.57 p: 0.573 	T: 186.5 np: 0.501 n.s.
m03 	t: -1.29 p: 0.209 	T: 133.5 np: 0.181 n.s.
m04 	t: -2.73 p: 0.011 	T: 87.0 np: 0.014 n.s.
m05 	t: -3.96 p: 0.0 	T: 70.5 np: 0.001 *
m06 	t: -6.77 p: 0.0 	T: 12.5 np: 0.0 *
m07 	t: -8.37 p: 0.0 	T: 0.0 np: 0.0 *
m08 	t: -8.97 p: 0.0 	T: 1.5 np: 0.0 *
m09 	t: -10.31 p: 0.0 	T: 0.0 np: 0.0 *
m10 	t: -9.36 p: 0.0 	T: 1.5 np: 0.0 *
fearUP angerUP
m00 	t: -10.59 p: 0.0 	T: 0.0 np: 0.0 *
m01 	t: -7.84 p: 0.0 	T: 0.0 np: 0.0 *
m02 	t: -7.55 p: 0.0 	T: 2.0 np: 0.0 *
m03 	t: -7.43 p: 0.0 	T: 2.5 np: 0.0 *
m04 	t: -8.12 p: 0.0 	T: 1.0 np: 0.0 *
m05 	t: -7.51 p: 0.0 	T: 4.0 np: 0.0 *
m06 	t: -8.19 p: 0.0 	T: 0.0 np: 0.0 *
m07 	t: -8.1 p: 0.0 	T: 1.0 np: 0.0 *
m08 	t: -7.61 p: 0.0 	T: 5.0 np: 0.0 *
m09 	t: -6.83 p: 0.0 	T: 3.5 np: 0.0 *
m10 	t: -6.16 p: 0.0 	T: 8.5 np: 0.0 *
angerUP fearLOW
m00 	t: 11.92 p: 0.0 	T: 0.0 np: 0.0 *
m01 	t: 13.9 p: 0.0 	T: 0.0 np: 0.0 *
m02 	t: 11.38 p: 0.0 	T: 0.0 np: 0.0 *
m03 	t: 10.5 p: 0.0 	T: 2.0 np: 0.0 *
m04 	t: 9.43 p: 0.0 	T: 2.5 np: 0.0 *
m05 	t: 10.52 p: 0.0 	T: 0.0 np: 0.0 *
m06 	t: 7.41 p: 0.0 	T: 7.5 np: 0.0 *
m07 	t: 2.62 p: 0.014 	T: 92.0 np: 0.02 n.s.
m08 	t: 1.26 p: 0.218 	T: 154.5 np: 0.269 n.s.
m09 	t: -0.7 p: 0.49 	T: 168.5 np: 0.622 n.s.
m10 	t: -2.2 p: 0.036 	T: 99.5 np: 0.031 n.s.
angerUP angerLOW
m00 	t: 8.74 p: 0.0 	T: 12.0 np: 0.0 *
m01 	t: 8.16 p: 0.0 	T: 11.5 np: 0.0 *
m02 	t: 7.9 p: 0.0 	T: 12.5 np: 0.0 *
m03 	t: 6.41 p: 0.0 	T: 20.0 np: 0.0 *
m04 	t: 6.32 p: 0.0 	T: 26.0 np: 0.0 *
m05 	t: 5.43 p: 0.0 	T: 32.0 np: 0.0 *
m06 	t: 2.73 p: 0.011 	T: 103.5 np: 0.014 n.s.
m07 	t: -0.89 p: 0.382 	T: 136.0 np: 0.475 n.s.
m08 	t: -2.43 p: 0.022 	T: 79.0 np: 0.024 n.s.
m09 	t: -3.61 p: 0.001 	T: 46.0 np: 0.002 *
m10 	t: -4.2 p: 0.0 	T: 38.0 np: 0.0 *
angerUP fearUP
m00 	t: 10.59 p: 0.0 	T: 0.0 np: 0.0 *
m01 	t: 7.84 p: 0.0 	T: 0.0 np: 0.0 *
m02 	t: 7.55 p: 0.0 	T: 2.0 np: 0.0 *
m03 	t: 7.43 p: 0.0 	T: 2.5 np: 0.0 *
m04 	t: 8.12 p: 0.0 	T: 1.0 np: 0.0 *
m05 	t: 7.51 p: 0.0 	T: 4.0 np: 0.0 *
m06 	t: 8.19 p: 0.0 	T: 0.0 np: 0.0 *
m07 	t: 8.1 p: 0.0 	T: 1.0 np: 0.0 *
m08 	t: 7.61 p: 0.0 	T: 5.0 np: 0.0 *
m09 	t: 6.83 p: 0.0 	T: 3.5 np: 0.0 *
m10 	t: 6.16 p: 0.0 	T: 8.5 np: 0.0 *
```

### Comparison of Bias between Composite Face Conditions¶

The difference of an angry or fearful to-be-ignored half is compared between upper and lower halves.  
This analysis asks whether the biasing influence of the to-be-ignored half is stronger for the eyes over the mouth or for the mouth over the eyes.

In [25]:

```
low_diff = exp2Table['angerLOW']-exp2Table['fearLOW']
up_diff = exp2Table['angerUP']-exp2Table['fearUP']
for m in ['m00','m01','m02','m03','m04','m05','m06','m07','m08','m09','m10']:
    t,p = stats.ttest_rel(up_diff[m],low_diff[m])
    W,wp = stats.wilcoxon(up_diff[m],low_diff[m])
    if p < 0.001 and wp < 0.001:
        sig = '*'
    else:
        sig = ''
    print m,'\tt:',round(t,2),'p:',round(p,3),'\tW:',round(W,2),'wp:',round(wp,3), sig
```

```
m00 	t: 6.22 p: 0.0 	W: 21.5 wp: 0.0 *
m01 	t: 4.08 p: 0.0 	W: 51.5 wp: 0.0 *
m02 	t: 4.21 p: 0.0 	W: 51.5 wp: 0.001 *
m03 	t: 3.98 p: 0.0 	W: 42.0 wp: 0.001 *
m04 	t: 4.27 p: 0.0 	W: 42.0 wp: 0.0 *
m05 	t: 4.11 p: 0.0 	W: 50.0 wp: 0.0 *
m06 	t: 4.39 p: 0.0 	W: 49.0 wp: 0.0 *
m07 	t: 4.16 p: 0.0 	W: 53.5 wp: 0.001 *
m08 	t: 4.18 p: 0.0 	W: 43.0 wp: 0.0 *
m09 	t: 4.69 p: 0.0 	W: 46.0 wp: 0.0 *
m10 	t: 4.36 p: 0.0 	W: 42.0 wp: 0.0 *
```
