# Supplementary material for: Categorical Perception of Fear and Anger Expressions in Whole, Masked and Composite Faces
Source: PLoS One. 2015 Aug 11;10(8):e0134790. doi: 10.1371/journal.pone.0134790 (PMC4532458; doi:10.1371/journal.pone.0134790)
Supplement: S4 Code — (HTML) [file pone.0134790.s005.html]

SupplementS4\_CurveFitting-FOR\_REAL!


# Supplement S4 of

## Categorical Perception of Fear and Anger Expressions in Whole, Masked and Composite Faces.

# Fitting Functions and Getting Derivatives¶

This script fits a logistic function to the raw data of each participant.

## Import Modules¶

In [77]:

```
import numpy as np
from scipy import stats
from scipy.optimize import curve_fit

import pandas as pd
pd.set_option('max_columns', 100)
pd.set_option('max_rows', 1000)

import os
import fnmatch

import seaborn as sns
sns.set_style("ticks")
%pylab inline
```

```
Populating the interactive namespace from numpy and matplotlib
```

## Load CSV file and tranform back to DataFrame¶

In [78]:

```
def get_table(whichfolder, whichfile):
    os.chdir(whichfolder)
    loglist = []
    for fileName in os.listdir(whichfolder):
        if fnmatch.fnmatch(fileName, whichfile):
            loglist.append(fileName)
    return loglist
```

In [79]:

```
my_folder = './data/'
my_df = '*.txt'
dflist = get_table(my_folder,my_df)
dflist.sort()
```

In [80]:

```
i = 0
for df in dflist:
    print i,':',df
    i+=1
```

```
0 : Exp1AvgResults.txt
1 : Exp1AvgResultsAll.txt
2 : Exp1Pt1MainResults.txt
3 : Exp1Pt1ResultsAll.txt
4 : Exp1Pt2MainResults.txt
5 : Exp1Pt2ResultsAll.txt
6 : Exp1RtMedian.txt
7 : Exp1RtMedianAll.txt
8 : Exp2AvgResults.txt
9 : Exp2AvgResultsAll.txt
10 : Exp2Pt1Results.txt
11 : Exp2Pt1ResultsAll.txt
12 : Exp2Pt2Results.txt
13 : Exp2Pt2ResultsAll.txt
14 : Exp2RtMedian.txt
15 : Exp2RtMedianAll.txt
16 : exp1Pt1Logistic.txt
17 : exp1Pt2Logistic.txt
18 : exp1fittable.txt
19 : exp1pt1Logistic.txt
20 : exp1pt2Logistic.txt
21 : exp2Pt1Logistic.txt
22 : exp2Pt2Logistic.txt
23 : exp2fittable.txt
24 : exp2pt1Logistic.txt
25 : exp2pt2Logistic.txt
```

In [81]:

```
exp1Results = pd.read_csv(dflist[0],
                 header=[0,1],
                 index_col=[0])/100
```

In [82]:

```
cols = exp1Results.columns.tolist()
cols = [ cols[0:11]+cols[22:33]+cols[11:22] ]
exp1Results = exp1Results[cols[0]]
```

In [83]:

```
exp1Results
```

Out[83]:

|  | whole | | | | | | | | | | | eyes | | | | | | | | | | | mouth | | | | | | | | | | |
| --- | --- | --- | --- | --- | --- | --- | --- | --- | --- | --- | --- | --- | --- | --- | --- | --- | --- | --- | --- | --- | --- | --- | --- | --- | --- | --- | --- | --- | --- | --- | --- | --- | --- |
| grade | m00 | m01 | m02 | m03 | m04 | m05 | m06 | m07 | m08 | m09 | m10 | m00 | m01 | m02 | m03 | m04 | m05 | m06 | m07 | m08 | m09 | m10 | m00 | m01 | m02 | m03 | m04 | m05 | m06 | m07 | m08 | m09 | m10 |
| p |  |  |  |  |  |  |  |  |  |  |  |  |  |  |  |  |  |  |  |  |  |  |  |  |  |  |  |  |  |  |  |  |  |
| p001 | 0.125 | 0.175 | 0.250 | 0.200 | 0.275 | 0.600 | 0.675 | 0.950 | 0.975 | 1.000 | 1.000 | 0.125 | 0.050 | 0.175 | 0.225 | 0.425 | 0.425 | 0.650 | 0.850 | 0.900 | 0.975 | 0.975 | 0.325 | 0.425 | 0.400 | 0.525 | 0.575 | 0.600 | 0.750 | 0.800 | 0.850 | 0.825 | 0.825 |
| p002 | 0.150 | 0.000 | 0.050 | 0.150 | 0.225 | 0.375 | 0.575 | 0.700 | 0.875 | 0.900 | 0.975 | 0.225 | 0.250 | 0.275 | 0.350 | 0.450 | 0.600 | 0.725 | 0.825 | 0.900 | 0.925 | 0.900 | 0.175 | 0.200 | 0.300 | 0.325 | 0.400 | 0.550 | 0.750 | 0.750 | 0.850 | 0.825 | 0.900 |
| p003 | 0.125 | 0.150 | 0.150 | 0.200 | 0.475 | 0.675 | 0.775 | 0.975 | 1.000 | 1.000 | 1.000 | 0.300 | 0.375 | 0.450 | 0.625 | 0.700 | 0.750 | 0.875 | 0.850 | 0.850 | 0.975 | 0.975 | 0.250 | 0.250 | 0.375 | 0.425 | 0.600 | 0.525 | 0.725 | 0.650 | 0.650 | 0.775 | 0.850 |
| p004 | 0.050 | 0.075 | 0.200 | 0.125 | 0.325 | 0.325 | 0.600 | 0.800 | 0.875 | 0.850 | 0.875 | 0.225 | 0.275 | 0.375 | 0.275 | 0.375 | 0.575 | 0.675 | 0.575 | 0.775 | 0.800 | 0.875 | 0.200 | 0.300 | 0.350 | 0.225 | 0.400 | 0.450 | 0.450 | 0.525 | 0.650 | 0.575 | 0.575 |
| p005 | 0.000 | 0.000 | 0.100 | 0.150 | 0.200 | 0.225 | 0.450 | 0.600 | 0.825 | 0.775 | 0.850 | 0.050 | 0.025 | 0.150 | 0.275 | 0.250 | 0.350 | 0.525 | 0.600 | 0.825 | 0.875 | 0.875 | 0.200 | 0.200 | 0.300 | 0.275 | 0.350 | 0.275 | 0.450 | 0.400 | 0.575 | 0.650 | 0.550 |
| p006 | 0.050 | 0.075 | 0.025 | 0.075 | 0.175 | 0.450 | 0.775 | 0.950 | 0.950 | 1.000 | 1.000 | 0.050 | 0.075 | 0.075 | 0.050 | 0.350 | 0.400 | 0.700 | 0.825 | 0.975 | 0.975 | 1.000 | 0.175 | 0.175 | 0.225 | 0.350 | 0.375 | 0.475 | 0.625 | 0.675 | 0.750 | 0.850 | 0.875 |
| p007 | 0.125 | 0.100 | 0.150 | 0.225 | 0.150 | 0.325 | 0.600 | 0.800 | 0.900 | 0.950 | 0.975 | 0.100 | 0.100 | 0.200 | 0.225 | 0.250 | 0.450 | 0.600 | 0.700 | 0.850 | 0.900 | 0.950 | 0.375 | 0.550 | 0.575 | 0.450 | 0.550 | 0.650 | 0.725 | 0.925 | 0.950 | 0.875 | 0.875 |
| p008 | 0.175 | 0.275 | 0.250 | 0.300 | 0.425 | 0.600 | 0.625 | 0.900 | 0.950 | 0.950 | 1.000 | 0.150 | 0.150 | 0.300 | 0.325 | 0.300 | 0.425 | 0.650 | 0.800 | 0.975 | 0.925 | 0.975 | 0.600 | 0.675 | 0.650 | 0.625 | 0.675 | 0.725 | 0.800 | 0.900 | 0.950 | 0.925 | 0.925 |
| p009 | 0.050 | 0.025 | 0.000 | 0.050 | 0.250 | 0.350 | 0.625 | 0.800 | 0.950 | 0.975 | 1.000 | 0.025 | 0.025 | 0.050 | 0.150 | 0.225 | 0.450 | 0.550 | 0.875 | 0.850 | 0.950 | 0.975 | 0.100 | 0.075 | 0.200 | 0.200 | 0.275 | 0.625 | 0.650 | 0.800 | 0.850 | 0.850 | 0.875 |
| p010 | 0.150 | 0.225 | 0.300 | 0.275 | 0.250 | 0.600 | 0.700 | 0.850 | 0.975 | 0.950 | 1.000 | 0.175 | 0.350 | 0.375 | 0.375 | 0.400 | 0.400 | 0.525 | 0.700 | 0.875 | 0.825 | 0.850 | 0.350 | 0.375 | 0.375 | 0.500 | 0.550 | 0.600 | 0.700 | 0.700 | 0.775 | 0.700 | 0.850 |
| p011 | 0.025 | 0.100 | 0.100 | 0.075 | 0.200 | 0.400 | 0.775 | 0.900 | 0.925 | 0.950 | 1.000 | 0.050 | 0.050 | 0.075 | 0.100 | 0.275 | 0.250 | 0.450 | 0.800 | 0.900 | 1.000 | 0.975 | 0.325 | 0.250 | 0.425 | 0.450 | 0.575 | 0.675 | 0.825 | 0.925 | 1.000 | 0.900 | 0.950 |
| p012 | 0.125 | 0.075 | 0.250 | 0.375 | 0.475 | 0.675 | 0.850 | 1.000 | 0.950 | 1.000 | 0.950 | 0.250 | 0.275 | 0.325 | 0.250 | 0.450 | 0.550 | 0.625 | 0.700 | 0.875 | 0.900 | 0.950 | 0.375 | 0.450 | 0.450 | 0.450 | 0.575 | 0.625 | 0.850 | 0.850 | 0.925 | 0.950 | 0.875 |
| p013 | 0.025 | 0.025 | 0.025 | 0.125 | 0.225 | 0.275 | 0.625 | 0.725 | 0.900 | 0.925 | 0.950 | 0.000 | 0.125 | 0.150 | 0.175 | 0.250 | 0.350 | 0.575 | 0.775 | 0.925 | 0.875 | 0.975 | 0.375 | 0.325 | 0.450 | 0.450 | 0.600 | 0.650 | 0.700 | 0.775 | 0.925 | 0.800 | 0.800 |
| p014 | 0.050 | 0.025 | 0.100 | 0.100 | 0.225 | 0.500 | 0.625 | 0.825 | 0.975 | 0.975 | 1.000 | 0.050 | 0.075 | 0.100 | 0.250 | 0.300 | 0.500 | 0.625 | 0.800 | 0.950 | 0.975 | 0.950 | 0.100 | 0.025 | 0.300 | 0.125 | 0.225 | 0.250 | 0.575 | 0.650 | 0.750 | 0.750 | 0.800 |
| p015 | 0.075 | 0.050 | 0.100 | 0.175 | 0.350 | 0.375 | 0.675 | 0.825 | 0.950 | 0.975 | 1.000 | 0.125 | 0.075 | 0.075 | 0.350 | 0.225 | 0.425 | 0.525 | 0.650 | 0.750 | 0.900 | 0.900 | 0.275 | 0.225 | 0.300 | 0.350 | 0.375 | 0.425 | 0.500 | 0.550 | 0.550 | 0.550 | 0.625 |
| p016 | 0.100 | 0.050 | 0.100 | 0.175 | 0.275 | 0.475 | 0.625 | 0.800 | 0.900 | 1.000 | 1.000 | 0.050 | 0.150 | 0.175 | 0.175 | 0.225 | 0.425 | 0.625 | 0.825 | 0.950 | 0.950 | 0.975 | 0.375 | 0.450 | 0.500 | 0.525 | 0.625 | 0.800 | 0.800 | 0.850 | 0.975 | 0.950 | 0.975 |
| p019 | 0.050 | 0.025 | 0.100 | 0.075 | 0.225 | 0.525 | 0.775 | 0.950 | 0.975 | 1.000 | 1.000 | 0.075 | 0.050 | 0.150 | 0.225 | 0.300 | 0.475 | 0.725 | 0.900 | 0.950 | 0.975 | 1.000 | 0.350 | 0.300 | 0.375 | 0.400 | 0.475 | 0.475 | 0.725 | 0.850 | 0.825 | 0.825 | 0.925 |
| p020 | 0.275 | 0.275 | 0.325 | 0.350 | 0.400 | 0.600 | 0.725 | 0.750 | 0.875 | 0.900 | 0.875 | 0.275 | 0.350 | 0.425 | 0.450 | 0.525 | 0.650 | 0.675 | 0.775 | 0.900 | 0.850 | 0.875 | 0.200 | 0.400 | 0.200 | 0.400 | 0.400 | 0.375 | 0.400 | 0.375 | 0.400 | 0.475 | 0.400 |
| p021 | 0.025 | 0.025 | 0.050 | 0.100 | 0.175 | 0.325 | 0.450 | 0.700 | 0.850 | 0.950 | 1.000 | 0.025 | 0.050 | 0.050 | 0.050 | 0.075 | 0.200 | 0.250 | 0.450 | 0.600 | 0.875 | 0.875 | 0.175 | 0.275 | 0.275 | 0.300 | 0.425 | 0.525 | 0.525 | 0.550 | 0.650 | 0.825 | 0.750 |
| p022 | 0.050 | 0.050 | 0.100 | 0.175 | 0.325 | 0.475 | 0.600 | 0.775 | 0.950 | 1.000 | 1.000 | 0.050 | 0.075 | 0.125 | 0.150 | 0.350 | 0.450 | 0.575 | 0.775 | 0.875 | 1.000 | 0.950 | 0.225 | 0.200 | 0.225 | 0.300 | 0.400 | 0.575 | 0.725 | 0.750 | 0.800 | 0.900 | 0.900 |
| p023 | 0.075 | 0.025 | 0.125 | 0.200 | 0.325 | 0.525 | 0.750 | 0.900 | 0.975 | 1.000 | 1.000 | 0.150 | 0.175 | 0.075 | 0.250 | 0.325 | 0.500 | 0.675 | 0.850 | 0.925 | 0.950 | 1.000 | 0.200 | 0.200 | 0.150 | 0.250 | 0.375 | 0.425 | 0.550 | 0.675 | 0.700 | 0.750 | 0.800 |
| p024 | 0.100 | 0.175 | 0.150 | 0.200 | 0.275 | 0.550 | 0.800 | 0.925 | 0.975 | 0.975 | 1.000 | 0.125 | 0.100 | 0.200 | 0.300 | 0.350 | 0.450 | 0.725 | 0.825 | 0.950 | 0.975 | 0.975 | 0.250 | 0.300 | 0.275 | 0.375 | 0.300 | 0.400 | 0.550 | 0.675 | 0.725 | 0.725 | 0.800 |
| p025 | 0.200 | 0.175 | 0.275 | 0.225 | 0.425 | 0.500 | 0.600 | 0.700 | 0.750 | 0.900 | 0.950 | 0.175 | 0.225 | 0.225 | 0.325 | 0.325 | 0.425 | 0.400 | 0.725 | 0.900 | 0.900 | 0.850 | 0.450 | 0.425 | 0.375 | 0.425 | 0.500 | 0.475 | 0.450 | 0.575 | 0.550 | 0.600 | 0.700 |
| p026 | 0.075 | 0.075 | 0.050 | 0.075 | 0.225 | 0.475 | 0.700 | 0.875 | 0.975 | 1.000 | 1.000 | 0.150 | 0.250 | 0.225 | 0.300 | 0.400 | 0.525 | 0.675 | 0.800 | 0.875 | 0.875 | 0.975 | 0.200 | 0.300 | 0.175 | 0.300 | 0.350 | 0.400 | 0.525 | 0.500 | 0.675 | 0.700 | 0.725 |
| p027 | 0.075 | 0.175 | 0.200 | 0.300 | 0.325 | 0.500 | 0.750 | 0.925 | 0.925 | 1.000 | 1.000 | 0.175 | 0.275 | 0.300 | 0.500 | 0.550 | 0.625 | 0.700 | 0.875 | 0.875 | 0.975 | 1.000 | 0.200 | 0.350 | 0.375 | 0.525 | 0.600 | 0.700 | 0.850 | 0.850 | 0.825 | 0.900 | 0.850 |
| p028 | 0.075 | 0.025 | 0.025 | 0.150 | 0.250 | 0.275 | 0.550 | 0.700 | 0.850 | 0.975 | 0.975 | 0.025 | 0.100 | 0.100 | 0.175 | 0.225 | 0.225 | 0.425 | 0.675 | 0.850 | 0.900 | 0.900 | 0.325 | 0.250 | 0.375 | 0.325 | 0.425 | 0.550 | 0.725 | 0.675 | 0.750 | 0.800 | 0.850 |
| p029 | 0.000 | 0.050 | 0.050 | 0.075 | 0.225 | 0.250 | 0.650 | 0.675 | 0.825 | 0.900 | 0.950 | 0.025 | 0.050 | 0.100 | 0.125 | 0.175 | 0.300 | 0.500 | 0.725 | 0.825 | 0.900 | 0.875 | 0.125 | 0.150 | 0.200 | 0.175 | 0.250 | 0.350 | 0.525 | 0.575 | 0.675 | 0.775 | 0.700 |
| p030 | 0.350 | 0.375 | 0.400 | 0.525 | 0.450 | 0.650 | 0.850 | 0.850 | 1.000 | 0.950 | 1.000 | 0.325 | 0.450 | 0.400 | 0.550 | 0.600 | 0.600 | 0.725 | 0.725 | 0.750 | 0.875 | 0.875 | 0.625 | 0.550 | 0.575 | 0.650 | 0.700 | 0.675 | 0.700 | 0.825 | 0.800 | 1.000 | 0.850 |

## Fit a Logistic Function to the Identification Data¶

Logistic Function: $F(x;{\alpha},{\beta})=\frac{1}{1+exp(- {\beta}(x-{\alpha}))}$

In [84]:

```
def logistic(x,a,b):
    y = 1 / (1 + np.exp(-b*(x-a)))
    return y
```

Intercept and slope of the best-fitting logistic function are determined interatively by using scipy's curve\_fit function.

In [85]:

```
def fitFunc(func,ydata):
    # for all designs in this study, there are 11 morphing steps, scaled between 0 and 1
    x = np.arange(0,len(ydata)/10.,0.1)
    # the scipy curvefit function is used here
    popt, pcov = curve_fit(func, x, ydata, maxfev=100000)

    intercept = popt[0] # intercept
    slope = popt[1]     # slope
    
    return intercept,slope
```

Example:

In [86]:

```
exampleData = [0,0,0.2,0.3,0.3,0.5,0.7,0.7,0.8,1,1]
```

In [87]:

```
fitFunc(logistic,exampleData)
```

Out[87]:

```
(0.49999999998800071, 6.0566394648348618)
```

These parameters and the definition of the logistic function can be used to generate the
logistic curve for this data set:

In [89]:

```
# get parameters
intercept,slope=fitFunc(logistic,exampleData)
# make fine-grained logistic function (100 steps)
exampleLog = []
for x in np.arange(0,1,0.01):
    exampleLog.append(logistic(x,intercept,slope))
# plot the raw data and adapt x axis to match the
# fine-grained resolution of the fitted function
plot(np.arange(0,101,10),exampleData,'o')
# plot the logistic function
plot(exampleLog)
# label x-axis
xticks(np.arange(0,101,10),np.arange(0,1.01,0.1))
# scale x- and y-axis
ylim(-0.1,1.1); xlim(-1,101)
sns.despine()
show()
```

## Recaling the data before fitting¶

The steepest point of the logistic curve is at 0.5 or 50%. This means that if a response is strongly shifted/biased in a way that it never crosses 50%, this will acutually lead to shallow slopes. Since we do not want the overall response level to influence our estimate of the slope parameter, we first rescale the data so that the lowest value becomes 0% and the highest value 100%. Thereby, even strongly shifted responses (as in Experiment 2) will be treated in a fair way when estimating the slope parameter.

### Short Example of the Issue:¶

If the responses never cross 50%, the logistic function becomes wide and the 50% value is acutally somewhere outside the response range. Therfore, the steepest point in the actual data is not found and secondly, the slope of the fitted function is very shallow because the function needs to be so wide to accomodate the data.

In [90]:

```
exampleData = [0,0,0,0.1,0.1,0.1,0.3,0.3,0.4,0.4,0.4]

intercept,slope=fitFunc(logistic,exampleData)
exampleLog = []
for x in np.arange(0,3,0.01):
    exampleLog.append(logistic(x,intercept,slope))
plot(np.arange(0,101,10),exampleData,'o')
plot(exampleLog)
xticks(np.arange(0,101,10),np.arange(0,1.01,0.1))
ylim(-0.1,1.1); xlim(-1,101)
sns.despine()
show()
```

The larger picture (the threshold is actually outside the response range):

In [91]:

```
plot(np.arange(0,101,10),exampleData,'o')
plot(exampleLog)
xticks(np.arange(0,301,50),np.arange(0,3.01,0.5))
ylim(-0.1,1.1); xlim(-1,301)
sns.despine()
show()
```

## Rescaling the data to their range¶

The logistic function will be fitted to these rescaled data

In [92]:

```
def transformData(thisList):
    # the starting values are choses in such a way,
    # that the lowest value can only decrease (starts high)
    # and the highest value can only increase (starts low)
    lowest = 100
    highest = 0

    # we move through the list and update
    # the highest and lowest value, once we
    # encounter an entry that is higher or lower, respectively
    for x in thisList:
        if x < lowest:
            lowest = x
        if x > highest:       
            highest = x
    
    # the difference between highest and lowest is the span
    span = highest-lowest

    # the new rescaled data are written
    newList = []
    for x in thisList:
        # we get the distance from the lowest point and
        # rescale by the span of highest-lowest
        # e.g. the lowest point is 5 the highest is 10
        # the value 6 will be rescaled to (6-5)/5 = 1/5 = 20%
        newPoint = (float(x)-lowest)/span
        newList.append(newPoint)
        
    return newList,lowest,span
```

### Transforming the rescaled data back to their original space¶

This will be used to transform the fitted logistic function back to the native space

In [93]:

```
def transformBack(newList,lowest,span):
    oldList = []
    for x in newList:
        oldPoint = float(x)*span+lowest
        oldList.append(oldPoint)
    return oldList
```

Example:

In [94]:

```
plot(exampleData)
plot(transformData(exampleData)[0])
ylim(-0.1,1.1);xlim(-0.1,10.1)
sns.despine()
show()
```

Checking if scaling back restores the original data:

In [96]:

```
newX,lowest,span=transformData(exampleData)
assert transformBack(newX,lowest,span) == exampleData, "rescaling produces an error!"
```

### The logistic is fitted to the rescaled data and transformed to the range of the original data in the aftermath¶

In [121]:

```
def rescaledFunc(thisData):
    #rescale the data
    newData,lowest,span = transformData(thisData)
    # fit a logistic function to the rescaled data
    # and extract intercept and slope of the function
    intercept,slope=fitFunc(logistic,newData)
    # use these parameters to make a logistic curve
    logData = []
    for x in np.arange(0,1.0001,0.0001):
        logData.append(logistic(x,intercept,slope))
    
    # scale the logistic curve back to the original dimensions
    nativeLog = transformBack(logData,lowest,span)
    
    return nativeLog
```

Fitting to the original data for comparison:

In [122]:

```
def originalFunc(thisData):
    intercept,slope = fitFunc(logistic,thisData)
    rawLog = []
    for x in np.arange(0,1.0001,0.0001):
        rawLog.append(logistic(x,intercept,slope))
    return rawLog
```

### Checking the effect this has with an example:¶

We see that when we rescale the data, fit the function and scale the function back to the native space,
we get a much better estimate of the slope and also - importantly- the position of the intercept in our acutal data; before, the intercept was located bascially in an extrapolated data space (which could be thought of as a "caricature", hence more than 100% fear/anger), far away on the x-axis.

In [123]:

```
plot(np.arange(0,10001,1000),exampleData,'o')
plot(rescaledFunc(exampleData))
plot(originalFunc(exampleData))
xticks(np.arange(0,10001,1000),np.arange(0,1.1,0.1))
ylim(0,1)
sns.despine()
show()
```

## Putting it all together for our acutal data:¶

### Get the logistic function for all participants¶

In [124]:

```
def logDict(df,cond,func=rescaledFunc):
    d={}
    for p in df.index:
        d[p]= func(df.ix[p][cond])
    return d
```

In [125]:

```
def showCurve(curveDict,rgb,myTitle,myX,xLab,yLab):
    for p in curveDict:
        plot(curveDict[p],
             color=rgb,
             alpha=0.5,
             linewidth=4
            )
    title(myTitle)
    xticks(myX[0],myX[1])
    xlabel(xLab)
    ylabel(yLab)
```

In [126]:

```
# global color palette
myPalette=['#ee4035','#4f5b66','#325da7','#da6b30']
```

In [127]:

```
myTicks = ['0%','10%','20%','30%','40%','50%','60%','70%','80%','90%','100%']
```

### Spaghetti plots of all raw data¶

In [128]:

```
def showRaw(df,condOrder,colors=myPalette):
    for index,cond in enumerate(condOrder):
        print cond
        for p in df[cond].index:
            plot(df[cond].ix[p],
                 alpha=0.5,
                 linewidth=4,
                 c=colors[index]
                )
        title(cond)
        xticks(np.arange(11),myTicks)
        yticks(np.arange(0,1.1,0.1),myTicks)
        xlabel('morphing grade')
        ylabel('% angry responses')     
        sns.despine()
        savefig('raw'+str(cond)+'.png',dpi=600)
        show()
```

In [105]:

```
showRaw(exp1Results,['whole','eyes','mouth'])
```

```
whole
```

```
eyes
```

```
mouth
```

### Spaghetti plots of all fitted functions¶

In [108]:

```
def showAll(df,condOrder,
            colors=myPalette,
            func=rescaledFunc):
    for index,cond in enumerate(condOrder):
        print cond
        condDict = logDict(df,cond,func=func)
        showCurve(condDict,
                  colors[index],
                  cond,
                  [np.arange(0,10001,1000),myTicks],
                  'morphing grade',
                  '% angry'
                 )
        sns.despine()
        yticks(np.arange(0,1.1,0.1),myTicks)
        savefig('fitted'+str(cond)+'.png',dpi=600)
        show()
```

In [109]:

```
showAll(exp1Results,['whole','eyes','mouth'])
```

```
whole
```

```
eyes
```

```
mouth
```

And with the unscaled data for comparison:

In [110]:

```
showAll(exp1Results,
        ['whole','eyes','mouth'],
        func=originalFunc)
```

```
whole
```

```
eyes
```

```
mouth
```

Overall, a first visual inspection shows that for Experiment 1, it does not seem to make much of a difference whether we rescale the data or not. Since each and every participants responses pass through 50% for all conditions, we would considered both approaches valid.

## Computing the first derivative of the functions¶

In [129]:

```
def getDeriv(thisList):
    derivList = []
    for index,x in enumerate(thisList):
        
        try:
            # the following is basically the formula for computing the derivative,
            # but the parameter h is defined by the resolution of the logisitic function
            # we have generated
            thisSlope = (float(thisList[index+1])-thisList[index])/(1./( len(thisList)-1 ))
            derivList.append(thisSlope)
        # this should not work with the very last item (but only with that item!)
        except:
            # if it doesnt work in any other case, throw and exception
            assert index == len(thisList)-1, 'something went wrong'
    return derivList
```

In [130]:

```
def showDeriv(curveDict,rgb,myTitle,myX,xLab,yLab):
    for p in curveDict:
        plot(
            getDeriv(curveDict[p]),
            color=rgb,
            alpha=0.5,
            linewidth=4
            )
    title(myTitle)
    xticks(myX[0],myX[1])
    xlabel(xLab)
    ylabel(yLab)
    ylim(0,3.75)
    sns.despine()
    savefig('deriv'+myTitle+'.png',dpi=600)
    show()
```

In [131]:

```
def showAllDeriv(df,condOrder,colors=myPalette,func=rescaledFunc):
    for index,cond in enumerate(condOrder):
        condDict = logDict(df,cond,func=func)
        showDeriv(condDict,
                  colors[index],
                  cond,
                  [np.arange(0,10001,1000),myTicks],
                  'morphing grade',
                  'steepness of slope'
                 )
```

In [132]:

```
showAllDeriv(exp1Results,['whole','eyes','mouth'])
```

Comparison of unscaled data:

In [133]:

```
showAllDeriv(exp1Results,['whole','eyes','mouth'],func=originalFunc)
```

### Extract threshold and slope parameters¶

The value of the first derivative (on the y-axis) is the slope parameter; its position on the x-axis is the x-intercept. We extract the point with the highest value.

In [134]:

```
def getPeak(d,p):
    # starting values are set
    highestVal = -999
    highestPos = -999
    for index,i in enumerate(getDeriv(d[p])):
        if i > highestVal:
            highestVal = i
            highestPos = index
    return highestPos,highestVal
```

In [136]:

```
def peakDict(df,func=rescaledFunc):
    d = {}
    for index,cond in enumerate(df.columns.levels[0]):
        d[cond] = {}
        condDict = logDict(df,cond,func=func)
        for p in condDict:
            # get slope and x-threshold form the first derivative
            threshold,slope = getPeak(condDict,p)
            # get value on y-threshold from the original logistic
            # function; this is the value of the logistic function
            # at the point where the value of its derivative is highest ( and this is simply the threshold)
            # we consider this the 'bias' or y-threshold - at which height (y-axis)
            # is the threshold located? i.e. how much is the curve shifted up or down.
            bias = condDict[p][threshold]
            # rescale threshold to value between 0 and 1
            threshold = float(threshold)/len(condDict[p])

            # we call the 
            d[cond][p] = {'slope':slope,'threshold':threshold,'bias':bias}
        
    return d
```

In [137]:

```
def peakDf(d):
    for cond in d:
        thisDf = pd.DataFrame(d[cond]).T
        thisDf.index=[[cond]*len(thisDf),thisDf.index]
        try:
            bigDf = pd.concat([bigDf,thisDf])
        except:
            bigDf = thisDf

    return bigDf.unstack(0)
```

In [138]:

```
exp1LogFuncTable = peakDf(peakDict(exp1Results))
```

In [139]:

```
cols = exp1LogFuncTable.columns.tolist()
# reordering the columns for clarity
cols = [ cols[8],cols[6],cols[7], cols[2],cols[0],cols[1], cols[5],cols[3],cols[4]]
exp1LogFuncTable = exp1LogFuncTable[cols]
```

In [140]:

```
exp1LogFuncTable
```

Out[140]:

|  | threshold | | | bias | | | slope | | |
| --- | --- | --- | --- | --- | --- | --- | --- | --- | --- |
|  | whole | eyes | mouth | whole | eyes | mouth | whole | eyes | mouth |
| p001 | 0.511649 | 0.498050 | 0.415858 | 0.562425 | 0.512472 | 0.587438 | 2.296437 | 1.705580 | 0.880567 |
| p002 | 0.555444 | 0.485551 | 0.480352 | 0.487459 | 0.574928 | 0.537403 | 1.821712 | 1.517784 | 1.353848 |
| p003 | 0.459554 | 0.369063 | 0.476552 | 0.562428 | 0.637425 | 0.549942 | 2.412706 | 1.066301 | 0.742892 |
| p004 | 0.519048 | 0.542646 | 0.491251 | 0.462445 | 0.549984 | 0.424957 | 1.903614 | 0.962355 | 0.557786 |
| p005 | 0.575942 | 0.534947 | 0.616538 | 0.424967 | 0.449890 | 0.424998 | 1.705129 | 1.418296 | 0.624480 |
| p006 | 0.516948 | 0.531547 | 0.532147 | 0.512433 | 0.524891 | 0.524908 | 3.508805 | 2.392012 | 1.173356 |
| p007 | 0.582242 | 0.558844 | 0.474453 | 0.537492 | 0.524888 | 0.662419 | 2.524960 | 1.625036 | 0.826195 |
| p008 | 0.511649 | 0.546145 | 0.546345 | 0.587446 | 0.562433 | 0.774938 | 1.564888 | 1.742184 | 0.788172 |
| p009 | 0.549045 | 0.539546 | 0.467953 | 0.499931 | 0.499802 | 0.474940 | 2.453070 | 2.145561 | 1.910188 |
| p010 | 0.520748 | 0.514649 | 0.507849 | 0.574955 | 0.524972 | 0.599949 | 1.867616 | 0.922131 | 0.683058 |
| p011 | 0.524248 | 0.602640 | 0.437256 | 0.512259 | 0.524978 | 0.624947 | 3.026834 | 2.461428 | 1.302983 |
| p012 | 0.411559 | 0.569043 | 0.486251 | 0.537494 | 0.599946 | 0.662425 | 1.952323 | 1.285799 | 1.197446 |
| p013 | 0.561944 | 0.537546 | 0.485351 | 0.487397 | 0.487459 | 0.624956 | 2.297931 | 1.750875 | 0.813170 |
| p014 | 0.529247 | 0.512149 | 0.525947 | 0.512308 | 0.512414 | 0.412421 | 2.316599 | 1.873855 | 1.407359 |
| p015 | 0.531547 | 0.556144 | 0.482752 | 0.524853 | 0.487374 | 0.424949 | 2.071867 | 1.402491 | 0.545118 |
| p016 | 0.536146 | 0.534447 | 0.431457 | 0.524812 | 0.512359 | 0.674991 | 1.985029 | 2.087575 | 1.024071 |
| p019 | 0.499950 | 0.503750 | 0.539246 | 0.512362 | 0.524999 | 0.612431 | 3.161677 | 2.150282 | 1.201446 |
| p020 | 0.513949 | 0.452155 | 0.313269 | 0.587388 | 0.587415 | 0.337499 | 1.442985 | 0.944290 | 0.205535 |
| p021 | 0.608239 | 0.691731 | 0.539546 | 0.512363 | 0.449848 | 0.499928 | 2.123432 | 2.104248 | 0.787359 |
| p022 | 0.533947 | 0.544346 | 0.499650 | 0.524911 | 0.524954 | 0.550000 | 1.937535 | 1.765065 | 1.495067 |
| p023 | 0.483352 | 0.515748 | 0.527547 | 0.512424 | 0.537445 | 0.474896 | 2.238710 | 1.855976 | 1.168153 |
| p024 | 0.500850 | 0.506449 | 0.585841 | 0.549883 | 0.537452 | 0.524951 | 2.671557 | 1.814443 | 1.142936 |
| p025 | 0.567743 | 0.590141 | 0.671933 | 0.562395 | 0.537374 | 0.537470 | 1.233148 | 1.530025 | 0.350917 |
| p026 | 0.527447 | 0.519748 | 0.536846 | 0.524907 | 0.562467 | 0.449970 | 2.797672 | 1.391004 | 0.837735 |
| p027 | 0.487951 | 0.448155 | 0.342766 | 0.537468 | 0.587389 | 0.549953 | 1.874006 | 1.187727 | 1.222959 |
| p028 | 0.581842 | 0.589341 | 0.503150 | 0.499917 | 0.462392 | 0.549992 | 2.034048 | 1.952930 | 0.997454 |
| p029 | 0.565043 | 0.569843 | 0.570343 | 0.474805 | 0.462486 | 0.449876 | 2.034426 | 2.076668 | 1.262053 |
| p030 | 0.509949 | 0.444056 | 0.657434 | 0.674907 | 0.599944 | 0.774992 | 1.415442 | 0.696189 | 0.500008 |

save to csv

In [141]:

```
exp1LogFuncTable.to_csv('exp1fittable.txt')
```

In [142]:

```
exp1LogFuncTable.describe()
```

Out[142]:

|  | threshold | | | bias | | | slope | | |
| --- | --- | --- | --- | --- | --- | --- | --- | --- | --- |
|  | whole | eyes | mouth | whole | eyes | mouth | whole | eyes | mouth |
| count | 28.000000 | 28.000000 | 28.000000 | 28.000000 | 28.000000 | 28.000000 | 28.000000 | 28.000000 | 28.000000 |
| mean | 0.527758 | 0.528872 | 0.505210 | 0.528030 | 0.530728 | 0.546376 | 2.166934 | 1.636718 | 0.964368 |
| std | 0.040681 | 0.059359 | 0.078724 | 0.046852 | 0.047211 | 0.106658 | 0.530281 | 0.465002 | 0.380377 |
| min | 0.411559 | 0.369063 | 0.313269 | 0.424967 | 0.449848 | 0.337499 | 1.233148 | 0.696189 | 0.205535 |
| 25% | 0.511224 | 0.505774 | 0.476027 | 0.509177 | 0.509219 | 0.468665 | 1.872408 | 1.364703 | 0.727933 |
| 50% | 0.525847 | 0.534697 | 0.501400 | 0.524832 | 0.524975 | 0.543706 | 2.053146 | 1.723882 | 0.939010 |
| 75% | 0.557069 | 0.556819 | 0.539321 | 0.553011 | 0.562441 | 0.615560 | 2.422797 | 1.983864 | 1.206824 |
| max | 0.608239 | 0.691731 | 0.671933 | 0.674907 | 0.637425 | 0.774992 | 3.508805 | 2.461428 | 1.910188 |

### violin plots of the curve parameters¶

In [143]:

```
for index, metric in enumerate(['threshold','bias','slope']):
    sns.violinplot(exp1LogFuncTable[metric],
                   inner='box',bw=.3, cut=.3,lw=.3,
                   color=myPalette)
    title(metric)
    if index <2:
        ylim(0,1)
        yticks(np.arange(0,1.1,0.1),myTicks)
    sns.despine()
    savefig('violin_'+metric+'.png',dpi=600)
    show()
```

### some more elaborate line plots for 100% transparent visualisation, taking repeated measures into account¶

In [145]:

```
def plot_curvestats(df,conds,plotname,yname,
                    lw=5,fasp1=6,fasp2=4,ymin=0,ymax=0):
    
    # figure proportions
    f = figure(figsize=(fasp1,fasp2))
    ax = plt.subplot(111)    
    
    # make single participant lineplots
    for p in range(len(df.index)):
        # get the values of all conditions
        this_values = []
        for c in conds:
            this_values.append(df.ix[p][c])
        i = 0
        while i < len(conds)-1:
        # plot all condition
            if this_values[i] > this_values[i+1]:
                plot(
                     [i+.015,i+1],
                     [this_values[i],this_values[i+1]],
                     alpha=0.2,
                     linewidth=lw,
                     c='k',
                     zorder=0
                     )
            if this_values[i] <= this_values[i+1]:
                plot(
                     [i+.015,i+1],
                     [this_values[i],this_values[i+1]],
                     alpha=0.2,
                     linewidth=lw,
                     c='b',
                     zorder=0
                     )
            i+=1
            
    # make aggregated plots (mean + ci)
    
    i = 0
    for c in conds:
        errorbar(i,
                 df[c].mean(),
                 yerr=df[c].std(ddof=1)/sqrt(len(df.index))*1.96,
                 ecolor="r",
                 elinewidth=5,
                 c='r',
                 capsize=5,
                 capthick=5,
                 zorder =1
                 )
        i+=1

    plt.xlim(-0.1,i-0.9)
    plt.xticks(range(len(conds)),conds)
    plt.title(plotname)
    if ymin == 0 and ymax == 0:
        ymin, ymax = ylim() 
    else:
        yticks(np.arange(0,1.1,0.1),myTicks)
    plt.ylim(ymin,ymax)
    plt.xlabel("Face Conditions\n(Raw Data with 95% CI)")
    plt.ylabel(yname)
    
    sns.despine()
    
    plt.savefig((plotname+str(conds)+'.png'),dpi=600)  
    plt.show()
```

In [146]:

```
for index, metric in enumerate(['threshold','bias','slope']):
    ymax = 0
    if index <2:
        ymax=1
        
    plot_curvestats(exp1LogFuncTable[metric],['whole','eyes','mouth'],metric,'y',
                   lw=5,fasp1=6,fasp2=4,ymin=0,ymax=ymax)
```

### t-tests for comparing everything with everything¶

In [147]:

```
from scipy import stats
```

In [148]:

```
def infStats(df,my_alpha):
    for c1 in df.columns:
        for c2 in df.columns:
            if c1 != c2:
                print c1,c2
                t,p = stats.ttest_rel(df[c1],df[c2])
                w,wp = stats.wilcoxon(df[c1],df[c2])
                if p < my_alpha and wp < my_alpha:
                    sig = '*'
                else:
                    sig = 'n.s.'
                    
                print 't:',round(t,2),'p:',p,'\tw:',round(w,2),'wp:',wp, sig
```

In [149]:

```
infStats(exp1LogFuncTable['threshold'],0.05)
```

```
whole eyes
t: -0.12 p: 0.907714277369 	w: 196.0 wp: 0.873353767434 n.s.
whole mouth
t: 1.49 p: 0.146924077385 	w: 143.0 wp: 0.171849400485 n.s.
eyes whole
t: 0.12 p: 0.907714277369 	w: 196.0 wp: 0.873353767434 n.s.
eyes mouth
t: 1.45 p: 0.157824063243 	w: 136.0 wp: 0.127088240298 n.s.
mouth whole
t: -1.49 p: 0.146924077385 	w: 143.0 wp: 0.171849400485 n.s.
mouth eyes
t: -1.45 p: 0.157824063243 	w: 136.0 wp: 0.127088240298 n.s.
```

In [150]:

```
infStats(exp1LogFuncTable['bias'],0.05)
```

```
whole eyes
t: -0.33 p: 0.741298236707 	w: 192.0 wp: 0.802211794992 n.s.
whole mouth
t: -1.05 p: 0.304216881588 	w: 146.0 wp: 0.194297254432 n.s.
eyes whole
t: 0.33 p: 0.741298236707 	w: 192.0 wp: 0.802211794992 n.s.
eyes mouth
t: -0.79 p: 0.43697932688 	w: 167.0 wp: 0.412346527888 n.s.
mouth whole
t: 1.05 p: 0.304216881588 	w: 146.0 wp: 0.194297254432 n.s.
mouth eyes
t: 0.79 p: 0.43697932688 	w: 167.0 wp: 0.412346527888 n.s.
```

In [151]:

```
infStats(exp1LogFuncTable['slope'],0.05)
```

```
whole eyes
t: 6.28 p: 1.01992102337e-06 	w: 20.0 wp: 3.08403852945e-05 *
whole mouth
t: 13.11 p: 3.16826357794e-13 	w: 0.0 wp: 3.78961944158e-06 *
eyes whole
t: -6.28 p: 1.01992102337e-06 	w: 20.0 wp: 3.08403852945e-05 *
eyes mouth
t: 9.36 p: 5.6957678885e-10 	w: 1.0 wp: 4.22840884725e-06 *
mouth whole
t: -13.11 p: 3.16826357794e-13 	w: 0.0 wp: 3.78961944158e-06 *
mouth eyes
t: -9.36 p: 5.6957678885e-10 	w: 1.0 wp: 4.22840884725e-06 *
```

## Spaghetti plots with threshold overlaid¶

In [152]:

```
def showPeak(curveDict,myColor):
    for p in curveDict:
        highestPos,highestVal = getPeak(curveDict,p)
        plot(highestPos,highestVal,
             'o',
             color=myColor,
             alpha=0.8
            )
    # dont show so it will merge with subsequent plot
    # show()
```

In [153]:

```
def allPeaks(df,condOrder,colors=myPalette,func=rescaledFunc):
    for index,cond in enumerate(condOrder):
        condDict = logDict(df,cond,func=func)
        showPeak(condDict,colors[index])
        showDeriv(condDict,
                  colors[index],
                  cond,
                  [np.arange(0,10001,1000),myTicks],
                  'morphing grade',
                  'steepness of slope')
```

In [154]:

```
allPeaks(exp1Results,['whole','eyes','mouth'])
```

In [155]:

```
def allLogWithPeaks(df,condOrder,colors=myPalette,func=rescaledFunc):
    
    for index,cond in enumerate(condOrder):
        condDict = logDict(df,cond,func=func)
        
        for p in condDict:
            highestPos,highestVal = getPeak(condDict,p)
            
            plot(condDict[p],
                 colors[index],
                 alpha=0.5,
                 linewidth=3)

            plot(highestPos,condDict[p][highestPos],
                 'o',
                 color=colors[index],
                 alpha=0.8
                )

        title(cond)
        xticks(np.arange(0,10001,1000),myTicks)
        yticks(np.arange(0,1.1,0.1),myTicks)
        xlabel('morphing grade',)
        ylabel('% angry responses')
        sns.despine()
        savefig('fitpoint'+cond+'.png',dpi=600)
        show()
```

In [156]:

```
allLogWithPeaks(exp1Results,['whole','eyes','mouth'])
```

The following plots nicely illustrate why allowing the threshold to vary on the y-axis by rescaling is so critical. Otherwise, the threshold is located at 50% always. This is fine for the current data (experiment 1) but problematic for experiment 2 (see below).

In [157]:

```
allLogWithPeaks(exp1Results,['whole','eyes','mouth'],func=originalFunc)
```

# Experiment 2 (Composite Faces)¶

In [158]:

```
i = 0
for df in dflist:
    print i,':',df
    i+=1
```

```
0 : Exp1AvgResults.txt
1 : Exp1AvgResultsAll.txt
2 : Exp1Pt1MainResults.txt
3 : Exp1Pt1ResultsAll.txt
4 : Exp1Pt2MainResults.txt
5 : Exp1Pt2ResultsAll.txt
6 : Exp1RtMedian.txt
7 : Exp1RtMedianAll.txt
8 : Exp2AvgResults.txt
9 : Exp2AvgResultsAll.txt
10 : Exp2Pt1Results.txt
11 : Exp2Pt1ResultsAll.txt
12 : Exp2Pt2Results.txt
13 : Exp2Pt2ResultsAll.txt
14 : Exp2RtMedian.txt
15 : Exp2RtMedianAll.txt
16 : exp1Pt1Logistic.txt
17 : exp1Pt2Logistic.txt
18 : exp1fittable.txt
19 : exp1pt1Logistic.txt
20 : exp1pt2Logistic.txt
21 : exp2Pt1Logistic.txt
22 : exp2Pt2Logistic.txt
23 : exp2fittable.txt
24 : exp2pt1Logistic.txt
25 : exp2pt2Logistic.txt
```

In [159]:

```
exp2Results = pd.read_csv(dflist[8],
                 header=[0,1],
                 index_col=[0])/100
```

In [160]:

```
exp2Results
```

Out[160]:

|  | fearLOW | | | | | | | | | | | angerLOW | | | | | | | | | | | fearUP | | | | | | | | | | | angerUP | | | | | | | | | | |
| --- | --- | --- | --- | --- | --- | --- | --- | --- | --- | --- | --- | --- | --- | --- | --- | --- | --- | --- | --- | --- | --- | --- | --- | --- | --- | --- | --- | --- | --- | --- | --- | --- | --- | --- | --- | --- | --- | --- | --- | --- | --- | --- | --- | --- |
| grade | m00 | m01 | m02 | m03 | m04 | m05 | m06 | m07 | m08 | m09 | m10 | m00 | m01 | m02 | m03 | m04 | m05 | m06 | m07 | m08 | m09 | m10 | m00 | m01 | m02 | m03 | m04 | m05 | m06 | m07 | m08 | m09 | m10 | m00 | m01 | m02 | m03 | m04 | m05 | m06 | m07 | m08 | m09 | m10 |
| p |  |  |  |  |  |  |  |  |  |  |  |  |  |  |  |  |  |  |  |  |  |  |  |  |  |  |  |  |  |  |  |  |  |  |  |  |  |  |  |  |  |  |  |  |
| p001 | 0.150 | 0.250 | 0.425 | 0.400 | 0.425 | 0.500 | 0.550 | 0.625 | 0.775 | 0.775 | 0.800 | 0.250 | 0.325 | 0.325 | 0.450 | 0.375 | 0.525 | 0.625 | 0.675 | 0.750 | 0.750 | 0.875 | 0.350 | 0.325 | 0.425 | 0.400 | 0.425 | 0.450 | 0.425 | 0.425 | 0.450 | 0.525 | 0.500 | 0.575 | 0.425 | 0.550 | 0.575 | 0.625 | 0.500 | 0.575 | 0.575 | 0.675 | 0.600 | 0.700 |
| p002 | 0.400 | 0.275 | 0.575 | 0.425 | 0.525 | 0.500 | 0.600 | 0.875 | 0.800 | 0.850 | 0.875 | 0.275 | 0.525 | 0.525 | 0.525 | 0.600 | 0.575 | 0.750 | 0.875 | 0.675 | 0.825 | 0.825 | 0.400 | 0.400 | 0.400 | 0.350 | 0.500 | 0.425 | 0.425 | 0.450 | 0.475 | 0.475 | 0.500 | 0.675 | 0.725 | 0.800 | 0.725 | 0.675 | 0.725 | 0.775 | 0.825 | 0.850 | 0.950 | 0.900 |
| p003 | 0.100 | 0.075 | 0.150 | 0.100 | 0.125 | 0.350 | 0.525 | 0.650 | 0.900 | 0.900 | 0.975 | 0.225 | 0.100 | 0.225 | 0.200 | 0.400 | 0.575 | 0.825 | 0.850 | 0.925 | 0.950 | 0.950 | 0.325 | 0.300 | 0.350 | 0.300 | 0.325 | 0.425 | 0.450 | 0.575 | 0.475 | 0.525 | 0.525 | 0.625 | 0.650 | 0.525 | 0.600 | 0.675 | 0.625 | 0.575 | 0.700 | 0.725 | 0.675 | 0.750 |
| p004 | 0.125 | 0.175 | 0.175 | 0.250 | 0.250 | 0.350 | 0.450 | 0.600 | 0.425 | 0.525 | 0.600 | 0.350 | 0.375 | 0.475 | 0.600 | 0.575 | 0.550 | 0.650 | 0.650 | 0.775 | 0.850 | 0.850 | 0.375 | 0.225 | 0.375 | 0.425 | 0.450 | 0.475 | 0.600 | 0.550 | 0.625 | 0.500 | 0.575 | 0.575 | 0.650 | 0.600 | 0.600 | 0.700 | 0.750 | 0.850 | 0.825 | 0.850 | 0.850 | 0.800 |
| p005 | 0.025 | 0.125 | 0.175 | 0.200 | 0.250 | 0.350 | 0.525 | 0.700 | 0.825 | 0.775 | 0.925 | 0.175 | 0.225 | 0.200 | 0.250 | 0.425 | 0.550 | 0.700 | 0.900 | 0.925 | 0.925 | 0.975 | 0.050 | 0.050 | 0.075 | 0.075 | 0.150 | 0.125 | 0.125 | 0.200 | 0.225 | 0.150 | 0.100 | 0.925 | 0.900 | 0.900 | 0.950 | 0.925 | 0.950 | 0.925 | 0.925 | 0.975 | 1.000 | 1.000 |
| p006 | 0.400 | 0.325 | 0.425 | 0.375 | 0.500 | 0.500 | 0.525 | 0.650 | 0.750 | 0.650 | 0.775 | 0.325 | 0.350 | 0.375 | 0.425 | 0.575 | 0.600 | 0.650 | 0.725 | 0.675 | 0.775 | 0.975 | 0.375 | 0.450 | 0.475 | 0.600 | 0.525 | 0.400 | 0.550 | 0.675 | 0.500 | 0.500 | 0.375 | 0.625 | 0.725 | 0.600 | 0.650 | 0.600 | 0.775 | 0.575 | 0.775 | 0.675 | 0.725 | 0.675 |
| p007 | 0.175 | 0.225 | 0.225 | 0.250 | 0.225 | 0.300 | 0.500 | 0.550 | 0.725 | 0.775 | 0.850 | 0.300 | 0.375 | 0.325 | 0.325 | 0.375 | 0.450 | 0.675 | 0.900 | 0.800 | 0.825 | 0.825 | 0.250 | 0.175 | 0.300 | 0.200 | 0.225 | 0.250 | 0.200 | 0.375 | 0.300 | 0.350 | 0.375 | 0.925 | 0.850 | 0.825 | 0.900 | 0.875 | 0.850 | 0.950 | 0.875 | 0.925 | 0.925 | 0.850 |
| p008 | 0.225 | 0.250 | 0.275 | 0.300 | 0.325 | 0.400 | 0.500 | 0.500 | 0.600 | 0.650 | 0.725 | 0.225 | 0.300 | 0.350 | 0.425 | 0.600 | 0.650 | 0.650 | 0.675 | 0.750 | 0.825 | 0.700 | 0.375 | 0.425 | 0.300 | 0.425 | 0.525 | 0.450 | 0.550 | 0.675 | 0.625 | 0.550 | 0.750 | 0.625 | 0.575 | 0.700 | 0.650 | 0.700 | 0.775 | 0.800 | 0.775 | 0.925 | 0.800 | 0.875 |
| p009 | 0.425 | 0.350 | 0.400 | 0.400 | 0.625 | 0.400 | 0.500 | 0.625 | 0.700 | 0.750 | 0.825 | 0.325 | 0.450 | 0.400 | 0.475 | 0.425 | 0.500 | 0.600 | 0.675 | 0.875 | 0.775 | 0.775 | 0.375 | 0.475 | 0.525 | 0.425 | 0.450 | 0.475 | 0.500 | 0.450 | 0.500 | 0.450 | 0.475 | 0.700 | 0.700 | 0.750 | 0.700 | 0.775 | 0.625 | 0.650 | 0.625 | 0.650 | 0.775 | 0.650 |
| p010 | 0.075 | 0.150 | 0.225 | 0.225 | 0.250 | 0.500 | 0.550 | 0.525 | 0.825 | 0.825 | 0.875 | 0.325 | 0.100 | 0.275 | 0.300 | 0.350 | 0.525 | 0.700 | 0.775 | 0.825 | 0.850 | 0.950 | 0.175 | 0.125 | 0.125 | 0.200 | 0.150 | 0.225 | 0.250 | 0.325 | 0.200 | 0.300 | 0.425 | 0.550 | 0.675 | 0.575 | 0.700 | 0.575 | 0.750 | 0.725 | 0.800 | 0.725 | 0.800 | 0.900 |
| p011 | 0.150 | 0.075 | 0.100 | 0.200 | 0.300 | 0.375 | 0.550 | 0.800 | 0.700 | 0.825 | 0.925 | 0.075 | 0.175 | 0.200 | 0.200 | 0.300 | 0.400 | 0.600 | 0.725 | 0.825 | 0.900 | 0.950 | 0.300 | 0.375 | 0.300 | 0.425 | 0.500 | 0.600 | 0.650 | 0.700 | 0.750 | 0.850 | 0.825 | 0.475 | 0.375 | 0.450 | 0.350 | 0.600 | 0.600 | 0.725 | 0.825 | 0.825 | 0.825 | 0.800 |
| p012 | 0.075 | 0.125 | 0.100 | 0.175 | 0.225 | 0.400 | 0.500 | 0.625 | 0.600 | 0.850 | 0.925 | 0.225 | 0.225 | 0.300 | 0.275 | 0.425 | 0.475 | 0.650 | 0.675 | 0.925 | 0.925 | 0.950 | 0.125 | 0.150 | 0.150 | 0.075 | 0.150 | 0.200 | 0.225 | 0.400 | 0.350 | 0.400 | 0.425 | 0.575 | 0.550 | 0.475 | 0.550 | 0.650 | 0.600 | 0.775 | 0.775 | 0.825 | 0.925 | 0.825 |
| p013 | 0.125 | 0.175 | 0.125 | 0.100 | 0.225 | 0.300 | 0.225 | 0.400 | 0.450 | 0.475 | 0.625 | 0.325 | 0.275 | 0.350 | 0.300 | 0.375 | 0.475 | 0.325 | 0.575 | 0.550 | 0.625 | 0.625 | 0.250 | 0.325 | 0.300 | 0.225 | 0.225 | 0.275 | 0.325 | 0.325 | 0.275 | 0.350 | 0.400 | 0.525 | 0.500 | 0.375 | 0.425 | 0.525 | 0.475 | 0.475 | 0.575 | 0.525 | 0.450 | 0.625 |
| p015 | 0.050 | 0.125 | 0.125 | 0.200 | 0.250 | 0.400 | 0.500 | 0.725 | 0.675 | 0.825 | 0.850 | 0.125 | 0.200 | 0.200 | 0.300 | 0.325 | 0.475 | 0.500 | 0.675 | 0.850 | 0.800 | 0.875 | 0.450 | 0.475 | 0.325 | 0.425 | 0.350 | 0.400 | 0.475 | 0.450 | 0.450 | 0.475 | 0.425 | 0.725 | 0.475 | 0.500 | 0.550 | 0.425 | 0.575 | 0.575 | 0.425 | 0.625 | 0.525 | 0.500 |
| p016 | 0.075 | 0.150 | 0.075 | 0.175 | 0.200 | 0.350 | 0.450 | 0.575 | 0.675 | 0.775 | 0.950 | 0.175 | 0.200 | 0.250 | 0.275 | 0.375 | 0.450 | 0.550 | 0.625 | 0.775 | 0.750 | 0.925 | 0.175 | 0.275 | 0.150 | 0.300 | 0.275 | 0.375 | 0.400 | 0.600 | 0.550 | 0.525 | 0.675 | 0.475 | 0.600 | 0.675 | 0.525 | 0.700 | 0.725 | 0.800 | 0.800 | 0.825 | 0.800 | 0.775 |
| p017 | 0.275 | 0.250 | 0.225 | 0.500 | 0.450 | 0.400 | 0.550 | 0.725 | 0.650 | 0.850 | 0.850 | 0.300 | 0.400 | 0.300 | 0.375 | 0.475 | 0.550 | 0.650 | 0.700 | 0.725 | 0.875 | 0.900 | 0.100 | 0.225 | 0.275 | 0.200 | 0.350 | 0.375 | 0.275 | 0.300 | 0.300 | 0.325 | 0.475 | 0.350 | 0.400 | 0.350 | 0.450 | 0.350 | 0.400 | 0.450 | 0.425 | 0.375 | 0.425 | 0.425 |
| p018 | 0.150 | 0.125 | 0.150 | 0.275 | 0.325 | 0.475 | 0.475 | 0.750 | 0.750 | 0.875 | 0.900 | 0.250 | 0.300 | 0.300 | 0.300 | 0.400 | 0.475 | 0.650 | 0.775 | 0.875 | 0.950 | 0.925 | 0.400 | 0.475 | 0.550 | 0.400 | 0.625 | 0.500 | 0.475 | 0.475 | 0.500 | 0.550 | 0.675 | 0.775 | 0.800 | 0.825 | 0.825 | 0.800 | 0.800 | 0.800 | 0.900 | 0.875 | 0.925 | 0.825 |
| p019 | 0.250 | 0.325 | 0.300 | 0.375 | 0.350 | 0.400 | 0.500 | 0.725 | 0.725 | 0.775 | 0.875 | 0.300 | 0.400 | 0.300 | 0.300 | 0.425 | 0.650 | 0.625 | 0.600 | 0.875 | 0.900 | 0.900 | 0.250 | 0.225 | 0.225 | 0.250 | 0.175 | 0.350 | 0.300 | 0.375 | 0.325 | 0.375 | 0.325 | 0.650 | 0.675 | 0.625 | 0.650 | 0.750 | 0.725 | 0.800 | 0.725 | 0.775 | 0.825 | 0.925 |
| p020 | 0.175 | 0.150 | 0.200 | 0.225 | 0.250 | 0.325 | 0.400 | 0.500 | 0.500 | 0.550 | 0.600 | 0.550 | 0.550 | 0.475 | 0.625 | 0.600 | 0.650 | 0.875 | 0.900 | 0.800 | 0.850 | 0.925 | 0.150 | 0.200 | 0.250 | 0.250 | 0.350 | 0.550 | 0.575 | 0.750 | 0.825 | 0.825 | 0.925 | 0.250 | 0.275 | 0.200 | 0.250 | 0.325 | 0.475 | 0.700 | 0.750 | 0.825 | 0.825 | 0.875 |
| p021 | 0.100 | 0.075 | 0.150 | 0.150 | 0.250 | 0.375 | 0.500 | 0.700 | 0.800 | 0.925 | 0.850 | 0.125 | 0.050 | 0.125 | 0.275 | 0.250 | 0.450 | 0.650 | 0.750 | 0.850 | 0.900 | 0.900 | 0.025 | 0.100 | 0.050 | 0.100 | 0.100 | 0.225 | 0.275 | 0.275 | 0.225 | 0.325 | 0.325 | 0.400 | 0.400 | 0.375 | 0.450 | 0.425 | 0.500 | 0.525 | 0.575 | 0.600 | 0.600 | 0.550 |
| p022 | 0.225 | 0.275 | 0.300 | 0.425 | 0.475 | 0.475 | 0.650 | 0.725 | 0.775 | 0.875 | 0.875 | 0.300 | 0.325 | 0.400 | 0.525 | 0.525 | 0.525 | 0.525 | 0.750 | 0.775 | 0.850 | 0.925 | 0.550 | 0.650 | 0.725 | 0.625 | 0.675 | 0.625 | 0.650 | 0.550 | 0.750 | 0.625 | 0.700 | 0.775 | 0.725 | 0.725 | 0.700 | 0.850 | 0.800 | 0.725 | 0.750 | 0.675 | 0.775 | 0.725 |
| p023 | 0.150 | 0.200 | 0.200 | 0.200 | 0.300 | 0.450 | 0.600 | 0.850 | 0.800 | 0.825 | 0.800 | 0.250 | 0.200 | 0.225 | 0.350 | 0.425 | 0.400 | 0.725 | 0.825 | 0.875 | 0.875 | 0.925 | 0.325 | 0.275 | 0.425 | 0.275 | 0.425 | 0.575 | 0.425 | 0.600 | 0.625 | 0.800 | 0.750 | 0.500 | 0.475 | 0.475 | 0.425 | 0.500 | 0.700 | 0.750 | 0.875 | 0.900 | 0.750 | 0.850 |
| p024 | 0.025 | 0.100 | 0.200 | 0.200 | 0.275 | 0.450 | 0.500 | 0.725 | 0.825 | 0.900 | 0.925 | 0.150 | 0.250 | 0.250 | 0.300 | 0.400 | 0.550 | 0.625 | 0.775 | 0.800 | 0.950 | 0.900 | 0.050 | 0.200 | 0.150 | 0.100 | 0.250 | 0.275 | 0.250 | 0.300 | 0.300 | 0.325 | 0.300 | 0.225 | 0.375 | 0.400 | 0.350 | 0.475 | 0.550 | 0.500 | 0.575 | 0.675 | 0.575 | 0.750 |
| p025 | 0.500 | 0.350 | 0.400 | 0.400 | 0.500 | 0.550 | 0.550 | 0.650 | 0.750 | 0.750 | 0.875 | 0.600 | 0.600 | 0.625 | 0.600 | 0.750 | 0.675 | 0.675 | 0.825 | 0.875 | 0.900 | 0.975 | 0.500 | 0.500 | 0.450 | 0.600 | 0.550 | 0.650 | 0.600 | 0.675 | 0.775 | 0.750 | 0.675 | 0.675 | 0.675 | 0.625 | 0.700 | 0.725 | 0.825 | 0.900 | 0.850 | 0.825 | 0.825 | 0.900 |
| p026 | 0.100 | 0.125 | 0.150 | 0.125 | 0.225 | 0.400 | 0.450 | 0.700 | 0.725 | 0.900 | 0.925 | 0.325 | 0.325 | 0.325 | 0.350 | 0.450 | 0.600 | 0.650 | 0.900 | 0.925 | 1.000 | 1.000 | 0.275 | 0.325 | 0.225 | 0.250 | 0.450 | 0.350 | 0.450 | 0.450 | 0.525 | 0.550 | 0.675 | 0.575 | 0.625 | 0.700 | 0.800 | 0.950 | 0.825 | 0.950 | 0.900 | 0.925 | 0.900 | 1.000 |
| p027 | 0.300 | 0.275 | 0.125 | 0.250 | 0.375 | 0.400 | 0.525 | 0.375 | 0.550 | 0.675 | 0.675 | 0.300 | 0.400 | 0.300 | 0.325 | 0.475 | 0.300 | 0.550 | 0.625 | 0.625 | 0.650 | 0.650 | 0.400 | 0.425 | 0.475 | 0.375 | 0.450 | 0.600 | 0.475 | 0.625 | 0.550 | 0.550 | 0.600 | 0.650 | 0.600 | 0.525 | 0.575 | 0.750 | 0.625 | 0.625 | 0.625 | 0.525 | 0.650 | 0.600 |
| p028 | 0.100 | 0.100 | 0.175 | 0.275 | 0.400 | 0.575 | 0.775 | 0.975 | 0.975 | 1.000 | 1.000 | 0.200 | 0.200 | 0.200 | 0.275 | 0.425 | 0.750 | 0.875 | 1.000 | 1.000 | 1.000 | 1.000 | 0.275 | 0.350 | 0.325 | 0.300 | 0.450 | 0.500 | 0.600 | 0.700 | 0.750 | 0.750 | 0.800 | 0.475 | 0.525 | 0.700 | 0.750 | 0.825 | 0.800 | 0.950 | 0.925 | 0.950 | 0.950 | 0.975 |
| p029 | 0.100 | 0.125 | 0.175 | 0.325 | 0.200 | 0.500 | 0.550 | 0.650 | 0.900 | 0.925 | 0.950 | 0.225 | 0.175 | 0.200 | 0.200 | 0.275 | 0.525 | 0.625 | 0.900 | 0.900 | 0.925 | 0.925 | 0.325 | 0.375 | 0.400 | 0.500 | 0.400 | 0.425 | 0.525 | 0.450 | 0.550 | 0.550 | 0.500 | 0.600 | 0.850 | 0.750 | 0.700 | 0.750 | 0.825 | 0.900 | 0.825 | 0.775 | 0.825 | 0.875 |
| p030 | 0.100 | 0.025 | 0.125 | 0.075 | 0.175 | 0.350 | 0.475 | 0.625 | 0.775 | 0.850 | 0.925 | 0.150 | 0.150 | 0.150 | 0.250 | 0.300 | 0.550 | 0.600 | 0.725 | 0.900 | 0.900 | 0.975 | 0.200 | 0.250 | 0.300 | 0.275 | 0.200 | 0.400 | 0.425 | 0.425 | 0.550 | 0.525 | 0.600 | 0.500 | 0.475 | 0.600 | 0.525 | 0.650 | 0.575 | 0.650 | 0.650 | 0.775 | 0.750 | 0.775 |

### Spaghetti plots of raw data¶

In [161]:

```
showRaw(exp2Results,['angerLOW','angerUP','fearLOW','fearUP'])
```

```
angerLOW
```

```
angerUP
```

```
fearLOW
```

```
fearUP
```

### fitted functions¶

In [162]:

```
def showAllGrouped(df,condOrder,
            colors=myPalette,
            func=rescaledFunc):
    for index,cond in enumerate(condOrder):
        print cond
        condDict = logDict(df,cond,func=func)
        showCurve(condDict,
                  colors[index],
                  cond,
                  [np.arange(0,10001,1000),np.arange(0,1.01,0.1)],
                  'morphing grade',
                  '% angry'
                 )
```

In [163]:

```
showAll(exp2Results,exp2Results.columns.levels[0])
```

```
angerLOW
```

```
angerUP
```

```
fearLOW
```

```
fearUP
```

### 1st derivatives¶

In [178]:

```
showAllDeriv(exp2Results,['angerLOW','angerUP','fearLOW','fearUP'])
```

In [ ]:

```

```

In [164]:

```
exp2LogFuncTable = peakDf(peakDict(exp2Results))
cols = exp2LogFuncTable.columns.tolist()
cols = [ cols[8],cols[10],cols[9],cols[11] ,  cols[0],cols[2],cols[1],cols[3] , cols[4],cols[6],cols[5],cols[7] ]
exp2LogFuncTable = exp2LogFuncTable[cols]
```

In [165]:

```
exp2LogFuncTable
```

Out[165]:

|  | threshold | | | | bias | | | | slope | | | |
| --- | --- | --- | --- | --- | --- | --- | --- | --- | --- | --- | --- | --- |
|  | angerLOW | fearLOW | angerUP | fearUP | angerLOW | fearLOW | angerUP | fearUP | angerLOW | fearLOW | angerUP | fearUP |
| p001 | 0.549045 | 0.428857 | 0.389161 | 0.481052 | 0.562468 | 0.474925 | 0.562496 | 0.424984 | 0.834755 | 0.783572 | 0.158079 | 0.166005 |
| p002 | 0.357464 | 0.455454 | 0.667333 | 0.393461 | 0.574949 | 0.574960 | 0.812471 | 0.424989 | 0.606712 | 0.759395 | 0.384758 | 0.118639 |
| p003 | 0.463154 | 0.609139 | 0.438256 | 0.568843 | 0.524951 | 0.524877 | 0.637500 | 0.437485 | 2.093929 | 2.154223 | 0.140549 | 0.491733 |
| p004 | 0.482052 | 0.503950 | 0.428657 | 0.335666 | 0.599963 | 0.362441 | 0.712452 | 0.424988 | 0.591612 | 0.747609 | 0.808050 | 0.443633 |
| p005 | 0.509449 | 0.552045 | 0.623838 | 0.606039 | 0.574852 | 0.474983 | 0.949994 | 0.137498 | 1.849724 | 1.415070 | 0.113389 | 0.142007 |
| p006 | 0.619738 | 0.555444 | 0.535846 | 0.999800 | 0.649953 | 0.549982 | 0.674996 | 0.511726 | 0.730381 | 0.612147 | 0.066664 | 0.037438 |
| p007 | 0.561444 | 0.642536 | 0.548045 | 0.554745 | 0.599974 | 0.512459 | 0.887498 | 0.274990 | 2.194922 | 1.418098 | 0.023768 | 0.189414 |
| p008 | 0.402660 | 0.607339 | 0.508349 | 0.538646 | 0.524959 | 0.474952 | 0.749985 | 0.524987 | 0.840455 | 0.752423 | 0.362454 | 0.432326 |
| p009 | 0.575742 | 0.634937 | 0.999800 | 0.155884 | 0.599994 | 0.587492 | 0.666136 | 0.449998 | 0.745337 | 0.642095 | -0.042356 | 0.042482 |
| p010 | 0.490151 | 0.537646 | 0.580042 | 0.725027 | 0.524902 | 0.474962 | 0.724971 | 0.274998 | 1.234805 | 1.222997 | 0.327304 | 0.322235 |
| p011 | 0.547745 | 0.560844 | 0.448755 | 0.505449 | 0.512427 | 0.499850 | 0.587489 | 0.574920 | 1.519367 | 1.516674 | 1.085386 | 0.882807 |
| p012 | 0.566843 | 0.614039 | 0.554045 | 0.568843 | 0.587459 | 0.499915 | 0.699963 | 0.249944 | 1.433742 | 1.343261 | 0.597427 | 0.660127 |
| p013 | 0.573743 | 0.675132 | 0.537446 | 0.676032 | 0.449951 | 0.362426 | 0.499999 | 0.312491 | 0.557379 | 0.757692 | 0.096885 | 0.125825 |
| p015 | 0.536046 | 0.536746 | 0.631137 | 0.000000 | 0.499927 | 0.449928 | 0.536365 | 0.408583 | 1.237714 | 1.399989 | 0.000001 | 0.039892 |
| p016 | 0.601040 | 0.649535 | 0.321768 | 0.556144 | 0.549947 | 0.512497 | 0.649996 | 0.412448 | 1.091507 | 1.405046 | 0.495463 | 0.689743 |
| p017 | 0.566943 | 0.544946 | 0.486751 | 0.476052 | 0.599918 | 0.537425 | 0.399996 | 0.287485 | 0.939315 | 0.873992 | 0.057844 | 0.229794 |
| p018 | 0.568543 | 0.556544 | 0.685431 | 0.708129 | 0.599867 | 0.512442 | 0.849988 | 0.537486 | 1.533890 | 1.379141 | 0.121171 | 0.146229 |
| p019 | 0.564544 | 0.616038 | 0.675432 | 0.420458 | 0.599937 | 0.562482 | 0.774995 | 0.274979 | 1.108939 | 1.088629 | 0.304826 | 0.208005 |
| p020 | 0.481152 | 0.561944 | 0.529647 | 0.535346 | 0.699930 | 0.374960 | 0.537343 | 0.537500 | 0.742235 | 0.761952 | 1.750982 | 1.313661 |
| p021 | 0.502250 | 0.579642 | 0.485251 | 0.462454 | 0.474901 | 0.499894 | 0.487458 | 0.174982 | 1.710924 | 1.695725 | 0.431930 | 0.495434 |
| p022 | 0.565843 | 0.503450 | 0.979102 | 0.546145 | 0.612424 | 0.549973 | 0.747728 | 0.650000 | 0.788684 | 1.011642 | 0.000001 | 0.049051 |
| p023 | 0.526047 | 0.522848 | 0.507349 | 0.601040 | 0.562386 | 0.499973 | 0.662481 | 0.537441 | 1.586671 | 1.957841 | 1.337147 | 0.693325 |
| p024 | 0.516548 | 0.525947 | 0.475952 | 0.316268 | 0.549916 | 0.474890 | 0.487487 | 0.187466 | 1.266291 | 1.468743 | 0.497095 | 0.349926 |
| p025 | 0.671333 | 0.622838 | 0.425857 | 0.498750 | 0.787467 | 0.612468 | 0.762467 | 0.612467 | 0.646251 | 0.693284 | 0.484793 | 0.373887 |
| p026 | 0.565743 | 0.605739 | 0.327067 | 0.625837 | 0.662459 | 0.512340 | 0.787460 | 0.449980 | 1.663806 | 1.682269 | 0.607553 | 0.507261 |
| p027 | 0.551245 | 0.498050 | 0.976902 | 0.479852 | 0.474961 | 0.399941 | 0.613639 | 0.499988 | 0.904465 | 0.597099 | 0.000003 | 0.266525 |
| p028 | 0.457154 | 0.473053 | 0.301370 | 0.528147 | 0.599725 | 0.549835 | 0.724975 | 0.537479 | 3.004358 | 2.187706 | 0.869308 | 1.001837 |
| p029 | 0.533247 | 0.558844 | 0.251475 | 0.386361 | 0.549832 | 0.524855 | 0.749983 | 0.437489 | 2.334222 | 1.541935 | 0.192237 | 0.230770 |
| p030 | 0.559844 | 0.594641 | 0.494851 | 0.581942 | 0.562343 | 0.474984 | 0.624983 | 0.399973 | 1.629243 | 1.715386 | 0.369973 | 0.593552 |

In [166]:

```
exp2LogFuncTable.describe()
```

Out[166]:

|  | threshold | | | | bias | | | | slope | | | |
| --- | --- | --- | --- | --- | --- | --- | --- | --- | --- | --- | --- | --- |
|  | angerLOW | fearLOW | angerUP | fearUP | angerLOW | fearLOW | angerUP | fearUP | angerLOW | fearLOW | angerUP | fearUP |
| count | 29.000000 | 29.000000 | 29.000000 | 29.000000 | 29.000000 | 29.000000 | 29.000000 | 29.000000 | 29.000000 | 29.000000 | 29.000000 | 29.000000 |
| mean | 0.533336 | 0.563040 | 0.545342 | 0.511463 | 0.574922 | 0.497349 | 0.674596 | 0.412750 | 1.290401 | 1.227091 | 0.401472 | 0.387709 |
| std | 0.063019 | 0.060222 | 0.187214 | 0.179162 | 0.069441 | 0.062714 | 0.129249 | 0.135167 | 0.606132 | 0.474071 | 0.428940 | 0.313439 |
| min | 0.357464 | 0.428857 | 0.251475 | 0.000000 | 0.449951 | 0.362426 | 0.399996 | 0.137498 | 0.557379 | 0.597099 | -0.042356 | 0.037438 |
| 25% | 0.502250 | 0.525947 | 0.438256 | 0.462454 | 0.524959 | 0.474952 | 0.587489 | 0.287485 | 0.788684 | 0.759395 | 0.096885 | 0.146229 |
| 50% | 0.549045 | 0.558844 | 0.508349 | 0.535346 | 0.574852 | 0.499973 | 0.674996 | 0.424989 | 1.234805 | 1.343261 | 0.327304 | 0.322235 |
| 75% | 0.566843 | 0.609139 | 0.623838 | 0.581942 | 0.599963 | 0.537425 | 0.749985 | 0.524987 | 1.629243 | 1.516674 | 0.497095 | 0.507261 |
| max | 0.671333 | 0.675132 | 0.999800 | 0.999800 | 0.787467 | 0.612468 | 0.949994 | 0.650000 | 3.004358 | 2.187706 | 1.750982 | 1.313661 |

save to csv

In [167]:

```
exp2LogFuncTable.to_csv('exp2fittable.txt')
```

### violin plots¶

In [168]:

```
for index, metric in enumerate(['threshold','bias','slope']):
    sns.violinplot(exp2LogFuncTable[metric],
                   inner='box',bw=.3, cut=.3,lw=.3,
                   color=myPalette)
    title(metric)
    if index <2:
        ylim(0,1)
        yticks(np.arange(0,1.1,0.1),myTicks)
    sns.despine()
    savefig('violin_exp2_'+metric+'.png',dpi=600)
    show()
```

### line plots¶

In [169]:

```
for index, metric in enumerate(['threshold','bias','slope']):
    ymax=0
    if index <2:
        ymax=1
    plot_curvestats(exp2LogFuncTable[metric],
                    exp2LogFuncTable[metric].columns,
                    metric,'y',lw=5,fasp1=6,fasp2=4,ymin=0,ymax=ymax)
```

### t-tests¶

In [175]:

```
infStats(exp2LogFuncTable['threshold'],0.05)
```

```
angerLOW fearLOW
t: -2.4 p: 0.023170281426 	w: 116.0 wp: 0.0281811829072 *
angerLOW angerUP
t: -0.33 p: 0.741285902731 	w: 215.0 wp: 0.956888952108 n.s.
angerLOW fearUP
t: 0.66 p: 0.515318675767 	w: 198.0 wp: 0.673278736316 n.s.
fearLOW angerLOW
t: 2.4 p: 0.023170281426 	w: 116.0 wp: 0.0281811829072 *
fearLOW angerUP
t: 0.48 p: 0.63813933986 	w: 160.5 wp: 0.217740533516 n.s.
fearLOW fearUP
t: 1.53 p: 0.136687251462 	w: 130.0 wp: 0.0584869701819 n.s.
angerUP angerLOW
t: 0.33 p: 0.741285902731 	w: 215.0 wp: 0.956888952108 n.s.
angerUP fearLOW
t: -0.48 p: 0.63813933986 	w: 160.5 wp: 0.217740533516 n.s.
angerUP fearUP
t: 0.65 p: 0.521206348803 	w: 201.0 wp: 0.721254399159 n.s.
fearUP angerLOW
t: -0.66 p: 0.515318675767 	w: 198.0 wp: 0.673278736316 n.s.
fearUP fearLOW
t: -1.53 p: 0.136687251462 	w: 130.0 wp: 0.0584869701819 n.s.
fearUP angerUP
t: -0.65 p: 0.521206348803 	w: 201.0 wp: 0.721254399159 n.s.
```

In [176]:

```
infStats(exp2LogFuncTable['bias'],0.05)
```

```
angerLOW fearLOW
t: 5.83 p: 2.91153645855e-06 	w: 7.0 wp: 5.32182652461e-06 *
angerLOW angerUP
t: -4.32 p: 0.000176506124218 	w: 52.0 wp: 0.00034538711039 *
angerLOW fearUP
t: 6.83 p: 2.03107544923e-07 	w: 15.0 wp: 1.19390311514e-05 *
fearLOW angerLOW
t: -5.83 p: 2.91153645855e-06 	w: 7.0 wp: 5.32182652461e-06 *
fearLOW angerUP
t: -7.74 p: 1.95071615281e-08 	w: 10.0 wp: 7.22960550776e-06 *
fearLOW fearUP
t: 3.27 p: 0.00285162990724 	w: 94.0 wp: 0.00757461902747 *
angerUP angerLOW
t: 4.32 p: 0.000176506124218 	w: 52.0 wp: 0.00034538711039 *
angerUP fearLOW
t: 7.74 p: 1.95071615281e-08 	w: 10.0 wp: 7.22960550776e-06 *
angerUP fearUP
t: 7.95 p: 1.17965938047e-08 	w: 1.0 wp: 2.84890331208e-06 *
fearUP angerLOW
t: -6.83 p: 2.03107544923e-07 	w: 15.0 wp: 1.19390311514e-05 *
fearUP fearLOW
t: -3.27 p: 0.00285162990724 	w: 94.0 wp: 0.00757461902747 *
fearUP angerUP
t: -7.95 p: 1.17965938047e-08 	w: 1.0 wp: 2.84890331208e-06 *
```

In [177]:

```
infStats(exp2LogFuncTable['slope'],0.05)
```

```
angerLOW fearLOW
t: 1.12 p: 0.272024085878 	w: 201.0 wp: 0.721254399159 n.s.
angerLOW angerUP
t: 6.69 p: 2.89729611796e-07 	w: 21.0 wp: 2.14766190022e-05 *
angerLOW fearUP
t: 8.18 p: 6.69915726825e-09 	w: 7.0 wp: 5.32182652461e-06 *
fearLOW angerLOW
t: -1.12 p: 0.272024085878 	w: 201.0 wp: 0.721254399159 n.s.
fearLOW angerUP
t: 7.9 p: 1.31642823427e-08 	w: 19.0 wp: 1.76907157933e-05 *
fearLOW fearUP
t: 9.94 p: 1.09433438896e-10 	w: 5.0 wp: 4.32894050003e-06 *
angerUP angerLOW
t: -6.69 p: 2.89729611796e-07 	w: 21.0 wp: 2.14766190022e-05 *
angerUP fearLOW
t: -7.9 p: 1.31642823427e-08 	w: 19.0 wp: 1.76907157933e-05 *
angerUP fearUP
t: 0.35 p: 0.731827027433 	w: 200.0 wp: 0.705128996463 n.s.
fearUP angerLOW
t: -8.18 p: 6.69915726825e-09 	w: 7.0 wp: 5.32182652461e-06 *
fearUP fearLOW
t: -9.94 p: 1.09433438896e-10 	w: 5.0 wp: 4.32894050003e-06 *
fearUP angerUP
t: -0.35 p: 0.731827027433 	w: 200.0 wp: 0.705128996463 n.s.
```

### thresholds visualised¶

In [179]:

```
allPeaks(exp2Results,['angerLOW','angerUP','fearLOW','fearUP'])
```

In [180]:

```
allLogWithPeaks(exp2Results,['angerLOW','angerUP','fearLOW','fearUP'])
```

### The same procedure with unscaled data shows that the slopes are located outside the response spectrum for the strongly biased conditions¶

In [185]:

```
allLogWithPeaks(exp2Results,['angerLOW','angerUP','fearLOW','fearUP'],func=originalFunc)
```

### visualisation with upper half / lower half conditions clusterd¶

In [186]:

```
def allLogWithPeaksClusterd(df,colors=myPalette,func=rescaledFunc):
    
    for index,cond in enumerate([u'fearLOW',u'angerLOW',u'fearUP',u'angerUP']):
        condDict = logDict(df,cond,func=func)
        
        for p in condDict:
            highestPos,highestVal = getPeak(condDict,p)
            
            plot(condDict[p],
                 colors[index],
                 alpha=0.5,
                 linewidth=3)

            plot(highestPos,condDict[p][highestPos],
                 'o',
                 color=colors[index],
                 alpha=0.8
                )

        title(cond)
        xticks(np.arange(0,10001,1000),np.arange(0,1.01,0.1))
        xlabel('morphing grade',)
        ylabel('% angry responses')
        sns.despine()
        
        if (index+1)%2==0:
            show()
```

In [187]:

```
allLogWithPeaksClusterd(exp2Results)
```

# Get Logistic data for cross-validation¶

We save all the fitted logistic functions to re-use them in the next notebook, where they will be used to predict out-of sample data. Therefore, we fit the functions separately to part one and part two of the experiment.

In [188]:

```
i = 0
for df in dflist:
    print i,':',df
    i+=1
```

```
0 : Exp1AvgResults.txt
1 : Exp1AvgResultsAll.txt
2 : Exp1Pt1MainResults.txt
3 : Exp1Pt1ResultsAll.txt
4 : Exp1Pt2MainResults.txt
5 : Exp1Pt2ResultsAll.txt
6 : Exp1RtMedian.txt
7 : Exp1RtMedianAll.txt
8 : Exp2AvgResults.txt
9 : Exp2AvgResultsAll.txt
10 : Exp2Pt1Results.txt
11 : Exp2Pt1ResultsAll.txt
12 : Exp2Pt2Results.txt
13 : Exp2Pt2ResultsAll.txt
14 : Exp2RtMedian.txt
15 : Exp2RtMedianAll.txt
16 : exp1Pt1Logistic.txt
17 : exp1Pt2Logistic.txt
18 : exp1fittable.txt
19 : exp1pt1Logistic.txt
20 : exp1pt2Logistic.txt
21 : exp2Pt1Logistic.txt
22 : exp2Pt2Logistic.txt
23 : exp2fittable.txt
24 : exp2pt1Logistic.txt
25 : exp2pt2Logistic.txt
```

In [189]:

```
def getPt(whichPt):
    ptResults = pd.read_csv(dflist[whichPt],
                            header=[0,1],
                            index_col=[0])/100
    return ptResults
```

These are the tables for the separate experiment parts:

In [190]:

```
ex1pt1 = getPt(2)
ex1pt2 = getPt(4)

ex2pt1 = getPt(10)
ex2pt2 = getPt(12)
```

Example:  
The raw data from part 1 are shown in blue
The fitted function is shown in green
By sampling every 1000th point from that function (red), we get a data set that is equal in structure to the raw data.

In [204]:

```
plot(np.arange(0,10001,1000),ex1pt1['whole'].ix['p001'],'o')
plot(rescaledFunc(ex1pt1['whole'].ix['p001']))
plot(np.arange(0,10001,1000),rescaledFunc(ex1pt1['whole'].ix['p001'])[::1000],'o')
```

Out[204]:

```
[<matplotlib.lines.Line2D at 0x7f805433ebd0>]
```

In [199]:

```
def makeFitTable(pt):

    for cond in pt.columns.levels[0]:
        d = {}
        for p in pt.index:
            # the fine-rained nature of the logistic data requires
            # that we extract every 1000th point 
            d[p] = rescaledFunc(pt[cond].ix[p])[::1000]

        thisDf = pd.DataFrame(d).T
        thisDf.columns = ['m00','m01','m02','m03','m04','m05','m06','m07','m08','m09','m10']
        thisDf.index = [[cond]*len(thisDf), thisDf.index]
        try:
            ptLogistic = pd.concat([ptLogistic,thisDf],axis=0)
        except:
            ptLogistic = thisDf

    ptLogistic = ptLogistic.unstack().T.unstack(0)
    return ptLogistic
```

Example:

In [200]:

```
makeFitTable(ex1pt1)
```

Out[200]:

|  | eyes | | | | | | | | | | | mouth | | | | | | | | | | | whole | | | | | | | | | | |
| --- | --- | --- | --- | --- | --- | --- | --- | --- | --- | --- | --- | --- | --- | --- | --- | --- | --- | --- | --- | --- | --- | --- | --- | --- | --- | --- | --- | --- | --- | --- | --- | --- | --- |
|  | m00 | m01 | m02 | m03 | m04 | m05 | m06 | m07 | m08 | m09 | m10 | m00 | m01 | m02 | m03 | m04 | m05 | m06 | m07 | m08 | m09 | m10 | m00 | m01 | m02 | m03 | m04 | m05 | m06 | m07 | m08 | m09 | m10 |
| p001 | 0.035055 | 0.063374 | 0.111919 | 0.190101 | 0.304191 | 0.448808 | 0.602633 | 0.738538 | 0.840281 | 0.907397 | 0.948053 | 0.256650 | 0.292185 | 0.344578 | 0.415257 | 0.499982 | 0.588271 | 0.667814 | 0.730617 | 0.775230 | 0.804598 | 0.822973 | 0.115070 | 0.133703 | 0.173462 | 0.251901 | 0.385195 | 0.563054 | 0.736923 | 0.862196 | 0.933998 | 0.969867 | 0.986557 |
| p002 | 0.175276 | 0.203768 | 0.259525 | 0.355992 | 0.492049 | 0.637449 | 0.753258 | 0.825765 | 0.864550 | 0.883565 | 0.892487 | 0.261547 | 0.275511 | 0.304911 | 0.362109 | 0.458057 | 0.584983 | 0.708932 | 0.798839 | 0.850961 | 0.877337 | 0.889768 | 0.014663 | 0.030182 | 0.061108 | 0.119807 | 0.221585 | 0.373167 | 0.554568 | 0.722510 | 0.844848 | 0.919276 | 0.959703 |
| p003 | 0.343028 | 0.388075 | 0.448351 | 0.523192 | 0.607949 | 0.694488 | 0.773986 | 0.840233 | 0.891098 | 0.927752 | 0.952974 | 0.259357 | 0.298603 | 0.346806 | 0.402764 | 0.463621 | 0.525288 | 0.583455 | 0.634730 | 0.677301 | 0.710921 | 0.736440 | 0.054734 | 0.065197 | 0.097623 | 0.188956 | 0.389539 | 0.661396 | 0.861552 | 0.952570 | 0.984867 | 0.995287 | 0.998543 |
| p004 | 0.283069 | 0.303089 | 0.333588 | 0.377854 | 0.437790 | 0.511739 | 0.593187 | 0.672406 | 0.740667 | 0.793611 | 0.831422 | 0.200407 | 0.222889 | 0.253099 | 0.291676 | 0.337856 | 0.389047 | 0.441175 | 0.489866 | 0.531812 | 0.565502 | 0.591071 | 0.025005 | 0.047623 | 0.088628 | 0.158377 | 0.265084 | 0.404475 | 0.553289 | 0.681549 | 0.773233 | 0.830368 | 0.862985 |
| p005 | 0.035731 | 0.061532 | 0.103728 | 0.169026 | 0.261911 | 0.379350 | 0.507560 | 0.626925 | 0.722669 | 0.790696 | 0.834975 | 0.246170 | 0.268032 | 0.298012 | 0.337012 | 0.384411 | 0.437480 | 0.491705 | 0.542174 | 0.585225 | 0.619303 | 0.644714 | 0.004201 | 0.010132 | 0.024211 | 0.056608 | 0.126113 | 0.255140 | 0.440957 | 0.629912 | 0.764915 | 0.838974 | 0.873821 |
| p006 | 0.060782 | 0.075761 | 0.110218 | 0.184077 | 0.320941 | 0.517470 | 0.716612 | 0.858453 | 0.936105 | 0.972601 | 0.988521 | 0.206153 | 0.241654 | 0.294911 | 0.368829 | 0.461067 | 0.562026 | 0.657841 | 0.737224 | 0.795918 | 0.835763 | 0.861269 | 0.050769 | 0.053142 | 0.062746 | 0.100151 | 0.226637 | 0.509302 | 0.803554 | 0.943182 | 0.985479 | 0.996415 | 0.999123 |
| p007 | 0.106250 | 0.115227 | 0.136540 | 0.184660 | 0.281975 | 0.441272 | 0.629488 | 0.782289 | 0.872857 | 0.916890 | 0.936236 | 0.412489 | 0.444903 | 0.490122 | 0.549156 | 0.619896 | 0.696454 | 0.770694 | 0.835402 | 0.886765 | 0.924590 | 0.950935 | 0.054952 | 0.062598 | 0.081657 | 0.127173 | 0.225617 | 0.399373 | 0.618776 | 0.803182 | 0.912163 | 0.963707 | 0.985513 |
| p008 | 0.165266 | 0.180653 | 0.210426 | 0.265056 | 0.356181 | 0.486437 | 0.636768 | 0.772809 | 0.871364 | 0.931844 | 0.965258 | 0.575570 | 0.592934 | 0.619890 | 0.658619 | 0.708507 | 0.764443 | 0.818197 | 0.862717 | 0.895268 | 0.916962 | 0.930542 | 0.161247 | 0.174055 | 0.200571 | 0.252683 | 0.345369 | 0.484293 | 0.647005 | 0.790545 | 0.888778 | 0.944903 | 0.973714 |
| p009 | 0.009248 | 0.021439 | 0.048863 | 0.107313 | 0.218713 | 0.391966 | 0.591460 | 0.755098 | 0.855930 | 0.907531 | 0.931441 | 0.105569 | 0.115622 | 0.142708 | 0.209257 | 0.341418 | 0.519832 | 0.667057 | 0.746733 | 0.780315 | 0.792956 | 0.797509 | 0.005438 | 0.013233 | 0.031842 | 0.074641 | 0.165154 | 0.326678 | 0.543358 | 0.744784 | 0.877407 | 0.946100 | 0.977298 |
| p010 | 0.137865 | 0.161995 | 0.199494 | 0.254914 | 0.331024 | 0.425650 | 0.529760 | 0.629967 | 0.714744 | 0.778982 | 0.823710 | 0.336336 | 0.357206 | 0.387793 | 0.429936 | 0.483312 | 0.544149 | 0.605687 | 0.660859 | 0.705223 | 0.737877 | 0.760380 | 0.060366 | 0.074852 | 0.108324 | 0.180519 | 0.315474 | 0.511402 | 0.712074 | 0.856042 | 0.935082 | 0.972221 | 0.988392 |
| p011 | 0.002133 | 0.005693 | 0.015104 | 0.039451 | 0.099096 | 0.227556 | 0.441022 | 0.678771 | 0.849831 | 0.938105 | 0.975957 | 0.320902 | 0.341680 | 0.380660 | 0.447905 | 0.548699 | 0.671910 | 0.789900 | 0.879236 | 0.935581 | 0.967129 | 0.983625 | 0.051448 | 0.054605 | 0.064542 | 0.094904 | 0.179856 | 0.368898 | 0.636382 | 0.845442 | 0.945480 | 0.982216 | 0.994355 |
| p012 | 0.273775 | 0.291150 | 0.319910 | 0.365254 | 0.431536 | 0.518437 | 0.617455 | 0.713749 | 0.793997 | 0.852706 | 0.891689 | 0.343323 | 0.378773 | 0.436695 | 0.520749 | 0.623875 | 0.727229 | 0.811805 | 0.870264 | 0.906114 | 0.926510 | 0.937620 | 0.097900 | 0.149665 | 0.245262 | 0.395319 | 0.579751 | 0.748844 | 0.866984 | 0.934742 | 0.969282 | 0.985833 | 0.993529 |
| p013 | 0.030283 | 0.056857 | 0.104111 | 0.182607 | 0.299343 | 0.447216 | 0.600697 | 0.730338 | 0.822108 | 0.879253 | 0.912043 | 0.331388 | 0.355551 | 0.395199 | 0.455084 | 0.535096 | 0.626129 | 0.712559 | 0.781614 | 0.829572 | 0.859726 | 0.877518 | 0.050573 | 0.052129 | 0.057876 | 0.078647 | 0.148164 | 0.331817 | 0.616369 | 0.827074 | 0.913325 | 0.939848 | 0.947251 |
| p014 | 0.068740 | 0.089090 | 0.129647 | 0.205120 | 0.329169 | 0.496676 | 0.671563 | 0.811349 | 0.901106 | 0.950912 | 0.976331 | 0.015329 | 0.032865 | 0.068704 | 0.136665 | 0.248966 | 0.398156 | 0.547861 | 0.661232 | 0.730129 | 0.766549 | 0.784391 | 0.008412 | 0.020243 | 0.047908 | 0.109169 | 0.229854 | 0.420914 | 0.639016 | 0.811718 | 0.913040 | 0.962365 | 0.984196 |
| p015 | 0.091814 | 0.119666 | 0.163466 | 0.228505 | 0.317296 | 0.425550 | 0.540725 | 0.646792 | 0.732250 | 0.793989 | 0.835165 | 0.250747 | 0.273731 | 0.304022 | 0.341367 | 0.383793 | 0.427741 | 0.469116 | 0.504712 | 0.533036 | 0.554206 | 0.569300 | 0.016148 | 0.034946 | 0.073982 | 0.149851 | 0.279998 | 0.461783 | 0.654331 | 0.806813 | 0.902096 | 0.953115 | 0.978190 |
| p016 | 0.063661 | 0.079108 | 0.110824 | 0.172249 | 0.278791 | 0.432520 | 0.604248 | 0.748941 | 0.844597 | 0.898122 | 0.925311 | 0.344825 | 0.376883 | 0.427054 | 0.498619 | 0.588274 | 0.683972 | 0.770148 | 0.836649 | 0.882112 | 0.910676 | 0.927683 | 0.105111 | 0.112509 | 0.130253 | 0.171151 | 0.257334 | 0.408992 | 0.607032 | 0.784907 | 0.898401 | 0.955868 | 0.981580 |
| p019 | 0.054149 | 0.062302 | 0.085871 | 0.149789 | 0.296845 | 0.536585 | 0.770562 | 0.908595 | 0.967349 | 0.988828 | 0.996235 | 0.308045 | 0.317215 | 0.336288 | 0.374196 | 0.443163 | 0.550569 | 0.683115 | 0.806722 | 0.895292 | 0.947481 | 0.974761 | 0.001115 | 0.004235 | 0.015951 | 0.058182 | 0.190569 | 0.472930 | 0.773738 | 0.928738 | 0.980264 | 0.994745 | 0.998616 |
| p020 | 0.264495 | 0.283054 | 0.322660 | 0.398313 | 0.516391 | 0.652968 | 0.765474 | 0.835006 | 0.870646 | 0.887171 | 0.894476 | 0.266139 | 0.281907 | 0.299829 | 0.319523 | 0.340378 | 0.361613 | 0.382390 | 0.401940 | 0.419672 | 0.435226 | 0.448475 | 0.166514 | 0.183582 | 0.216788 | 0.277371 | 0.375973 | 0.510047 | 0.653846 | 0.773664 | 0.854221 | 0.900875 | 0.925592 |
| p021 | 0.005288 | 0.011072 | 0.023011 | 0.047101 | 0.093557 | 0.175799 | 0.301520 | 0.456300 | 0.603158 | 0.711824 | 0.778336 | 0.288341 | 0.311823 | 0.347198 | 0.397275 | 0.462261 | 0.537699 | 0.614729 | 0.683738 | 0.738684 | 0.778466 | 0.805331 | 0.006905 | 0.015547 | 0.034631 | 0.075345 | 0.156182 | 0.295987 | 0.488489 | 0.684467 | 0.831292 | 0.917982 | 0.962155 |
| p022 | 0.066963 | 0.083538 | 0.115180 | 0.172676 | 0.268375 | 0.406553 | 0.570019 | 0.723389 | 0.838992 | 0.912555 | 0.954449 | 0.180873 | 0.206652 | 0.250934 | 0.321278 | 0.420236 | 0.538032 | 0.653309 | 0.746405 | 0.810609 | 0.850215 | 0.872991 | 0.017193 | 0.035197 | 0.070699 | 0.136929 | 0.248604 | 0.408273 | 0.589973 | 0.750038 | 0.862211 | 0.928823 | 0.964556 |
| p023 | 0.169179 | 0.188836 | 0.226779 | 0.295159 | 0.404401 | 0.549283 | 0.700423 | 0.823310 | 0.904531 | 0.951126 | 0.975712 | 0.224887 | 0.244853 | 0.278963 | 0.333720 | 0.413400 | 0.514215 | 0.621410 | 0.716239 | 0.787413 | 0.834517 | 0.863150 | 0.060515 | 0.078145 | 0.123035 | 0.225868 | 0.413476 | 0.646903 | 0.830700 | 0.930039 | 0.973098 | 0.989958 | 0.996294 |
| p024 | 0.157112 | 0.166616 | 0.188208 | 0.234820 | 0.325219 | 0.468991 | 0.638495 | 0.780084 | 0.868105 | 0.913193 | 0.934010 | 0.058230 | 0.067437 | 0.086255 | 0.122612 | 0.185793 | 0.277724 | 0.382007 | 0.471575 | 0.531891 | 0.566154 | 0.583761 | 0.101864 | 0.106307 | 0.121093 | 0.167910 | 0.295523 | 0.536998 | 0.786208 | 0.924466 | 0.976390 | 0.992926 | 0.997908 |
| p025 | 0.160975 | 0.172769 | 0.196444 | 0.241633 | 0.320156 | 0.436668 | 0.574548 | 0.700020 | 0.789687 | 0.843295 | 0.871992 | 0.429767 | 0.440159 | 0.453654 | 0.470791 | 0.491949 | 0.517179 | 0.546051 | 0.577570 | 0.610259 | 0.642407 | 0.672414 | 0.178024 | 0.199999 | 0.237127 | 0.296071 | 0.380996 | 0.487688 | 0.600850 | 0.701236 | 0.777116 | 0.827822 | 0.858973 |
| p026 | 0.224978 | 0.246677 | 0.285015 | 0.348205 | 0.441320 | 0.558123 | 0.678302 | 0.779202 | 0.850432 | 0.894787 | 0.920286 | 0.222762 | 0.238612 | 0.264019 | 0.302431 | 0.355661 | 0.421166 | 0.490945 | 0.554732 | 0.605385 | 0.641299 | 0.664765 | 0.004101 | 0.012212 | 0.035786 | 0.100252 | 0.250659 | 0.501057 | 0.750925 | 0.900508 | 0.964504 | 0.987890 | 0.995933 |
| p027 | 0.281646 | 0.308716 | 0.355537 | 0.429976 | 0.533797 | 0.654960 | 0.770139 | 0.860148 | 0.920311 | 0.956440 | 0.976757 | 0.254494 | 0.300979 | 0.375436 | 0.477085 | 0.589315 | 0.687568 | 0.757581 | 0.800481 | 0.824382 | 0.836997 | 0.843465 | 0.057294 | 0.104196 | 0.182078 | 0.298759 | 0.449152 | 0.609455 | 0.749160 | 0.851100 | 0.916244 | 0.954414 | 0.975651 |
| p028 | 0.006887 | 0.015983 | 0.036601 | 0.081365 | 0.170092 | 0.317991 | 0.505438 | 0.675194 | 0.788086 | 0.848602 | 0.877336 | 0.247536 | 0.280310 | 0.330776 | 0.401724 | 0.489689 | 0.583220 | 0.667747 | 0.733625 | 0.779291 | 0.808433 | 0.826061 | 0.010415 | 0.022783 | 0.049109 | 0.102658 | 0.202182 | 0.359533 | 0.554268 | 0.733657 | 0.859190 | 0.931112 | 0.967680 |
| p029 | 0.006761 | 0.015033 | 0.033053 | 0.070940 | 0.144995 | 0.271079 | 0.442537 | 0.616191 | 0.746753 | 0.824601 | 0.864769 | 0.150318 | 0.151309 | 0.155362 | 0.171416 | 0.227966 | 0.366340 | 0.529477 | 0.614282 | 0.640853 | 0.647753 | 0.649454 | 0.005244 | 0.012527 | 0.029594 | 0.068126 | 0.148283 | 0.289886 | 0.480311 | 0.660421 | 0.782195 | 0.847037 | 0.877228 |
| p030 | 0.395626 | 0.420462 | 0.456228 | 0.504839 | 0.565966 | 0.635736 | 0.707087 | 0.772312 | 0.826100 | 0.866813 | 0.895675 | 0.474459 | 0.498953 | 0.529519 | 0.566391 | 0.609096 | 0.656282 | 0.705787 | 0.754979 | 0.801291 | 0.842727 | 0.878147 | 0.407766 | 0.416716 | 0.435368 | 0.472254 | 0.538193 | 0.637254 | 0.753040 | 0.854529 | 0.923364 | 0.962327 | 0.982156 |

save as csv for re-use in the next notebook

In [201]:

```
def savePandas(where,df,csv):
    os.chdir(where)
    df.to_csv(csv)
```

In [202]:

```
my_folder = './data/'

savePandas(my_folder,makeFitTable(ex1pt1),'exp1Pt1Logistic.txt')
savePandas(my_folder,makeFitTable(ex1pt2),'exp1Pt2Logistic.txt')
savePandas(my_folder,makeFitTable(ex2pt1),'exp2Pt1Logistic.txt')
savePandas(my_folder,makeFitTable(ex2pt2),'exp2Pt2Logistic.txt')
```
