# Supplementary material for: Categorical Perception of Fear and Anger Expressions in Whole, Masked and Composite Faces
Source: PLoS One. 2015 Aug 11;10(8):e0134790. doi: 10.1371/journal.pone.0134790 (PMC4532458; doi:10.1371/journal.pone.0134790)
Supplement: S5 Code — (HTML) [file pone.0134790.s006.html]

SupplementS5\_Functions\_CrossValidation


# Supplement S5 of

## Categorical Perception of Fear and Anger Expressions in Whole, Masked and Composite Faces.

# Cross-Validating¶

- logistic functions are fitted to the raw data of each participant
- fit of both functions as measured by sums of squared errors between raw and fitted data are computed
- the data are cross-validated by splitting them within each participant and using either raw or fitted data to predict the left-out half of the experiment

## Import Libraries¶

In [1]:

```
import fnmatch # filename matching
import os # navigating directories

import numpy as np

from scipy import stats
from scipy.optimize import curve_fit

import pandas as pd
pd.set_option('max_columns', 100)
pd.set_option('max_rows', 1000)

import seaborn as sns

%pylab inline
plt.rcParams['lines.linewidth'] = 5 # default line width for figures
```

```
Populating the interactive namespace from numpy and matplotlib
```

## Get List of previously saved df's (pickle)¶

For generation of the logistic data please refer to S4

In [2]:

```
def get_list(where, what):
    os.chdir(where)
    fileList = []
    for fileName in os.listdir(where):
        if fnmatch.fnmatch(fileName, what):
            fileList.append(fileName)
    return fileList
```

In [3]:

```
fileList = get_list('./data/','*Pt*.txt')
fileList.sort()
for i in range(len(fileList)):
    print i,':\t',fileList[i]
```

```
0 :	Exp1Pt1MainResults.txt
1 :	Exp1Pt1ResultsAll.txt
2 :	Exp1Pt2MainResults.txt
3 :	Exp1Pt2ResultsAll.txt
4 :	Exp2Pt1Results.txt
5 :	Exp2Pt1ResultsAll.txt
6 :	Exp2Pt2Results.txt
7 :	Exp2Pt2ResultsAll.txt
8 :	exp1Pt1Logistic.txt
9 :	exp1Pt2Logistic.txt
10 :	exp2Pt1Logistic.txt
11 :	exp2Pt2Logistic.txt
```

## Load df's¶

In [4]:

```
def load_this(csv):
    df = pd.read_csv(csv,
                     index_col=[0],
                     header=[0,1])
    return df
```

# Experiment 1¶

In [5]:

```
exp1RawPt1 = load_this(fileList[0])/100
exp1RawPt2 = load_this(fileList[2])/100
exp1LogPt1 = load_this(fileList[8])
exp1LogPt2 = load_this(fileList[9])
```

Example:

In [6]:

```
exp1RawPt1
```

Out[6]:

|  | whole | | | | | | | | | | | mouth | | | | | | | | | | | eyes | | | | | | | | | | |
| --- | --- | --- | --- | --- | --- | --- | --- | --- | --- | --- | --- | --- | --- | --- | --- | --- | --- | --- | --- | --- | --- | --- | --- | --- | --- | --- | --- | --- | --- | --- | --- | --- | --- |
| grade | m00 | m01 | m02 | m03 | m04 | m05 | m06 | m07 | m08 | m09 | m10 | m00 | m01 | m02 | m03 | m04 | m05 | m06 | m07 | m08 | m09 | m10 | m00 | m01 | m02 | m03 | m04 | m05 | m06 | m07 | m08 | m09 | m10 |
| p |  |  |  |  |  |  |  |  |  |  |  |  |  |  |  |  |  |  |  |  |  |  |  |  |  |  |  |  |  |  |  |  |  |
| p001 | 0.10 | 0.15 | 0.25 | 0.25 | 0.35 | 0.60 | 0.65 | 0.90 | 1.00 | 1.00 | 1.00 | 0.20 | 0.40 | 0.35 | 0.40 | 0.45 | 0.50 | 0.80 | 0.75 | 0.85 | 0.70 | 0.75 | 0.15 | 0.00 | 0.20 | 0.20 | 0.40 | 0.30 | 0.50 | 0.85 | 0.85 | 1.00 | 0.95 |
| p002 | 0.20 | 0.00 | 0.05 | 0.20 | 0.20 | 0.30 | 0.60 | 0.70 | 0.85 | 0.95 | 1.00 | 0.25 | 0.25 | 0.35 | 0.35 | 0.40 | 0.60 | 0.80 | 0.75 | 0.85 | 0.75 | 0.90 | 0.15 | 0.25 | 0.35 | 0.35 | 0.40 | 0.65 | 0.75 | 0.90 | 0.90 | 0.90 | 0.85 |
| p003 | 0.15 | 0.10 | 0.05 | 0.20 | 0.35 | 0.75 | 0.75 | 1.00 | 1.00 | 1.00 | 1.00 | 0.15 | 0.30 | 0.35 | 0.50 | 0.50 | 0.45 | 0.70 | 0.55 | 0.55 | 0.75 | 0.80 | 0.30 | 0.25 | 0.50 | 0.60 | 0.65 | 0.65 | 0.90 | 0.70 | 0.80 | 1.00 | 0.95 |
| p004 | 0.00 | 0.05 | 0.25 | 0.10 | 0.35 | 0.25 | 0.55 | 0.70 | 0.90 | 0.80 | 0.85 | 0.15 | 0.25 | 0.35 | 0.25 | 0.25 | 0.40 | 0.50 | 0.45 | 0.65 | 0.45 | 0.60 | 0.30 | 0.25 | 0.40 | 0.40 | 0.25 | 0.65 | 0.75 | 0.45 | 0.75 | 0.80 | 0.90 |
| p005 | 0.00 | 0.00 | 0.05 | 0.20 | 0.15 | 0.20 | 0.40 | 0.60 | 0.90 | 0.85 | 0.80 | 0.20 | 0.20 | 0.35 | 0.35 | 0.45 | 0.40 | 0.50 | 0.45 | 0.65 | 0.70 | 0.55 | 0.00 | 0.00 | 0.15 | 0.25 | 0.30 | 0.35 | 0.40 | 0.65 | 0.70 | 0.90 | 0.85 |
| p006 | 0.05 | 0.10 | 0.05 | 0.10 | 0.20 | 0.55 | 0.75 | 1.00 | 0.95 | 1.00 | 1.00 | 0.20 | 0.15 | 0.30 | 0.45 | 0.45 | 0.55 | 0.70 | 0.70 | 0.70 | 0.90 | 0.90 | 0.05 | 0.05 | 0.05 | 0.10 | 0.45 | 0.50 | 0.70 | 0.80 | 1.00 | 0.95 | 1.00 |
| p007 | 0.20 | 0.05 | 0.15 | 0.20 | 0.10 | 0.40 | 0.70 | 0.75 | 0.90 | 1.00 | 0.95 | 0.35 | 0.50 | 0.55 | 0.55 | 0.60 | 0.65 | 0.70 | 0.90 | 1.00 | 0.85 | 0.95 | 0.10 | 0.10 | 0.20 | 0.20 | 0.25 | 0.45 | 0.65 | 0.70 | 0.95 | 0.95 | 0.95 |
| p008 | 0.15 | 0.20 | 0.15 | 0.25 | 0.35 | 0.60 | 0.50 | 0.80 | 0.95 | 0.95 | 1.00 | 0.55 | 0.70 | 0.60 | 0.65 | 0.70 | 0.75 | 0.75 | 0.95 | 0.90 | 0.95 | 0.90 | 0.15 | 0.15 | 0.30 | 0.35 | 0.30 | 0.45 | 0.65 | 0.70 | 1.00 | 0.90 | 1.00 |
| p009 | 0.05 | 0.00 | 0.00 | 0.00 | 0.25 | 0.35 | 0.50 | 0.70 | 0.95 | 0.95 | 1.00 | 0.10 | 0.10 | 0.35 | 0.15 | 0.25 | 0.60 | 0.65 | 0.75 | 0.80 | 0.75 | 0.80 | 0.05 | 0.00 | 0.05 | 0.15 | 0.15 | 0.50 | 0.45 | 0.85 | 0.85 | 0.90 | 0.95 |
| p010 | 0.05 | 0.10 | 0.20 | 0.20 | 0.20 | 0.60 | 0.65 | 0.90 | 0.95 | 0.95 | 1.00 | 0.35 | 0.30 | 0.35 | 0.40 | 0.45 | 0.65 | 0.70 | 0.65 | 0.60 | 0.65 | 0.80 | 0.10 | 0.35 | 0.15 | 0.25 | 0.35 | 0.35 | 0.50 | 0.60 | 0.90 | 0.75 | 0.75 |
| p011 | 0.05 | 0.10 | 0.10 | 0.05 | 0.15 | 0.35 | 0.70 | 0.85 | 0.85 | 0.90 | 1.00 | 0.35 | 0.30 | 0.35 | 0.50 | 0.55 | 0.65 | 0.75 | 0.95 | 1.00 | 0.85 | 0.95 | 0.00 | 0.00 | 0.05 | 0.10 | 0.15 | 0.20 | 0.40 | 0.70 | 0.85 | 1.00 | 0.95 |
| p012 | 0.05 | 0.10 | 0.30 | 0.40 | 0.60 | 0.70 | 0.85 | 1.00 | 0.95 | 1.00 | 0.95 | 0.30 | 0.45 | 0.40 | 0.55 | 0.60 | 0.65 | 0.95 | 0.85 | 0.90 | 0.90 | 0.85 | 0.40 | 0.25 | 0.35 | 0.35 | 0.50 | 0.50 | 0.55 | 0.65 | 0.85 | 0.90 | 0.95 |
| p013 | 0.05 | 0.05 | 0.05 | 0.10 | 0.20 | 0.25 | 0.70 | 0.75 | 0.95 | 0.95 | 0.95 | 0.30 | 0.35 | 0.40 | 0.45 | 0.55 | 0.60 | 0.70 | 0.85 | 0.90 | 0.80 | 0.70 | 0.00 | 0.15 | 0.20 | 0.20 | 0.25 | 0.40 | 0.55 | 0.75 | 0.95 | 0.85 | 0.95 |
| p014 | 0.05 | 0.00 | 0.15 | 0.10 | 0.20 | 0.50 | 0.50 | 0.85 | 1.00 | 1.00 | 1.00 | 0.10 | 0.00 | 0.25 | 0.15 | 0.25 | 0.25 | 0.55 | 0.75 | 0.80 | 0.80 | 0.80 | 0.05 | 0.05 | 0.10 | 0.25 | 0.35 | 0.50 | 0.65 | 0.75 | 0.95 | 1.00 | 1.00 |
| p015 | 0.05 | 0.00 | 0.15 | 0.20 | 0.25 | 0.40 | 0.70 | 0.75 | 1.00 | 0.95 | 1.00 | 0.20 | 0.25 | 0.30 | 0.40 | 0.40 | 0.40 | 0.50 | 0.45 | 0.60 | 0.50 | 0.55 | 0.20 | 0.05 | 0.05 | 0.35 | 0.30 | 0.45 | 0.50 | 0.65 | 0.70 | 0.80 | 0.90 |
| p016 | 0.10 | 0.10 | 0.10 | 0.15 | 0.30 | 0.40 | 0.60 | 0.80 | 0.85 | 1.00 | 1.00 | 0.30 | 0.45 | 0.45 | 0.50 | 0.50 | 0.75 | 0.75 | 0.80 | 0.95 | 0.90 | 0.95 | 0.05 | 0.15 | 0.20 | 0.20 | 0.25 | 0.40 | 0.55 | 0.80 | 0.90 | 0.90 | 0.95 |
| p019 | 0.00 | 0.05 | 0.15 | 0.05 | 0.15 | 0.50 | 0.75 | 0.95 | 1.00 | 1.00 | 1.00 | 0.30 | 0.30 | 0.35 | 0.45 | 0.45 | 0.45 | 0.70 | 0.90 | 0.85 | 0.90 | 1.00 | 0.05 | 0.05 | 0.05 | 0.20 | 0.30 | 0.50 | 0.80 | 0.90 | 1.00 | 0.95 | 1.00 |
| p020 | 0.20 | 0.15 | 0.25 | 0.25 | 0.30 | 0.60 | 0.70 | 0.65 | 0.90 | 0.95 | 0.85 | 0.20 | 0.35 | 0.20 | 0.45 | 0.35 | 0.35 | 0.40 | 0.25 | 0.50 | 0.45 | 0.45 | 0.25 | 0.40 | 0.40 | 0.40 | 0.35 | 0.75 | 0.80 | 0.80 | 0.90 | 0.90 | 0.90 |
| p021 | 0.00 | 0.00 | 0.05 | 0.10 | 0.20 | 0.30 | 0.40 | 0.75 | 0.80 | 0.95 | 1.00 | 0.25 | 0.30 | 0.35 | 0.35 | 0.50 | 0.60 | 0.60 | 0.65 | 0.70 | 0.85 | 0.75 | 0.00 | 0.05 | 0.05 | 0.05 | 0.10 | 0.25 | 0.25 | 0.45 | 0.50 | 0.85 | 0.80 |
| p022 | 0.00 | 0.00 | 0.10 | 0.20 | 0.25 | 0.45 | 0.50 | 0.65 | 1.00 | 1.00 | 1.00 | 0.20 | 0.15 | 0.25 | 0.35 | 0.35 | 0.65 | 0.65 | 0.65 | 0.80 | 0.90 | 0.90 | 0.05 | 0.15 | 0.15 | 0.15 | 0.25 | 0.40 | 0.55 | 0.80 | 0.75 | 1.00 | 0.90 |
| p023 | 0.10 | 0.05 | 0.15 | 0.25 | 0.40 | 0.60 | 0.90 | 0.90 | 1.00 | 1.00 | 1.00 | 0.25 | 0.30 | 0.20 | 0.30 | 0.45 | 0.55 | 0.60 | 0.70 | 0.80 | 0.80 | 0.90 | 0.30 | 0.20 | 0.15 | 0.30 | 0.45 | 0.55 | 0.65 | 0.80 | 0.95 | 1.00 | 1.00 |
| p024 | 0.10 | 0.20 | 0.15 | 0.25 | 0.25 | 0.50 | 0.85 | 0.90 | 1.00 | 0.95 | 1.00 | 0.05 | 0.15 | 0.10 | 0.25 | 0.10 | 0.20 | 0.40 | 0.55 | 0.55 | 0.50 | 0.60 | 0.15 | 0.15 | 0.20 | 0.30 | 0.35 | 0.35 | 0.75 | 0.70 | 0.90 | 0.95 | 0.95 |
| p025 | 0.20 | 0.15 | 0.20 | 0.20 | 0.50 | 0.50 | 0.65 | 0.65 | 0.65 | 0.90 | 0.90 | 0.50 | 0.50 | 0.40 | 0.45 | 0.60 | 0.55 | 0.45 | 0.50 | 0.55 | 0.65 | 0.80 | 0.15 | 0.25 | 0.30 | 0.25 | 0.35 | 0.40 | 0.45 | 0.75 | 0.90 | 0.90 | 0.80 |
| p026 | 0.05 | 0.15 | 0.00 | 0.05 | 0.25 | 0.55 | 0.70 | 0.90 | 1.00 | 1.00 | 1.00 | 0.20 | 0.40 | 0.20 | 0.30 | 0.35 | 0.40 | 0.50 | 0.50 | 0.70 | 0.60 | 0.70 | 0.20 | 0.25 | 0.25 | 0.35 | 0.45 | 0.55 | 0.70 | 0.85 | 0.75 | 0.85 | 0.95 |
| p027 | 0.00 | 0.20 | 0.20 | 0.30 | 0.45 | 0.50 | 0.80 | 0.90 | 0.90 | 1.00 | 1.00 | 0.20 | 0.30 | 0.40 | 0.55 | 0.50 | 0.65 | 0.85 | 0.80 | 0.80 | 0.85 | 0.80 | 0.25 | 0.25 | 0.30 | 0.55 | 0.55 | 0.60 | 0.75 | 0.90 | 0.90 | 0.95 | 1.00 |
| p028 | 0.10 | 0.00 | 0.00 | 0.20 | 0.20 | 0.30 | 0.55 | 0.80 | 0.80 | 0.95 | 1.00 | 0.25 | 0.20 | 0.50 | 0.40 | 0.35 | 0.60 | 0.75 | 0.70 | 0.75 | 0.85 | 0.85 | 0.00 | 0.10 | 0.05 | 0.20 | 0.20 | 0.20 | 0.50 | 0.70 | 0.85 | 0.85 | 0.90 |
| p029 | 0.00 | 0.00 | 0.00 | 0.05 | 0.15 | 0.20 | 0.65 | 0.60 | 0.70 | 0.85 | 0.90 | 0.20 | 0.20 | 0.25 | 0.20 | 0.15 | 0.40 | 0.55 | 0.55 | 0.65 | 0.65 | 0.65 | 0.00 | 0.05 | 0.05 | 0.15 | 0.15 | 0.25 | 0.35 | 0.70 | 0.75 | 0.90 | 0.75 |
| p030 | 0.45 | 0.60 | 0.45 | 0.55 | 0.40 | 0.60 | 0.85 | 0.80 | 1.00 | 0.90 | 1.00 | 0.65 | 0.40 | 0.60 | 0.60 | 0.50 | 0.60 | 0.65 | 0.80 | 0.80 | 1.00 | 0.80 | 0.35 | 0.45 | 0.35 | 0.60 | 0.65 | 0.55 | 0.75 | 0.70 | 0.80 | 0.90 | 0.95 |

In [7]:

```
exp1LogPt1
```

Out[7]:

|  | eyes | | | | | | | | | | | mouth | | | | | | | | | | | whole | | | | | | | | | | |
| --- | --- | --- | --- | --- | --- | --- | --- | --- | --- | --- | --- | --- | --- | --- | --- | --- | --- | --- | --- | --- | --- | --- | --- | --- | --- | --- | --- | --- | --- | --- | --- | --- | --- |
|  | m00 | m01 | m02 | m03 | m04 | m05 | m06 | m07 | m08 | m09 | m10 | m00 | m01 | m02 | m03 | m04 | m05 | m06 | m07 | m08 | m09 | m10 | m00 | m01 | m02 | m03 | m04 | m05 | m06 | m07 | m08 | m09 | m10 |
| p001 | 0.035055 | 0.063374 | 0.111919 | 0.190101 | 0.304191 | 0.448808 | 0.602633 | 0.738538 | 0.840281 | 0.907397 | 0.948053 | 0.256650 | 0.292185 | 0.344578 | 0.415257 | 0.499982 | 0.588271 | 0.667814 | 0.730617 | 0.775230 | 0.804598 | 0.822973 | 0.115070 | 0.133703 | 0.173462 | 0.251901 | 0.385195 | 0.563054 | 0.736923 | 0.862196 | 0.933998 | 0.969867 | 0.986557 |
| p002 | 0.175276 | 0.203768 | 0.259525 | 0.355992 | 0.492049 | 0.637449 | 0.753258 | 0.825765 | 0.864550 | 0.883565 | 0.892487 | 0.261547 | 0.275511 | 0.304911 | 0.362109 | 0.458057 | 0.584983 | 0.708932 | 0.798839 | 0.850961 | 0.877337 | 0.889768 | 0.014663 | 0.030182 | 0.061108 | 0.119807 | 0.221585 | 0.373167 | 0.554568 | 0.722510 | 0.844848 | 0.919276 | 0.959703 |
| p003 | 0.343028 | 0.388075 | 0.448351 | 0.523192 | 0.607949 | 0.694488 | 0.773986 | 0.840233 | 0.891098 | 0.927752 | 0.952974 | 0.259357 | 0.298603 | 0.346806 | 0.402764 | 0.463621 | 0.525288 | 0.583455 | 0.634730 | 0.677301 | 0.710921 | 0.736440 | 0.054734 | 0.065197 | 0.097623 | 0.188956 | 0.389539 | 0.661396 | 0.861552 | 0.952570 | 0.984867 | 0.995287 | 0.998543 |
| p004 | 0.283069 | 0.303089 | 0.333588 | 0.377854 | 0.437790 | 0.511739 | 0.593187 | 0.672406 | 0.740667 | 0.793611 | 0.831422 | 0.200407 | 0.222889 | 0.253099 | 0.291676 | 0.337856 | 0.389047 | 0.441175 | 0.489866 | 0.531812 | 0.565502 | 0.591071 | 0.025005 | 0.047623 | 0.088628 | 0.158377 | 0.265084 | 0.404475 | 0.553289 | 0.681549 | 0.773233 | 0.830368 | 0.862985 |
| p005 | 0.035731 | 0.061532 | 0.103728 | 0.169026 | 0.261911 | 0.379350 | 0.507560 | 0.626925 | 0.722669 | 0.790696 | 0.834975 | 0.246170 | 0.268032 | 0.298012 | 0.337012 | 0.384411 | 0.437480 | 0.491705 | 0.542174 | 0.585225 | 0.619303 | 0.644714 | 0.004201 | 0.010132 | 0.024211 | 0.056608 | 0.126113 | 0.255140 | 0.440957 | 0.629912 | 0.764915 | 0.838974 | 0.873821 |
| p006 | 0.060782 | 0.075761 | 0.110218 | 0.184077 | 0.320941 | 0.517470 | 0.716612 | 0.858453 | 0.936105 | 0.972601 | 0.988521 | 0.206153 | 0.241654 | 0.294911 | 0.368829 | 0.461067 | 0.562026 | 0.657841 | 0.737224 | 0.795918 | 0.835763 | 0.861269 | 0.050769 | 0.053142 | 0.062746 | 0.100151 | 0.226637 | 0.509302 | 0.803554 | 0.943182 | 0.985479 | 0.996415 | 0.999123 |
| p007 | 0.106250 | 0.115227 | 0.136540 | 0.184660 | 0.281975 | 0.441272 | 0.629488 | 0.782289 | 0.872857 | 0.916890 | 0.936236 | 0.412489 | 0.444903 | 0.490122 | 0.549156 | 0.619896 | 0.696454 | 0.770694 | 0.835402 | 0.886765 | 0.924590 | 0.950935 | 0.054952 | 0.062598 | 0.081657 | 0.127173 | 0.225617 | 0.399373 | 0.618776 | 0.803182 | 0.912163 | 0.963707 | 0.985513 |
| p008 | 0.165266 | 0.180653 | 0.210426 | 0.265056 | 0.356181 | 0.486437 | 0.636768 | 0.772809 | 0.871364 | 0.931844 | 0.965258 | 0.575570 | 0.592934 | 0.619890 | 0.658619 | 0.708507 | 0.764443 | 0.818197 | 0.862717 | 0.895268 | 0.916962 | 0.930542 | 0.161247 | 0.174055 | 0.200571 | 0.252683 | 0.345369 | 0.484293 | 0.647005 | 0.790545 | 0.888778 | 0.944903 | 0.973714 |
| p009 | 0.009248 | 0.021439 | 0.048863 | 0.107313 | 0.218713 | 0.391966 | 0.591460 | 0.755098 | 0.855930 | 0.907531 | 0.931441 | 0.105569 | 0.115622 | 0.142708 | 0.209257 | 0.341418 | 0.519832 | 0.667057 | 0.746733 | 0.780315 | 0.792956 | 0.797509 | 0.005438 | 0.013233 | 0.031842 | 0.074641 | 0.165154 | 0.326678 | 0.543358 | 0.744784 | 0.877407 | 0.946100 | 0.977298 |
| p010 | 0.137865 | 0.161995 | 0.199494 | 0.254914 | 0.331024 | 0.425650 | 0.529760 | 0.629967 | 0.714744 | 0.778982 | 0.823710 | 0.336336 | 0.357206 | 0.387793 | 0.429936 | 0.483312 | 0.544149 | 0.605687 | 0.660859 | 0.705223 | 0.737877 | 0.760380 | 0.060366 | 0.074852 | 0.108324 | 0.180519 | 0.315474 | 0.511402 | 0.712074 | 0.856042 | 0.935082 | 0.972221 | 0.988392 |
| p011 | 0.002133 | 0.005693 | 0.015104 | 0.039451 | 0.099096 | 0.227556 | 0.441022 | 0.678771 | 0.849831 | 0.938105 | 0.975957 | 0.320902 | 0.341680 | 0.380660 | 0.447905 | 0.548699 | 0.671910 | 0.789900 | 0.879236 | 0.935581 | 0.967129 | 0.983625 | 0.051448 | 0.054605 | 0.064542 | 0.094904 | 0.179856 | 0.368898 | 0.636382 | 0.845442 | 0.945480 | 0.982216 | 0.994355 |
| p012 | 0.273775 | 0.291150 | 0.319910 | 0.365254 | 0.431536 | 0.518437 | 0.617455 | 0.713749 | 0.793997 | 0.852706 | 0.891689 | 0.343323 | 0.378773 | 0.436695 | 0.520749 | 0.623875 | 0.727229 | 0.811805 | 0.870264 | 0.906114 | 0.926510 | 0.937620 | 0.097900 | 0.149665 | 0.245262 | 0.395319 | 0.579751 | 0.748844 | 0.866984 | 0.934742 | 0.969282 | 0.985833 | 0.993529 |
| p013 | 0.030283 | 0.056857 | 0.104111 | 0.182607 | 0.299343 | 0.447216 | 0.600697 | 0.730338 | 0.822108 | 0.879253 | 0.912043 | 0.331388 | 0.355551 | 0.395199 | 0.455084 | 0.535096 | 0.626129 | 0.712559 | 0.781614 | 0.829572 | 0.859726 | 0.877518 | 0.050573 | 0.052129 | 0.057876 | 0.078647 | 0.148164 | 0.331817 | 0.616369 | 0.827074 | 0.913325 | 0.939848 | 0.947251 |
| p014 | 0.068740 | 0.089090 | 0.129647 | 0.205120 | 0.329169 | 0.496676 | 0.671563 | 0.811349 | 0.901106 | 0.950912 | 0.976331 | 0.015329 | 0.032865 | 0.068704 | 0.136665 | 0.248966 | 0.398156 | 0.547861 | 0.661232 | 0.730129 | 0.766549 | 0.784391 | 0.008412 | 0.020243 | 0.047908 | 0.109169 | 0.229854 | 0.420914 | 0.639016 | 0.811718 | 0.913040 | 0.962365 | 0.984196 |
| p015 | 0.091814 | 0.119666 | 0.163466 | 0.228505 | 0.317296 | 0.425550 | 0.540725 | 0.646792 | 0.732250 | 0.793989 | 0.835165 | 0.250747 | 0.273731 | 0.304022 | 0.341367 | 0.383793 | 0.427741 | 0.469116 | 0.504712 | 0.533036 | 0.554206 | 0.569300 | 0.016148 | 0.034946 | 0.073982 | 0.149851 | 0.279998 | 0.461783 | 0.654331 | 0.806813 | 0.902096 | 0.953115 | 0.978190 |
| p016 | 0.063661 | 0.079108 | 0.110824 | 0.172249 | 0.278791 | 0.432520 | 0.604248 | 0.748941 | 0.844597 | 0.898122 | 0.925311 | 0.344825 | 0.376883 | 0.427054 | 0.498619 | 0.588274 | 0.683972 | 0.770148 | 0.836649 | 0.882112 | 0.910676 | 0.927683 | 0.105111 | 0.112509 | 0.130253 | 0.171151 | 0.257334 | 0.408992 | 0.607032 | 0.784907 | 0.898401 | 0.955868 | 0.981580 |
| p019 | 0.054149 | 0.062302 | 0.085871 | 0.149789 | 0.296845 | 0.536585 | 0.770562 | 0.908595 | 0.967349 | 0.988828 | 0.996235 | 0.308045 | 0.317215 | 0.336288 | 0.374196 | 0.443163 | 0.550569 | 0.683115 | 0.806722 | 0.895292 | 0.947481 | 0.974761 | 0.001115 | 0.004235 | 0.015951 | 0.058182 | 0.190569 | 0.472930 | 0.773738 | 0.928738 | 0.980264 | 0.994745 | 0.998616 |
| p020 | 0.264495 | 0.283054 | 0.322660 | 0.398313 | 0.516391 | 0.652968 | 0.765474 | 0.835006 | 0.870646 | 0.887171 | 0.894476 | 0.266139 | 0.281907 | 0.299829 | 0.319523 | 0.340378 | 0.361613 | 0.382390 | 0.401940 | 0.419672 | 0.435226 | 0.448475 | 0.166514 | 0.183582 | 0.216788 | 0.277371 | 0.375973 | 0.510047 | 0.653846 | 0.773664 | 0.854221 | 0.900875 | 0.925592 |
| p021 | 0.005288 | 0.011072 | 0.023011 | 0.047101 | 0.093557 | 0.175799 | 0.301520 | 0.456300 | 0.603158 | 0.711824 | 0.778336 | 0.288341 | 0.311823 | 0.347198 | 0.397275 | 0.462261 | 0.537699 | 0.614729 | 0.683738 | 0.738684 | 0.778466 | 0.805331 | 0.006905 | 0.015547 | 0.034631 | 0.075345 | 0.156182 | 0.295987 | 0.488489 | 0.684467 | 0.831292 | 0.917982 | 0.962155 |
| p022 | 0.066963 | 0.083538 | 0.115180 | 0.172676 | 0.268375 | 0.406553 | 0.570019 | 0.723389 | 0.838992 | 0.912555 | 0.954449 | 0.180873 | 0.206652 | 0.250934 | 0.321278 | 0.420236 | 0.538032 | 0.653309 | 0.746405 | 0.810609 | 0.850215 | 0.872991 | 0.017193 | 0.035197 | 0.070699 | 0.136929 | 0.248604 | 0.408273 | 0.589973 | 0.750038 | 0.862211 | 0.928823 | 0.964556 |
| p023 | 0.169179 | 0.188836 | 0.226779 | 0.295159 | 0.404401 | 0.549283 | 0.700423 | 0.823310 | 0.904531 | 0.951126 | 0.975712 | 0.224887 | 0.244853 | 0.278963 | 0.333720 | 0.413400 | 0.514215 | 0.621410 | 0.716239 | 0.787413 | 0.834517 | 0.863150 | 0.060515 | 0.078145 | 0.123035 | 0.225868 | 0.413476 | 0.646903 | 0.830700 | 0.930039 | 0.973098 | 0.989958 | 0.996294 |
| p024 | 0.157112 | 0.166616 | 0.188208 | 0.234820 | 0.325219 | 0.468991 | 0.638495 | 0.780084 | 0.868105 | 0.913193 | 0.934010 | 0.058230 | 0.067437 | 0.086255 | 0.122612 | 0.185793 | 0.277724 | 0.382007 | 0.471575 | 0.531891 | 0.566154 | 0.583761 | 0.101864 | 0.106307 | 0.121093 | 0.167910 | 0.295523 | 0.536998 | 0.786208 | 0.924466 | 0.976390 | 0.992926 | 0.997908 |
| p025 | 0.160975 | 0.172769 | 0.196444 | 0.241633 | 0.320156 | 0.436668 | 0.574548 | 0.700020 | 0.789687 | 0.843295 | 0.871992 | 0.429767 | 0.440159 | 0.453654 | 0.470791 | 0.491949 | 0.517179 | 0.546051 | 0.577570 | 0.610259 | 0.642407 | 0.672414 | 0.178024 | 0.199999 | 0.237127 | 0.296071 | 0.380996 | 0.487688 | 0.600850 | 0.701236 | 0.777116 | 0.827822 | 0.858973 |
| p026 | 0.224978 | 0.246677 | 0.285015 | 0.348205 | 0.441320 | 0.558123 | 0.678302 | 0.779202 | 0.850432 | 0.894787 | 0.920286 | 0.222762 | 0.238612 | 0.264019 | 0.302431 | 0.355661 | 0.421166 | 0.490945 | 0.554732 | 0.605385 | 0.641299 | 0.664765 | 0.004101 | 0.012212 | 0.035786 | 0.100252 | 0.250659 | 0.501057 | 0.750925 | 0.900508 | 0.964504 | 0.987890 | 0.995933 |
| p027 | 0.281646 | 0.308716 | 0.355537 | 0.429976 | 0.533797 | 0.654960 | 0.770139 | 0.860148 | 0.920311 | 0.956440 | 0.976757 | 0.254494 | 0.300979 | 0.375436 | 0.477085 | 0.589315 | 0.687568 | 0.757581 | 0.800481 | 0.824382 | 0.836997 | 0.843465 | 0.057294 | 0.104196 | 0.182078 | 0.298759 | 0.449152 | 0.609455 | 0.749160 | 0.851100 | 0.916244 | 0.954414 | 0.975651 |
| p028 | 0.006887 | 0.015983 | 0.036601 | 0.081365 | 0.170092 | 0.317991 | 0.505438 | 0.675194 | 0.788086 | 0.848602 | 0.877336 | 0.247536 | 0.280310 | 0.330776 | 0.401724 | 0.489689 | 0.583220 | 0.667747 | 0.733625 | 0.779291 | 0.808433 | 0.826061 | 0.010415 | 0.022783 | 0.049109 | 0.102658 | 0.202182 | 0.359533 | 0.554268 | 0.733657 | 0.859190 | 0.931112 | 0.967680 |
| p029 | 0.006761 | 0.015033 | 0.033053 | 0.070940 | 0.144995 | 0.271079 | 0.442537 | 0.616191 | 0.746753 | 0.824601 | 0.864769 | 0.150318 | 0.151309 | 0.155362 | 0.171416 | 0.227966 | 0.366340 | 0.529477 | 0.614282 | 0.640853 | 0.647753 | 0.649454 | 0.005244 | 0.012527 | 0.029594 | 0.068126 | 0.148283 | 0.289886 | 0.480311 | 0.660421 | 0.782195 | 0.847037 | 0.877228 |
| p030 | 0.395626 | 0.420462 | 0.456228 | 0.504839 | 0.565966 | 0.635736 | 0.707087 | 0.772312 | 0.826100 | 0.866813 | 0.895675 | 0.474459 | 0.498953 | 0.529519 | 0.566391 | 0.609096 | 0.656282 | 0.705787 | 0.754979 | 0.801291 | 0.842727 | 0.878147 | 0.407766 | 0.416716 | 0.435368 | 0.472254 | 0.538193 | 0.637254 | 0.753040 | 0.854529 | 0.923364 | 0.962327 | 0.982156 |

# Compute Fit of Functions to Raw Data by Sums of Squared Errors¶

## Compute Sum of Squared Errors¶

In [8]:

```
def getSqrdErrors(fit1,fit2,raw1,raw2,cond,thisName):
    firstDf = (fit1[cond]-raw2[cond])**2
    scndDf = (fit2[cond]-raw1[cond])**2
    
    allSums = {}
    for p in firstDf.index:
        allSums[p] = [firstDf.ix[p].sum()]
    
    for p in scndDf.index:
         allSums[p].append(scndDf.ix[p].sum())
    
    meanSums = {}
    for p in allSums:
        meanSums[p] = mean(allSums[p])
    
    meanDf = pd.DataFrame(meanSums,index=[thisName]).T
    meanDf.index = [[cond]*len(meanDf), meanDf.index]
    return meanDf
```

In [9]:

```
def allSqrdConds(fit1,fit2,raw1,raw2,thisName):
    assert raw1.columns.levels[0].all() == raw2.columns.levels[0].all() == fit1.columns.levels[0].all() == fit2.columns.levels[0].all(),"comparing wrong df's!"
    for cond in raw1.columns.levels[0]:
        thisDf = getSqrdErrors(fit1,fit2,raw1,raw2,cond,thisName)
        try:
            bigDf = pd.concat([bigDf,thisDf],axis=0)
        except:
            bigDf = thisDf
    return bigDf
```

In [13]:

```
def allSqrdFits(fit1,fit2,raw1,raw2):
    dfRaw = allSqrdConds(raw1,raw2,raw1,raw2,'raw').unstack(0)
    dfLog = allSqrdConds(fit1,fit2,raw1,raw2,'log').unstack(0)
    dfAll = pd.concat([dfRaw,dfLog],axis=1)
    return dfAll
```

In [14]:

```
exp1CvFits = allSqrdFits(exp1LogPt1,exp1LogPt2,exp1RawPt1,exp1RawPt2)
```

In [15]:

```
exp1CvFits
```

Out[15]:

|  | raw | | | log | | |
| --- | --- | --- | --- | --- | --- | --- |
|  | eyes | mouth | whole | eyes | mouth | whole |
| p001 | 0.1875 | 0.3450 | 0.0525 | 0.149333 | 0.265627 | 0.064312 |
| p002 | 0.1025 | 0.0875 | 0.0625 | 0.042005 | 0.072233 | 0.043708 |
| p003 | 0.2325 | 0.2325 | 0.1425 | 0.137744 | 0.176726 | 0.087216 |
| p004 | 0.2650 | 0.2200 | 0.1150 | 0.125638 | 0.127602 | 0.102354 |
| p005 | 0.1650 | 0.1875 | 0.0975 | 0.088643 | 0.208577 | 0.065074 |
| p006 | 0.1025 | 0.1600 | 0.0625 | 0.096943 | 0.132299 | 0.047785 |
| p007 | 0.0625 | 0.1050 | 0.1300 | 0.047719 | 0.089317 | 0.071827 |
| p008 | 0.0525 | 0.0650 | 0.2000 | 0.055936 | 0.033602 | 0.161427 |
| p009 | 0.0925 | 0.1900 | 0.1175 | 0.060067 | 0.129095 | 0.091930 |
| p010 | 0.4150 | 0.2675 | 0.1975 | 0.332058 | 0.171006 | 0.198118 |
| p011 | 0.1575 | 0.0850 | 0.0900 | 0.153384 | 0.054574 | 0.086016 |
| p012 | 0.1900 | 0.1325 | 0.1025 | 0.129551 | 0.083025 | 0.080577 |
| p013 | 0.0375 | 0.1200 | 0.0525 | 0.052056 | 0.073280 | 0.054525 |
| p014 | 0.0375 | 0.0800 | 0.0850 | 0.026090 | 0.112619 | 0.042682 |
| p015 | 0.1050 | 0.1225 | 0.1050 | 0.091338 | 0.053448 | 0.047318 |
| p016 | 0.0575 | 0.1425 | 0.0500 | 0.070338 | 0.135383 | 0.037818 |
| p019 | 0.0825 | 0.0875 | 0.0550 | 0.053206 | 0.079611 | 0.038567 |
| p020 | 0.2650 | 0.1475 | 0.2450 | 0.121693 | 0.083308 | 0.197509 |
| p021 | 0.0800 | 0.1775 | 0.0400 | 0.084117 | 0.193834 | 0.019602 |
| p022 | 0.1525 | 0.1200 | 0.1600 | 0.083365 | 0.059233 | 0.111726 |
| p023 | 0.2225 | 0.2575 | 0.1550 | 0.176299 | 0.242828 | 0.137028 |
| p024 | 0.1325 | 1.3925 | 0.0425 | 0.097887 | 1.418230 | 0.018808 |
| p025 | 0.0775 | 0.1725 | 0.1200 | 0.078258 | 0.105389 | 0.069021 |
| p026 | 0.1150 | 0.0900 | 0.0675 | 0.072086 | 0.064482 | 0.039078 |
| p027 | 0.0550 | 0.0975 | 0.1025 | 0.045581 | 0.077883 | 0.090140 |
| p028 | 0.0550 | 0.1650 | 0.0850 | 0.054809 | 0.075891 | 0.041974 |
| p029 | 0.2050 | 0.1750 | 0.1600 | 0.150490 | 0.114681 | 0.135082 |
| p030 | 0.0825 | 0.3100 | 0.2950 | 0.052315 | 0.212242 | 0.210392 |

### violin plots and t-tests¶

In [18]:

```
def ssStats(fitDf):
    
    # get differences between logistic and raw fit
    diffDf = fitDf['log']-fitDf['raw']
    
    # plot results
    sns.violinplot(diffDf)
    axhline(0,linewidth=1,color='k')
    sns.despine()
    show()
    
    # inf statistics
    for cond in diffDf.columns:
        t2,p2 = stats.ttest_rel(fitDf['log'][cond],fitDf['raw'][cond])
        t,p = stats.ttest_1samp(diffDf[cond],0)
        assert t2 == t and p2 == p
        w,wp = stats.wilcoxon(fitDf['log'][cond],fitDf['raw'][cond])
        print "--------",cond,"--------"
        print "ttest: t:",t,"\tp:",p
        print "wilco: w:",w, "\tp:",wp
        if p < 0.001 and wp <0.001:
            print "***"
        
    # return df for further use
    return diffDf
```

In [19]:

```
exp1CvFitsDiff = ssStats(exp1CvFits)
```

```
-------- eyes --------
ttest: t: -4.78342872047 	p: 5.45108696799e-05
wilco: w: 30.0 	p: 8.16664930892e-05
***
-------- mouth --------
ttest: t: -5.45511993652 	p: 8.98960590706e-06
wilco: w: 38.0 	p: 0.000171766929212
***
-------- whole --------
ttest: t: -6.91581738083 	p: 1.97164403461e-07
wilco: w: 7.0 	p: 8.0745002878e-06
***
```

In [20]:

```
exp1CvFitsDiff
```

Out[20]:

|  | eyes | mouth | whole |
| --- | --- | --- | --- |
| p001 | -0.038167 | -0.079373 | 0.011812 |
| p002 | -0.060495 | -0.015267 | -0.018792 |
| p003 | -0.094756 | -0.055774 | -0.055284 |
| p004 | -0.139362 | -0.092398 | -0.012646 |
| p005 | -0.076357 | 0.021077 | -0.032426 |
| p006 | -0.005557 | -0.027701 | -0.014715 |
| p007 | -0.014781 | -0.015683 | -0.058173 |
| p008 | 0.003436 | -0.031398 | -0.038573 |
| p009 | -0.032433 | -0.060905 | -0.025570 |
| p010 | -0.082942 | -0.096494 | 0.000618 |
| p011 | -0.004116 | -0.030426 | -0.003984 |
| p012 | -0.060449 | -0.049475 | -0.021923 |
| p013 | 0.014556 | -0.046720 | 0.002025 |
| p014 | -0.011410 | 0.032619 | -0.042318 |
| p015 | -0.013662 | -0.069052 | -0.057682 |
| p016 | 0.012838 | -0.007117 | -0.012182 |
| p019 | -0.029294 | -0.007889 | -0.016433 |
| p020 | -0.143307 | -0.064192 | -0.047491 |
| p021 | 0.004117 | 0.016334 | -0.020398 |
| p022 | -0.069135 | -0.060767 | -0.048274 |
| p023 | -0.046201 | -0.014672 | -0.017972 |
| p024 | -0.034613 | 0.025730 | -0.023692 |
| p025 | 0.000758 | -0.067111 | -0.050979 |
| p026 | -0.042914 | -0.025518 | -0.028422 |
| p027 | -0.009419 | -0.019617 | -0.012360 |
| p028 | -0.000191 | -0.089109 | -0.043026 |
| p029 | -0.054510 | -0.060319 | -0.024918 |
| p030 | -0.030185 | -0.097758 | -0.084608 |

In [21]:

```
exp1CvFitsDiff.describe()
```

Out[21]:

|  | eyes | mouth | whole |
| --- | --- | --- | --- |
| count | 28.000000 | 28.000000 | 28.000000 |
| mean | -0.037805 | -0.038892 | -0.028514 |
| std | 0.041821 | 0.037725 | 0.021817 |
| min | -0.143307 | -0.097758 | -0.084608 |
| 25% | -0.060460 | -0.064921 | -0.044142 |
| 50% | -0.031309 | -0.039059 | -0.024305 |
| 75% | -0.005197 | -0.015118 | -0.014198 |
| max | 0.014556 | 0.032619 | 0.011812 |

## Experiment 2¶

In [22]:

```
exp2RawPt1 = load_this(fileList[4])/100
exp2RawPt2 = load_this(fileList[6])/100
exp2LogPt1 = load_this(fileList[10])
exp2LogPt2 = load_this(fileList[11])
```

In [23]:

```
exp2CvFits = allSqrdFits(exp2LogPt1,exp2LogPt2,exp2RawPt1,exp2RawPt2)
```

In [24]:

```
ssStats(exp2CvFits)
```

```
-------- angerLOW --------
ttest: t: -4.42642526836 	p: 0.000132741178533
wilco: w: 30.0 	p: 5.02738502025e-05
***
-------- angerUP --------
ttest: t: -6.38010736219 	p: 6.62192971514e-07
wilco: w: 3.0 	p: 3.51496308127e-06
***
-------- fearLOW --------
ttest: t: -5.0491342719 	p: 2.42470626711e-05
wilco: w: 30.0 	p: 5.02738502025e-05
***
-------- fearUP --------
ttest: t: -4.96182137504 	p: 3.07851727189e-05
wilco: w: 2.0 	p: 3.16517001863e-06
***
```

Out[24]:

|  | angerLOW | angerUP | fearLOW | fearUP |
| --- | --- | --- | --- | --- |
| p001 | -0.084272 | -0.030249 | -0.070008 | -0.059798 |
| p002 | -0.102911 | -0.105584 | -0.167147 | -0.029719 |
| p003 | -0.022267 | -0.100270 | -0.024158 | -0.012700 |
| p004 | -0.049678 | -0.000708 | -0.051475 | -0.199237 |
| p005 | -0.029045 | -0.026242 | 0.010404 | -0.043107 |
| p006 | -0.028620 | -0.030973 | -0.036631 | -0.011539 |
| p007 | -0.032632 | -0.049288 | -0.026426 | -0.091691 |
| p008 | -0.064121 | -0.078360 | -0.042827 | -0.082052 |
| p009 | -0.084836 | -0.135693 | 0.017191 | -0.065153 |
| p010 | -0.004575 | -0.233808 | -0.053123 | -0.037701 |
| p011 | -0.041573 | 0.008346 | -0.017556 | -0.061048 |
| p012 | -0.044300 | -0.012832 | -0.019036 | -0.040236 |
| p013 | -0.023962 | -0.013540 | -0.064104 | -0.092105 |
| p015 | -0.028012 | -0.019082 | -0.034090 | -0.031758 |
| p016 | -0.109957 | -0.051782 | -0.039384 | -0.243565 |
| p017 | -0.103207 | -0.046854 | -0.005778 | -0.011682 |
| p018 | -0.026883 | -0.081158 | -0.044127 | -0.010942 |
| p019 | -0.050533 | -0.020738 | -0.061877 | -0.024083 |
| p020 | 0.129312 | -0.088079 | -0.044518 | -0.050302 |
| p021 | -0.036158 | -0.050030 | -0.032269 | -0.028426 |
| p022 | -0.089165 | -0.069409 | -0.094327 | -0.064729 |
| p023 | 0.002289 | -0.070078 | -0.075973 | -0.253826 |
| p024 | -0.059416 | -0.072293 | -0.005323 | -0.015331 |
| p025 | -0.011029 | -0.051220 | -0.035923 | -0.006256 |
| p026 | -0.002382 | -0.038162 | -0.019116 | -0.024297 |
| p027 | -0.032805 | -0.132650 | 0.032613 | -0.161600 |
| p028 | -0.008263 | -0.044252 | -0.023819 | 0.010478 |
| p029 | -0.019269 | -0.008083 | 0.025841 | -0.068623 |
| p030 | -0.019670 | -0.057594 | -0.096564 | -0.016455 |

# Cross-Predicting Conditions with Raw Data¶

## Split-Half within Subject (pt1 vs. pt2)¶

In [25]:

```
def logistic(x,a,b):
    y = 1 / (1 + np.exp(-b*(x-a)))
    return y
```

In [26]:

```
def fit_func(func,ydata):
    # for all designs in this study, there are 11 morphing steps, scaled between 0 and 1
    x = arange(0,len(ydata)/10.,0.1)
    # the scipy curvefit function is used here
    popt, pcov = curve_fit(func, x, ydata, maxfev=100000)

    intercept = popt[0] # intercept
    slope = popt[1]     # slope
    y = func(x, intercept,slope) # the y-data of the fitted function

    return x,y,intercept,slope
```

In [27]:

```
def get_cv_ss2(func,ytrain,ytest):
    ''' a certain function (linear or logisitic) is fitted to some training data ytrain. 
    Then the y-values of the fitted function are compared to a left-out dataset ytest
    by a simple sum-of-squares method. Thereby, the fit of the function to new data is
    computed. '''
    
    # instead of fitting a function we might also just compare two raw data sets
    if func == 'raw':
        ss2 = sum(square(ytest-ytrain))
        
    # fitting a function
    else:
        x,y,intercept,slope = fit_func(func,ytrain)
        ss2 = sum( square(ytest-y) )
        
    return ss2
```

## CV with raw data between different conditions¶

Get ss2 for a certain pairing of conditions:

In [28]:

```
def cond_cv_ss2(func,train_df,test_df,train_cond,test_cond):
    assert train_df.index.all() == test_df.index.all(), "comparing incompatible tables!"
    cases = train_df.index
    
    ss2 = []
    for entry in cases:
        ytrain = np.array( train_df.ix[entry][train_cond] )
        ytest = np.array( test_df.ix[entry][test_cond] )
        ss2.append( get_cv_ss2(func,ytrain,ytest) )

    return ss2
```

Get ss2 for all pairings of conditions:

In [29]:

```
def cond_cv_table(test_df,train_df,func):
    d = {}
    for c1 in ['whole','eyes','mouth']:
        for c2 in ['whole','eyes','mouth']:
            d[str(c1+'_'+c2)] = cond_cv_ss2(func,test_df,train_df,c1,c2)
    return pd.DataFrame(d)
```

Average results for both split-half variants:

## Experiment 1¶

In [30]:

```
exp1_cond_cv = (cond_cv_table(exp1RawPt1,exp1RawPt2,'raw') + cond_cv_table(exp1RawPt2,exp1RawPt1,'raw'))/2.
```

In [31]:

```
exp1_cond_cv
```

Out[31]:

|  | eyes\_eyes | eyes\_mouth | eyes\_whole | mouth\_eyes | mouth\_mouth | mouth\_whole | whole\_eyes | whole\_mouth | whole\_whole |
| --- | --- | --- | --- | --- | --- | --- | --- | --- | --- |
| 0 | 0.1875 | 0.58125 | 0.15250 | 0.58125 | 0.3450 | 0.50375 | 0.15250 | 0.50375 | 0.0525 |
| 1 | 0.1025 | 0.08500 | 0.33000 | 0.08500 | 0.0875 | 0.27500 | 0.33000 | 0.27500 | 0.0625 |
| 2 | 0.2325 | 0.44500 | 0.54250 | 0.44500 | 0.2325 | 0.56750 | 0.54250 | 0.56750 | 0.1425 |
| 3 | 0.2650 | 0.41000 | 0.34000 | 0.41000 | 0.2200 | 0.55250 | 0.34000 | 0.55250 | 0.1150 |
| 4 | 0.1650 | 0.42625 | 0.11375 | 0.42625 | 0.1875 | 0.44000 | 0.11375 | 0.44000 | 0.0975 |
| 5 | 0.1025 | 0.35125 | 0.12250 | 0.35125 | 0.1600 | 0.43875 | 0.12250 | 0.43875 | 0.0625 |
| 6 | 0.0625 | 0.72375 | 0.10875 | 0.72375 | 0.1050 | 0.85250 | 0.10875 | 0.85250 | 0.1300 |
| 7 | 0.0525 | 0.97375 | 0.15875 | 0.97375 | 0.0650 | 0.79250 | 0.15875 | 0.79250 | 0.2000 |
| 8 | 0.0925 | 0.18875 | 0.13250 | 0.18875 | 0.1900 | 0.28125 | 0.13250 | 0.28125 | 0.1175 |
| 9 | 0.4150 | 0.39625 | 0.42375 | 0.39625 | 0.2675 | 0.52500 | 0.42375 | 0.52500 | 0.1975 |
| 10 | 0.1575 | 0.87625 | 0.25375 | 0.87625 | 0.0850 | 0.65000 | 0.25375 | 0.65000 | 0.0900 |
| 11 | 0.1900 | 0.26875 | 0.31625 | 0.26875 | 0.1325 | 0.37000 | 0.31625 | 0.37000 | 0.1025 |
| 12 | 0.0375 | 0.64125 | 0.06250 | 0.64125 | 0.1200 | 0.87125 | 0.06250 | 0.87125 | 0.0525 |
| 13 | 0.0375 | 0.29125 | 0.05625 | 0.29125 | 0.0800 | 0.33250 | 0.05625 | 0.33250 | 0.0850 |
| 14 | 0.1050 | 0.41875 | 0.19500 | 0.41875 | 0.1225 | 0.80875 | 0.19500 | 0.80875 | 0.1050 |
| 15 | 0.0575 | 0.81750 | 0.06875 | 0.81750 | 0.1425 | 0.84375 | 0.06875 | 0.84375 | 0.0500 |
| 16 | 0.0825 | 0.33750 | 0.06625 | 0.33750 | 0.0875 | 0.52625 | 0.06625 | 0.52625 | 0.0550 |
| 17 | 0.2650 | 1.10375 | 0.21000 | 1.10375 | 0.1475 | 1.10375 | 0.21000 | 1.10375 | 0.2450 |
| 18 | 0.0800 | 0.58125 | 0.25500 | 0.58125 | 0.1775 | 0.47125 | 0.25500 | 0.47125 | 0.0400 |
| 19 | 0.1525 | 0.20125 | 0.08625 | 0.20125 | 0.1200 | 0.28250 | 0.08625 | 0.28250 | 0.1600 |
| 20 | 0.2225 | 0.38250 | 0.17875 | 0.38250 | 0.2575 | 0.49875 | 0.17875 | 0.49875 | 0.1550 |
| 21 | 0.1325 | 0.72750 | 0.11500 | 0.72750 | 1.3925 | 0.76000 | 0.11500 | 0.76000 | 0.0425 |
| 22 | 0.0775 | 0.49250 | 0.15625 | 0.49250 | 0.1725 | 0.48875 | 0.15625 | 0.48875 | 0.1200 |
| 23 | 0.1150 | 0.32250 | 0.22375 | 0.32250 | 0.0900 | 0.63375 | 0.22375 | 0.63375 | 0.0675 |
| 24 | 0.0550 | 0.11875 | 0.17375 | 0.11875 | 0.0975 | 0.32750 | 0.17375 | 0.32750 | 0.1025 |
| 25 | 0.0550 | 0.52000 | 0.09250 | 0.52000 | 0.1650 | 0.53500 | 0.09250 | 0.53500 | 0.0850 |
| 26 | 0.2050 | 0.23000 | 0.18000 | 0.23000 | 0.1750 | 0.30500 | 0.18000 | 0.30500 | 0.1600 |
| 27 | 0.0825 | 0.25625 | 0.24375 | 0.25625 | 0.3100 | 0.42250 | 0.24375 | 0.42250 | 0.2950 |

In [32]:

```
def make_cv_lineplot(df,cond,start):

    for j in df.index:
        this_results = []
        for c in cond:
            this_results.append(df.ix[j][c])
        
        for n in range(len(cond)-1):
        
            if this_results[n] < this_results[n+1]: 
                plot(
                     [start+n/4.,start+0.25+n/4.],
                     [this_results[n], this_results[n+1]],
                     color='k',
                     alpha=0.2,
                     zorder =0
                    )
            elif this_results[n] >= this_results[n+1]:
                 plot(
                     [start+n/4.,start+0.255+n/4.],
                     [this_results[n], this_results[n+1]],
                     color='b',
                     alpha=0.2,
                     zorder =0
                    )      
                
    i = start
    for c in cond:     
        errorbar(
                 i,
                 df[c].mean(),
                 yerr=df[c].std(ddof=1)/sqrt(len(df.index))*1.96,
                 ecolor='r',
                 capsize=5,
                 capthick=5,
                 zorder=1
                 )
        i+=0.25
```

In [33]:

```
def make_all_cv_plots(pt1,pt2,cond,pltname):
    ax = plt.subplot(111)
    ax.spines['top'].set_visible(False)
    ax.spines['right'].set_visible(False)
    ax.yaxis.set_ticks_position('none')
    ax.xaxis.set_ticks_position('none')

    # do this for raw data or with fitted functions
    for func in ['raw',logistic]:
        df = (cond_cv_table(pt1,pt2,func) + cond_cv_table(pt2,pt1,func))/2.
        print func

        # make list with conditions names (could also be achieved using cond_cv.columns()
        # but the order would not be custom
        all_conds = []

        for c1 in cond:
            for c2 in cond:
                all_conds.append(str(c1+'_'+c2))

        # line plot
        # plot separately for each masking condition (3x3)
        make_cv_lineplot(df,all_conds[0:3],0)
        make_cv_lineplot(df,all_conds[3:6],0.75)
        make_cv_lineplot(df,all_conds[6:9],1.5)
        plt.xticks(arange(0,2.01,0.25),all_conds,rotation=90)
        plt.xlim(-0.1,2.1)

        plt.title("Cross-Validation with "+str(func)+" between conditions")
        plt.xlabel("Face Conditions\n(Raw Data with 95% CI)")
        plt.ylabel("Sums of Squared Errors")
        plt.savefig(pltname+str(func)+'.png',dpi=600)
        plt.show()
```

In [34]:

```
make_all_cv_plots(exp1RawPt1,exp1RawPt2,
                  ['whole','eyes','mouth'],
                  'exp1 cv plot')
```

```
raw
```

```
<function logistic at 0x7fdb6d150500>
```

### Inferential statistics for comparing whether each condition is best at predicting itself¶

In [35]:

```
def cv_stats(df,sig_level):
    for c1 in df.columns:
        for c2 in df.columns:
            if c1 != c2 and c1[:4] == c2[:4]:
                t,p = stats.ttest_rel(df[c1],df[c2])
                W,wp = stats.wilcoxon(df[c1],df[c2])
                if p < sig_level and wp <sig_level:
                    x = '*'
                else:
                    x = ''
                print c1,'\t',c2,x
                print 't=',t,'\tp=',p
                print 'W=',W,'\tp=',wp,'\n'
```

In [36]:

```
cv_stats(exp1_cond_cv,0.05)
```

```
eyes_eyes 	eyes_mouth *
t= -6.51402008274 	p= 5.52585567371e-07
W= 3.0 	p= 5.25641332585e-06 

eyes_eyes 	eyes_whole *
t= -3.29136837308 	p= 0.00278072637318
W= 81.0 	p= 0.00546754901291 

eyes_mouth 	eyes_eyes *
t= 6.51402008274 	p= 5.52585567371e-07
W= 3.0 	p= 5.25641332585e-06 

eyes_mouth 	eyes_whole *
t= 4.96420123919 	p= 3.35312453137e-05
W= 33.0 	p= 0.000108326671921 

eyes_whole 	eyes_eyes *
t= 3.29136837308 	p= 0.00278072637318
W= 81.0 	p= 0.00546754901291 

eyes_whole 	eyes_mouth *
t= -4.96420123919 	p= 3.35312453137e-05
W= 33.0 	p= 0.000108326671921 

mouth_eyes 	mouth_mouth *
t= 4.30152086719 	p= 0.000198496797642
W= 31.0 	p= 8.97749938226e-05 

mouth_eyes 	mouth_whole *
t= -3.23403694977 	p= 0.00321303806999
W= 66.0 	p= 0.00312593174125 

mouth_mouth 	mouth_eyes *
t= -4.30152086719 	p= 0.000198496797642
W= 31.0 	p= 8.97749938226e-05 

mouth_mouth 	mouth_whole *
t= -6.07772163451 	p= 1.72428278039e-06
W= 22.0 	p= 3.76204941685e-05 

mouth_whole 	mouth_eyes *
t= 3.23403694977 	p= 0.00321303806999
W= 66.0 	p= 0.00312593174125 

mouth_whole 	mouth_mouth *
t= 6.07772163451 	p= 1.72428278039e-06
W= 22.0 	p= 3.76204941685e-05 

whole_eyes 	whole_mouth *
t= -7.50020701136 	p= 4.55447606516e-08
W= 3.0 	p= 5.25641332585e-06 

whole_eyes 	whole_whole *
t= 3.56815348855 	p= 0.00137032098393
W= 74.0 	p= 0.00330855640197 

whole_mouth 	whole_eyes *
t= 7.50020701136 	p= 4.55447606516e-08
W= 3.0 	p= 5.25641332585e-06 

whole_mouth 	whole_whole *
t= 10.5982083552 	p= 4.0237030983e-11
W= 0.0 	p= 3.78961944158e-06 

whole_whole 	whole_eyes *
t= -3.56815348855 	p= 0.00137032098393
W= 74.0 	p= 0.00330855640197 

whole_whole 	whole_mouth *
t= -10.5982083552 	p= 4.0237030983e-11
W= 0.0 	p= 3.78961944158e-06
```

Inferential statistics for the difference of differences, comparing whether the whole face and the eyes condition are most similar to each other

In [37]:

```
print "\nwhole-eyes vs whole-mouth"
print "t-test\t",stats.ttest_rel( exp1_cond_cv['whole_whole']-exp1_cond_cv['whole_eyes'] ,  exp1_cond_cv['whole_whole']-exp1_cond_cv['whole_mouth'] )
print "Wilcoxon\t",stats.wilcoxon(  exp1_cond_cv['whole_whole']-exp1_cond_cv['whole_eyes'] ,  exp1_cond_cv['whole_whole']-exp1_cond_cv['whole_mouth'] )
print "t-test\t",stats.ttest_rel( exp1_cond_cv['whole_eyes'] -exp1_cond_cv['whole_whole'] , exp1_cond_cv['whole_eyes'] -exp1_cond_cv['whole_mouth'] )
print "Wilcoxon\t",stats.wilcoxon(  exp1_cond_cv['whole_eyes'] -exp1_cond_cv['whole_whole'] , exp1_cond_cv['whole_eyes'] -exp1_cond_cv['whole_mouth'] )

print "\neyes-whole vs eyes-mouth"
print "t-test\t",stats.ttest_rel( exp1_cond_cv['eyes_eyes']-exp1_cond_cv['eyes_whole'] ,  exp1_cond_cv['eyes_eyes']-exp1_cond_cv['eyes_mouth'] )
print "Wilcoxon\t",stats.wilcoxon(  exp1_cond_cv['eyes_eyes']-exp1_cond_cv['eyes_whole'] ,  exp1_cond_cv['eyes_eyes']-exp1_cond_cv['eyes_mouth'] )
print "t-test\t",stats.ttest_rel( exp1_cond_cv['eyes_whole'] -exp1_cond_cv['eyes_eyes'] , exp1_cond_cv['eyes_whole'] -exp1_cond_cv['eyes_mouth'] )
print "Wilcoxon\t",stats.wilcoxon(  exp1_cond_cv['eyes_whole'] -exp1_cond_cv['eyes_eyes'] , exp1_cond_cv['eyes_whole'] -exp1_cond_cv['eyes_mouth'] )

print "\nmouth-whole vs mouth-eyes"
print "t-test\t",stats.ttest_rel( exp1_cond_cv['mouth_mouth']-exp1_cond_cv['mouth_whole'] ,  exp1_cond_cv['mouth_mouth']-exp1_cond_cv['mouth_eyes'] )
print "Wilcoxon\t",stats.wilcoxon(  exp1_cond_cv['mouth_mouth']-exp1_cond_cv['mouth_whole'] ,  exp1_cond_cv['mouth_mouth']-exp1_cond_cv['mouth_eyes'] )
print "t-test\t",stats.ttest_rel( exp1_cond_cv['mouth_whole'] -exp1_cond_cv['mouth_mouth'] , exp1_cond_cv['mouth_whole'] -exp1_cond_cv['mouth_eyes'] )
print "Wilcoxon\t",stats.wilcoxon(  exp1_cond_cv['mouth_whole'] -exp1_cond_cv['mouth_mouth'] , exp1_cond_cv['mouth_whole'] -exp1_cond_cv['mouth_eyes'] )
```

```
whole-eyes vs whole-mouth
t-test	(array(7.500207011363163), 4.5544760651617197e-08)
Wilcoxon	(3.0, 5.2564133258508337e-06)
t-test	(array(10.598208355184427), 4.0237030983028878e-11)
Wilcoxon	(0.0, 3.7896194415808708e-06)

eyes-whole vs eyes-mouth
t-test	(array(4.9642012391914925), 3.3531245313709309e-05)
Wilcoxon	(33.0, 0.00010832667192099258)
t-test	(array(6.514020082741697), 5.5258556737109939e-07)
Wilcoxon	(3.0, 5.2564133258508337e-06)

mouth-whole vs mouth-eyes
t-test	(array(-3.234036949773224), 0.0032130380699925245)
Wilcoxon	(66.0, 0.0031259317412509913)
t-test	(array(4.301520867194583), 0.00019849679764164768)
Wilcoxon	(31.0, 8.9774993822563996e-05)
```

## Experiment 2¶

In [38]:

```
def cond_cv_table2(test_df,train_df,func):
    d = {}
    for c1 in ['fearLOW','angerLOW','fearUP','angerUP']:
        for c2 in ['fearLOW','angerLOW','fearUP','angerUP']:
            d[str(c1+'_'+c2)] = cond_cv_ss2(func,test_df,train_df,c1,c2)
    return pd.DataFrame(d)
```

In [39]:

```
exp2_cond_cv = (cond_cv_table2(exp2RawPt1,exp2RawPt2,'raw') + cond_cv_table2(exp2RawPt2,exp2RawPt1,'raw'))/2.
exp2_cond_cv
```

Out[39]:

|  | angerLOW\_angerLOW | angerLOW\_angerUP | angerLOW\_fearLOW | angerLOW\_fearUP | angerUP\_angerLOW | angerUP\_angerUP | angerUP\_fearLOW | angerUP\_fearUP | fearLOW\_angerLOW | fearLOW\_angerUP | fearLOW\_fearLOW | fearLOW\_fearUP | fearUP\_angerLOW | fearUP\_angerUP | fearUP\_fearLOW | fearUP\_fearUP |
| --- | --- | --- | --- | --- | --- | --- | --- | --- | --- | --- | --- | --- | --- | --- | --- | --- |
| 0 | 0.2475 | 0.40500 | 0.19750 | 0.52125 | 0.40500 | 0.1475 | 0.40750 | 0.41375 | 0.19750 | 0.40750 | 0.2625 | 0.43375 | 0.52125 | 0.41375 | 0.43375 | 0.1950 |
| 1 | 0.2825 | 0.52000 | 0.33125 | 0.83375 | 0.52000 | 0.2275 | 0.73625 | 1.65125 | 0.33125 | 0.73625 | 0.3550 | 0.80250 | 0.83375 | 1.65125 | 0.80250 | 0.4900 |
| 2 | 0.0925 | 1.12000 | 0.39875 | 1.06000 | 1.12000 | 0.3275 | 1.65875 | 1.00250 | 0.39875 | 1.65875 | 0.2800 | 0.85625 | 1.06000 | 1.00250 | 0.85625 | 0.7075 |
| 3 | 0.2200 | 0.36000 | 1.08125 | 0.42125 | 0.36000 | 0.0850 | 1.74875 | 0.91625 | 1.08125 | 1.74875 | 0.5525 | 0.37500 | 0.42125 | 0.91625 | 0.37500 | 0.3575 |
| 4 | 0.3150 | 2.56875 | 0.43625 | 3.12125 | 2.56875 | 0.0425 | 3.66250 | 7.53750 | 0.43625 | 3.66250 | 0.1875 | 1.99500 | 3.12125 | 7.53750 | 1.99500 | 0.1425 |
| 5 | 0.1300 | 0.53750 | 0.19125 | 0.64875 | 0.53750 | 0.1800 | 0.50875 | 0.59125 | 0.19125 | 0.50875 | 0.0925 | 0.40500 | 0.64875 | 0.59125 | 0.40500 | 0.2525 |
| 6 | 0.1675 | 1.79875 | 0.34625 | 1.39375 | 1.79875 | 0.1350 | 2.89500 | 4.29000 | 0.34625 | 2.89500 | 0.0750 | 0.81000 | 1.39375 | 4.29000 | 0.81000 | 0.1900 |
| 7 | 0.1800 | 0.59750 | 0.38500 | 0.41250 | 0.59750 | 0.1850 | 1.22500 | 0.80250 | 0.38500 | 1.22500 | 0.2000 | 0.43750 | 0.41250 | 0.80250 | 0.43750 | 0.4850 |
| 8 | 0.1975 | 0.67125 | 0.20625 | 0.46125 | 0.67125 | 0.2800 | 0.64500 | 0.74500 | 0.20625 | 0.64500 | 0.0600 | 0.39000 | 0.46125 | 0.74500 | 0.39000 | 0.1150 |
| 9 | 0.0925 | 0.90000 | 0.23000 | 1.63125 | 0.90000 | 0.3625 | 1.27750 | 2.66125 | 0.23000 | 1.27750 | 0.1475 | 1.15625 | 1.63125 | 2.66125 | 1.15625 | 0.0950 |
| 10 | 0.1200 | 0.54250 | 0.11500 | 0.30875 | 0.54250 | 0.0650 | 0.58750 | 0.16875 | 0.11500 | 0.58750 | 0.0800 | 0.40625 | 0.30875 | 0.16875 | 0.40625 | 0.2375 |
| 11 | 0.1500 | 0.53875 | 0.32000 | 1.46500 | 0.53875 | 0.2875 | 1.19125 | 2.32375 | 0.32000 | 1.19125 | 0.0750 | 0.73750 | 1.46500 | 2.32375 | 0.73750 | 0.1400 |
| 12 | 0.2500 | 0.34375 | 0.40625 | 0.52125 | 0.34375 | 0.2825 | 0.74500 | 0.66750 | 0.40625 | 0.74500 | 0.1375 | 0.28000 | 0.52125 | 0.66750 | 0.28000 | 0.2175 |
| 13 | 0.2825 | 0.99625 | 0.34750 | 0.77625 | 0.99625 | 0.2600 | 1.31625 | 0.38750 | 0.34750 | 1.31625 | 0.4725 | 0.88375 | 0.77625 | 0.38750 | 0.88375 | 0.1850 |
| 14 | 0.2150 | 0.90000 | 0.22250 | 0.32750 | 0.90000 | 0.1850 | 1.53500 | 1.31250 | 0.22250 | 1.53500 | 0.1050 | 0.34500 | 0.32750 | 1.31250 | 0.34500 | 0.5350 |
| 15 | 0.2300 | 0.79500 | 0.14875 | 1.16750 | 0.79500 | 0.0800 | 0.63375 | 0.30750 | 0.14875 | 0.63375 | 0.0875 | 1.01875 | 1.16750 | 0.30750 | 1.01875 | 0.2050 |
| 16 | 0.0600 | 1.43250 | 0.18250 | 0.72625 | 1.43250 | 0.1750 | 2.17000 | 1.31125 | 0.18250 | 2.17000 | 0.1050 | 0.81375 | 0.72625 | 1.31125 | 0.81375 | 0.1725 |
| 17 | 0.1675 | 0.97750 | 0.19875 | 1.50500 | 0.97750 | 1.4075 | 1.26875 | 2.38250 | 0.19875 | 1.26875 | 0.1450 | 1.01375 | 1.50500 | 2.38250 | 1.01375 | 0.7775 |
| 18 | 2.1500 | 1.16000 | 2.48125 | 1.43250 | 1.16000 | 0.2150 | 0.96625 | 0.18000 | 2.48125 | 0.96625 | 2.3175 | 1.17875 | 1.43250 | 0.18000 | 1.17875 | 0.1800 |
| 19 | 0.1175 | 0.72875 | 0.10750 | 1.58500 | 0.72875 | 0.1400 | 0.69375 | 1.11125 | 0.10750 | 0.69375 | 0.0975 | 1.30000 | 1.58500 | 1.11125 | 1.30000 | 0.0575 |
| 20 | 0.2275 | 0.87250 | 0.17750 | 0.60250 | 0.87250 | 0.2475 | 1.20000 | 0.31000 | 0.17750 | 1.20000 | 0.2025 | 0.80250 | 0.60250 | 0.31000 | 0.80250 | 0.1925 |
| 21 | 0.7775 | 0.72125 | 0.58500 | 0.49125 | 0.72125 | 0.2700 | 0.69875 | 0.53500 | 0.58500 | 0.69875 | 0.3325 | 0.39375 | 0.49125 | 0.53500 | 0.39375 | 0.8300 |
| 22 | 0.1950 | 0.39750 | 0.24875 | 1.61500 | 0.39750 | 0.2250 | 0.54875 | 0.90000 | 0.24875 | 0.54875 | 0.2125 | 1.37125 | 1.61500 | 0.90000 | 1.37125 | 0.0550 |
| 23 | 0.0450 | 0.15375 | 0.39875 | 0.26875 | 0.15375 | 0.1475 | 0.62000 | 0.40000 | 0.39875 | 0.62000 | 0.1525 | 0.19500 | 0.26875 | 0.40000 | 0.19500 | 0.0675 |
| 24 | 0.1300 | 0.97000 | 0.48375 | 1.01125 | 0.97000 | 0.1100 | 2.34375 | 2.15375 | 0.48375 | 2.34375 | 0.1525 | 0.64500 | 1.01125 | 2.15375 | 0.64500 | 0.3925 |
| 25 | 0.2900 | 0.78750 | 0.33375 | 0.31375 | 0.78750 | 0.6150 | 0.96875 | 0.40375 | 0.33375 | 0.96875 | 0.2675 | 0.45000 | 0.31375 | 0.40375 | 0.45000 | 0.2825 |
| 26 | 0.0775 | 0.86500 | 0.11625 | 0.54625 | 0.86500 | 0.1225 | 1.14125 | 1.04375 | 0.11625 | 1.14125 | 0.0850 | 0.48000 | 0.54625 | 1.04375 | 0.48000 | 0.3850 |
| 27 | 0.0975 | 1.65250 | 0.16375 | 0.95125 | 1.65250 | 0.1125 | 1.91625 | 1.37875 | 0.16375 | 1.91625 | 0.1500 | 0.87500 | 0.95125 | 1.37875 | 0.87500 | 0.1900 |
| 28 | 0.0550 | 0.75875 | 0.20500 | 0.65250 | 0.75875 | 0.1125 | 1.19375 | 0.84125 | 0.20500 | 1.19375 | 0.2200 | 0.49250 | 0.65250 | 0.84125 | 0.49250 | 0.1400 |

In [40]:

```
# remove extreme outlier for visualisation 
exp2_cond_cv_rev = pd.concat([exp2_cond_cv[:4], exp2_cond_cv[5:6], exp2_cond_cv[7:]])
```

In [41]:

```
ax = plt.subplot(111)
ax.spines['top'].set_visible(False)
ax.spines['right'].set_visible(False)
ax.yaxis.set_ticks_position('none')
ax.xaxis.set_ticks_position('none')

# make custom condition order
cond = ['fearLOW','angerLOW','fearUP','angerUP']
all_conds = []
for c in cond:
    all_conds = all_conds + ['fearLOW_'+c,'angerLOW_'+c,'fearUP_'+c,'angerUP_'+c]
print all_conds
make_cv_lineplot(exp2_cond_cv_rev,all_conds[0:4],0)
make_cv_lineplot(exp2_cond_cv_rev,all_conds[4:8],1)
make_cv_lineplot(exp2_cond_cv_rev,all_conds[8:12],2)
make_cv_lineplot(exp2_cond_cv_rev,all_conds[12:16],3)

# customise plot
plt.xlim(-0.2,3.9)
#plt.ylim(0,2.5)
plt.xticks(arange(0,3.76,0.25),all_conds,rotation=90)
plt.title("Cross-Validation with Raw Data between Conditions")
plt.xlabel("Face Conditions\n(Raw Data with 95% CI)")
plt.ylabel("Sums of Squared Errors")
plt.savefig('Experiment2_CV_rawcond_line.png',dpi=600)
plt.show()
```

```
['fearLOW_fearLOW', 'angerLOW_fearLOW', 'fearUP_fearLOW', 'angerUP_fearLOW', 'fearLOW_angerLOW', 'angerLOW_angerLOW', 'fearUP_angerLOW', 'angerUP_angerLOW', 'fearLOW_fearUP', 'angerLOW_fearUP', 'fearUP_fearUP', 'angerUP_fearUP', 'fearLOW_angerUP', 'angerLOW_angerUP', 'fearUP_angerUP', 'angerUP_angerUP']
```

### Inferential Statistics Checking Whether Each Condition is Best At Predicting Itself¶

In [42]:

```
for i in [0,4,8,12]:
    print '\n'
    for c1 in exp2_cond_cv.columns[i:i+4]:
        for c2 in exp2_cond_cv.columns[i:i+4]:
            if c1.split('_')[0] == c1.split('_')[1]:
                if (exp2_cond_cv[c1] == exp2_cond_cv[c2] ).all() == False:
                   
                    t,p = stats.ttest_rel(exp2_cond_cv[c1],exp2_cond_cv[c2])
                    W,wp = stats.wilcoxon(exp2_cond_cv[c1],exp2_cond_cv[c2])
                    
                    if p < 0.01 and wp <0.01:
                        x = '*'
                    else:
                        x = ''
    
                    print c1,'\t',c2,x
                    print round(t,2),'\t',p,'\t',round(W,2),'\t',wp
```

```
angerLOW_angerLOW 	angerLOW_angerUP *
-5.43 	8.50783725631e-06 	25.0 	3.14828720478e-05
angerLOW_angerLOW 	angerLOW_fearLOW *
-3.35 	0.00234444758825 	62.0 	0.000772659290871
angerLOW_angerLOW 	angerLOW_fearUP *
-5.23 	1.47132219571e-05 	28.0 	4.17461934344e-05


angerUP_angerUP 	angerUP_angerLOW *
-5.87 	2.60551112516e-06 	12.0 	8.84792402511e-06
angerUP_angerUP 	angerUP_fearLOW *
-6.61 	3.63404503644e-07 	1.0 	2.847002324e-06
angerUP_angerUP 	angerUP_fearUP *
-3.87 	0.000592226591254 	6.0 	4.79779518445e-06


fearLOW_fearLOW 	fearLOW_angerLOW *
-4.58 	8.710747889e-05 	41.0 	0.000135360813605
fearLOW_fearLOW 	fearLOW_angerUP *
-6.03 	1.68021816462e-06 	22.0 	2.3647459451e-05
fearLOW_fearLOW 	fearLOW_fearUP *
-4.9 	3.6184762645e-05 	31.0 	5.5133311257e-05


fearUP_fearUP 	fearUP_angerLOW *
-4.96 	3.09741692117e-05 	21.0 	2.14766190022e-05
fearUP_fearUP 	fearUP_angerUP *
-3.7 	0.000938585262434 	8.0 	8.97803219694e-06
fearUP_fearUP 	fearUP_fearLOW *
-4.88 	3.81934278308e-05 	29.0 	4.58222531384e-05
```
