# Supplementary material for: Categorical Perception of Fear and Anger Expressions in Whole, Masked and Composite Faces
Source: PLoS One. 2015 Aug 11;10(8):e0134790. doi: 10.1371/journal.pone.0134790 (PMC4532458; doi:10.1371/journal.pone.0134790)
Supplement: S6 Code — (HTML) [file pone.0134790.s007.html]

SupplementS6\_AdditionalIdentityAnalyses


# Supplement S6 of

## Categorical Perception of Fear and Anger Expressions in Whole, Masked and Composite Faces.

# Aggregate Data by Face Identitiy¶

This analysis describes the response function for each face identity individually. This helps to check whether there are outlier faces that have a shifted or odd response curve.  
In a second step, the responses for each face idenitity are described by a sigmoid (logistic) function and the parameters for slope and threshold are extracted. To get an estimate of how the average curve across all face identities would be shaped if the threshold for all these curves were identical, we take the threshold parameters and shift the (raw) data so that all curves have the same (mean) x-threshold.  
These shifted data should be more sensitive to detecting a sigmoid shape. This may also allow to check if the more linear responses for the lower face half condition can be explained by more variability in the threshold of the curves (while still being sigmoid individually), or if the slopes for the condition are really as flat as suggested by the pooled analysis.

## Import Libraries¶

In [1]:

```
import fnmatch
import os

import numpy as np
from scipy.optimize import curve_fit

import pandas as pd
pd.set_option('max_columns', 200)
pd.set_option('max_rows', 1000)

import seaborn as sns
sns.set_style("ticks")

%pylab inline
plt.rcParams['lines.linewidth'] = 5
```

```
Populating the interactive namespace from numpy and matplotlib
```

## Get List of Files¶

In [2]:

```
def getsubject(whichFolder, whichExperiment):
    os.chdir(whichFolder)
    pList = []
    for fileName in os.listdir(whichFolder):
        if fnmatch.fnmatch(fileName, whichExperiment):
            pList.append(fileName)
    return pList
```

In [3]:

```
pList = getsubject("/home/martin/FACES_PARAMETRIC/SUPPLEMENT/RAWDATA/Exp1/", "*Vp*.log")
pList.sort()
```

## Make Empty Dictionary¶

The dictionary is structured by masking condition and then by face identity. Therefore, responses from all participants are aggregated for each stimulus, violating some assumptions such as independence (as each participant contributes two values). However, we only use the data to estimate shape and position of each curve.

In [5]:

```
def make_structure(conds):
    
    # empty dict to fill
    d = {}
    
    # names of conditions
    idents= ['F1_','F2_','F3_','F4_','F5_','F6_','F7_','F8_','F9_','F10_','M1_','M2_','M3_','M4_','M5_','M6_','M7_','M8_','M9_','M10_']
    grades = ['_00_','_01_','_02_','_03_','_04_','_05_','_06_','_07_','_08_','_09_','_10_']
    
    for cond in conds:
        # make a dict for each masking type
        d[cond] = {}
        for ident in idents:
            # make a dict for each face identitiy
            d[cond][ident] = {}
            for grade in grades:
                # make a dict for each morphing grade and set starting value at zero
                # later, this value will increase each time a participant makes an angry response
                d[cond][ident][grade] = 0
    
    return d
```

define condition names for later use:  
  
'orig' = whole face;  
'noe' ('no eyes') = mouth is visible;  
'nom' ('no mouth') = eyes are visible;

In [6]:

```
conds = ['orig','noe','nom']
```

## Get Data From Files and Add to Dictionary¶

In [7]:

```
def findentry_sub(d,filename,conds,cond,ident,grade):
    # open the file
    filecontent = open(filename,'r')
    
    # conditions to choose from
    idents= ['F1_','F2_','F3_','F4_','F5_','F6_','F7_','F8_','F9_','F10_','M1_','M2_','M3_','M4_','M5_','M6_','M7_','M8_','M9_','M10_']
    grades = ['_00_','_01_','_02_','_03_','_04_','_05_','_06_','_07_','_08_','_09_','_10_']
    
    # go through all lines of textfile
    for entry in filecontent:
        # split for experiment 2
        entry = entry.split(';')
        # if the line contains all conditions in question (masking type, face identity & morphing grade),
        if entry[-1].find(conds[cond]) != -1 and entry[0].find(idents[ident]) != -1 and entry[0].find(grades[grade]) != -1:
            # and if the response was a hit (i.e. participant chose angry)
            if entry[-1].find("hit") != -1:
                # increase response count of respective condition in the dict
                d[conds[cond]][idents[ident]][grades[grade]] += 1
    return d
```

## Work Through all Files¶

Go through all grades for a certain masking type, face identity and participant file

In [9]:

```
def all_grades(d,filename,conds,cond,ident):
    for grade in range(11):
        findentry_sub(d,filename,conds,cond,ident,grade)
    return d
```

Go through all face identities for a certain masking type and partipant file

In [10]:

```
def all_idents(d,filename,conds,cond):
    for ident in range(20):
        all_grades(d,filename,conds,cond,ident)
    return d
```

Go through all masking types for a certain participant file

In [11]:

```
def all_conds(d,filename,conds):
    for cond in range(len(conds)):
        all_idents(d,filename,conds,cond)
    return d
```

Go through all participant files (due to my poor coding skills, this will take really long - sorry)

In [12]:

```
def all_files(file_list,conds):
    d = make_structure(conds)
    for f in range(len(file_list)):
        #print "file %s is being processed..." % str(f+1)
        all_conds(d,file_list[f],conds)
    return d
```

## Process all Data¶

For all participants (N=30)

In [13]:

```
my_results = all_files(pList,conds)
```

Excluding non-compliant participants (N=28)

In [14]:

```
RevList = pList[:32] + pList[36:]
my_rev_results = all_files(RevList,conds)
```

# Analysis at Level of Face Identitites¶

## Make Pandas Data Frame¶

Here a legacy naming of the conditions is used:

'orig': whole face  
'nom' (no mouth)': upper half  
'noe' (no eyes)': lower half

In [16]:

```
orig_df = pd.DataFrame(my_rev_results['orig'])
orig_df.index = [['orig']*11,list(orig_df.index)]

noe_df = pd.DataFrame(my_rev_results['noe'])
noe_df.index = [['noe']*11,list(noe_df.index)]

nom_df = pd.DataFrame(my_rev_results['nom'])
nom_df.index = [['nom']*11,list(nom_df.index)]

n_of_participants = 28 # for sample with N=28
ident_df = pd.concat([orig_df,noe_df,nom_df],axis = 0)
ident_df = ident_df/(n_of_participants*2.0) # there are N subjects, each providing 2 responses, so we need to divide by twice the participant number
```

In [17]:

```
ident_df
```

Out[17]:

|  |  | F10\_ | F1\_ | F2\_ | F3\_ | F4\_ | F5\_ | F6\_ | F7\_ | F8\_ | F9\_ | M10\_ | M1\_ | M2\_ | M3\_ | M4\_ | M5\_ | M6\_ | M7\_ | M8\_ | M9\_ |
| --- | --- | --- | --- | --- | --- | --- | --- | --- | --- | --- | --- | --- | --- | --- | --- | --- | --- | --- | --- | --- | --- |
| orig | \_00\_ | 0.178571 | 0.053571 | 0.250000 | 0.000000 | 0.017857 | 0.017857 | 0.071429 | 0.053571 | 0.035714 | 0.071429 | 0.089286 | 0.017857 | 0.053571 | 0.089286 | 0.107143 | 0.214286 | 0.053571 | 0.178571 | 0.392857 | 0.017857 |
|  | \_01\_ | 0.303571 | 0.035714 | 0.178571 | 0.017857 | 0.017857 | 0.017857 | 0.035714 | 0.089286 | 0.017857 | 0.089286 | 0.017857 | 0.071429 | 0.017857 | 0.178571 | 0.017857 | 0.214286 | 0.053571 | 0.267857 | 0.375000 | 0.035714 |
|  | \_02\_ | 0.321429 | 0.089286 | 0.321429 | 0.017857 | 0.000000 | 0.035714 | 0.053571 | 0.196429 | 0.017857 | 0.089286 | 0.125000 | 0.053571 | 0.017857 | 0.214286 | 0.142857 | 0.214286 | 0.089286 | 0.321429 | 0.517857 | 0.017857 |
|  | \_03\_ | 0.375000 | 0.071429 | 0.285714 | 0.125000 | 0.035714 | 0.035714 | 0.125000 | 0.160714 | 0.053571 | 0.071429 | 0.214286 | 0.035714 | 0.053571 | 0.285714 | 0.214286 | 0.267857 | 0.071429 | 0.553571 | 0.678571 | 0.017857 |
|  | \_04\_ | 0.696429 | 0.071429 | 0.410714 | 0.142857 | 0.053571 | 0.107143 | 0.196429 | 0.285714 | 0.089286 | 0.267857 | 0.750000 | 0.125000 | 0.035714 | 0.339286 | 0.321429 | 0.339286 | 0.035714 | 0.750000 | 0.732143 | 0.053571 |
|  | \_05\_ | 0.803571 | 0.178571 | 0.678571 | 0.392857 | 0.142857 | 0.464286 | 0.500000 | 0.571429 | 0.142857 | 0.321429 | 0.964286 | 0.267857 | 0.178571 | 0.464286 | 0.750000 | 0.517857 | 0.178571 | 0.910714 | 0.625000 | 0.142857 |
|  | \_06\_ | 0.928571 | 0.267857 | 0.785714 | 0.625000 | 0.535714 | 0.839286 | 0.732143 | 0.821429 | 0.500000 | 0.446429 | 1.000000 | 0.446429 | 0.446429 | 0.750000 | 0.964286 | 0.678571 | 0.410714 | 0.982143 | 0.910714 | 0.339286 |
|  | \_07\_ | 0.982143 | 0.535714 | 0.892857 | 0.767857 | 0.821429 | 0.910714 | 0.875000 | 0.892857 | 0.821429 | 0.767857 | 1.000000 | 0.553571 | 0.785714 | 0.839286 | 0.946429 | 0.839286 | 0.660714 | 1.000000 | 0.964286 | 0.660714 |
|  | \_08\_ | 1.000000 | 0.678571 | 1.000000 | 0.892857 | 0.892857 | 0.982143 | 0.982143 | 0.982143 | 0.982143 | 0.928571 | 0.982143 | 0.714286 | 0.946429 | 0.964286 | 0.982143 | 0.839286 | 0.803571 | 1.000000 | 1.000000 | 0.875000 |
|  | \_09\_ | 1.000000 | 0.892857 | 0.946429 | 0.892857 | 0.964286 | 0.928571 | 0.964286 | 0.964286 | 1.000000 | 0.946429 | 1.000000 | 0.857143 | 0.964286 | 0.946429 | 0.982143 | 0.928571 | 0.964286 | 1.000000 | 1.000000 | 0.946429 |
|  | \_10\_ | 1.000000 | 0.910714 | 0.982143 | 0.946429 | 1.000000 | 1.000000 | 0.946429 | 0.982143 | 0.982143 | 1.000000 | 1.000000 | 0.928571 | 0.982143 | 0.964286 | 1.000000 | 0.964286 | 0.946429 | 0.982143 | 1.000000 | 1.000000 |
| noe | \_00\_ | 0.214286 | 0.767857 | 0.500000 | 0.482143 | 0.035714 | 0.285714 | 0.053571 | 0.035714 | 0.089286 | 0.053571 | 0.250000 | 0.446429 | 0.089286 | 0.607143 | 0.285714 | 0.642857 | 0.160714 | 0.339286 | 0.196429 | 0.107143 |
|  | \_01\_ | 0.375000 | 0.750000 | 0.553571 | 0.500000 | 0.071429 | 0.321429 | 0.107143 | 0.071429 | 0.107143 | 0.089286 | 0.232143 | 0.535714 | 0.071429 | 0.642857 | 0.321429 | 0.589286 | 0.142857 | 0.375000 | 0.160714 | 0.160714 |
|  | \_02\_ | 0.375000 | 0.642857 | 0.535714 | 0.589286 | 0.071429 | 0.375000 | 0.089286 | 0.089286 | 0.160714 | 0.142857 | 0.535714 | 0.535714 | 0.089286 | 0.714286 | 0.357143 | 0.660714 | 0.125000 | 0.500000 | 0.142857 | 0.232143 |
|  | \_03\_ | 0.535714 | 0.750000 | 0.517857 | 0.571429 | 0.125000 | 0.375000 | 0.160714 | 0.107143 | 0.232143 | 0.071429 | 0.678571 | 0.660714 | 0.107143 | 0.732143 | 0.410714 | 0.625000 | 0.125000 | 0.517857 | 0.214286 | 0.107143 |
|  | \_04\_ | 0.642857 | 0.750000 | 0.553571 | 0.642857 | 0.107143 | 0.500000 | 0.232143 | 0.125000 | 0.339286 | 0.214286 | 0.875000 | 0.625000 | 0.142857 | 0.750000 | 0.517857 | 0.750000 | 0.250000 | 0.642857 | 0.267857 | 0.303571 |
|  | \_05\_ | 0.517857 | 0.785714 | 0.464286 | 0.875000 | 0.321429 | 0.482143 | 0.392857 | 0.250000 | 0.464286 | 0.160714 | 0.964286 | 0.589286 | 0.267857 | 0.767857 | 0.875000 | 0.803571 | 0.196429 | 0.821429 | 0.267857 | 0.321429 |
|  | \_06\_ | 0.732143 | 0.821429 | 0.607143 | 0.875000 | 0.571429 | 0.714286 | 0.428571 | 0.267857 | 0.642857 | 0.339286 | 0.964286 | 0.660714 | 0.428571 | 0.821429 | 0.982143 | 0.839286 | 0.357143 | 0.964286 | 0.428571 | 0.500000 |
|  | \_07\_ | 0.696429 | 0.767857 | 0.517857 | 0.875000 | 0.714286 | 0.696429 | 0.642857 | 0.571429 | 0.714286 | 0.535714 | 0.982143 | 0.732143 | 0.535714 | 0.821429 | 0.964286 | 0.803571 | 0.357143 | 0.875000 | 0.660714 | 0.535714 |
|  | \_08\_ | 0.839286 | 0.839286 | 0.642857 | 0.964286 | 0.803571 | 0.892857 | 0.696429 | 0.571429 | 0.750000 | 0.500000 | 0.964286 | 0.839286 | 0.625000 | 0.803571 | 0.946429 | 0.857143 | 0.375000 | 0.910714 | 0.785714 | 0.642857 |
|  | \_09\_ | 0.875000 | 0.821429 | 0.642857 | 0.910714 | 0.875000 | 0.785714 | 0.750000 | 0.839286 | 0.750000 | 0.642857 | 1.000000 | 0.803571 | 0.607143 | 0.732143 | 0.964286 | 0.821429 | 0.428571 | 0.946429 | 0.785714 | 0.750000 |
|  | \_10\_ | 0.910714 | 0.767857 | 0.660714 | 0.928571 | 0.875000 | 0.821429 | 0.750000 | 0.821429 | 0.767857 | 0.678571 | 0.982143 | 0.857143 | 0.714286 | 0.642857 | 0.964286 | 0.857143 | 0.410714 | 1.000000 | 0.839286 | 0.732143 |
| nom | \_00\_ | 0.142857 | 0.053571 | 0.160714 | 0.053571 | 0.035714 | 0.017857 | 0.053571 | 0.142857 | 0.035714 | 0.089286 | 0.089286 | 0.035714 | 0.089286 | 0.142857 | 0.089286 | 0.142857 | 0.035714 | 0.482143 | 0.571429 | 0.053571 |
|  | \_01\_ | 0.196429 | 0.017857 | 0.285714 | 0.017857 | 0.000000 | 0.053571 | 0.160714 | 0.410714 | 0.071429 | 0.125000 | 0.125000 | 0.017857 | 0.017857 | 0.178571 | 0.053571 | 0.142857 | 0.035714 | 0.642857 | 0.607143 | 0.107143 |
|  | \_02\_ | 0.107143 | 0.125000 | 0.321429 | 0.035714 | 0.035714 | 0.035714 | 0.196429 | 0.464286 | 0.142857 | 0.178571 | 0.285714 | 0.035714 | 0.071429 | 0.142857 | 0.089286 | 0.232143 | 0.089286 | 0.678571 | 0.750000 | 0.053571 |
|  | \_03\_ | 0.357143 | 0.107143 | 0.321429 | 0.017857 | 0.071429 | 0.017857 | 0.303571 | 0.625000 | 0.125000 | 0.267857 | 0.571429 | 0.089286 | 0.107143 | 0.214286 | 0.107143 | 0.125000 | 0.160714 | 0.767857 | 0.875000 | 0.178571 |
|  | \_04\_ | 0.500000 | 0.053571 | 0.535714 | 0.071429 | 0.107143 | 0.107143 | 0.446429 | 0.750000 | 0.142857 | 0.339286 | 0.803571 | 0.071429 | 0.196429 | 0.160714 | 0.267857 | 0.178571 | 0.267857 | 0.892857 | 0.821429 | 0.178571 |
|  | \_05\_ | 0.517857 | 0.125000 | 0.607143 | 0.107143 | 0.089286 | 0.553571 | 0.553571 | 0.839286 | 0.285714 | 0.392857 | 1.000000 | 0.071429 | 0.500000 | 0.250000 | 0.410714 | 0.285714 | 0.446429 | 0.928571 | 0.928571 | 0.214286 |
|  | \_06\_ | 0.696429 | 0.285714 | 0.803571 | 0.357143 | 0.303571 | 0.785714 | 0.696429 | 0.928571 | 0.428571 | 0.607143 | 1.000000 | 0.142857 | 0.696429 | 0.410714 | 0.535714 | 0.446429 | 0.589286 | 0.982143 | 0.982143 | 0.428571 |
|  | \_07\_ | 0.892857 | 0.464286 | 0.839286 | 0.410714 | 0.589286 | 0.875000 | 0.875000 | 0.928571 | 0.785714 | 0.875000 | 1.000000 | 0.339286 | 0.910714 | 0.642857 | 0.732143 | 0.535714 | 0.785714 | 0.946429 | 0.964286 | 0.785714 |
|  | \_08\_ | 0.946429 | 0.625000 | 1.000000 | 0.696429 | 0.821429 | 0.946429 | 0.964286 | 0.946429 | 0.910714 | 0.964286 | 1.000000 | 0.589286 | 0.946429 | 0.660714 | 0.910714 | 0.750000 | 0.946429 | 0.964286 | 0.982143 | 0.857143 |
|  | \_09\_ | 0.964286 | 0.785714 | 0.964286 | 0.910714 | 0.946429 | 0.964286 | 0.946429 | 0.964286 | 0.946429 | 0.982143 | 0.982143 | 0.696429 | 0.964286 | 0.696429 | 0.946429 | 0.892857 | 0.946429 | 0.964286 | 0.946429 | 1.000000 |
|  | \_10\_ | 0.982143 | 0.803571 | 0.964286 | 0.892857 | 0.964286 | 1.000000 | 0.946429 | 0.982143 | 0.928571 | 1.000000 | 1.000000 | 0.803571 | 1.000000 | 0.767857 | 0.910714 | 0.857143 | 0.964286 | 1.000000 | 0.964286 | 1.000000 |

In [18]:

```
ident_df.describe()
```

Out[18]:

|  | F10\_ | F1\_ | F2\_ | F3\_ | F4\_ | F5\_ | F6\_ | F7\_ | F8\_ | F9\_ | M10\_ | M1\_ | M2\_ | M3\_ | M4\_ | M5\_ | M6\_ | M7\_ | M8\_ | M9\_ |
| --- | --- | --- | --- | --- | --- | --- | --- | --- | --- | --- | --- | --- | --- | --- | --- | --- | --- | --- | --- | --- |
| count | 33.000000 | 33.000000 | 33.000000 | 33.000000 | 33.000000 | 33.000000 | 33.000000 | 33.000000 | 33.000000 | 33.000000 | 33.000000 | 33.000000 | 33.000000 | 33.000000 | 33.000000 | 33.000000 | 33.000000 | 33.000000 | 33.000000 | 33.000000 |
| mean | 0.624459 | 0.475649 | 0.597944 | 0.503247 | 0.394481 | 0.513528 | 0.482684 | 0.537338 | 0.438312 | 0.431818 | 0.709957 | 0.431818 | 0.413961 | 0.555736 | 0.577922 | 0.571429 | 0.377706 | 0.760281 | 0.676948 | 0.404762 |
| std | 0.299057 | 0.334220 | 0.256031 | 0.363187 | 0.383289 | 0.373029 | 0.345384 | 0.356678 | 0.360035 | 0.337916 | 0.365882 | 0.316034 | 0.366981 | 0.282479 | 0.366148 | 0.283579 | 0.323102 | 0.257231 | 0.288607 | 0.346483 |
| min | 0.107143 | 0.017857 | 0.160714 | 0.000000 | 0.000000 | 0.017857 | 0.035714 | 0.035714 | 0.017857 | 0.053571 | 0.017857 | 0.017857 | 0.017857 | 0.089286 | 0.017857 | 0.125000 | 0.035714 | 0.178571 | 0.142857 | 0.017857 |
| 25% | 0.375000 | 0.107143 | 0.410714 | 0.107143 | 0.053571 | 0.107143 | 0.160714 | 0.160714 | 0.107143 | 0.125000 | 0.285714 | 0.071429 | 0.089286 | 0.250000 | 0.267857 | 0.267857 | 0.125000 | 0.553571 | 0.428571 | 0.107143 |
| 50% | 0.696429 | 0.625000 | 0.553571 | 0.571429 | 0.142857 | 0.500000 | 0.446429 | 0.571429 | 0.339286 | 0.339286 | 0.964286 | 0.535714 | 0.267857 | 0.642857 | 0.535714 | 0.642857 | 0.267857 | 0.892857 | 0.750000 | 0.303571 |
| 75% | 0.910714 | 0.767857 | 0.803571 | 0.875000 | 0.821429 | 0.875000 | 0.750000 | 0.892857 | 0.767857 | 0.678571 | 1.000000 | 0.696429 | 0.714286 | 0.767857 | 0.946429 | 0.839286 | 0.589286 | 0.964286 | 0.946429 | 0.732143 |
| max | 1.000000 | 0.910714 | 1.000000 | 0.964286 | 1.000000 | 1.000000 | 0.982143 | 0.982143 | 1.000000 | 1.000000 | 1.000000 | 0.928571 | 1.000000 | 0.964286 | 1.000000 | 0.964286 | 0.964286 | 1.000000 | 1.000000 | 1.000000 |

## Adopt the Functions form S4¶

Please refer to S4 for explainations of these functions

In [26]:

```
def logistic(x,a,b):
    y = 1 / (1 + np.exp(-b*(x-a)))
    return y
```

In [27]:

```
def fitFunc(func,ydata):
    # for all designs in this study, there are 11 morphing steps, scaled between 0 and 1
    x = np.arange(0,len(ydata)/10.,0.1)
    # the scipy curvefit function is used here
    popt, pcov = curve_fit(func, x, ydata, maxfev=100000)

    intercept = popt[0] # intercept
    slope = popt[1]     # slope
    
    return intercept,slope
```

In [28]:

```
def transformData(thisList):
    # the starting values are choses in such a way,
    # that the lowest value can only decrease (starts high)
    # and the highest value can only increase (starts low)
    lowest = 100
    highest = 0

    # we move through the list and update
    # the highest and lowest value, once we
    # encounter an entry that is higher or lower, respectively
    for x in thisList:
        if x < lowest:
            lowest = x
        if x > highest:       
            highest = x
    
    # the difference between highest and lowest is the span
    span = highest-lowest

    # the new rescaled data are written
    newList = []
    for x in thisList:
        # we get the distance from the lowest point and
        # rescale by the span of highest-lowest
        # e.g. the lowest point is 5 the highest is 10
        # the value 6 will be rescaled to (6-5)/5 = 1/5 = 20%
        newPoint = (float(x)-lowest)/span
        newList.append(newPoint)
        
    return newList,lowest,span
```

In [29]:

```
def transformBack(newList,lowest,span):
    oldList = []
    for x in newList:
        oldPoint = float(x)*span+lowest
        oldList.append(oldPoint)
    return oldList
```

In [30]:

```
def rescaledFunc(thisData):
    #rescale the data
    newData,lowest,span = transformData(thisData)
    # fit a logistic function to the rescaled data
    # and extract intercept and slope of the function
    intercept,slope=fitFunc(logistic,newData)
    # use these parameters to make a logistic curve
    logData = []
    for x in np.arange(0,1.0001,0.0001):
        logData.append(logistic(x,intercept,slope))
    
    # scale the logistic curve back to the original dimensions
    nativeLog = transformBack(logData,lowest,span)
    
    return nativeLog
```

In [31]:

```
def logDict(df,cond,func=rescaledFunc):
    d={}
    for ident in df.columns:
        d[ident]= func(df.ix[cond][ident])
    return d
```

In [32]:

```
def showCurve(curveDict,rgb,myTitle,myX,xLab,yLab):
    for p in curveDict:
        plot(curveDict[p],
             color=rgb,
             alpha=0.5,
             linewidth=4
            )
    title(myTitle)
    xticks(myX[0],myX[1])
    xlabel(xLab)
    ylabel(yLab)
    sns.despine()
    show()
```

In [33]:

```
origDict = logDict(ident_df,'orig')
```

visualise the original fits

In [34]:

```
for entry in origDict:
    plot(origDict[entry])
```

In [35]:

```
def getDeriv(thisList):
    derivList = []
    for index,x in enumerate(thisList):
        try:
            thisSlope = (float(thisList[index+1])-thisList[index])/(1./len(thisList)) 
            derivList.append(thisSlope)
        # this should not work with the very last item only
        except:
            # if it doesnt work in any other case, throw and exception
            assert index == len(thisList)-1, 'something went wrong'
    return derivList
```

In [36]:

```
def showDeriv(curveDict,rgb,myTitle,myX,xLab,yLab):
    for p in curveDict:
        plot(
            getDeriv(curveDict[p]),
            color=rgb,
            alpha=0.5,
            linewidth=4
            )
    title(myTitle)
    xticks(myX[0],myX[1])
    xlabel(xLab)
    ylabel(yLab)
    ylim(0,3.75)
    sns.despine()
    show()
```

In [37]:

```
# global color palette
myPalette=['#ee4035','#4f5b66','#325da7','#da6b30']
```

In [42]:

```
def showAllDeriv(df,condOrder,colors=myPalette,func=rescaledFunc):
    for index,cond in enumerate(condOrder):
        condDict = logDict(df,cond,func=func)
        showDeriv(condDict,
                  colors[index],
                  cond,
                  [np.arange(0,10001,1000),np.arange(0,1.01,0.1)],
                  'morphing grade',
                  'steepness of slope'
                 )
```

In [43]:

```
def getPeak(d,p):
    highestVal = -999
    highestPos = -999
    for index,i in enumerate(getDeriv(d[p])):
        if i > highestVal:
            highestVal = i
            highestPos = index
    return highestPos,highestVal
```

In [44]:

```
def logDict(df,cond,func=rescaledFunc):
    d={}
    for ident in df.columns:
        d[ident]= func(df.ix[cond][ident])
    return d
```

In [45]:

```
def peakDict(df,func=rescaledFunc):
    d = {}
    for index,cond in enumerate(df.index.levels[0]):
        d[cond] = {}
        condDict = logDict(df,cond,func=func)
        for p in condDict:
            intercept,slope = getPeak(condDict,p)
            # get value on y-axis where slope is maximal
            # we consider this the bias - at which height (y-axis)
            # is the intercept localted:
            bias = condDict[p][intercept]
            # rescale intercept to value between 0 and 1
            intercept = float(intercept)/len(condDict[p])

            d[cond][p] = {'slope':slope,'intercept':intercept,'bias':bias}
        
    return d
```

In [46]:

```
def peakDf(d):
    for cond in d:
        thisDf = pd.DataFrame(d[cond]).T
        thisDf.index=[[cond]*len(thisDf),thisDf.index]
        try:
            bigDf = pd.concat([bigDf,thisDf])
        except:
            bigDf = thisDf

    return bigDf.unstack(0)
```

In [47]:

```
exp1LogFuncTable = peakDf(peakDict(ident_df))
```

### here, the actual shifting is done¶

We know all the threshold values and also the mean threshold value. So we can get the amount we need to shift each individual data to the mean threshold by computing the difference between the mean threshold and the individual threshold and add that to all the data.

In [49]:

```
def shiftRaw(rawDf,paramDf):
    
    # empty dict to write to
    d = {}

    # loop through all masking conditions
    # cave: this df is structured differently than the main results
    for cond in rawDf.index.levels[0]:
        # make a dict for this condition
        d[cond] = {}
        # get the mean parameters of all fitted functions
        meanThresh = paramDf['intercept'][cond].mean()
        meanBias = paramDf['bias'][cond].mean()
        
        # get an array with the current data
        thisCond = rawDf.ix[cond].T

        for p in thisCond.index:

            # getting parameters of the present fitted function
            thisThresh = paramDf['intercept'][cond].ix[p]
            thisBias = paramDf['bias'][cond].ix[p]
            # compute how much the paramters need to be shifted
            diffThresh = meanThresh-thisThresh
            diffBias = meanBias-thisBias
            # the shifting of the thresholds requires to actually
            # change the values of the keys in the dict, so we
            # need to rename the keys to be integer-esque
            thisIdent = rawDf.ix[cond][p]
            myIndex = np.arange(0,1.01,0.1)
            thisIdent.index = myIndex
            thisDict= thisIdent.to_dict()
            
            # we shift the values of the current response curve
            # by modifying the key (x-axis) and the value (y-axis)
            d[cond][p] = {}
            for key,entry in thisDict.items():
                d[cond][p][key+diffThresh] = entry+diffBias
                
    return d
```

In [50]:

```
exp1Shifted = shiftRaw(ident_df,exp1LogFuncTable)
```

Example:

In [51]:

```
exp1Shifted['orig']['F1_']
```

Out[51]:

```
{-0.15665433456654343: 0.11025130848169876,
 -0.056654334566543424: 0.092394165624555893,
 0.043345665433456582: 0.14596559419598448,
 0.14334566543345661: 0.12810845133884161,
 0.24334566543345659: 0.12810845133884161,
 0.34334566543345657: 0.23525130848169876,
 0.44334566543345666: 0.32453702276741303,
 0.54334566543345664: 0.59239416562455594,
 0.64334566543345661: 0.73525130848169873,
 0.74334566543345659: 0.94953702276741314,
 0.84334566543345657: 0.96739416562455594}
```

### Spaghetti Plots of shifted raw data¶

vertical lines show the original response range (0 to 1). Responses that are shifted outside this range could be thought of as being responses to (imaginary) carricatures, that are more than 100% fearful or more than 100% angry.

In [54]:

```
def singleShiftPlots(d):
    for cond in d:
        print cond
        thisDf = pd.DataFrame(d[cond])
        for ident in thisDf:
            x = []
            y = []
            for index,entry in enumerate(thisDf[ident]):
                if entry > 0:
                    x.append(thisDf['F10_'].index[index])
                    y.append(entry)
            plot(x,y)
        sns.despine()
        axvline(0,linewidth=1, color='k');axvline(1,linewidth=1, color='k')
        show()
```

In [55]:

```
singleShiftPlots(exp1Shifted)
```

```
noe
```

```
orig
```

```
nom
```

## Transform fine-grainded x-axis into 11 bins¶

Looping through the dict and checking for each key, into which bin it would belong. The value is appended to the respective bin.  
Subsequently, descriptive statistics are computed.

Since no pooling of data would mean no estimate of variance, this is a necessary step, although all small shifts have no effect (as the data stay in the same bin if the shift is smaller than 5%)

In [56]:

```
def makeBins(d):
    
    dBin = {}

    for cond in d:
        dBin[cond] = {0:[],1:[],2:[],3:[],4:[],5:[],6:[],7:[],8:[],9:[],10:[]}
        for ident in d[cond]:
            for key in d[cond][ident]:
                if key >=-0.05 and key <0.05:
                    dBin[cond][0].append(d[cond][ident][key])
                elif key >=0.05 and key <0.15:
                    dBin[cond][1].append(d[cond][ident][key])
                elif key >=0.15 and key <0.25:
                    dBin[cond][2].append(d[cond][ident][key])
                elif key >=0.25 and key <0.35:
                    dBin[cond][3].append(d[cond][ident][key])
                elif key >=0.35 and key <0.45:
                    dBin[cond][4].append(d[cond][ident][key])
                elif key >=0.45 and key <0.55:
                    dBin[cond][5].append(d[cond][ident][key])
                elif key >=0.55 and key <0.65:
                    dBin[cond][6].append(d[cond][ident][key])
                elif key >=0.65 and key <0.75:
                    dBin[cond][7].append(d[cond][ident][key])
                elif key >=0.75 and key <0.85:
                    dBin[cond][8].append(d[cond][ident][key])
                elif key >=0.85 and key <0.95:
                    dBin[cond][9].append(d[cond][ident][key])
                elif key >=0.95 and key <1.05:
                    dBin[cond][10].append(d[cond][ident][key])
    
    return dBin
```

In [57]:

```
exp1Bins = makeBins(exp1Shifted)
```

In [58]:

```
myPalette=['#325da7','#4f5b66','#ee4035']
myTicks = ['0%','10%','20%','30%','40%','50%','60%','70%','80%','90%','100%']
```

In [59]:

```
def makeBinPlot(d):

    for index,cond in enumerate(d):
        thisMean =[]
        thisCi = []
        
        for x in d[cond]:
            thisMean.append(mean(d[cond][x]))
            thisCi.append(std(d[cond][x])/sqrt(len(d[cond][x]))*1.96)
            
        errorbar(range(11),thisMean,yerr=thisCi,color=myPalette[index],label=cond)
        
    xlim(-0.25,10.25)
    xticks(np.arange(0,11,1),myTicks)
    yticks(np.arange(0,1.1,0.1),myTicks)
        
    sns.despine()
    
    xlabel('Morphing Grade (%Angry)\n(95% CI)')
    ylabel('Response (Angry)')
    
    legend(loc='best')
    
    savefig('exp2_identity.png',dpi=600)
    show()
```

In [60]:

```
makeBinPlot(exp1Bins)
```

# Experiment 2¶

Get Data of Experiment 2

In [61]:

```
List = getsubject("/home/martin/FACES_PARAMETRIC/SUPPLEMENT/RAWDATA/Exp2/", "*Vp*.log")
List.sort()
```

Exclude outlier

In [63]:

```
Exp2RevList = List[:13] + List[14:]
```

In [64]:

```
Exp2RevList
```

Out[64]:

```
['Vp001-Vp001_UpDown_Identification.log',
 'Vp002-Vp002_UpDown_Identification.log',
 'Vp003-Vp003_UpDown_Identification.log',
 'Vp004-Vp004_UpDown_Identification.log',
 'Vp005-Vp005_UpDown_Identification.log',
 'Vp006-Vp006_UpDown_Identification.log',
 'Vp007-Vp007_UpDown_Identification.log',
 'Vp008-Vp008_UpDown_Identification.log',
 'Vp009-Vp009_UpDown_Identification.log',
 'Vp010-Vp010_UpDown_Identification.log',
 'Vp011-Vp011_UpDown_Identification.log',
 'Vp012-Vp012_UpDown_Identification.log',
 'Vp013-Vp013_UpDown_Identification.log',
 'Vp015-Vp015_UpDown_Identification.log',
 'Vp016-Vp016_UpDown_Identification.log',
 'Vp017-Vp017_UpDown_Identification.log',
 'Vp018-Vp018_UpDown_Identification.log',
 'Vp019-Vp019_UpDown_Identification.log',
 'Vp020-Vp020_UpDown_Identification.log',
 'Vp021-Vp021_UpDown_Identification.log',
 'Vp022-Vp022_UpDown_Identification.log',
 'Vp023-Vp023_UpDown_Identification.log',
 'Vp024-Vp024_UpDown_Identification.log',
 'Vp025-Vp025_UpDown_Identification.log',
 'Vp026-Vp026_UpDown_Identification.log',
 'Vp027-Vp027_UpDown_Identification.log',
 'Vp028-Vp028_UpDown_Identification.log',
 'Vp029-Vp029_UpDown_Identification.log',
 'Vp030-Vp030_UpDown_Identification.log']
```

Define Condition Names for Experiment 2

In [65]:

```
conds = ['00_UPPER','10_UPPER','00_LOWER','10_LOWER']
```

Compute Results for Experiment 2

In [66]:

```
exp2_results = all_files(Exp2RevList,conds)
```

Transform into Data Frame

In [67]:

```
fear_eyes_df = pd.DataFrame(exp2_results['00_UPPER'])
fear_eyes_df.index = [['00_UPPER']*11,list(fear_eyes_df.index)]

anger_eyes_df = pd.DataFrame(exp2_results['10_UPPER'])
anger_eyes_df.index = [['10_UPPER']*11,list(anger_eyes_df.index)]

fear_mouth_df = pd.DataFrame(exp2_results['00_LOWER'])
fear_mouth_df.index = [['00_LOWER']*11,list(fear_mouth_df.index)]

anger_mouth_df = pd.DataFrame(exp2_results['10_LOWER'])
anger_mouth_df.index = [['10_LOWER']*11,list(anger_mouth_df.index)]

n_of_participants = 29 # for sample with N=29
exp2_ident_df = pd.concat([fear_eyes_df,anger_eyes_df,fear_mouth_df,anger_mouth_df],axis = 0)
exp2_ident_df = exp2_ident_df/(n_of_participants*2.0) # convert probability into percent (there are N subjects, each providing 2 responses)
```

### Table with Identity Data¶

In [71]:

```
exp2_ident_df
```

Out[71]:

|  |  | F10\_ | F1\_ | F2\_ | F3\_ | F4\_ | F5\_ | F6\_ | F7\_ | F8\_ | F9\_ | M10\_ | M1\_ | M2\_ | M3\_ | M4\_ | M5\_ | M6\_ | M7\_ | M8\_ | M9\_ |
| --- | --- | --- | --- | --- | --- | --- | --- | --- | --- | --- | --- | --- | --- | --- | --- | --- | --- | --- | --- | --- | --- |
| 00\_UPPER | \_00\_ | 0.448276 | 0.379310 | 0.258621 | 0.327586 | 0.120690 | 0.275862 | 0.068966 | 0.155172 | 0.206897 | 0.172414 | 0.327586 | 0.413793 | 0.155172 | 0.327586 | 0.258621 | 0.465517 | 0.155172 | 0.534483 | 0.431034 | 0.155172 |
|  | \_01\_ | 0.517241 | 0.413793 | 0.362069 | 0.396552 | 0.103448 | 0.293103 | 0.206897 | 0.120690 | 0.137931 | 0.224138 | 0.310345 | 0.396552 | 0.137931 | 0.413793 | 0.362069 | 0.637931 | 0.155172 | 0.586207 | 0.379310 | 0.137931 |
|  | \_02\_ | 0.568966 | 0.431034 | 0.293103 | 0.310345 | 0.172414 | 0.362069 | 0.189655 | 0.224138 | 0.224138 | 0.206897 | 0.344828 | 0.413793 | 0.172414 | 0.396552 | 0.344828 | 0.568966 | 0.172414 | 0.517241 | 0.413793 | 0.155172 |
|  | \_03\_ | 0.534483 | 0.413793 | 0.344828 | 0.396552 | 0.103448 | 0.224138 | 0.206897 | 0.224138 | 0.241379 | 0.241379 | 0.500000 | 0.362069 | 0.137931 | 0.431034 | 0.431034 | 0.448276 | 0.172414 | 0.603448 | 0.327586 | 0.103448 |
|  | \_04\_ | 0.620690 | 0.431034 | 0.362069 | 0.465517 | 0.172414 | 0.396552 | 0.275862 | 0.310345 | 0.137931 | 0.155172 | 0.637931 | 0.327586 | 0.103448 | 0.413793 | 0.500000 | 0.517241 | 0.206897 | 0.724138 | 0.413793 | 0.189655 |
|  | \_05\_ | 0.586207 | 0.448276 | 0.327586 | 0.396552 | 0.224138 | 0.362069 | 0.310345 | 0.275862 | 0.344828 | 0.189655 | 0.844828 | 0.431034 | 0.206897 | 0.586207 | 0.465517 | 0.603448 | 0.189655 | 0.758621 | 0.413793 | 0.275862 |
|  | \_06\_ | 0.586207 | 0.327586 | 0.310345 | 0.500000 | 0.448276 | 0.482759 | 0.275862 | 0.327586 | 0.431034 | 0.241379 | 0.827586 | 0.413793 | 0.224138 | 0.448276 | 0.568966 | 0.482759 | 0.172414 | 0.793103 | 0.465517 | 0.258621 |
|  | \_07\_ | 0.758621 | 0.344828 | 0.379310 | 0.534483 | 0.431034 | 0.500000 | 0.310345 | 0.379310 | 0.310345 | 0.396552 | 0.931034 | 0.517241 | 0.293103 | 0.465517 | 0.672414 | 0.534483 | 0.172414 | 0.896552 | 0.620690 | 0.293103 |
|  | \_08\_ | 0.810345 | 0.465517 | 0.327586 | 0.517241 | 0.517241 | 0.482759 | 0.413793 | 0.500000 | 0.293103 | 0.241379 | 0.896552 | 0.534483 | 0.275862 | 0.448276 | 0.741379 | 0.534483 | 0.189655 | 0.793103 | 0.586207 | 0.293103 |
|  | \_09\_ | 0.793103 | 0.344828 | 0.344828 | 0.482759 | 0.620690 | 0.465517 | 0.379310 | 0.620690 | 0.327586 | 0.344828 | 0.913793 | 0.534483 | 0.206897 | 0.448276 | 0.689655 | 0.603448 | 0.137931 | 0.879310 | 0.655172 | 0.379310 |
|  | \_10\_ | 0.793103 | 0.344828 | 0.379310 | 0.551724 | 0.672414 | 0.603448 | 0.344828 | 0.620690 | 0.465517 | 0.431034 | 0.931034 | 0.465517 | 0.379310 | 0.379310 | 0.706897 | 0.620690 | 0.241379 | 0.844828 | 0.689655 | 0.344828 |
| 10\_UPPER | \_00\_ | 0.879310 | 0.603448 | 0.724138 | 0.517241 | 0.517241 | 0.551724 | 0.534483 | 0.413793 | 0.448276 | 0.586207 | 0.655172 | 0.689655 | 0.500000 | 0.586207 | 0.568966 | 0.724138 | 0.448276 | 0.758621 | 0.568966 | 0.431034 |
|  | \_01\_ | 0.896552 | 0.672414 | 0.689655 | 0.603448 | 0.482759 | 0.482759 | 0.448276 | 0.448276 | 0.465517 | 0.500000 | 0.775862 | 0.706897 | 0.482759 | 0.517241 | 0.637931 | 0.793103 | 0.534483 | 0.793103 | 0.482759 | 0.482759 |
|  | \_02\_ | 0.931034 | 0.568966 | 0.706897 | 0.603448 | 0.500000 | 0.568966 | 0.534483 | 0.362069 | 0.517241 | 0.500000 | 0.758621 | 0.672414 | 0.482759 | 0.568966 | 0.500000 | 0.758621 | 0.465517 | 0.827586 | 0.500000 | 0.517241 |
|  | \_03\_ | 0.810345 | 0.689655 | 0.637931 | 0.637931 | 0.586207 | 0.689655 | 0.500000 | 0.362069 | 0.431034 | 0.551724 | 0.827586 | 0.741379 | 0.500000 | 0.568966 | 0.568966 | 0.706897 | 0.413793 | 0.862069 | 0.500000 | 0.551724 |
|  | \_04\_ | 0.810345 | 0.689655 | 0.706897 | 0.620690 | 0.655172 | 0.689655 | 0.586207 | 0.534483 | 0.568966 | 0.620690 | 0.913793 | 0.758621 | 0.465517 | 0.586207 | 0.793103 | 0.706897 | 0.431034 | 0.913793 | 0.586207 | 0.568966 |
|  | \_05\_ | 0.844828 | 0.534483 | 0.706897 | 0.620690 | 0.534483 | 0.810345 | 0.637931 | 0.586207 | 0.741379 | 0.603448 | 0.913793 | 0.741379 | 0.603448 | 0.724138 | 0.844828 | 0.758621 | 0.431034 | 0.948276 | 0.500000 | 0.517241 |
|  | \_06\_ | 0.844828 | 0.689655 | 0.706897 | 0.706897 | 0.862069 | 0.741379 | 0.620690 | 0.706897 | 0.741379 | 0.603448 | 0.931034 | 0.724138 | 0.534483 | 0.741379 | 0.931034 | 0.741379 | 0.482759 | 0.931034 | 0.603448 | 0.620690 |
|  | \_07\_ | 0.827586 | 0.603448 | 0.741379 | 0.655172 | 0.827586 | 0.793103 | 0.689655 | 0.655172 | 0.775862 | 0.637931 | 0.982759 | 0.758621 | 0.655172 | 0.775862 | 0.879310 | 0.724138 | 0.500000 | 0.931034 | 0.810345 | 0.568966 |
|  | \_08\_ | 0.896552 | 0.741379 | 0.758621 | 0.724138 | 0.793103 | 0.862069 | 0.655172 | 0.741379 | 0.793103 | 0.706897 | 0.913793 | 0.793103 | 0.603448 | 0.637931 | 0.862069 | 0.741379 | 0.568966 | 0.931034 | 0.775862 | 0.724138 |
|  | \_09\_ | 0.913793 | 0.620690 | 0.689655 | 0.758621 | 0.862069 | 0.827586 | 0.741379 | 0.775862 | 0.793103 | 0.672414 | 0.931034 | 0.706897 | 0.689655 | 0.655172 | 0.913793 | 0.758621 | 0.551724 | 0.948276 | 0.810345 | 0.706897 |
|  | \_10\_ | 0.948276 | 0.517241 | 0.775862 | 0.706897 | 0.879310 | 0.844828 | 0.844828 | 0.689655 | 0.827586 | 0.672414 | 0.965517 | 0.758621 | 0.724138 | 0.672414 | 0.931034 | 0.844828 | 0.534483 | 0.965517 | 0.844828 | 0.689655 |
| 00\_LOWER | \_00\_ | 0.275862 | 0.051724 | 0.258621 | 0.137931 | 0.068966 | 0.120690 | 0.189655 | 0.172414 | 0.103448 | 0.137931 | 0.172414 | 0.051724 | 0.086207 | 0.155172 | 0.086207 | 0.258621 | 0.120690 | 0.413793 | 0.603448 | 0.068966 |
|  | \_01\_ | 0.362069 | 0.034483 | 0.189655 | 0.103448 | 0.068966 | 0.086207 | 0.086207 | 0.293103 | 0.172414 | 0.172414 | 0.120690 | 0.068966 | 0.068966 | 0.224138 | 0.120690 | 0.172414 | 0.120690 | 0.534483 | 0.603448 | 0.086207 |
|  | \_02\_ | 0.448276 | 0.086207 | 0.275862 | 0.137931 | 0.086207 | 0.120690 | 0.206897 | 0.344828 | 0.189655 | 0.241379 | 0.120690 | 0.068966 | 0.086207 | 0.189655 | 0.137931 | 0.189655 | 0.172414 | 0.534483 | 0.741379 | 0.068966 |
|  | \_03\_ | 0.517241 | 0.034483 | 0.310345 | 0.103448 | 0.120690 | 0.068966 | 0.206897 | 0.431034 | 0.189655 | 0.327586 | 0.293103 | 0.137931 | 0.172414 | 0.241379 | 0.120690 | 0.224138 | 0.258621 | 0.568966 | 0.775862 | 0.120690 |
|  | \_04\_ | 0.551724 | 0.086207 | 0.362069 | 0.137931 | 0.137931 | 0.241379 | 0.310345 | 0.517241 | 0.224138 | 0.362069 | 0.465517 | 0.120690 | 0.206897 | 0.327586 | 0.241379 | 0.258621 | 0.189655 | 0.655172 | 0.810345 | 0.172414 |
|  | \_05\_ | 0.586207 | 0.258621 | 0.586207 | 0.224138 | 0.137931 | 0.500000 | 0.327586 | 0.827586 | 0.206897 | 0.517241 | 0.741379 | 0.189655 | 0.310345 | 0.224138 | 0.310345 | 0.310345 | 0.344828 | 0.758621 | 0.844828 | 0.137931 |
|  | \_06\_ | 0.724138 | 0.362069 | 0.741379 | 0.206897 | 0.241379 | 0.534483 | 0.482759 | 0.655172 | 0.379310 | 0.448276 | 0.948276 | 0.396552 | 0.517241 | 0.379310 | 0.465517 | 0.482759 | 0.396552 | 0.775862 | 0.862069 | 0.310345 |
|  | \_07\_ | 0.810345 | 0.534483 | 0.810345 | 0.362069 | 0.517241 | 0.758621 | 0.603448 | 0.741379 | 0.500000 | 0.689655 | 0.965517 | 0.603448 | 0.706897 | 0.534483 | 0.603448 | 0.500000 | 0.586207 | 0.879310 | 0.913793 | 0.551724 |
|  | \_08\_ | 0.948276 | 0.534483 | 0.879310 | 0.534483 | 0.586207 | 0.896552 | 0.689655 | 0.810345 | 0.637931 | 0.724138 | 0.896552 | 0.603448 | 0.724138 | 0.517241 | 0.758621 | 0.620690 | 0.689655 | 0.879310 | 0.862069 | 0.637931 |
|  | \_09\_ | 0.931034 | 0.672414 | 0.879310 | 0.586207 | 0.793103 | 0.793103 | 0.793103 | 0.827586 | 0.741379 | 0.810345 | 0.913793 | 0.775862 | 0.844828 | 0.586207 | 0.844828 | 0.637931 | 0.844828 | 0.827586 | 0.913793 | 0.775862 |
|  | \_10\_ | 0.879310 | 0.741379 | 0.827586 | 0.706897 | 0.931034 | 0.844828 | 0.879310 | 0.862069 | 0.827586 | 0.931034 | 0.913793 | 0.844828 | 0.862069 | 0.586207 | 0.862069 | 0.758621 | 0.913793 | 0.931034 | 0.931034 | 0.879310 |
| 10\_LOWER | \_00\_ | 0.534483 | 0.086207 | 0.206897 | 0.120690 | 0.172414 | 0.120690 | 0.155172 | 0.465517 | 0.137931 | 0.241379 | 0.672414 | 0.103448 | 0.120690 | 0.120690 | 0.172414 | 0.258621 | 0.068966 | 0.758621 | 0.724138 | 0.086207 |
|  | \_01\_ | 0.534483 | 0.068966 | 0.293103 | 0.172414 | 0.224138 | 0.103448 | 0.258621 | 0.500000 | 0.206897 | 0.258621 | 0.706897 | 0.086207 | 0.086207 | 0.327586 | 0.189655 | 0.327586 | 0.086207 | 0.706897 | 0.620690 | 0.120690 |
|  | \_02\_ | 0.586207 | 0.086207 | 0.310345 | 0.137931 | 0.241379 | 0.155172 | 0.189655 | 0.672414 | 0.189655 | 0.310345 | 0.637931 | 0.155172 | 0.086207 | 0.241379 | 0.241379 | 0.310345 | 0.137931 | 0.741379 | 0.689655 | 0.051724 |
|  | \_03\_ | 0.672414 | 0.137931 | 0.379310 | 0.189655 | 0.241379 | 0.189655 | 0.344828 | 0.741379 | 0.275862 | 0.275862 | 0.793103 | 0.137931 | 0.137931 | 0.241379 | 0.275862 | 0.327586 | 0.120690 | 0.827586 | 0.775862 | 0.068966 |
|  | \_04\_ | 0.758621 | 0.241379 | 0.431034 | 0.258621 | 0.293103 | 0.275862 | 0.534483 | 0.862069 | 0.310345 | 0.431034 | 0.879310 | 0.189655 | 0.137931 | 0.431034 | 0.344828 | 0.293103 | 0.224138 | 0.810345 | 0.879310 | 0.155172 |
|  | \_05\_ | 0.844828 | 0.189655 | 0.534483 | 0.344828 | 0.465517 | 0.655172 | 0.620690 | 0.775862 | 0.465517 | 0.500000 | 0.948276 | 0.258621 | 0.275862 | 0.362069 | 0.551724 | 0.431034 | 0.293103 | 0.896552 | 0.982759 | 0.241379 |
|  | \_06\_ | 0.844828 | 0.362069 | 0.827586 | 0.413793 | 0.603448 | 0.741379 | 0.810345 | 0.844828 | 0.586207 | 0.637931 | 0.965517 | 0.448276 | 0.482759 | 0.517241 | 0.655172 | 0.482759 | 0.568966 | 0.879310 | 0.913793 | 0.344828 |
|  | \_07\_ | 0.913793 | 0.637931 | 0.879310 | 0.534483 | 0.793103 | 0.758621 | 0.827586 | 0.896552 | 0.810345 | 0.706897 | 1.000000 | 0.568966 | 0.758621 | 0.568966 | 0.844828 | 0.672414 | 0.620690 | 0.931034 | 0.931034 | 0.534483 |
|  | \_08\_ | 0.913793 | 0.637931 | 0.879310 | 0.758621 | 0.913793 | 0.810345 | 0.827586 | 0.931034 | 0.948276 | 0.931034 | 0.948276 | 0.620690 | 0.931034 | 0.586207 | 0.810345 | 0.568966 | 0.689655 | 0.965517 | 0.931034 | 0.741379 |
|  | \_09\_ | 0.913793 | 0.724138 | 0.879310 | 0.793103 | 0.965517 | 0.879310 | 0.896552 | 0.931034 | 0.862069 | 0.965517 | 0.982759 | 0.620690 | 0.931034 | 0.655172 | 0.879310 | 0.672414 | 0.793103 | 0.948276 | 0.948276 | 0.913793 |
|  | \_10\_ | 0.948276 | 0.741379 | 0.896552 | 0.758621 | 0.879310 | 0.879310 | 0.931034 | 0.948276 | 0.931034 | 0.913793 | 1.017241 | 0.793103 | 0.896552 | 0.741379 | 0.982759 | 0.810345 | 0.931034 | 0.948276 | 0.982759 | 0.896552 |

In [72]:

```
exp2_ident_df.describe()
```

Out[72]:

|  | F10\_ | F1\_ | F2\_ | F3\_ | F4\_ | F5\_ | F6\_ | F7\_ | F8\_ | F9\_ | M10\_ | M1\_ | M2\_ | M3\_ | M4\_ | M5\_ | M6\_ | M7\_ | M8\_ | M9\_ |
| --- | --- | --- | --- | --- | --- | --- | --- | --- | --- | --- | --- | --- | --- | --- | --- | --- | --- | --- | --- | --- |
| count | 44.000000 | 44.000000 | 44.000000 | 44.000000 | 44.000000 | 44.000000 | 44.000000 | 44.000000 | 44.000000 | 44.000000 | 44.000000 | 44.000000 | 44.000000 | 44.000000 | 44.000000 | 44.000000 | 44.000000 | 44.000000 | 44.000000 | 44.000000 |
| mean | 0.730016 | 0.422414 | 0.549373 | 0.449060 | 0.467476 | 0.519201 | 0.476097 | 0.570141 | 0.457288 | 0.478056 | 0.753135 | 0.470611 | 0.408307 | 0.467085 | 0.560737 | 0.541928 | 0.375000 | 0.793495 | 0.695925 | 0.384796 |
| std | 0.181590 | 0.228778 | 0.237062 | 0.216496 | 0.292223 | 0.269940 | 0.249636 | 0.241161 | 0.258594 | 0.238596 | 0.261049 | 0.249812 | 0.275405 | 0.170268 | 0.270791 | 0.192438 | 0.240292 | 0.145990 | 0.190984 | 0.261261 |
| min | 0.275862 | 0.034483 | 0.189655 | 0.103448 | 0.068966 | 0.068966 | 0.068966 | 0.120690 | 0.103448 | 0.137931 | 0.120690 | 0.051724 | 0.068966 | 0.120690 | 0.086207 | 0.172414 | 0.068966 | 0.413793 | 0.327586 | 0.051724 |
| 25% | 0.581897 | 0.254310 | 0.327586 | 0.250000 | 0.172414 | 0.275862 | 0.271552 | 0.362069 | 0.219828 | 0.241379 | 0.650862 | 0.241379 | 0.150862 | 0.353448 | 0.336207 | 0.405172 | 0.172414 | 0.737069 | 0.551724 | 0.150862 |
| 50% | 0.801724 | 0.431034 | 0.560345 | 0.491379 | 0.491379 | 0.517241 | 0.465517 | 0.603448 | 0.431034 | 0.474138 | 0.887931 | 0.491379 | 0.344828 | 0.456897 | 0.568966 | 0.568966 | 0.318966 | 0.827586 | 0.706897 | 0.327586 |
| 75% | 0.883621 | 0.625000 | 0.745690 | 0.620690 | 0.702586 | 0.767241 | 0.663793 | 0.775862 | 0.741379 | 0.646552 | 0.931034 | 0.706897 | 0.616379 | 0.586207 | 0.818966 | 0.711207 | 0.538793 | 0.918103 | 0.862069 | 0.568966 |
| max | 0.948276 | 0.741379 | 0.896552 | 0.793103 | 0.965517 | 0.896552 | 0.931034 | 0.948276 | 0.948276 | 0.965517 | 1.017241 | 0.844828 | 0.931034 | 0.775862 | 0.982759 | 0.844828 | 0.931034 | 0.965517 | 0.982759 | 0.913793 |

## perform the shift¶

In [76]:

```
exp2LogFuncTable = peakDf(peakDict(exp2_ident_df))
```

In [77]:

```
exp2Shifted = shiftRaw(exp2_ident_df,exp2LogFuncTable)
```

In [78]:

```
exp2Bins = makeBins(exp2Shifted)
```

In [79]:

```
myPalette=['#4f5b66','#da6b30','#325da7','#ee4035']
```

### spaghetti plots of shifted raw data¶

In [81]:

```
singleShiftPlots(exp2Shifted)
```

```
10_UPPER
```

```
00_UPPER
```

```
00_LOWER
```

```
10_LOWER
```

### plot averaged shifted data¶

In [80]:

```
makeBinPlot(exp2Bins)
```

Re-analysis does not have to stop here. A logical next step would be to apply these shifts to each single response of each participant and re-calculate the results not on the face stimulus but on the participant level. This should give the same means but different (and more valid) estimates of the variance and allow do re-do all analyses.
